# A prognostic six‐gene expression risk‐score derived from proteomic profiling of the metastatic colorectal cancer secretome

**DOI:** 10.1002/cjp2.294

**Published:** 2022-09-22

**Authors:** Javier Robles, Laura Pintado‐Berninches, Issam Boukich, Beatriz Escudero, Vivian de los Rios, Rubén A Bartolomé, Marta Jaén, Ángela Martín‐Regalado, María Jesús Fernandez‐Aceñero, Juan Ignacio Imbaud, José Ignacio Casal

**Affiliations:** ^1^ Protein Alternatives SL Madrid Spain; ^2^ Department of Molecular Biomedicine Centro de Investigaciones Biológicas Margarita Salas, CSIC Madrid Spain; ^3^ Proteomics Core Facility Centro de Investigaciones Biológicas Margarita Salas, CSIC Madrid Spain; ^4^ Pathology Department Hospital Clínico San Carlos (HCSC) Madrid Spain; ^5^ Fundación de Investigación Biomédica del HCSC (FIBHCSC) Madrid Spain; ^6^ Present address: Biochemistry Department Universidad Autónoma de Madrid Madrid Spain

**Keywords:** colorectal cancer metastasis, secreted proteins, risk‐score, prognostic signature, patient stratification

## Abstract

The necessity to accurately predict recurrence and clinical outcome in early stage colorectal cancer (CRC) is critical to identify those patients who may benefit from adjuvant chemotherapy. Here, we developed and validated a gene‐based risk‐score algorithm for patient stratification and personalised treatment in early stage disease based on alterations in the secretion of metastasis‐related proteins. A quantitative label‐free proteomic analysis of the secretome of highly and poorly metastatic CRC cell lines with different genetic backgrounds revealed 153 differentially secreted proteins (fold‐change >5). These changes in the secretome were validated at the transcriptomic level. Starting from 119 up‐regulated proteins, a six‐gene/protein‐based prognostic signature composed of IGFBP3, CD109, LTBP1, PSAP, BMP1, and NPC2 was identified after sequential discovery, training, and validation in four different cohorts. This signature was used to develop a risk‐score algorithm, named SEC6, for patient stratification. SEC6 risk‐score components showed higher expression in the poor prognosis CRC subtypes: consensus molecular subtype 4 (CMS4), CRIS‐B, and stem‐like. High expression of the signature was also associated with patients showing dMMR, CIMP^+^ status, and *BRAF* mutations. In addition, the SEC6 signature was associated with lower overall survival, progression‐free interval, and disease‐specific survival in stage II and III patients. SEC6‐based risk stratification indicated that 5‐FU treatment was beneficial for low‐risk patients, whereas only aggressive treatments (FOLFOX and FOLFIRI) provided benefits to high‐risk patients in stages II and III. In summary, this novel risk‐score demonstrates the value of the secretome compartment as a reliable source for the retrieval of biomarkers with high prognostic and chemotherapy‐predictive capacity, providing a potential new tool for tailoring decision‐making in patient care.

## Introduction

Colorectal cancer (CRC) is considered a heterogeneous disease with different outcomes according to the molecular subtypes [1, 2]. This heterogeneity is reflected in differential epigenetic and genetic events such as microsatellite and chromosomal instability (MSI and CIN), CpG island methylator phenotype (CIMP), and *TP53*, *KRAS*, and *BRAF* mutations (among others) that lead to different pathogenesis and drug sensitivity [3]. This heterogeneity has been addressed by implementing global gene expression classifiers [1, 2, 4, 5]. Still, given the heterogeneity of CRC and the various clinical outcomes, novel and simpler predictive algorithms are necessary to facilitate clinical decision‐making and individually designed management approaches. Current pathological staging presents some predictive limitations, as a significant number of CRC patients relapse after surgical resection and are likely to develop metastasis within 5 years. Particularly necessary is the stratification of stage II and stage III patients to prevent recurrence and to identify those patients who would benefit from more aggressive therapies [6, 7].

We hypothesised that protein expression profiles associated with invasive and metastatic capacity might be useful for building more accurate risk predictors. The conditioned medium or cellular secretome, including exosomes, has been demonstrated to be a rich source of metastatic effectors and biomarkers of metastasis in different tumours [8]. Cancer cells can communicate by secreting soluble factors and/or extracellular vesicles in order to activate fibroblasts and immune cells, and promote extracellular matrix (ECM) remodelling in metastatic progression [9]. Therefore, the use of gene expression profiles derived from secreted proteins might be a suitable alternative to determine risk assessment and recurrence in CRC patients.

For secretome characterisation, cell lines present multiple advantages over whole tumour tissues as they facilitate a more exhaustive and complete recovery of secreted factors. Cell lines constitute a useful resource to recover functional molecular data and design mechanistic studies overcoming the high heterogeneity of human CRC. Multiple studies have confirmed the capacity of cell lines to faithfully represent the molecular subtypes of CRC [10, 11]. Considering this heterogeneity, we have selected three cell lines that represent different molecular subtypes and different liver metastatic capacity, which was determined after intrasplenic injection and survival analysis. Highly metastatic KM12SM and KM12L4 were derived from parental KM12 cells isolated from a patient with Dukes' stage B disease after successive inoculations in mice [12]. SW620 cells were isolated from a metastatic lymph node from a cancer patient and they are poorly metastatic in liver [13, 14]. According to the current CRC molecular classifiers [11], KM12 cells were classified as CMS1, CRIS‐A, and secretory ‘goblet‐like’ cells, whereas SW620 cells were assigned to the CMS4, CRIS‐D, or ‘stem‐like’ subtype. Previous studies suggest that molecular changes associated with CRC progression can be used to predict patient prognosis and response to chemotherapy [15, 16]. Given the vastly different functional and phenotypic properties between both cell lines, deregulated proteins in highly metastatic KM12SM and KM12L4 cells, compared to poorly metastatic SW620 cells, should constitute a rich source of information for the identification of potential prognostic biomarkers in CRC.

To explore the secretome compartment, we performed a combined multi‐omic approach: first, we used a label‐free quantitative proteomic analysis of cell line supernatants, which was validated using a global gene expression analysis of the three cell lines. Then, by using iterative training of hazard ratios (HRs) and survival log‐rank tests in four different datasets from primary tumours containing all the CRC stages, we identified and validated a panel of six genes/proteins with strong prognostic power for stage II/III disease. A risk‐score algorithm was developed and tested with the currently existing CRC molecular classifications. Finally, the predictive value in response to chemotherapy was investigated.

## Materials and methods

### Cell lines

KM12SM and KM12L4 cells were obtained from Dr Fidler (MD Anderson Cancer Center, Houston, TX, USA). SW620 cells were obtained from the ATCC (Manassas, VA, USA). Cells were grown in Dulbeccos Modified Eagle Medium containing 10% fetal bovine serum (Thermo‐Fisher Scientific, Madrid, Spain) and antibiotics at 37 °C in a 5% CO_2_ humidified atmosphere. Cells were regularly tested for mycoplasma contamination and authenticated by short tandem repeat determination.

### Secretome preparation from SW620, KM12SM, and KM12L4 cell lines and label‐free quantification analysis

See Supplementary materials and methods for description of protein preparation and *in silico* analysis. Raw data files have been deposited to the Proteome Xchange Consortium via the PRIDE repository with accession number PXD032899.

### Microarray analysis of differential gene expression

See Supplementary materials and methods for description of the global gene expression analysis. Raw data files have been deposited to the Gene Expression Omnibus repository with the accession number GSE199223.

### Bioinformatics tools

The proteins identified and quantified in the proteomic studies were analysed by systems biology in order to obtain the prediction of the enriched functions. Gene ontology (GO) analysis was performed using g:Profiler web site [17]. Venn diagrams were constructed using InteractiVenn [18]. Unsupervised hierarchical clustering was performed by the Euclidean distance method using Perseus 1.6.14. The Xena platform was used for visualising and interpreting cancer genomics data [19].

### Prognostic analyses using public datasets

Different public gene expression datasets were used for prognosis analysis including datasets from the Gene Expression Omnibus (GSE14333 [20], GSE17538 [21], and GSE39582 [22]) and the TCGA Research Network (COADREAD [23]). The Australian GSE14333 dataset contains clinical and gene expression and disease‐free survival (DFS) data from 290 CRC patients. GSE17538, GSE39582, and COADREAD databases contain 232 CRC, 566 colon cancer, and 736 CRC patients, respectively. These cohorts were also used for TNM staging system classification. Predictive value for chemotherapy treatment was evaluated in the GSE39582 cohort. In addition, datasets GSE72970 [24] and GSE106584 [25] were used in order to increase the number of patients treated with FOLFIRI and FOLFOX. The GSE39582 dataset was also used for information on genome instability and other genetic alterations. CMS subgrouping was performed in the GSE14333, GSE39582, and TCGA COADREAD datasets using the ‘CMSclassifier’ R package [2]. Sadanandam classification of GSE14333 was obtained directly from Sadanandam *et al* [1]. The expression levels for all probes within each sample (patient) were transformed to a *z*‐score value. CRIS classification of the GSE14333, GSE39582, and TCGA COADREAD datasets was obtained from Isella *et al* [5].

### Signature design and risk‐score development

Gene selection for the prognostic signature was sequentially performed using the GSE39582, TCGA, COADREAD and, then, GSE14333 cohorts. Genes with a HR > 1 in GSE39582 were investigated for a significant log‐rank *P* value by applying the median cut‐off method in the TCGA COADREAD. Then, selected genes were asked to have a significant HR > 1 in GSE14333 for validation. For risk‐score development, the GSE14333 database was used. In brief, the risk‐score for each patient was calculated as the sum of each gene's score, which is derived by multiplying the normalised expression level of each gene by its corresponding coefficient Risk score = ∑ *β*_*i*_ × *E*_*i*_ (*β*_*i*_ is the Cox regression coefficient of gene *G*_*i*_ and *E*_*i*_ is the expression value of gene *G*_*i*_). Then, patients were divided into two groups (i.e. high or low risk) by the optimal cut‐off method using X‐tile software [26]. The optimal cut‐off was defined as the point with the most significant log‐rank test split to achieve the lowest *P* value. Gene prognostic signature and risk‐score classification were validated in the GSE39582, TCGA COADREAD, and GSE17538 datasets.

### Statistical analysis

Univariate and multivariate Cox regression analysis were performed using ‘survival’ and ‘survminer’ R packages. Forest plot representation was obtained using the ‘ggplot2’ R package. Kaplan–Meier analysis was performed using OriginPro Version 2020 (OriginLab Corporation, Northampton, MA, USA). The significance of gene expression differences between groups was obtained by using two‐sample *t* tests for each protein or gene. ANOVA tests were performed in order to detect significant differences in risk‐score between three or more groups. *F* statistic and *P* value are shown.

## Results

### Quantitative label‐free proteomic characterisation of the metastatic secretome

**Figure 1:**
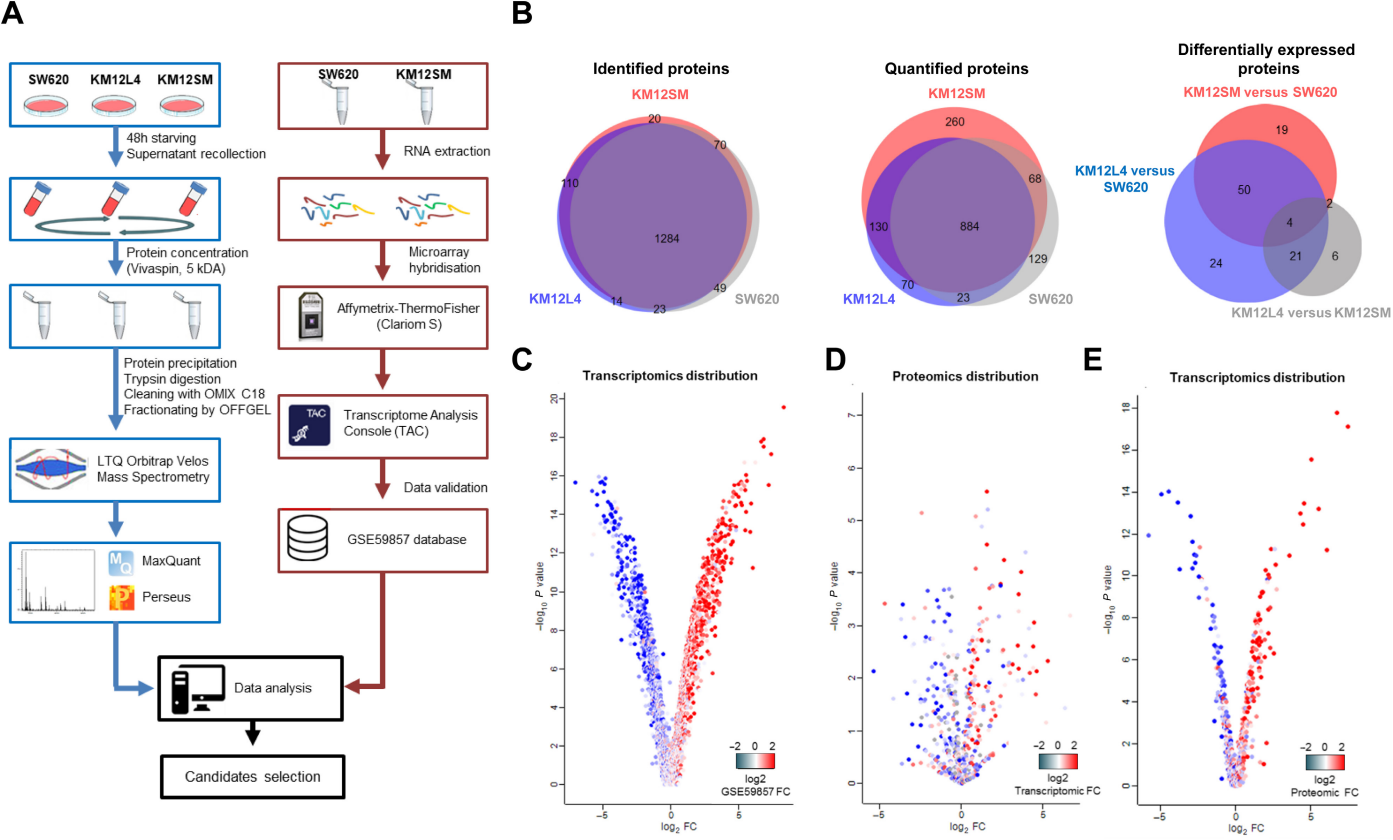
Work‐flow, quantified proteins, and correlations between proteomic and transcriptomic results. (A) Work‐flow scheme of label‐free proteomic analysis of secreted proteins from SW620, KM12SM, and KM12L4 cells and the corresponding transcriptomic experiments. (B) Proportional Venn diagrams of the identified, quantified, and differentially expressed proteins in the secretome fractions of the SW620, KM12SM, and KM12L4 cell lines. (C) Volcano plot distribution of transcriptomic data (KM12SM/SW620) according to the fold‐change obtained from GSE59857 for the same cell lines. Both gene expression studies, internal and dataset, showed an excellent correlation and were used indistinctly. (D) Volcano plot distributions of proteomics results coloured according to transcriptomic data and (E) transcriptomic results distribution represented on proteomic data.

The biomarker discovery workflow is detailed in Figure 1A. Protein extracts from concentrated supernatants of each cell line were trypsin‐digested and peptides separated into six fractions using an OFFGEL instrument (Agilent, Santa Clara, CA, USA). Mass spectrometry results were quantified using MaxQuant LFQ and Perseus. Comparison of the intensity values for each of the quantified proteins among all triplicates and the analysis of the linear correlation between each comparison revealed a high reproducibility of the quantitative data (supplementary material, Figure S1A). Furthermore, the histograms of the distributions of the LFQ log_2_ values of the quantified proteins in each triplicate showed a similar normal distribution pattern, confirming the robustness of the proteomic analysis (supplementary material, Figure S1B). Principal component analysis (PCA) of the three replicates for each cell line confirmed that KM12SM and L4 cells clustered together, whereas SW620 cells clustered apart (supplementary material, Figure S1C). In total, 1,570 proteins were identified and 1,564 were quantified in the secretome of the three cell lines (supplementary material, Table S1). Most of the identified and quantified proteins, 1,284 and 884 proteins, respectively, were common to the three cell lines (Figure 1B). However, 260, 70, and 129 proteins were exclusively quantified in the secretome of KM12SM, KM12L4, and SW620, respectively (Figure 1B). Among the quantified proteins, 153 proteins (119 up‐regulated and 34 down‐regulated) were differentially secreted between KM12 (L4 and SM) and SW620 cells with a fold‐change ≥5 (*P* value ≤0.05) (supplementary material, Table S2). Location of the quantified and identified proteins reveals a combination of secreted and cellular proteins likely derived from exosomes and microvesicles (supplementary material, Figure S1D). GO analysis of the 119 up‐regulated proteins in the KM12 cells showed that ECM constituent, cell adhesion molecule binding, calcium ion binding, and exocytosis are among the most significantly biological functions (supplementary material, Figure S1E). Down‐regulated proteins were related to cell migration, including cell polarity, podosome assembly, actin binding, and granulocyte activation.

### Gene expression analysis and correlation with proteomic data

For an initial validation of the expression alterations observed in the secreted proteins of the three cell lines, we combined in‐house transcriptomic analysis of KM12SM and SW620 cells (GSE199223) with the publicly available GSE59857 dataset, which contains the gene expression analysis for 155 CRC cell lines [10]. Quality control of our transcriptional study indicated a robust Pearson correlation coefficient (supplementary material, Figure S2A) and normal distribution of the histograms of the signal intensities corresponding to the gene expression (supplementary material, Figure S2B). We observed an excellent agreement between our transcriptomic analysis and the GSE59857 results, supporting a direct data comparison between both datasets (Figure 1C). PCA confirmed a similar clustering of the cell lines by using either our transcriptomic data (supplementary material, Figure S2C) or the GSE59857 dataset values (supplementary material, Figure S2D). Gene expression differences between KM12SM and SW620 are shown in supplementary material, Table S3. Although the overlapping of the proteomic data over the global transcriptomic analysis revealed a weaker match, likely as a consequence of a lower representation of proteins versus identified genes (Figure 1D), a distribution analysis of the transcriptomic values on the proteomics data indicated an excellent overlap between protein and mRNA alterations for the identified secreted proteins (Figure 1E). Therefore, gene expression results validated the protein alterations identified in the secretome analysis.

### Discovery, training, and validation of prognostic biomarkers

**Figure 2:**
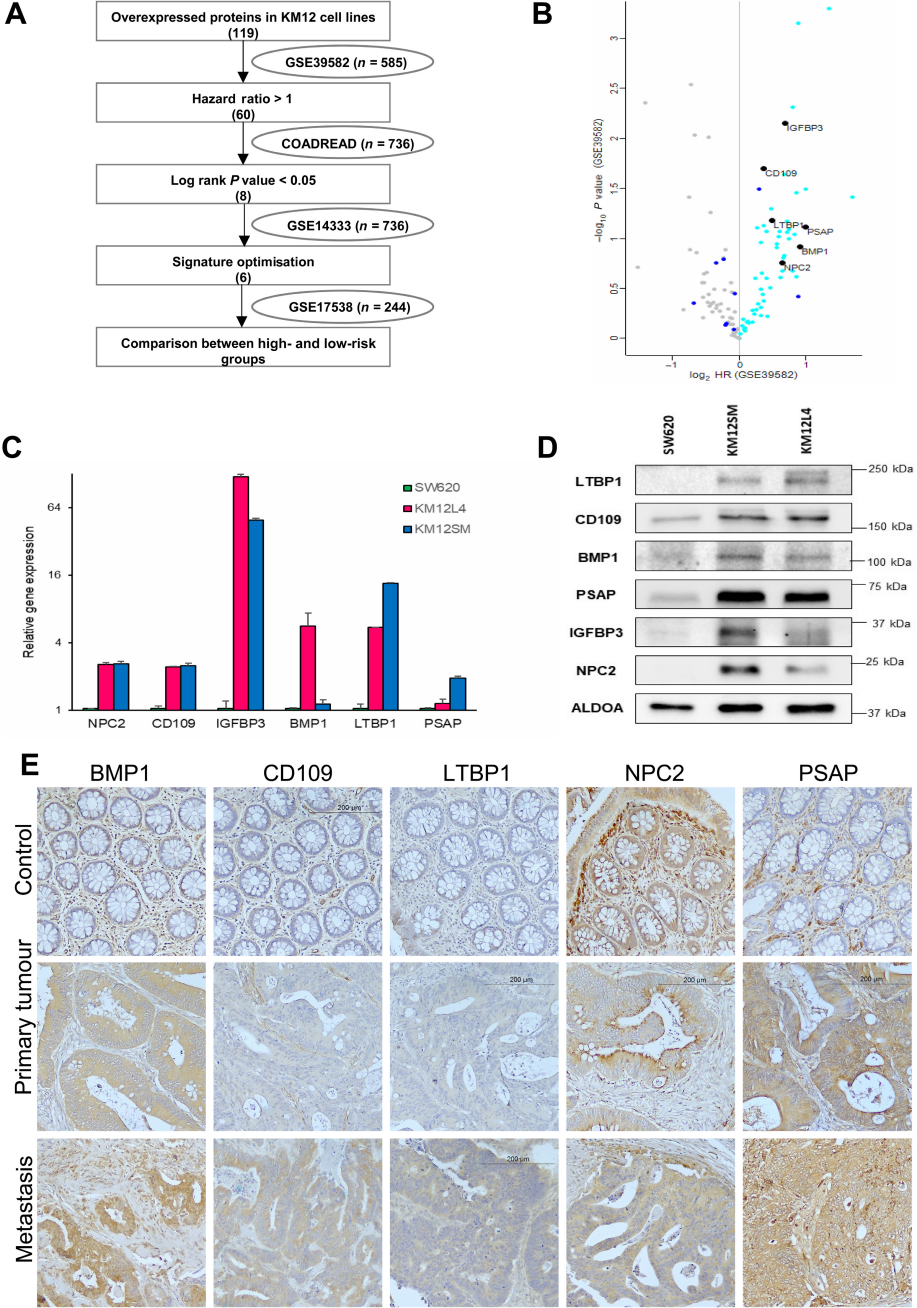
Discovery and validation of the gene‐based prognostic signature. (A) Flow‐chart representation of sequential prognostic signature selection using different datasets (GSE39582, TCGA COADREAD, GSE14333, and GSE17538). (B) Volcano plot of the overexpressed proteins in KM12SM and/or KM12L4 compared to SW620 cell lines. Distribution corresponds to the GSE39582 cohort data. Colour labelling corresponds to GSE39582 HR (cyan), COADREAD log‐rank *P* value (blue), and GSE14333 HR *P* value (black). Genes corresponding to the SEC6 proteins genes, which fit the threshold, are indicated. (C) Colorectal cancer cell lines KM12SM, KM12L4, and SW620 were subjected to qPCR using specific primers for NPC2, CD109, IGFBP3, BMP1, LTBP1, and PSAP. (D) Western blot analysis of the secreted fractions using specific primary antibodies. Secreted aldolase (ALDOA) was used as a loading control. (E) Representative immunohistochemical images of control colon, primary tumour, and liver metastasis tissues from CRC patients using antibodies against BMP1, CD109, LTBP1, PSAP, and NPC2 and counterstained with haematoxylin. Images were taken at ×200 magnification.

Then, those 119 genes corresponding to the up‐regulated proteins were investigated for their prognostic value according to the REMARK guidelines [27]. Four different datasets were consecutively used for discovery, training, and validation of potential prognostic biomarkers (Figure 2A). For the initial selection, we used, as discovery dataset, the CIT cohort (GSE39582, *n* = 566) containing stage and relapse information. Sixty of 119 genes were found to have a HR > 1, according to the Cox model estimator (Figure 2A and supplementary material, Table S4). Training of the 60 genes in the TCGA COADREAD database (*n* = 736) resulted in the selection of 8 genes with a log‐rank *P* value <0.05 (Figure 2A). Final validation using the GSE14333 dataset (AUS cohort, *n* = 290 patients) resulted in the selection of six genes (IGFBP3, CD109, LTBP1, PSAP, BMP1, and NPC2) showing a HR > 1 with *P* value <0.05 (Figure 2B and supplementary material, Table S4). Moreover, the six genes consistently showed significant HRs in four datasets (supplementary material, Table S5). As a further validation, the expression of these markers in the metastatic cells and tissues was tested by qPCR, western blot, and immunohistochemistry. Significant differences in gene expression between KM12 and SW620 cells were confirmed by qPCR, with IGFBP3 and PSAP showing the highest and lowest expression, respectively (Figure 2C). LTBP1, CD109, BMP1, IGFBP3, PSAP, and NPC2 protein overexpression in the cell line supernatants was confirmed by western blot (Figure 2D). Immunohistochemical staining was performed to visualise the expression of BMP1, CD109, LTBP1, PSAP, and NPC2 in human control colonic tissue, primary tumour, and liver metastasis. IGFBP3 was not tested due to the lack of suitable antibodies. Significant differences were found in the location and expression levels of the five biomarkers. BMP1, CD109, and LTBP1 showed a gradual and clear increase of expression from the control tissues to the metastatic tissues, with preferential cytoplasmic staining in the tumour tissue, being more intense in metastasis. NPC2 gave a distinctive pattern with strong staining of stromal vessels (likely endothelium) in the periphery of the control colonic tissue and some weak cytoplasmic staining in the crypts. In the primary tumour, NPC2 showed preferential membrane staining of the tumour, being more cytoplasmic in the metastasis. PSAP showed stromal but no epithelial staining in the normal colon. However, primary tumours exhibited clear cytoplasmic staining that was more pronounced in the metastatic tissues, including the stromal compartment (Figure 2E). Collectively, these results indicate higher epithelial expression of the five biomarkers in metastasis, although NPC2, PSAP, and BMP1 also showed stromal staining, mainly in normal colon.

### SEC6 risk‐score classifier development and validation

**Figure 3:**
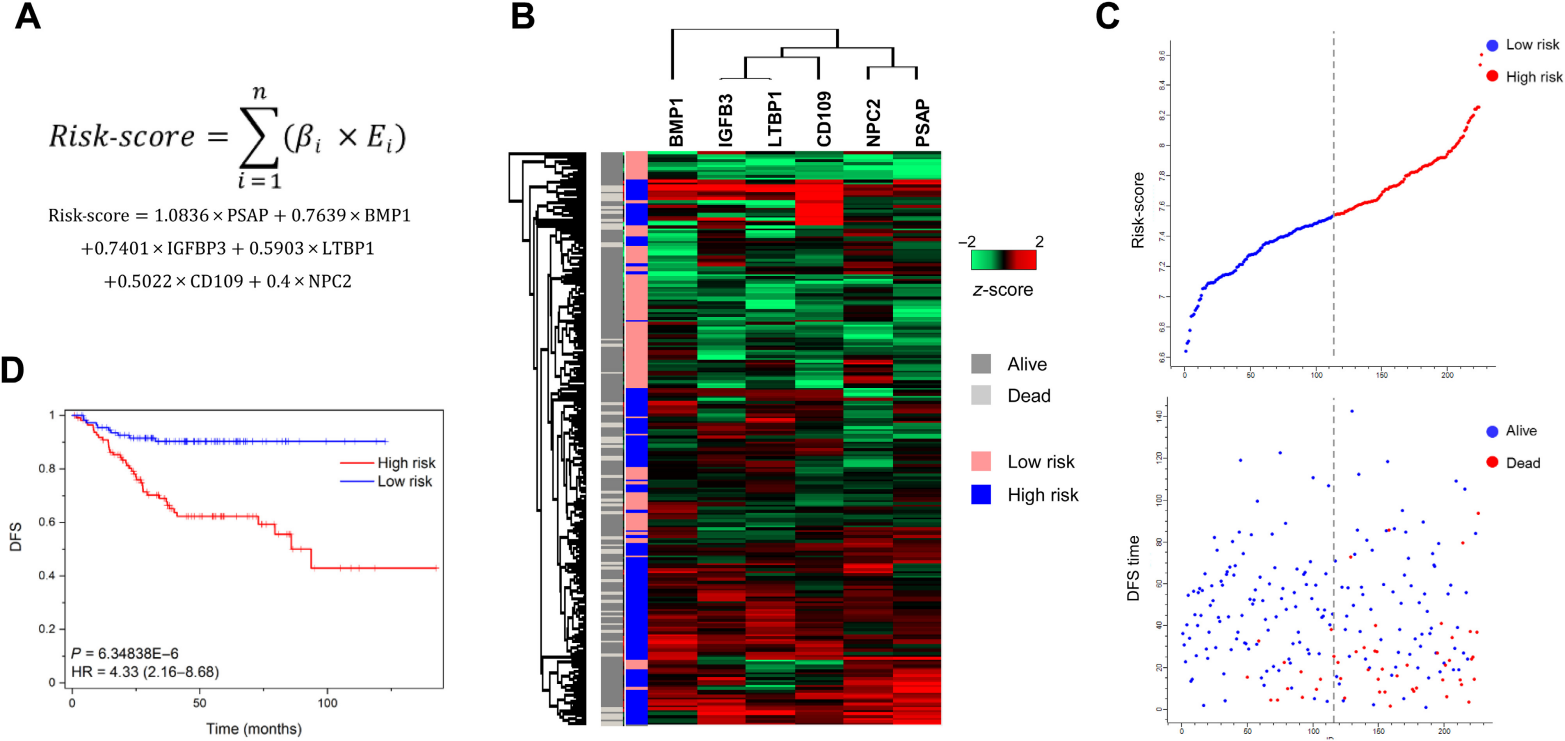
Risk‐score development and validation in the GSE14333 cohort. (A) Risk‐score calculation formula. *β* is the regression coefficient (univariate Cox model) and *E* is the normalised expression value for each gene. (B) Hierarchical clustering of mRNA expression in GSE1433. OS events (alive/dead) and low–high risk distribution are shown. (C) Risk‐score distribution and corresponding survival status. (D) Kaplan–Meier analysis of high‐ and low‐risk patients. HRs were determined according to the Cox regression model. *P* values were obtained by log‐rank test.

Next, a risk‐score classifier algorithm was developed according to the regression coefficients and normalised expression values for each of the six genes (SEC6) using the GSE14333 dataset (Figure 3A), as described in the ‘Materials and methods’ section. Patients were divided into two groups (i.e. high or low risk) by the optimal cut‐off method using X‐tile software [26]. Unsupervised hierarchical clustering showed a robust correlation between high expression of the six markers and high risk of the patients as well as a good association between high risk and dead events in the GSE14333 cohort (Figure 3B). The risk‐score was validated using the GSE17538 dataset (supplementary material, Figure S3A). The risk‐score distribution showed that the high‐risk subset of patients presented poorer survival than the low‐risk subgroup (Figure 3C and supplementary material, Figure S3B). In agreement with these data, patients were correctly stratified according to high and low risk using Kaplan–Meier analyses in both datasets, HR: 2.56, 95% CI (1.69–3.87), *P* value: 3.67E−6 and HR: 4.33, 95% CI (2.16–8.69), *P* value: 6.34E−6, respectively (Figure 3D and supplementary material, Figure S3C).

Then, we evaluated the independence of the SEC6 classifier using the GSE14333 and GSE17538 datasets (Table 1). In the GSE14333 cohort, the association between SEC6 and other potential risk factors was supervised by univariate and multivariate Cox regression analysis. Stage, chemotherapy, and SEC6 expression were found to be significant risk factors for DFS in univariate analysis, while age, gender and location were not. By multivariate Cox regression analysis, the tumour stage and the SEC6 classifier were independent risk factors: HR: 3.231, 95% CI (1.62–6.46), *p* < 0.001 and HR: 3.527, 95% CI (1.74–12.44), *P* value: 4.56E−04, respectively. In a similar way, tumour grade, American Joint Committee on Cancer (AJCC) stage, and SEC6 risk classification were independent risk factors for overall survival (OS) in the GSE17538 cohort in the multivariate analyses (Table 1).

**Table 1:** Univariate and multivariate Cox regression analysis of DFS in GSE14333 and OS in GSE17538

GSE14333					
		Univariate		Multivariate	
Variable	*n*	HR (95% CI)	*P* value	HR (95% CI)	*P* value
Gender (male or female)	226	0.908 (0.52–1.59)	0.736		
Age	226	0.981 (0.96–1.01)	0.069		
Location (left, right, rectum)	226	0.831 (0.67–1.11)	0.223		
Duke's stage (A, B, or C)	226	2.949 (2.03–6.68)	4.39E−04	3.231 (1.62–6.46)	0.001
Chemotherapy (yes or no)	226	1.892 (1.09–3.30)	0.025	1.189 (0.44–1.60)	0.597
Risk classification (high risk or low risk)	226	4.334 (2.16–8.86)	3.49E−05	3.527 (1.74–12.44)	4.56E−04

GSE17538					
		Univariate		Multivariate	
Variable	*n*	HR (95% CI)	*P* value	HR (95% CI)	*P* value
Gender (male or female)	239	1.006 (0.67–1.52)	0.975		
Age	239	1.008 (0.99–1.02)	0.304		
Ethnicity (Caucasian or other)	239	0.651 (0.30–1.41)	0.277		
Grade (WD, MD, or PD)	239	2.339 (1.40–3.92)	1.27E−03	1.807 (1.07–3.04)	0.026
AJCC stage (I, II or III, IV)	239	3.696 (2.23–6.13)	3.97E−07	3.255 (1.95–5.43)	6.03E−06
Risk classification (high risk or low risk)	239	2.096 (1.30–3.39)	2.51E−03	1.781 (1.06–2.79)	0.0283

### SEC6 expression correlates with CRC molecular classifiers and specific genetic events

**Figure 4:**
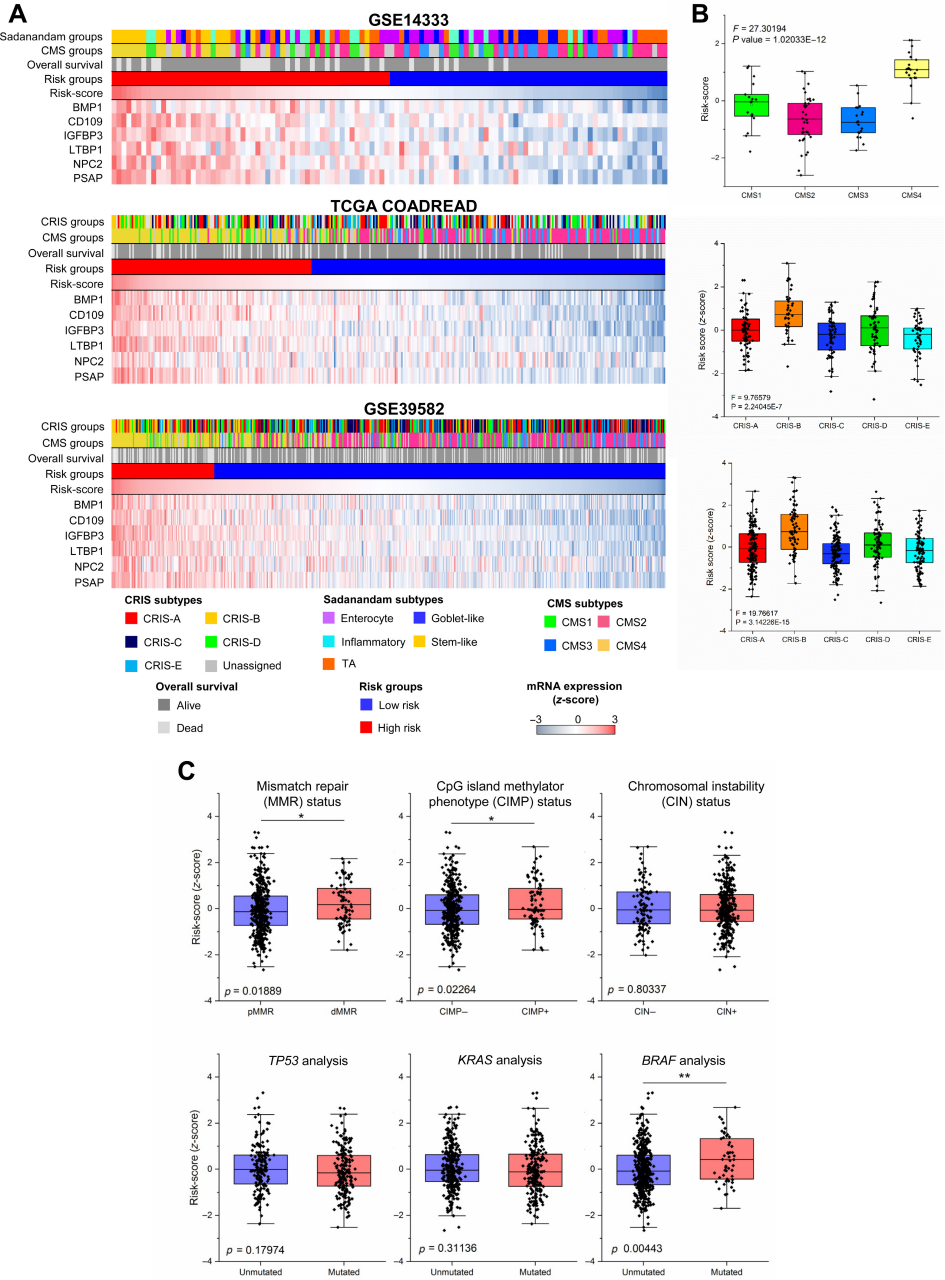
Risk‐score correlates with current colorectal cancer classifications. (A) Patients from GSE14333, TCGA COADREAD, and GSE39582 cohorts were subjected to supervised clustering according to the risk‐score value. Sadanandam, CMS, and CRIS subtypes distribution together with OS are represented for comparative purposes. mRNA and risk‐score values expression (*z*‐score) were coloured according to its determined value. (B) Risk‐score (*z*‐score) distribution according to the CMS and the CRIS classifiers in GSE14333 and TCGA COADREAD and GSE39582 databases, respectively. (C) SEC6 expression according to MMR status, CIMP status, chromosomal instability and *TP53*, *KRAS*, and *BRAF* mutations.

The association between SEC6 expression and current CRC subtype classifiers Sadanandam, CMS, and CRIS was explored using the GSE14333, TCGA COADREAD, and GSE39582 cohorts. Clustering of the patients according to risk‐score values revealed a clear association between SEC6‐positive expression, high‐risk prediction in the three classifiers, and dead events in the three cohorts (Figure 4A). SEC6‐positive expression correlated with CMS4 and CRIS‐B patients who exhibited the highest risk‐score, followed by CMS1 and CRIS‐A (Figure 4B and supplementary material, Figure S4A). It is of note that CMS4 and CRIS‐B are associated with worse prognosis [2, 5]. The highest risk‐score also correlated with the poor survival‐associated stem‐like and inflammatory subtypes [1] (supplementary material, Figure S4B). To further support this notion, we carried out an analysis of the concordance between risk groups and CMS and CRIS subtypes using a Caleydo view (supplementary material, Figure S4C). A high level of concordance was observed between the high‐risk group and the CMS4 and CMS1 subtypes. Also, CRIS‐B and CRIS‐A contributed mostly to the high‐risk group. Therefore, SEC6‐stratified high‐risk patients showed a clear enrichment in those subtypes commonly associated with worse prognosis in three different classifications. Then, we explored the association of the SEC6‐based risk prediction with some common genetic events observed in CRC patients (GSE39582 cohort). Deficient mismatch repair status (dMMR) (*P* value = 0.018), CIMP^+^ status (*P* value = 0.022), or the presence of *BRAF* mutations (*P* value = 0.004) were significantly associated with higher expression of the SEC6 genes (Figure 4C). These results agree well with previous studies showing a poor prognosis for patients displaying dMMR and *BRAF* mutations [28, 29]. In contrast, mutations in *TP53* or *KRAS*, or chromosomal instability status, were not significantly associated with higher risk according to SEC6 expression.

### Molecular events underlying the risk‐score classifier

To better understand the molecular mechanisms underlying the risk‐score classifier, we investigated genes expressed differentially between the high‐ and low‐risk subgroups in the GSE39582 cohort using *in silico* approaches. Up‐regulated genes in high‐risk SEC6‐positive patients were significantly enriched in extracellular region and cell periphery location, whereas down‐regulated genes corresponded to intracellular proteins (supplementary material, Figure S5A). Most up‐regulated genes in high‐risk groups were linked with cell differentiation, ECM organisation, cell adhesion, and migration and developmental process. In contrast, down‐regulation was observed in DNA replication, regulation of cell cycle, and chromosome organisation genes (supplementary material, Figure S5B). A heat map representation indicates the association of genes involved in cell adhesion, migration, and ECM organisation with high SEC6 expression, whereas cell cycle protein expression was higher in the low‐risk subgroup (supplementary material, Figure S5C). A list of genes associated with high and low risk is given in supplementary material, Table S6.

### SEC6 expression associates with poor prognosis in stage II and III patients

**Figure 5:**
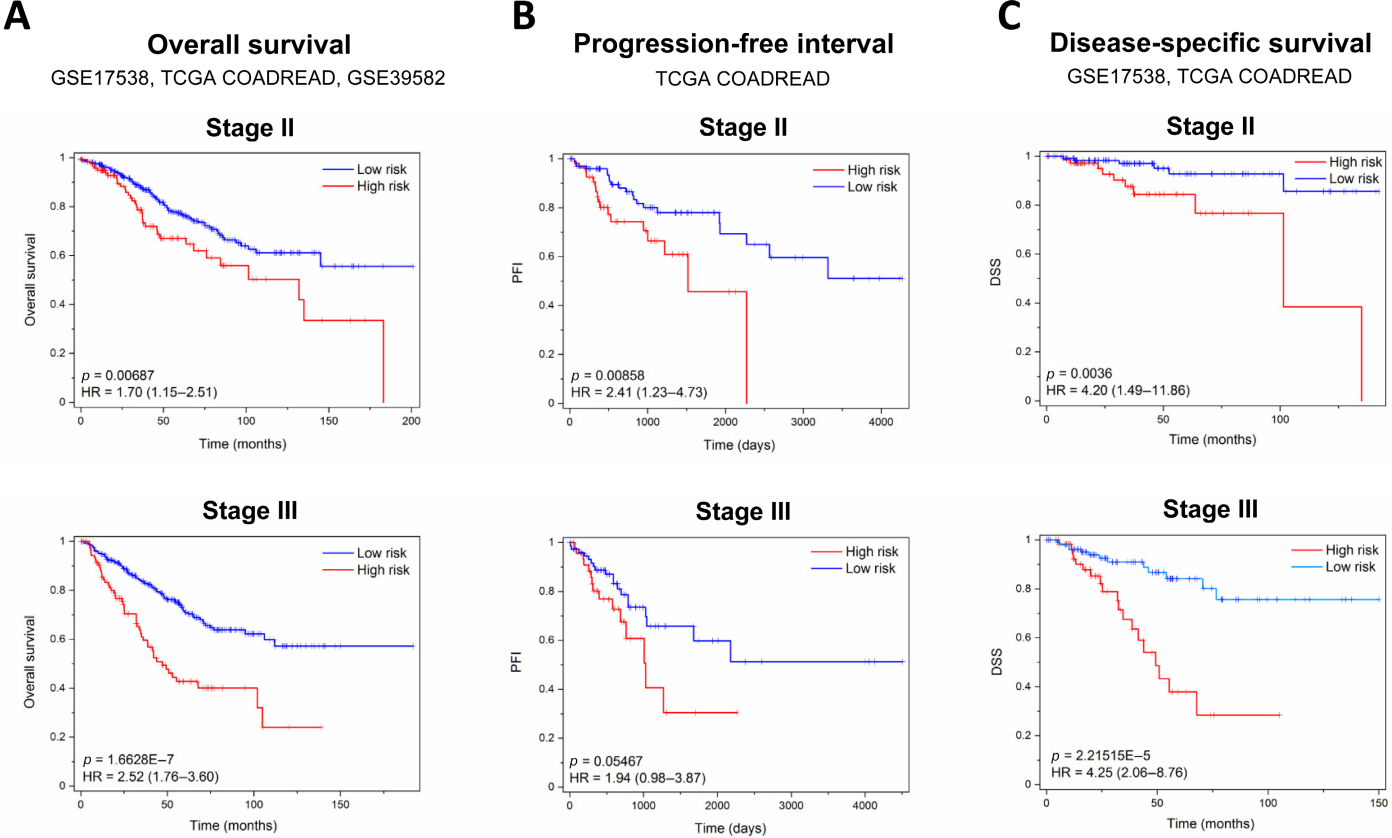
High SEC6 expression is associated with lower OS, PFI, and disease‐specific survival (DSS). (A) OS analysis in the pooled cohorts GSE17538, TCGA COADREAD, and GSE39582. (B) PFI analysis in the TCGA COADREAD database. (C) DSS analysis in GSE17538 and TCGA COADREAD datasets. All determinations were made for stage II and III patients using Kaplan–Meier plots. HRs were determined according to the Cox regression model. *P* values were obtained by log‐rank test.

The capacity of SEC6 to identify patients with poor prognosis at stage II and III was evaluated using Kaplan–Meier curves. We investigated the survival capacity of SEC6‐positive and SEC6‐negative subgroups in a meta‐dataset (*n* = 1,534 patients) representing the sum of GSE17358, GSE39582, and TCGA COADREAD (Figure 5) or in each individual cohort (supplementary material, Table S7). OS analysis in the meta‐dataset showed the capacity of SEC6 to correctly classify the samples as high and low risk for stage II and III patients. The estimated HR was higher for stage III patients, HR: 2.52, 95% CI (1.76–3.60), *P* value: 1.66E−7 than for stage II patients, HR: 1.70, 95% CI (1.15–2.51), *P* value: 0.00687 (Figure 5A). Progression‐free interval analysis using the TCGA COADREAD indicated a slightly higher HR for stage II than stage III, HR: 2.41, 95% CI (1.23–4.71), *P* value: 0.0085 (stage II) and HR: 1.94, 95% CI (0.96–3.57), *P* value: 0.05 (stage III) (Figure 5B). Finally, disease‐specific survival prognostic capacity in two cohorts, GSE17538 and TCGA COADREAD, showed equally high HRs for both stages: HR: 4.20, 95% CI (1.49–11.86), *P* value: 0.0036 (stage II) and HR: 4.25, 95% CI (2.06–8.76), *P* value: 2.21E−5 (stage III) (Figure 5C). The results for the individual datasets showed that SEC6‐positive tumours were associated with a lower rate of survival and higher recurrence probabilities than SEC6‐negative tumours in the three datasets (supplementary material, Table S7). These studies confirm the value of the secreted protein‐based signature in the prediction of patient outcome at early CRC stages.

### High‐risk subgroups require aggressive adjuvant chemotherapy

**Figure 6:**
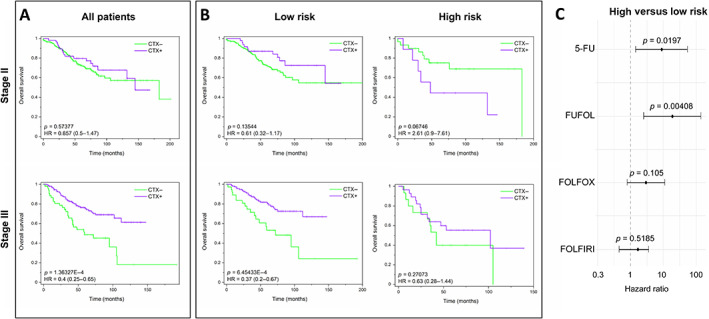
SEC6‐predicted high‐risk subgroups require more aggressive chemotherapy. Kaplan–Meier plots of (A) OS for all combined patients receiving 5‐FU or FUFOL chemotherapy (CTX+) or not (CTX−) according to the AJCC stage (II or III) and (B) OS for high‐ and low‐risk subgroups after receiving chemotherapy (CTX+) or not (CTX−). HRs were determined according to the Cox regression model. *P* values were obtained by log‐rank test. (C) Forest plots of HRs associated to each treatment. *P* values were obtained by Cox regression analysis. Patient data were obtained from GSE39582 (5‐FU, FUFOL), GSE39582 and GSE72970 (FOLFIRI), and GSE39852, GSE72970, and GSE106584 (FOLFOX) cohorts.

Finally, we explored the association between SEC6 expression and response to adjuvant chemotherapy for stage II and III patients using the GSE39582 cohort. Most of these patients received only 5‐FU combined with calcium folinate (FUFOL), whereas the number of patients who received more aggressive treatments (FOLFIRI or FOLFOX) was much lower. When high and low‐risk patients were examined together, stage III (but not stage II) patients showed a significant improvement in OS after chemotherapy (Figure 6A). However, when patients were divided into risk subgroups, only SEC6‐negative, low‐risk, and stage III patients showed improved survival after FUFOL chemotherapy: HR: 0.37, 95% CI (0.2–0.67), *P* value: 6.45E−4. In contrast, high‐risk stage II and III patients did not significantly benefit from the use of FUFOL (Figure 6B), suggesting that FUFOL is insufficient for the treatment of SEC6‐positive, high‐risk patients. Then, we used a forest plot to determine the HRs in high‐risk versus low‐risk subgroups after receiving 5‐FU, FUFOL, FOLFOX, or FOLFIRI (Figure 6C). In 5‐FU and FUFOL‐treated patients, as HR > 1 and *P* values <0.05, high‐risk patients showed shorter survival than low‐risk patients. FOLFOX‐ and FOLFIRI‐treated patients showed lower HRs, suggesting that these treatments were more effective for high‐risk patients. In summary, only more aggressive therapies are likely to cause increased survival in high‐risk early‐stage patients, although low‐risk patients will benefit from receiving 5‐FU‐based treatments.

## Discussion

The necessity to predict recurrence and clinical outcome in early stage CRC is critical to identify those patients who may benefit from adjuvant chemotherapy and for the implementation of therapeutic guidelines for histologically similar tumours. An adequate stratification of stage II and III patients should facilitate a continuous follow‐up and adequate chemotherapy administration regardless of surgical approaches. Here, we have developed a prognostic six gene‐based signature (SEC6) following a combination of proteomic and transcriptomic analyses using a three‐step approach. First, 119 secreted proteins were identified as up‐regulated in metastatic versus non‐metastatic cells after the proteomic analysis of soluble factors. Second, differential expression of secreted proteins was validated at the transcriptional level using global gene expression analyses. Third, an iterative analysis of HRs and log‐rank tests for the 119 genes through 4 different datasets that included a total of 1,855 patients revealed a signature of six genes: IGFBP3, CD109, LTBP1, PSAP, BMP1, and NPC2 with robust prognostic power. Their overexpression was confirmed in metastatic cell lines and tissues. Our studies indicate that SEC6‐positive tumours were associated with lower OS and higher recurrence rates than SEC6‐negative tumours across four independent datasets. Prognostic value was independent of sex, age, or location of the tumours.

Given the heterogeneity of CRC, different CRC classifications have been developed that associate with different outcomes and responses to chemo‐ and biological therapies [30]. According to the CRIS and CMS classifiers, SEC6 expression preferentially correlates with CRIS‐B and CMS4, the more aggressive subtypes in three different datasets, as well as with the stem‐like and the inflammatory subtypes. In addition, SEC6‐positive expression consistently predicted high‐risk patients showing characteristics of stem‐cell signature, MSI, dMMR, and CIMP^+^ status as well as *BRAF* mutations, which are commonly associated with worse prognosis in CRC [31, 32]. It is of note that the CIMP phenotype is tightly associated with *BRA*F mutations in CRC [33]. Although dMMR patients usually have better prognosis, those whose tumours contain *BRAF* mutations have shown worse prognosis in metastatic CRC [29, 31].

Microarray‐derived gene expression signatures have shown great potential for patient stratification. However, the large number of genes usually involved in these signatures complicates the translation of these findings to clinically useful tests. Current tests are based on a limited number of genes, e.g. the seven‐gene Oncotype DX recurrence score [34]. Therefore, one added value of this SEC6 signature is that only six genes/proteins provide sufficient predictive and prognostic capacity. Our SEC6 signature is composed of proteins likely relevant for metastatic progression, colonisation, and metabolic adaptation. High expression of IGFBP3 (insulin‐like growth factor binding protein 3) has been associated with lymph node and liver metastasis, and poor outcome in CRC [35, 36] and pancreatic endocrine neoplasms [37]. Other groups reported the positive association of IGFBP3 gene methylation with recurrence of stage II CRC patients [38]. PSAP (Prosaposin) participates in the lysosomal degradation of sphingolipids that function as effector molecules in cell signalling and the regulation of multiple cellular processes [39]. Still, no clear association of PSAP with prognosis has been described, except for glioblastoma [40]. BMP1 (bone morphogenetic protein 1) is a metalloprotease involved in the formation of ECM, including proteolysis of collagens and activation of lysyl oxidase. High BMP1 expression has been associated with poor prognosis in gastric cancer [41]. Another secreted protein, NPC2 (Niemann‐Pick C2 protein) regulates the transport of cholesterol through the late endosomal/lysosomal system and has not been previously associated to prognosis or metastasis [42]. CD109 is a glycophosphatidylinositol‐anchored membrane glycoprotein characterised as a component of the receptor complex of TGFβ [43]. In lung cancer metastasis, CD109 expression led to the activation of the Jak‐Stat3 signalling pathway [44]. CD109 expression enhances stromal TGFβ activation in the presence of LTBP1 [45], another regulator of TGFβ. An association between CD109 expression and OS in other types of cancer has been described [46].

Regarding chemotherapy response, our results indicate that SEC6‐positive, high‐risk, stage II/III patients require aggressive therapies such as FOLFOX or FOLFIRI and they do not benefit from first‐line 5‐FU therapy in contrast to low‐risk patients. Although our SEC6 predictor needs to be further validated in larger cohorts of chemotherapy‐receiving patients and prospective analyses, SEC6 recapitulates the prognostic information to identify high‐risk CRC subtypes and facilitate the development of novel clinical tools for a correct patient stratification. The low number of biomarkers involved may simplify the development of clinical tools for predicting patient survival and personalising therapies according to the molecular characteristics of the tumours. In summary, these results confirm that the secretome analysis of aggressive metastatic cells constitutes a rich mine of information for the discovery of new prognostic and predictive biomarkers.

## Author contributions statement

JIC and JR designed the study and wrote the manuscript. JR, LPB, IB, BE, VR, RAB, MJ and AMR performed the experiments. JIC, JR, VR, MJFA and JII analysed the data. JIC supervised the whole study. JIC and JII participated in the acquisition of funding.

## Supporting information


Supplementary materials and methods

**Figure S1.** Quality control and gene ontology analysis of the proteomic assay
**Figure S2.** Quality control of the gene expression analysis
**Figure S3.** Validation of the six‐gene risk‐score in GSE17538
**Figure S4.** Association of risk score‐based classification with current colorectal cancer classifications
**Figure S5.** Analysis of differentially expressed genes between high‐ and low‐risk patients in GSE39582

**Table S1.** List of deregulated proteins identified and quantified in the secretome of KM12SM, KM12L4, and SW620

**Table S2.** Up‐ and down‐regulated proteins in KM12L4 and/or KM12SM compared with SW620

**Table S3.** List of gene alterations between KM12SM and SW620 cells

**Table S4.** Determination of HRs and long rank *P* values for the up‐regulated secreted proteins in four CRC datasets

**Table S5.** HRs of the six selected genes (SEC6) in different datasets

**Table S6.** List of genes associated to cell migration, adhesion, EC organisation, and cell cycle in high‐risk CRC patients

**Table S7.** HRs (95% CI) of high‐ and low‐risk patients classified by stages in survival analyses

## Data Availability

All expression profiling transcriptomic data are publicly available in the Gene Expression Omnibus (GEO) repository under the GEO accession number GSE199223 (https://www.ncbi.nlm.nih.gov/geo/query/acc.cgi?acc=GSE199223). Quantitative proteomic data are publicly available in the PRIDE repository under accession number PXD032899 (http://proteomecentral.proteomexchange.org/cgi/GetDataset?ID=PXD032899).
