# Supplementary material for: A prognostic six‐gene expression risk‐score derived from proteomic profiling of the metastatic colorectal cancer secretome
Source: J Pathol Clin Res. 2022 Sep 22;8(6):495–508. doi: 10.1002/cjp2.294 (PMC9535096; doi:10.1002/cjp2.294)
Supplement: Supplementary file 1 — Supplementary materials and methods [file CJP2-8-495-s007.docx]

**A prognostic six-gene expression risk-score derived from proteomic profiling of the metastatic colorectal cancer secretome**

J Robles *et al*. *J Pathol Clin Res* DOI: <https://doi.org/10.1002/cjp2.294>

**Supplementary Materials and Methods**

**Reference numbers refer to the list in the main paper**

**Ethics approval statement**

All the tissue samples were obtained from the Surgical Pathology Department of the Hospital Clínico (Madrid) after approval of the Research Ethics Committee of the Hospital.

**Secretome preparation from SW620, KM12SM and KM12L4 cell lines**

To obtain the secretome, cells were seeded at a density of 6 x 10^6^ cells per plate. Twenty-four hours later, cells were washed with PBS to remove serum and cell debris and cultured with serum-free DMEM for 48 h. Then, medium was collected, centrifuged to remove cell debris and concentrated using Vivaspin devices (Sartorius, Göttingen, Germany), at 6000 g for 1 h at 4° C.

**Label-free quantification of secreted proteins**

Concentrated supernatants containing 50 µg of protein were precipitated with 5 vol of acetone overnight at -20 ° C. Next day, the samples were resuspended and cleaned with OMIXs C18. Samples were reconstituted in 5% glycerol and 2% IPG, pH 3.0-10 (GE Healthcare) and loaded onto 12-well isoelectric focusing strips (13 cm, ImmobilineDryStrip, pH 3.0-10) (GE Healthcare, Madrid, Spain) for OFFGEL fractionation. A total of 6 fractions were obtained from each sample and cleaned again with OMIXs before being reconstituted in 5 μL of a 0.1% FA, 2% ACN solution. The peptides were run on a C18-A1 ASY column (Thermo Fisher Scientific, Madrid, Spain) and then eluted on a C18 Biosphere column (10 cm in length, 75 µm in diameter, and 3 µm in particle size). After that, they were separated in a 180 min gradient of 0-35% buffer B in buffer A (buffer A: 0.1% FA / 2% ACN; buffer B: 0.1% FA in ACN) at a flow of 300 nL/min on a nanoEasy HPLC with nanoelectrospray (Thermo Fisher Scientific). Mass spectra corresponding to the full scan spectrum (m/z 400-1200) were obtained with a resolution of 60,000 and the 15 most intense ions were selected for fragmentation by collision-induced dissociation (CID) in the ionic trap, with collision energy normalised to 35%. A dynamic exclusion window of 30 s was applied. The tolerance for the parent ion was set at 10 ppm and 0.5 Da for the product ions. Mass spectrometry data acquired on the LTQ-Orbitrap-Velos were analysed with Maxquant 1.6.15.0 using LFQ quantification [47]. The raw data of the mass spectra (* .raw) corresponding to the cell line experiment were interrogated against the Uniprot human reference proteome UP000005640 (79,038 proteins) database. Statistical and quality control analysis were performed using Perseus 1.6.14 [48].

**Microarray analysis of differential gene expression**

For global gene expression analysis, total RNA was isolated from KM12SM and SW620 cells using NucleoSpin RNA kit (Macherey-Nagel. Düren. Germany). Quality assessment of the RNA was assessed with an Agilent 2100 bio-analyser. Samples were processed with “GeneChip® WT PLUS Reagent Kit” (Thermo Fisher Scientific), hybridised with “Clariom™ S Array, human” (Thermo Fisher Scientific) and scanned with a “GeneChip® Scanner 3000 7G” (Thermo Fisher Scientific. Madrid. Spain). Raw data were processed with RMA algorithm included in Transcriptome Analysis Console (Thermo Fisher Scientific) for normalisation and gene level analysis. For each experimental condition, three independent RNA replicates were processed and analysed. Fold-changes between experimental conditions were calculated as a ratio between the mean of the gene expression signals. Statistical analysis was performed with e-bayes limma included in Transcriptome Analysis Console. Gene expression results were compared with the KM12SM and SW620 expression values from GSE59857 dataset, which contains the gene expression analysis for 155 CRC cell lines [10].

**GO categories enriched in high and low risk groups**

Gene differential expression analysis of high *vs* low risk patients was performed in GSE39582 and GSE17538 datasets. Only significantly altered genes (p-value<0.05) in both datasets were considered for further analysis. Most significant top 200 up and down-expressed genes in GSE39582 cohort were selected. Cellular Component (CC) and Biological Process (BP) GO categories were analysed using g:Profiler. Altered genes corresponding to cell migration, cell adhesion, extracellular matrix organisation and cell cycle GO categories were represented in a heatmap, where genes belonging to more than one category were excluded.

**Real-time quantitative PCR**

Total RNA was isolated using TRI-Reagent (Sigma-Aldrich, Madrid, Spain). 1 μg of RNA was retrotranscribed with M-MLV Reverse Transcriptase (Promega, Madrid, Spain) and the mRNA levels were determined by quantitative real-time PCR analysis using LightCycler® 96 (Roche, Barcelona, Spain) using the following primers:

*CD109*, Fw GAACACTGCCCTTCACAGGT, Rev CTGGGTACGTCCGGTTACAC; *NPC2*, Fw TCCTGGCAGCTACATTCCTG, Rev TGGTGAAGGTGACATTGACG; *LTBP1*, Fw CCGAGCATCTGTAAAGTGAC, Rev GACATTTGTCCCTTGAACTG;

*IGFBP3*, Fw GCACAGATACCCAGAACTTC, Rev AGCACATTGAGGAACTTCAG; *PSAP*, FwGCTGGTTTATTTGGATC, Rev AAGCACACGAAGGAAGGATC;

*BMP1*, Fw AAGACAGCACAGGCAACTTC Rev CATCTCGGACCTCCACATAG. PCR conditions were 5 min at 95°C, 35 three-step cycles (30 s at 94°C; 30 s at 60°C and 30 s at 72°C) and 5 min at 72°C. *GAPDH* was amplified as loading control. Each sample was analysed in triplicate, and the relative gene expression quantification was calculated according to the comparative threshold cycle method (2−∆∆Ct).

**Western blot**

Western blot was carried out as previously described [49]. Antibodies were used at the following dilutions: PSAP 1:2000 (E-AB-60459, Elabscience, Houston, TX, USA), NPC2 1:2000 (19888-1-AP, Proteintech, Manchester, UK), CD109 1:1000 (sc-271085, Santa Cruz Biotechnology, Santa Cruz, CA, USA), BMP1 1:2000 (E-AB-5602, Elabscience), IGFBP3 1:1000 (sc-374365, Santa Cruz Biotechnology) and LTBP1 1.1000 (SC-271140, Santa Cruz Biotechnology).

**Immunohistochemistry**

Human liver metastatic tissues were obtained from the Surgical Pathology Department of the Hospital Clínico San Carlos (Madrid). For mouse liver metastasis, KM12SM human colon carcinoma cells were inoculated into the spleen in Swiss nude mice following standard procedures [50]. Two months later, mice were sacrificed and metastatic livers were recovered. To preserve tissue morphology, the liver was fixed 24h with 15mL of 4% PFA, then the tissue was embedded in paraffin at 58ºC and sections were mounted onto gelatin-coated histological slides. Slides were deparaffined for antigen retrieval using citrate sodium buffer (pH 6.0) or Tris/EDTA buffer (pH 9.0) for 25 min and subsequent incubation with the primary antibody at 1:200 dilution (CD109 (sc-271085, Santa Cruz Biotechnology) and NPC2 (19888-1-AP, Proteintech)), 1:100 dilution (BMP1 (E-AB-5602, Elabscience) and PSAP (E-AB-60459, Elabscience) and 1:50 dilution (LTBP1 (SC-271140, Santa Cruz Biotechnology)). No suitable antibody was found for IGFBP3 staining. Then, a peroxidase labelled polymer conjugated to goat anti-mouse and goat anti-rabbit (EnVision Dual Link system-HRP (Dako, Glostrup, Denmark)) was added and the reaction was developed using diaminobenzidine as chromogen and hematoxylin for counterstaining. Images were acquired with a Leica DM2000 LED microscope (Leica Microsystems, Wetzlar, Germany) using a ×20 objective and processed with the LAS-V4.8 Leica software. Staining was assessed by two independent pathologists.
