# Supplementary material for: A prognostic six‐gene expression risk‐score derived from proteomic profiling of the metastatic colorectal cancer secretome
Source: J Pathol Clin Res. 2022 Sep 22;8(6):495–508. doi: 10.1002/cjp2.294 (PMC9535096; doi:10.1002/cjp2.294)
Supplement: Supplementary file 2 — Figure S1. Quality control and gene ontology analysis of the proteomic assay Figure S2. Quality control of the gene expression analysis Figure S3. Validation of the six‐gene risk‐score in GSE17538 Figure S4. Association of risk score‐based classification with current colorectal cancer classifications Figure S5. Analysis of differentially expressed genes between high‐ and low‐risk patients in GSE39582 [file CJP2-8-495-s005.pdf]

# A prognostic six-gene expression risk-score derived from proteomic profiling of the metastatic colorectal cancer secretome

J Robles *et al*. *J Pathol Clin Res*
DOI: https://doi.org/10.1002/cjp2.294

Supplementary Figures S1 – S5

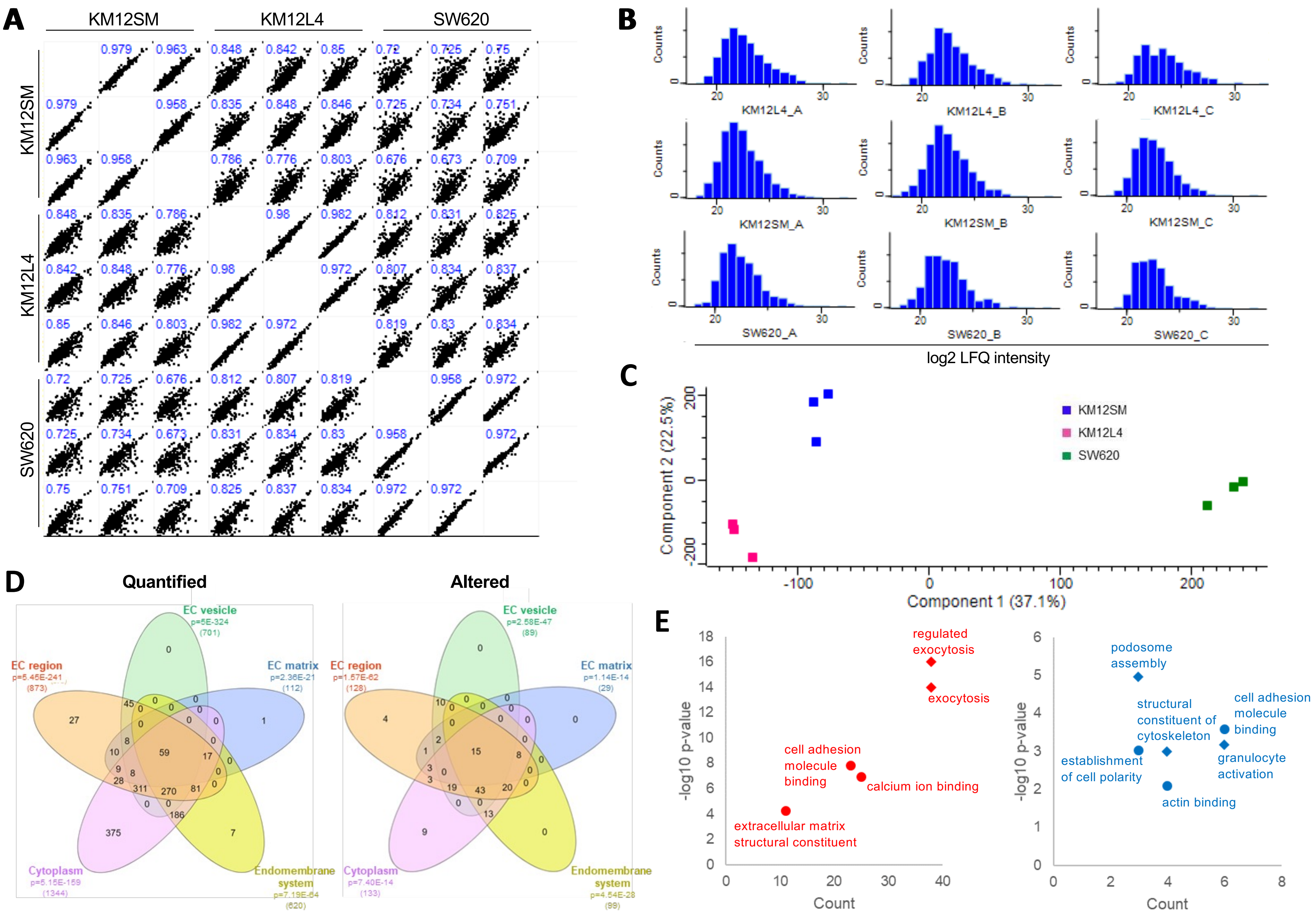


**Figure S1. Quality control and gene ontology analysis of the proteomic assay.** (A) Multi-scatter plot of LFQ intensity values of all the quantified proteins in each triplicate of each analysed sample. Pearson's correlation coefficient is indicated. (B) Histograms of the LFQ intensity ($log_2$) of the quantified proteins in the triplicates. (C) Principal Component Analysis (PCA) of the KM12SM, KM12L4 and SW620 triplicates. (D) Venn diagram representation of significantly altered Cellular Component categories corresponding to total quantified and altered proteins. (E) Molecular Function (circle) and Biological Process (diamond) most significant GO categories corresponding to overexpressed (red) and repressed (blue) proteins in KM12SM and KM12L4 compared to SW620 cells.

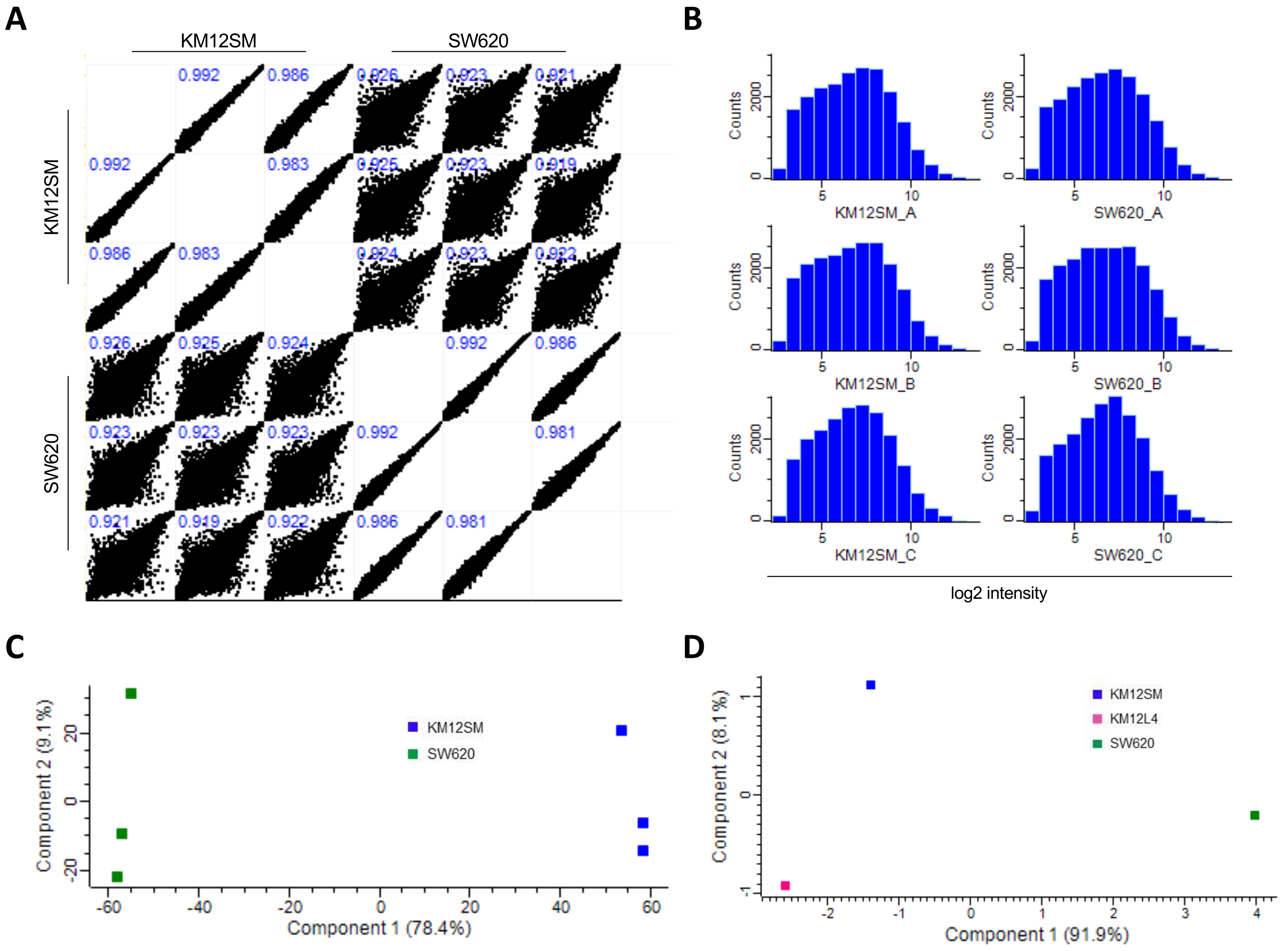


**Figure S2. Quality control of the gene expression analysis**. (A) Multi-scatter plot of intensity value of all the quantified genes in each triplicate of each analysed sample (SW620 and KM12SM). Pearson's correlation coefficient is indicated. (B) Histograms of the intensity ($log_2$) of the quantified genes in the triplicates. (C) Principal Components Analysis (PCA) of KM12SM and SW620 samples using the transcriptomic assay and (D) KM12SM, KM12L4 and SW620 using the GSE59857 database.

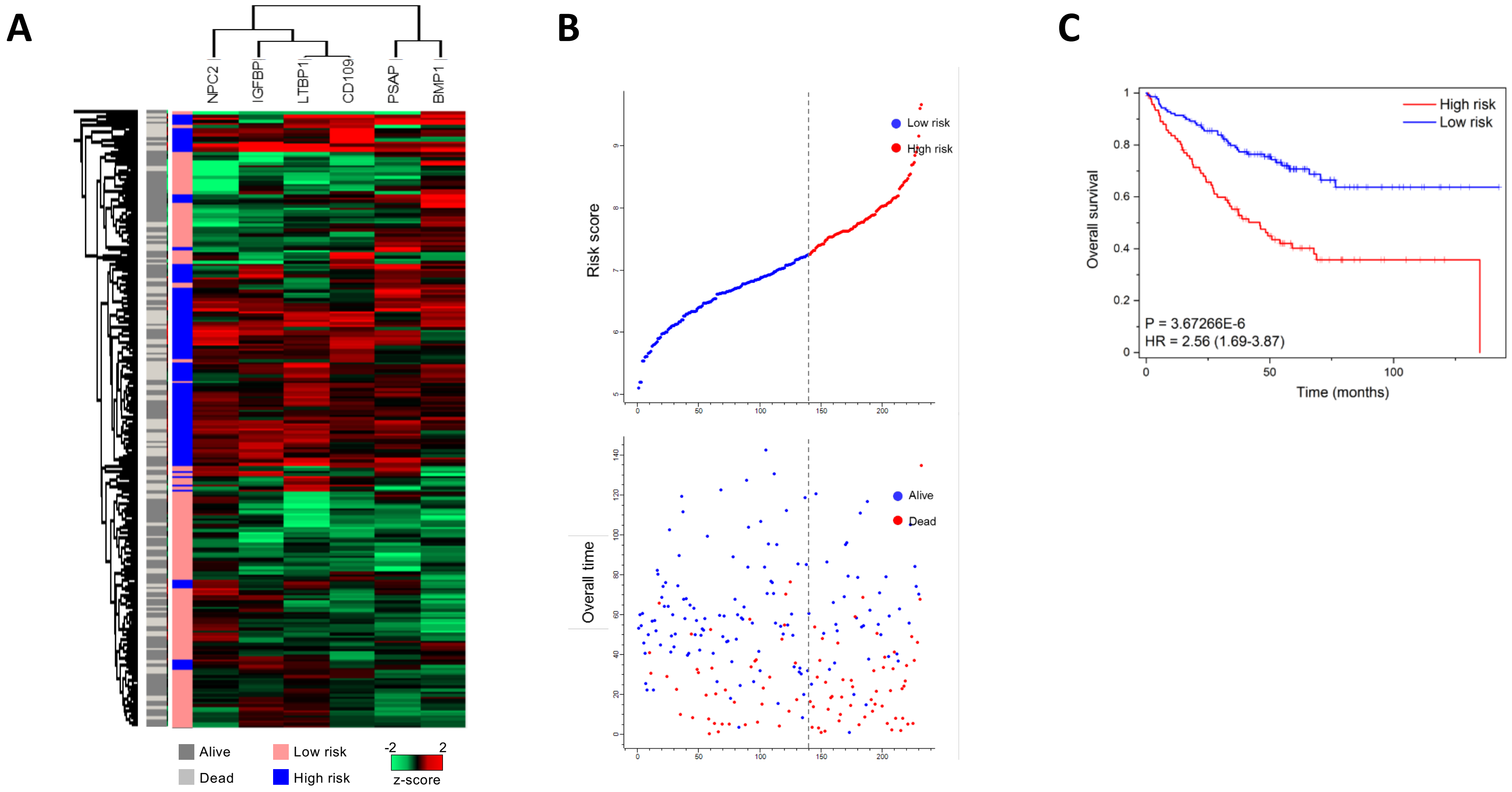


**Figure S3. Design and evaluation of the 6 genes signature in GSE17538.** (A) Hierarchical clustering of mRNA expression in GSE17538. Overall survival event and low-high risk distribution are shown. (B) Risk score distribution and corresponding survival status in GSE17538. (C) Kaplan-Meier analysis of high and low risk patients.

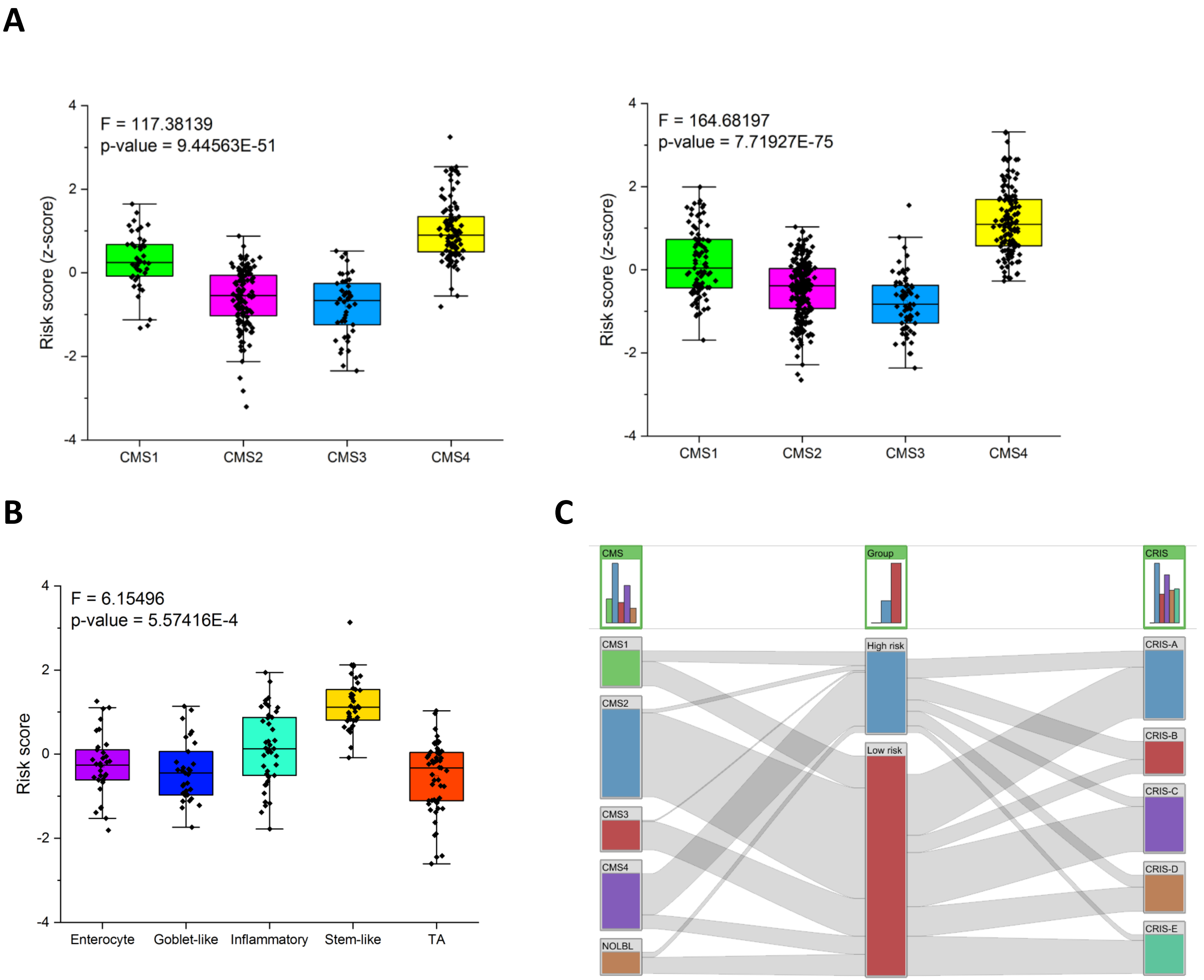


**Figure S4. Association of risk score-based classification with current colorectal cancer classifications.** (A) Risk score (z-score) in CMS subgroups according to GSE14333 and GSE39582 datasets. (B) Risk score (z-score) in Sandanandam subgroups according to GSE14333 dataset. (C) Caleydo view of the correspondence between CMS, SEC6 risk score and CRIS subgrouping according to GSE14333, GSE39582 and TCGA COADREAD data.

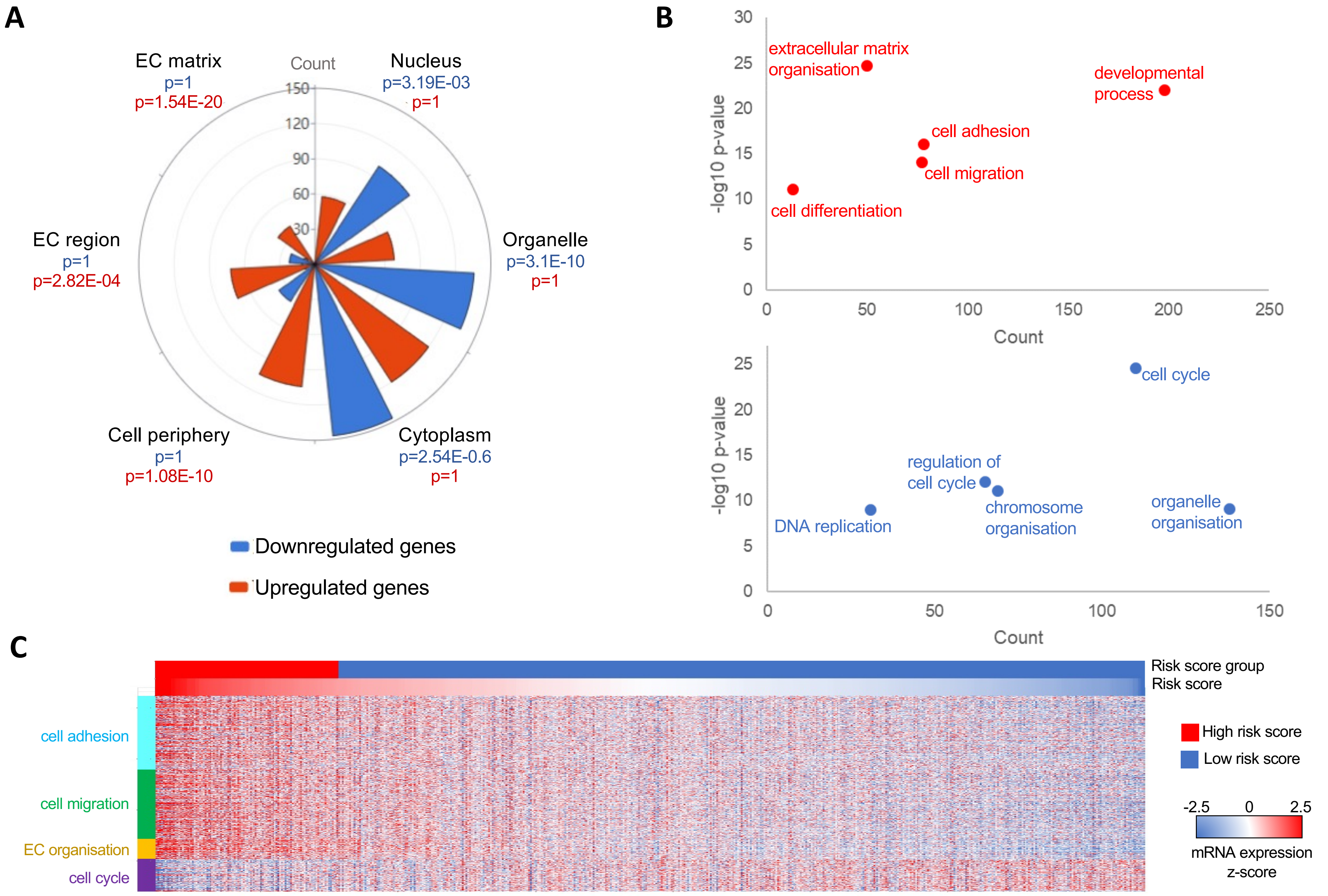


**Figure S5. Analysis of differentially expressed genes between high and low risk patients in GSE39582.** (A) Rose plot of altered Cellular Component and (B) scatter plot of altered Biological Process GO categories in the 200 most significantly upregulated (red) and downregulated (blue) genes when comparing high versus low risk patients. (C) Expression pattern of significantly altered genes from cell adhesion, cell migration, extracellular matrix organisation and cell cycle GO categories. Patients from GSE39582 were ordered according to the risk score.
