# Supplementary material for: A prognostic six‐gene expression risk‐score derived from proteomic profiling of the metastatic colorectal cancer secretome
Source: J Pathol Clin Res. 2022 Sep 22;8(6):495–508. doi: 10.1002/cjp2.294 (PMC9535096; doi:10.1002/cjp2.294)
Supplement: Supplementary file 3 — Table S1. List of deregulated proteins identified and quantified in the secretome of KM12SM, KM12L4, and SW620 [file CJP2-8-495-s006.pdf]

A prognostic six-gene expression risk-score derived from proteomic profiling of the metastatic colorectal cancer secretome

J Robles *et al.* *J Pathol Clin Res* DOI: https://doi.org/10.1002/cjp2.294

Table S1. List of deregulated proteins identified and quantified in the secretome of KM12SM, KM12L4 and SW620

Protein IDs	Gene names	LFQ intensity			Fold change			p-value		
		SW620	KM12L4	KM12SM	L4/620	SM/620	L4/SM	L4/620	SM/620	L4/SM
O60613	SEP15	0	9227900	0	NaN	NaN	NaN	0.37390098	1	0.37390098
P04217	A1BG	0	0	130400	NaN	NaN	0	0.37390098	0.37390098	1
P01023	A2M	1313300	0	0	0	0	NaN	0.37390098	0.37390098	1
A8K2U0	A2ML1	19185000	605950016	407140000	31.5845718	21.2217884	1.48830879	0.00081525	0.00055706	0.06829024
P49588	AARS	93488000	37229000	55207000	0.39822224	0.59052497	0.67435288	0.00022097	0.00083064	0.00817151
Q9NRN7	AASDHPPT	0	0	6600500	NaN	NaN	0	1	0.37390098	0.37390098
P61221	ABCE1	40952000	20072000	28953000	0.49013481	0.70699841	0.6932615	0.12491227	0.98181456	0.11728708
Q9BUJ0	ABHD14A	0	0	11367000	NaN	NaN	0	1	0.54796535	0.5748325
Q96IU4	ABHD14B	54528000	10999000	12853000	0.20171288	0.23571377	0.85575354	0.0050985	0.0050985	1
P24752	ACAT1	0	0	2052400	NaN	NaN	0	0.37390098	0.37390098	1
Q9BWD1	ACAT2	39022000	24950000	36389000	0.6393829	0.93252522	0.68564677	0.11631453	0.52769148	0.11735933
Q9UKV3	ACIN1	0	9427200	6556200	NaN	NaN	1.43790615	1	0.37390098	0.37390098
P53396	ACLY	101560000	68218000	155320000	0.67170143	1.52934229	0.43920937	0.09561997	0.00519718	0.00896867
P21399	ACO1	0	0	23875000	NaN	NaN	0	1	0.26493192	0.72209376
P24666	ACP1	53379000	22969000	62812000	0.4303003	1.1767174	0.36567852	0.99269515	0.14411557	0.04391851
P68133	ACTA1	32259000	0	0	0	0	NaN	1	0.37390098	0.37390098
Q562R1	ACTBL2	3131099904	0	0	0	0	NaN	0.37390098	0.37390098	1
O94805	ACTL6B	0	0	4738500	NaN	NaN	0	1	0.37390098	0.37390098
P12814	ACTN1	509369984	162700000	160390000	0.3194142	0.31487918	1.01440239	0.00159445	0.0008346	0.17718181
O43707	ACTN4	2510599936	1441400064	1919100032	0.57412577	0.76439899	0.75108123	0.00276794	0.11736844	0.00521346
P61160	ACTR2	21597000	9840300	12412000	0.45563272	0.57470948	0.79280537	0.12270122	0.41867098	0.37390098
P61158	ACTR3	134380000	50843000	77241000	0.37835243	0.57479537	0.65823847	0.02504174	0.69780147	0.0216393
P07311	ACYP1	11690000	11225000	11138000	0.96022242	0.95278013	1.00781107	0.37390098	1	0.37390098
O14672	ADAM10	53978000	430740000	194980000	7.97991753	3.61221242	2.2091496	4.61E-05	5.15E-05	0.02861929
Q13444	ADAM15	0	0	5126300	NaN	NaN	0	1	0.37390098	0.37390098
P78536	ADAM17	0	0	6702000	NaN	NaN	0	1	0.37390098	0.37390098
Q13443	ADAM9	0	290390016	126770000	NaN	NaN	2.29068398	2.42E-05	0.00041521	0.00144785
Q86TH1	ADAMTSL2	11687000	0	0	0	0	NaN	0.11611766	0.11611766	1
Q9BV57	ADI1	0	0	6312800	NaN	NaN	0	1	0.37390098	0.37390098
P55263	ADK	67735000	19425000	26996000	0.28677937	0.39855319	0.71955103	0.12392485	0.12392485	1

Q9NX46	ADPRHL2	38010000	22865000	25185000	0.60155225	0.66258878	0.90788168	0.12684681	0.12684681	1
P30566	ADSL	80259000	27127000	43702000	0.33799323	0.54451215	0.62072676	0.00423666	0.0196455	0.03234163
P30520	ADSS	17953000	10258000	7104700	0.57138085	0.39573887	1.44383299	0.37390098	0.37390098	1
P43652	AFM	3199900	0	2201900	0	0.68811524	0	0.37390098	0.37390098	1
P20933	AGA	0	0	16449000	NaN	NaN	0	1	0.58150309	0.94568974
P35573	AGL	77900000	20564000	31312000	0.26397946	0.40195122	0.65674502	0.11616844	0.11616844	1
O95994	AGR2	0	4228300032	411110016	NaN	NaN	10.2850819	0.02034	0.3739	0.02103
Q8TD06	AGR3	0	965139968	255420000	NaN	NaN	3.77863908	0.00133702	0.00479857	0.00627414
O00468	AGRN	86248000	510600000	162470000	5.92013741	1.88375378	3.14273405	0.00108379	0.00052273	0.00332596
P23526	AHCY	709350016	537470016	1049200000	0.75769365	1.47910058	0.51226652	0.03980173	0.0014305	0.00048833
O43865	AHCYL1	0	2591800	0	NaN	NaN	NaN	0.76211649	1	0.36506638
Q09666	AHNAK	7171600	17203000	33440000	2.39876747	4.66283655	0.51444376	0.13822877	0.00021282	0.02925143
O95433	AHSA1	51312000	27287000	60256000	0.53178596	1.17430615	0.45285118	0.34302923	0.18148194	0.01096651
P02765	AHSG	23633000	0	16948000	0	0.71713281	0	0.19073597	0.46096233	0.12325215
O95831	AIFM1	253350000	79681000	66220000	0.31450957	0.26137754	1.20327699	0.01257033	0.01031453	0.94910187
Q12904	AIMP1	13189000	6204400	10705000	0.47042233	0.81166124	0.57957965	0.37390098	0.37390098	1
Q13155	AIMP2	0	0	2757200	NaN	NaN	0	1	0.37390098	0.37390098
O00170	AIP	8896300	0	4337300	0	0.48753977	0	0.37390098	0.37390098	1
P00568	AK1	30577000	13559000	24569000	0.44343787	0.80351245	0.55187434	0.12111014	0.72225171	8.80E-05
P54819	AK2	76094000	24366000	22573000	0.32020921	0.29664627	1.07943118	0.00155245	0.01127539	0.37390098
Q02952	AKAP12	49224000	0	16926000	0	0.34385666	0	0.37390098	0.37390098	1
P14550	AKR1A1	147580000	161730000	231880000	1.09588015	1.57121563	0.69747281	0.09210984	0.02217175	0.01535063
P42330	AKR1C3	102030000	19101000	34520000	0.18720965	0.33833185	0.55333138	0.00019028	0.00164091	0.37390098
Q13740	ALCAM	0	137590000	245930000	NaN	NaN	0.55946815	0.3739	0	0.00714
P00352	ALDH1A1	0	248170000	305400000	NaN	NaN	0.81260639	0.0069	4.00E-05	0.55544001
P05091	ALDH2	0	0	0	NaN	NaN	NaN	1	1	1
P30838	ALDH3A1	0	26616000	42503000	NaN	NaN	0.62621462	0.37390098	8.27E-05	0.00843435
P49419	ALDH7A1	0	0	3803300	NaN	NaN	0	1	0.37390098	0.37390098
P49189	ALDH9A1	18103000	18219000	32153000	1.00640774	1.77611446	0.56663454	0.3658036	0.03050308	0.0419788
P04075	ALDOA	2434400000	2535800064	2500000000	1.04165304	1.02694714	1.01432002	0.24797064	0.01629752	0.35634774
P09972	ALDOC	5377400	5057900	1789000	0.94058466	0.33268866	2.82722187	0.96777076	0.37390098	0.37390098
Q86V81	ALYREF	29493000	0	0	0	0	NaN	0.37390098	0.37390098	1
Q01433	AMPD2	2734900	0	0	0	0	NaN	0.37390098	0.37390098	1

P03950	ANG	0	130130000	69407000	NaN	NaN	1.87488294	0	8.00E-05	0.00122
Q9BYT9	ANO3	9428100	0	0	0	0	NaN	0.37390098	1	0.37390098
P39687	ANP32A	50139000	228800000	279280000	4.56331396	5.57011509	0.81924951	0.00554622	0.00112511	0.0344012
Q92688	ANP32B	14977000	28450000	43854000	1.89957941	2.92808986	0.64874357	1	1.18E-05	1.18E-05
Q9BTT0	ANP32E	0	0	7401900	NaN	NaN	0	1	0.37390098	0.37390098
P04083	ANXA1	0	5367200	3689800	NaN	NaN	1.45460463	1	0.37390098	0.37390098
P50995	ANXA11	5225700	10719000	5461000	2.0512085	1.04502749	1.96282732	0.02167309	0.37390098	0.00039683
P07355	ANXA2	78318000	329289984	205760000	4.20452499	2.62723756	1.60035956	0.29466699	0.00045014	0.8054589
P08758	ANXA5	12459000	43641000	12152000	3.50276899	0.9753592	3.59126067	0.11617087	1	0.11617087
Q10567	AP1B1	4188100	3585100	4291000	0.85602063	1.02456963	0.83549291	0.03873081	0.73774755	0.03976638
O43747	AP1G1	33468000	17377000	18467000	0.51921237	0.55178082	0.94097579	1	0.37390098	0.37390098
Q9Y6Q5	AP1M2	0	0	7698800	NaN	NaN	0	0.37390098	1	0.37390098
P63010	AP2B1	38803000	20095000	31534000	0.51787233	0.8126691	0.63724869	1	0.37390098	0.37390098
P13798	APEH	10630000	7967300	19638000	0.74951082	1.84741294	0.40570831	0.31475449	0.62066633	0.6309855
P27695	APEX1	163260000	96415000	133030000	0.59056109	0.81483525	0.72476131	0.00331927	0.18413381	0.00874342
Q9BZZ5	API5	41655000	23769000	32939000	0.57061577	0.79075742	0.72160661	0.02243861	0.05718835	0.07166502
Q96GX9	APIP	35697000	0	3536800	0	0.09907836	0	0.37390098	0.37390098	1
Q06481	APLP2	10172000	538480000	516040000	52.9374771	50.7314186	1.04348505	0.01916514	2.62E-05	0.39511356
P06727	APOA4	6024000	3449900	4530500	0.57269257	0.75207502	0.76148325	0.2637763	0.39465225	0.37390098
P04114	APOB	50809000	9998100	23129000	0.19677813	0.45521462	0.4322755	0.31717405	0.40758237	0.19448258
P02649	APOE	477940	0	0	0	0	NaN	0.37390098	0.37390098	1
P05067	APP	1107200000	5028800000	4286299904	4.54190731	3.87129688	1.17322636	0.00214795	0.00303456	0.34020433
P07741	APRT	0	0	7526000	NaN	NaN	0	1	0.37390098	0.37390098
P48444	ARCN1	15989000	11779000	13474000	0.73669398	0.84270436	0.87420219	0.99221778	0.42753541	0.42045635
P15514	AREG	14150000	119750000	147080000	8.4628973	10.3943462	0.81418276	0.11636214	0.11687395	0.7193433
P84077	ARF1	50146000	28633000	30910000	0.57099271	0.61640012	0.9263345	0.07721483	0.14215089	0.34701684
P18085	ARF4	0	0	5983300	NaN	NaN	0	1	0.37390098	0.37390098
P53367	ARFIP1	0	0	1762200	NaN	NaN	0	1	0.37390098	0.37390098
P53365	ARFIP2	0	0	1235900	NaN	NaN	0	1	0.37390098	0.37390098
P05089	ARG1	25170000	22665000	0	0.90047675	0	NaN	0.12722827	0.12722827	1
P78540	ARG2	874100	0	0	0	0	NaN	0.37390098	1	0.37390098
Q07960	ARHGAP1	34941000	40126000	41180000	1.14839303	1.17855811	0.97440505	0.37390098	0.9672997	0.11616877
Q8N392	ARHGAP18	9984300	0	0	0	0	NaN	0.37390098	0.37390098	1

P52565	ARHGDIA	234320000	49297000	71879000	0.21038324	0.30675572	0.68583316	0.00242915	0.00516305	0.12774892
Q9NZN5	ARHGEF12	0	53315000	0	NaN	NaN	NaN	0.65175796	1	0.37227702
O15143	ARPC1B	55935000	38506000	84210000	0.68840617	1.50549746	0.45726162	0.37390098	0.37390098	1
O15144	ARPC2	160500000	69901000	128540000	0.43552026	0.80087227	0.54380739	2.10E-05	0.00018513	0.00348819
P59998	ARPC4	131570000	55886000	93963000	0.42476249	0.71416736	0.59476602	0.2534954	0.78191411	0.30798602
O15511	ARPC5	0	0	43445000	NaN	NaN	0	1	0.37390098	0.37390098
P56211	ARPP19	0	0	3369000	NaN	NaN	0	0.37390098	0.37390098	1
Q6UWY0	ARSK	0	0	1108600	NaN	NaN	0	0.37390098	1	0.37390098
Q13510	ASAH1	37560000	997880000	864860032	26.567625	23.0260925	1.15380526	6.64E-05	0.00011971	0.03763254
P04424	ASL	9564800	9514900	19645000	0.99478292	2.05388498	0.48434207	0.7934925	0.22426866	0.01561604
P08243	ASNS	7391300	0	10128000	0	1.37025964	0	1	0.01095315	0.01095315
Q676U5	ATG16L1	0	0	3549400	NaN	NaN	0	1	0.37390098	0.37390098
P31939	ATIC	207010000	115390000	164610000	0.55741268	0.79517895	0.7009902	0.00019949	0.24197595	0.87576681
P05023	ATP1A1	37956000	159170000	35886000	4.1935401	0.94546318	4.43543434	0.00050566	0.50844252	1.99E-05
P05026	ATP1B1	45755000	311590016	35792000	6.80996656	0.78225332	8.7055769	0.00014129	0.11698487	0.00049896
Q15904	ATP6AP1	0	92501000	61389000	NaN	NaN	1.50680089	0.11731472	0.11781423	0.91144866
O75787	ATP6AP2	10887000	73363000	54064000	6.73858738	4.96592283	1.35696578	1	0.11860574	0.11860574
P38606	ATP6V1A	34041000	12615000	36221000	0.37058252	1.06404042	0.34827861	0.37390098	0.41030043	0.11616866
P21281	ATP6V1B2	13295000	0	14765000	0	1.11056793	0	0.37390098	0.37390098	1
P21283	ATP6V1C1	22516000	15481000	32917000	0.68755549	1.46193814	0.4703041	1	0.00018414	0.00018414
Q96A05	ATP6V1E2	0	0	6216700	NaN	NaN	0	1	0.37390098	0.37390098
O75348	ATP6V1G1	0	0	2260200	NaN	NaN	0	1	0.37390098	0.37390098
P98198	ATP8B2	0	0	0	NaN	NaN	NaN	1	1	1
Q9UBB4	ATXN10	4637500	0	0	0	0	NaN	0.37390098	0.37390098	1
Q8WWM7	ATXN2L	13606000	10735000	18898000	0.78899014	1.38894606	0.56804955	0.37390098	0.37390098	1
P25311	AZGP1	254910000	49448000	42200000	0.19398218	0.16554862	1.17175353	0.0566476	0.07343865	0.36247852
P61769	B2M	245750000	5306299904	3310599936	21.592268	13.4714136	1.60282123	0.01187395	0.14491903	0.27077347
O94766	B3GAT3	9845200	12260000	20304000	1.24527693	2.06232476	0.60382193	1	0.37390098	0.37390098
O43505	B3GNT1	32636000	31190000	28957000	0.95569313	0.8872717	1.07711434	0.64446795	0.50826061	0.88262123
Q9Y2A9	B3GNT3	0	32915000	43263000	NaN	NaN	0.76081181	0.37390098	0.37390098	0.72277182
Q8NFL0	B3GNT7	0	6848800	0	NaN	NaN	NaN	0.37390098	1	0.37390098
P15291	B4GALT1	0	0	2000000	NaN	NaN	0	1	0.37390098	0.37390098
O43286	B4GALT5	0	24367000	29892000	NaN	NaN	0.81516796	0.37390098	0.1717602	0.64393336

Q99933	BAG1	0	0	5668800	NaN	NaN	0	1	0.11611865	0.11611865
O95816	BAG2	0	0	22473000	NaN	NaN	0	0.37390098	0.34629354	0.12298628
O95817	BAG3	4512800	0	13066000	0	2.89531994	0	1	0.37390098	0.37390098
Q9UHR4	BAIAP2L1	0	0	2482600	NaN	NaN	0	1	0.84773004	0.45855418
Q07812	BAX	1318400	0	0	0	0	NaN	0.37390098	0.37390098	1
Q13867	BLMH	27745000	23183000	29076000	0.83557397	1.04797256	0.79732424	0.1464034	0.11633515	0.83083224
P53004	BLVRA	0	0	4379600	NaN	NaN	0	0.37390098	0.37390098	0.91011131
P30043	BLVRB	0	0	5374100	NaN	NaN	0	1	0.37390098	0.37390098
P13497	BMP1	8620900	64805000	17920000	7.51719666	2.07866931	3.61635041	0.37390098	1	0.37390098
P18075	BMP7	13898000	33299000	17565000	2.39595628	1.26385093	1.89575863	0.22954054	0.07724521	0.6210295
Q9H3K6	BOLA2	34643000	31927000	57385000	0.92160034	1.65646744	0.55636489	1	0.11850858	0.11850858
O95861	BPNT1	89907000	244930000	229070000	2.72425961	2.5478549	1.06923652	0.02248631	0.02934772	0.84570283
Q5VW32	BROX	0	0	17242000	NaN	NaN	0	1	0.37390098	0.37390098
P35613	BSG	83987000	115760000	47735000	1.37830853	0.56836176	2.42505503	0.61419392	0.13547382	0.1215523
P43251	BTD	0	54474000	41874000	NaN	NaN	1.30090272	0.00048504	1.93E-05	0.86967671
O43684	BUB3	38825000	24157000	40653000	0.62220222	1.04708302	0.59422427	0.71640742	0.82473081	0.60261226
Q7L1Q6	BZW1	24169000	0	38630000	0	1.59832847	0	0.11803669	0.98414588	0.37390098
Q9Y6E2	BZW2	43923000	40762000	78100000	0.92803317	1.7781117	0.52192062	0.95678413	0.28097874	0.30012664
Q53FT3	C11orf73	0	0	9886000	NaN	NaN	0	0.37390098	0.37390098	1
Q9HB07	C12orf10	18662000	0	0	0	0	NaN	1	0.37390098	0.37390098
Q9Y224	C14orf166	49848000	12166000	32800000	0.24406195	0.65800035	0.37091464	2.39E-06	0.22782864	0.1357507
Q969H8	C19orf10	37324000	966739968	149300000	25.9012966	4.00010729	6.47515059	0.01245458	0.37390097	0.02512344
Q96EU7	C1GALT1C1	0	69671000	71937000	NaN	NaN	0.9685002	0.11644182	0.00096443	0.46221837
Q07021	C1QBP	0	0	1773500	NaN	NaN	0	0.37390098	1	0.37390098
P00736	C1R	1541900	0	0	0	0	NaN	0.37390098	0.37390098	1
P09871	C1S	0	0	514400	NaN	NaN	0	1	0.37390098	0.37390098
Q9Y3I0	C22orf28	65607000	19949000	39665000	0.30406815	0.60458487	0.50293708	0.1229151	0.68538529	0.13194644
P01024	C3	5457200	1169600	1439100	0.21432236	0.26370665	0.81273019	0.37390098	0.37390098	1
Q8NDZ4	C3orf58	0	0	1612200	NaN	NaN	0	1	0.37390098	0.37390098
P0C0L5	C4B	16130000	27729000	12534000	1.71909487	0.7770614	2.21230245	0.29063985	0.52539653	0.41448644
P01031	C5	1010200	0	0	0	0	NaN	0.37390098	0.37390098	1
Q8WVR3	C7orf43	0	8073400	0	NaN	NaN	NaN	0.08055233	1	0.35694909
P07358	C8B	0	0	2539400	NaN	NaN	0	1	0.37390098	0.37390098

P07360	C8G	0	0	872510	NaN	NaN	0	1	0.37390098	0.37390098
P00918	CA2	0	25248000	27551000	NaN	NaN	0.91640955	0.11633	1.00E-05	0.1582
Q9Y376	CAB39	0	0	1578300	NaN	NaN	0	1	0.37390098	0.37390098
Q9H9S4	CAB39L	15926000	0	0	0	0	NaN	0.00025404	0.00025404	1
Q5VU97	CACHD1	30560000	9950400	8306800	0.32560208	0.27181938	1.19786203	0.37390098	0.37390098	1
Q9HB71	CACYBP	217960000	137760000	233130000	0.63204259	1.06959999	0.59091496	0.0024833	0.1069835	0.00641896
P27708	CAD	42383000	0	20265000	0	0.47813982	0	0.37390098	0.37390098	1
Q05682	CALD1	4024600	0	0	0	0	NaN	0.37390098	0.37390098	1
P62158	CALM1	135120000	129320000	90935000	0.95707518	0.67299438	1.42211473	0.04705747	0.04685712	0.12346306
P27482	CALML3	0	34063000	0	NaN	NaN	NaN	0.37390098	1	0.37390098
Q9NZT1	CALML5	44931000	20340000	0	0.45269412	0	NaN	0.13291162	1	0.13291162
P27797	CALR	114710000	7042100224	751379968	61.3904648	6.55025673	9.3722229	2.35E-05	0.00366006	3.10E-05
O43852	CALU	98864000	4381599744	2091299968	44.3194656	21.1533012	2.09515595	0.10604148	0.03586979	0.82575566
Q86VP6	CAND1	96295000	107840000	157960000	1.119892	1.64037597	0.68270451	0.6371308	0.00812591	0.02533928
Q8WVQ1	CANT1	29209000	68317000	155740000	2.33890247	5.33191824	0.43866059	0.37390097	0.11618599	0.42171994
P27824	CANX	0	48461000	19904000	NaN	NaN	2.43473673	0.11659439	1	0.11659439
Q01518	CAP1	524209984	380320000	364649984	0.72551078	0.69561815	1.0429728	0.22349028	0.54576111	0.24826404
P40121	CAPG	12375000	18246000	44593000	1.47442424	3.60347486	0.40916735	1	0.11673227	0.11673227
P07384	CAPN1	9899200	10447000	7452700	1.05533779	0.75285882	1.40177381	1	0.37390098	0.37390098
P17655	CAPN2	0	6397900	4644600	NaN	NaN	1.37749219	0.37390098	0.37390098	1
P04632	CAPNS1	0	0	5374800	NaN	NaN	0	0.37390098	0.37390098	1
Q14444	CAPRIN1	90538000	36960000	52193000	0.40822637	0.57647616	0.70814091	0.12346592	0.13118163	0.86801118
P52907	CAPZA1	22494000	29504000	30830000	1.31163871	1.37058771	0.95698994	0.38917351	0.37390098	0.11774035
P47755	CAPZA2	0	0	540730	NaN	NaN	0	0.37390098	0.37390098	1
P47756	CAPZB	117920000	90433000	118080000	0.76690131	1.00135684	0.76586211	0.05686536	0.45347044	0.95077664
Q86X55	CARM1	0	0	5313400	NaN	NaN	0	1	0.37390098	0.37390098
P49589	CARS	96457000	27702000	39669000	0.28719532	0.41126096	0.69832867	0.00028885	0.01386883	1.31E-05
P42574	CASP3	0	4214900	0	NaN	NaN	NaN	0.88147652	1	0.12433118
P55210	CASP7	4171600	0	4081700	0	0.97844952	0	0.71456367	0.78206754	0.20860188
P20810	CAST	3096700	9460500	12573000	3.05502629	4.06012869	0.7524457	1	0.37390098	0.37390098
P04040	CAT	60463000	86808000	85846000	1.43572104	1.41981041	1.01120615	0.33823317	0.00517915	0.30695605
Q13951	CBFB	60437000	13058000	16771000	0.2160597	0.27749556	0.77860594	0.11861558	0.11861558	1
P16152	CBR1	13051000	11876000	11859000	0.90996861	0.90866601	1.00143349	1	0.37390098	0.37390098

O75828	CBR3	0	0	15779000	NaN	NaN	0	1	0.08065383	0.83996314
Q13185	CBX3	358070016	242700000	364670016	0.67780042	1.01843214	0.66553319	0.53462207	0.49876407	0.07003515
Q8WUD4	CCDC12	0	0	0	NaN	NaN	NaN	1	1	1
Q96CT7	CCDC124	0	0	9348600	NaN	NaN	0	1	0.37390098	0.37390098
Q5T9S5	CCDC18	95315000	274320000	0	2.87803602	0	NaN	0.37390098	0.37390098	1
Q5TID7	CCDC181	41534000	0	0	0	0	NaN	0.41789296	0.45131919	1
Q86WR0	CCDC25	5019800	5654300	14640000	1.12639952	2.91645074	0.38622269	1	0.37390098	0.37390098
Q96MW1	CCDC43	0	0	8142500	NaN	NaN	0	0.37390098	0.37390098	1
Q4VC31	CCDC58	11227000	0	0	0	0	NaN	0.37390098	0.37390098	1
P78371	CCT2	584609984	204660000	398280000	0.35007954	0.68127471	0.51385957	0.00049587	0.0542074	0.00232465
P49368	CCT3	528649984	165430000	366260000	0.31292918	0.69282138	0.45167369	2.14E-05	0.00930425	7.91E-05
P50991	CCT4	425620000	171340000	335910016	0.40256566	0.78922516	0.51007706	6.69E-05	0.03762946	0.0006913
P48643	CCT5	339740000	139550000	310760000	0.41075528	0.91469949	0.44906038	0.00391237	0.16945995	0.00031034
P40227	CCT6A	289689984	111890000	219070000	0.38624048	0.75622219	0.51075	0.00067268	0.30244625	0.00024203
Q99832	CCT7	291430016	106440000	249510000	0.36523348	0.85615754	0.42659613	0.00100131	0.69737881	0.00025419
P50990	CCT8	737569984	252410000	513000000	0.34221837	0.6955272	0.49202728	0.0042995	0.10837343	0.00018375
Q6YHK3	CD109	22201000	996440000	1020600000	44.8826637	45.9709015	0.97632766	0.00015271	0.00012052	0.25060171
P16070	CD44	13682000	462329984	49523000	33.791111	3.61957312	9.33566189	2.05E-06	0.13054971	0.00447128
P15529	CD46	19274000	17869000	12414000	0.92710388	0.6440801	1.4394232	0.27311271	8.54E-06	0.37390098
P08174	CD55	0	69442000	19580000	NaN	NaN	3.54657817	0.00246957	1	0.00246957
P19256	CD58	0	0	10927000	NaN	NaN	0	1	0.37390098	0.37390098
P13987	CD59	19135000	68367000	38215000	3.57287693	1.99712574	1.78900957	0.37390097	0.37390097	0.83076894
P08962	CD63	0	0	35504000	NaN	NaN	0	1	0.37390098	0.37390098
P21926	CD9	85368000	847900032	558430016	9.93229389	6.5414443	1.51836395	0.00150509	0.16933268	0.06819507
Q16543	CDC37	24088000	23661000	40912000	0.98227334	1.698439	0.57833886	0.07164154	0.29287603	0.01539431
O60508	CDC40	0	0	3092200	NaN	NaN	0	1	0.37390098	0.37390098
P60953	CDC42	0	0	13153000	NaN	NaN	0	1	0.37390098	0.37390098
Q6P1J9	CDC73	5743100	4761100	4393900	0.82901222	0.76507461	1.08357036	0.37390098	0.37390098	1
Q9H5V8	CDCP1	0	4734400	0	NaN	NaN	NaN	0.37390098	1	0.37390098
P12830	CDH1	18126000	944510016	846470016	52.1080208	46.6992188	1.1158222	0.00100209	8.14E-05	0.69347769
Q12864	CDH17	66324000	198250000	270790016	2.98911405	4.08283615	0.73211712	1.57E-05	5.22E-05	0.00398979
P22223	CDH3	0	36400000	23831000	NaN	NaN	1.52742231	0.12529001	0.11617	0.91117001
P06493	CDK1	56159000	19967000	29867000	0.35554409	0.5318293	0.66853046	0.00016075	0.03928741	0.11614255

Q00537	CDK17	0	25957000	0	NaN	NaN	NaN	0.37390098	1	0.37390098
Q00534	CDK6	9226300	0	3768700	0	0.40847361	0	0.37390098	0.37390098	1
Q16878	CDO1	0	37067000	0	NaN	NaN	NaN	1	0.37390098	0.37390098
Q9UKY7	CDV3	79243000	47216000	47784000	0.59583813	0.60300595	0.98811316	0.01127908	0.02656915	0.30740809
P06731	CEACAM5	0	8619500	0	NaN	NaN	NaN	0.37390098	1	0.37390098
P00751	CFB	2572900	0	1468300	0	0.57067901	0	0.37390098	0.49404007	0.37390098
P08603	CFH	6005300	0	2439300	0	0.4061912	0	0.37390098	0.37390098	1
P23528	CFL1	8266299904	3427699968	5568999936	0.41465953	0.67369926	0.61549652	0.01835938	0.92110693	0.03987916
Q99674	CGREF1	24716000	26394000	15989000	1.06789124	0.64690888	1.65075994	0.14377573	0.37390098	0.30645692
Q9Y6H1	CHCHD2	0	0	5749500	NaN	NaN	0	1	0.37390098	0.37390098
P05060	CHGB	19837000	0	0	0	0	NaN	0.37390098	0.37390098	1
Q9BWS9	CHID1	0	3973100	0	NaN	NaN	NaN	1	0.37390098	0.37390098
Q9UQN3	CHMP2B	0	18399000	14994000	NaN	NaN	1.22709084	1	0.00035042	0.00035042
Q9H444	CHMP4B	0	0	5483200	NaN	NaN	0	1	0.37390098	0.37390098
Q9NZZ3	CHMP5	6305900	0	0	0	0	NaN	0.31686318	0.91902488	1
Q9UHD1	CHORDC1	184980000	60125000	135780000	0.32503513	0.7340253	0.44281191	0.00193859	0.08349147	0.01126809
Q8NCH0	CHST14	2209400	6872800	6864900	3.11070871	3.10713315	1.00115073	1	0.1161589	0.1161589
Q6FI81	CIAPIN1	59940000	25181000	30447000	0.42010343	0.50795794	0.82704371	0.37390098	0.37390098	1
Q14011	CIRBP	0	0	1229300	NaN	NaN	0	0.37390098	0.37390098	1
Q14008	CKAP5	5703400	2824400	4182700	0.49521339	0.73336959	0.67525762	0.37390098	0.37390098	1
P12532	CKMT1A	0	0	29429000	NaN	NaN	0	1	0.17015004	0.66238302
O15551	CLDN3	0	18542000	6746500	NaN	NaN	2.74838805	0.12222	1	0.12222
O00299	CLIC1	325400000	178800000	381430016	0.54947758	1.17218816	0.46876228	0.072387	0.00538643	0.00097375
Q9Y696	CLIC4	110870000	112130000	225520000	1.0113647	2.0340941	0.49720645	0.37390098	0.27085525	0.1197013
Q14677	CLINT1	10747000	0	6728400	0	0.62607241	0	1	0.37390098	0.37390098
O75503	CLN5	0	81904000	81715000	NaN	NaN	1.0023129	0.00059905	0.00166633	0.86181194
O94985	CLSTN1	201490000	3111899904	1758599936	15.444438	8.72797585	1.76953256	1.27E-05	0.00025284	0.0005764
Q9BQT9	CLSTN3	0	0	4839700	NaN	NaN	0	1	0.37390098	0.37390098
Q00610	CLTC	392830016	348920000	399620000	0.88822132	1.01728475	0.87312949	0.67842823	0.03421854	0.00166121
P10909	CLU	21710000	409129984	539720000	18.845232	24.8604336	0.7580412	0.00023526	3.10E-05	0.01767678
Q96DG6	CMBL	0	11344000	17313000	NaN	NaN	0.65523016	1	0.37390098	0.37390098
Q9NRP2	CMC2	0	0	2317500	NaN	NaN	0	0.37390098	0.37390098	1
P30085	CMPK1	149680000	98980000	178470000	0.66127741	1.19234371	0.55460304	0.42500192	0.99085921	0.42744964

P62633	CNBP	52779000	14406000	10032000	0.27294946	0.19007559	1.43600476	0.06919289	0.08059372	0.49411145
Q96KP4	CNDP2	38731000	18807000	30456000	0.48558003	0.78634685	0.61751378	0.11684592	0.52120155	0.37390098
Q15417	CNN3	47386000	9953100	13048000	0.21004306	0.27535558	0.76280653	0.11686914	0.11686914	1
Q9Y2B0	CNPY2	21677000	26108000	18752000	1.2044102	0.86506438	1.39227819	0.78789705	0.65782654	0.8459776
Q9BT09	CNPY3	0	3825000	0	NaN	NaN	NaN	0.37390098	1	0.37390098
Q13057	COASY	17373000	0	8796700	0	0.50634319	0	0.37390098	0.37390098	1
P02452	COL1A1	21416000	19476000	17502000	0.90941352	0.81723946	1.11278713	0.88237906	0.37390098	0.37390098
P08123	COL1A2	0	14458000	0	NaN	NaN	NaN	1	0.37390098	0.37390098
P02458	COL2A1	17361000	0	0	0	0	NaN	0.37390098	0.37390098	1
P12109	COL6A1	26480000	19791000	45335000	0.74739426	1.71204686	0.43655014	0.86905915	0.69604945	0.61314088
Q8NBJ5	COLGALT1	41894000	173860000	160970000	4.14999771	3.84231639	1.08007705	0.00633561	3.71E-05	0.02415364
P53621	COPA	13149000	17767000	11752000	1.35120547	0.89375615	1.51182783	0.37390098	1	0.37390098
P53618	COPB1	16152000	22916000	22207000	1.41877162	1.37487614	1.03192687	0.37390098	1	0.37390098
P35606	COPB2	77397000	56817000	55021000	0.7340982	0.71089321	1.03264213	0.20542666	0.10587802	0.44881472
O14579	COPE	0	11820000	10702000	NaN	NaN	1.10446644	1	0.37390098	0.37390098
Q9Y678	COPG1	26311000	29603000	33464000	1.12511873	1.27186346	0.88462228	0.37390098	0.37390098	1
Q9UNS2	COPS3	0	0	8236700	NaN	NaN	0	1	0.20597976	0.59386683
Q9BT78	COPS4	0	0	2525500	NaN	NaN	0	1	0.14275883	0.65083623
Q92905	COPS5	0	0	9470000	NaN	NaN	0	1	0.37390098	0.37390098
Q7L5N1	COPS6	35903000	0	0	0	0	NaN	0.37390098	0.37390098	1
Q9UBW8	COPS7A	720620	0	0	0	0	NaN	1	0.37390098	0.37390098
Q99627	COPS8	0	0	3250700	NaN	NaN	0	0.37390098	0.37390098	1
P61923	COPZ1	18526000	10984000	10621000	0.59289646	0.57330239	1.03417754	0.37390098	0.37390098	1
Q9BR76	CORO1B	8335400	0	0	0	0	NaN	0.37390098	1	0.37390098
Q9ULV4	CORO1C	238540000	129630000	139120000	0.54343086	0.58321458	0.93178552	0.07523368	0.15544176	0.2059014
Q14019	COTL1	127310000	118670000	73516000	0.93213415	0.57745659	1.61420643	0.97637957	0.37390098	0.37390098
Q14061	COX17	187000000	0	145090000	0	0.77588236	0	0.37390098	0.85169506	0.37390098
P14854	COX6B1	0	588290	0	NaN	NaN	NaN	0.37390098	0.37390098	1
P00450	CP	26846000	11269000	16609000	0.41976458	0.6186769	0.67848754	0.25142202	0.40444869	0.1593219
P15088	CPA3	0	206600	0	NaN	NaN	NaN	0.37390098	1	0.37390098
O75976	CPD	0	0	3512700	NaN	NaN	0	1	0.37390098	0.37390098
P36551	CPOX	4250600	4979500	5994600	1.17148173	1.41029501	0.83066428	1	0.37390098	0.37390098
Q9BRF8	CPPED1	48399000	0	15739000	0	0.32519266	0	0.12429719	0.19521257	0.37390098

Q10570	CPSF1	5295700	5256200	9667900	0.99254113	1.82561326	0.54367548	1	0.37390098	0.37390098
Q8N684	CPSF7	0	0	3076300	NaN	NaN	0	1	0.47622752	0.58985627
Q9H3G5	CPVL	10191000	666110016	595740032	65.3625793	58.4574661	1.11812198	2.64E-05	8.75E-05	0.00732756
P29373	CRABP2	3026500	0	0	0	0	NaN	0.37390098	0.37390098	1
O75629	CREG1	0	0	4831400	NaN	NaN	0	1	0.37390098	0.37390098
Q6UXH1	CRELD2	0	187180000	13866000	NaN	NaN	13.4992065	0.00200187	1	0.00200187
Q9NZV1	CRIM1	0	6937800	2796200	NaN	NaN	2.48115301	0.37390098	1	0.37390098
P46108	CRK	25299000	10223000	13059000	0.40408713	0.51618642	0.78283179	0.37390098	0.37390098	1
P46109	CRKL	0	0	243360	NaN	NaN	0	0.37390098	0.37390098	1
Q5TZA2	CROCC	0	0	582990016	NaN	NaN	0	0.37390098	0.37390098	1
P02741	CRP	0	0	175750	NaN	NaN	0	0.37390098	0.37390098	1
O75718	CRTAP	0	7061400	2092200	NaN	NaN	3.37510753	0.37390098	1	0.37390098
Q9Y2S2	CRYL1	0	0	4424900	NaN	NaN	0	1	0.37390098	0.37390098
Q08257	CRYZ	18960000	13640000	22536000	0.71940929	1.18860757	0.60525382	0.37390098	0.37390098	1
O75390	CS	12796000	0	0	0	0	NaN	0.42577291	0.32155457	1
O75534	CSDE1	16073000	0	16655000	0	1.03620982	0	0.37390098	0.37390098	1
P55060	CSE1L	154390000	118010000	260820000	0.76436299	1.68935812	0.45245764	0.74454105	0.05874316	0.00059093
P21291	CSRP1	0	18365000	0	NaN	NaN	NaN	0.47669944	1	0.79898721
P01034	CST3	883969984	2224600064	1727399936	2.51660132	1.95413864	1.28783154	0.00465687	0.05070025	0.00192988
O76096	CST7	24524000	0	0	0	0	NaN	0.37390098	0.37390098	1
P04080	CSTB	0	0	33819000	NaN	NaN	0	1	0.37390098	0.37390098
Q05048	CSTF1	0	0	1784900	NaN	NaN	0	1	0.45688584	0.0756381
P33240	CSTF2	0	0	9468500	NaN	NaN	0	1	0.37390098	0.37390098
Q12996	CSTF3	5620200	6268800	7206000	1.1154052	1.28216076	0.86994171	0.7917617	0.94429022	0.73538667
Q8IX94	CTAGE4	27750000	0	0	0	0	NaN	0.37390098	0.37390098	1
P29279	CTGF	44077000	880460032	955030016	19.9754982	21.6673107	0.92191869	0.01617313	0.00032223	0.68979204
P32929	CTH	0	39037000	410129984	NaN	NaN	0.09518202	1	0.00013254	0.00013254
Q96CG8	CTHRC1	0	4151700	0	NaN	NaN	NaN	0.37390098	1	0.37390098
P35221	CTNNA1	25977000	97960000	112760000	3.77102828	4.34076309	0.86874777	0.11922566	2.65E-05	0.0694821
P35222	CTNNB1	11326000	26426000	27714000	2.33321571	2.44693637	0.9535253	1	3.84E-05	3.84E-05
P17812	CTPS1	53563000	34613000	34167000	0.64621103	0.63788438	1.01305354	0.00056307	0.00056307	1
P10619	CTSA	72604000	1185900032	667190016	16.3338108	9.18943882	1.77745473	0.0056578	0.00735917	0.15672357
P07858	CTSB	53875000	734049984	770830016	13.6250582	14.3077497	0.95228517	0.0003849	0.06842694	0.21649769

P53634	CTSC	165810000	2224499968	1669200000	13.4159575	10.0669441	1.33267426	0.00074066	0.01114035	0.05047913
P07339	CTSD	134800000	1668000000	1188499968	12.3738871	8.81676579	1.40344977	0.00105587	0.00644374	0.1739895
P09668	CTSH	0	12308000	0	NaN	NaN	NaN	0.37390098	1	0.37390098
O60911	CTSL2	0	335510016	432990016	NaN	NaN	0.77486777	3.00E-05	0.00083	0.0469
P25774	CTSS	0	29211000	13943000	NaN	NaN	2.09502983	0.00038422	1	0.00038422
Q9UBR2	CTSZ	108580000	424089984	287440000	3.90578365	2.64726472	1.47540355	0.06845647	0.00499957	0.90695119
Q14247	CTTN	31844000	24890000	56562000	0.78162289	1.77622163	0.4400481	0.12955049	0.99409008	0.11895248
Q13619	CUL4A	10508000	2289200	2959700	0.21785307	0.2816616	0.77345675	1	0.37390098	0.37390098
Q69YN2	CWF19L1	0	0	980710	NaN	NaN	0	1	0.22945863	0.85917211
P49238	CX3CR1	0	0	4459400	NaN	NaN	0	1	0.88080692	0.8416723
P09341	CXCL1	0	117020000	45359000	NaN	NaN	2.57986283	0.03406	0.11911	0.06224
Q9H2A7	CXCL16	0	37279000	10898000	NaN	NaN	3.42071939	0.37390098	1	0.37390098
P19876	CXCL3	0	18673000	4028300	NaN	NaN	4.63545418	0.11734225	1	0.11734225
P99999	CYCS	97242000	120700000	90932000	1.24123323	0.93511033	1.32736552	0.86960655	0.95357561	0.86501426
Q7L576	CYFIP1	0	0	3709200	NaN	NaN	0	1	0.21738926	0.83939427
O00622	CYR61	0	20464000	7798000	NaN	NaN	2.62426257	0.37390098	1	0.37390098
Q14118	DAG1	15929000	174710000	207410000	10.9680452	13.0209055	0.84234124	0.00501308	0.12115284	0.32491821
Q3LXA3	DAK	1770300	0	0	0	0	NaN	0.27572161	0.61332726	1
P14868	DARS	90458000	29060000	40031000	0.32125407	0.44253686	0.72593743	0.27424988	0.53497756	0.10832372
P07108	DBI	139360000	97863000	174850000	0.70223165	1.25466418	0.55969691	0.99656349	0.21753505	0.10261726
Q16643	DBN1	6654300	0	0	0	0	NaN	0.37390098	0.37390098	1
Q9UJU6	DBNL	0	0	15895000	NaN	NaN	0	1	0.37390098	0.37390098
Q9UK59	DBR1	3138100	0	0	0	0	NaN	0.37390098	0.37390098	1
P81605	DCD	40316000	23093000	5834800	0.57279986	0.14472666	3.95780492	0.26147053	0.11915336	0.37390098
Q96C86	DCPS	9089000	7226600	9152400	0.79509294	1.00697541	0.78958523	0.00016603	0.07904297	0.37390098
Q13561	DCTN2	5638200	4511500	12959000	0.80016673	2.29842854	0.34813643	1	0.13580984	0.13580984
Q9H773	DCTPP1	20315000	14050000	12326000	0.69160718	0.60674381	1.13986695	0.01379083	0.11049131	0.38615373
Q96GG9	DCUN1D1	0	0	5482600	NaN	NaN	0	1	0.37390098	0.37390098
Q7Z4W1	DCXR	0	4101900	0	NaN	NaN	NaN	0.37390098	0.37390098	1
O94760	DDAH1	35666000	81551000	110480000	2.28651929	3.09762788	0.73815173	1	0.11776449	0.11776449
O95865	DDAH2	44920000	15302000	15557000	0.34065005	0.3463268	0.98360866	6.10E-06	6.10E-06	1
Q16531	DDB1	150550000	68431000	114760000	0.45454001	0.76227164	0.59629661	0.06470844	0.31635553	0.05695416
P20711	DDC	0	0	8107100	NaN	NaN	0	1	0.37390098	0.37390098

P30046	DDT	47247000	128800000	231920000	2.72609901	4.90867138	0.55536389	0.37390097	0.12476812	0.26734936
Q92499	DDX1	81499000	39936000	70982000	0.49001828	0.87095547	0.56262153	0.37390098	0.99160624	0.37390098
Q92841	DDX17	27950000	29953000	55169000	1.07166374	1.9738462	0.54293174	0.61456203	0.11839025	0.37390098
Q9NR30	DDX21	30123000	15676000	22381000	0.52039969	0.7429871	0.70041555	0.1416225	0.49773896	0.37390098
Q9BUQ8	DDX23	9427600	6206600	6835600	0.65834361	0.72506261	0.90798175	1	0.37390098	0.37390098
O00148	DDX39A	0	0	33480000	NaN	NaN	0	1	0.37390098	0.37390098
Q13838	DDX39B	688880000	251990000	539440000	0.36579666	0.78306818	0.46713257	0.00366048	0.01097876	0.01019232
O00571	DDX3X	57227000	25773000	40347000	0.45036435	0.70503432	0.63878357	0.11613067	0.83054841	0.12410111
Q86XP3	DDX42	8498800	3588900	4073800	0.42228314	0.47933826	0.88097107	1	0.37390098	0.37390098
Q7L014	DDX46	12511000	0	0	0	0	NaN	0.37390098	0.37390098	1
P17844	DDX5	88363000	32222000	46385000	0.3646549	0.52493691	0.69466424	0.00018668	0.00020727	0.09477039
P26196	DDX6	19226000	11678000	24575000	0.60740662	1.27821696	0.47519836	0.37390098	0.48375937	0.12243287
Q01523	DEFA5	21546000	0	0	0	0	NaN	0.37390098	0.37390098	1
O00273	DFFA	0	0	544880	NaN	NaN	0	1	0.93257338	0.20605808
Q9P1J3	DHRS4-AS1	0	657760	0	NaN	NaN	NaN	0.12371916	1	0.06190757
O43143	DHX15	37170000	51790000	68198000	1.39332795	1.83475924	0.75940645	0.60976595	0.00556269	0.0062837
Q08211	DHX9	20270000	24028000	40069000	1.18539715	1.97676373	0.59966558	0.37390098	0.11880948	0.4452914
O60610	DIAPH1	17532000	7640400	15366000	0.43579739	0.87645447	0.49722764	1	0.37390098	0.37390098
Q9Y2L1	DIS3	17462000	0	12503000	0	0.71601194	0	0.11908434	0.11908434	1
P09622	DLD	0	0	16889000	NaN	NaN	0	1	0.37390098	0.37390098
Q9Y2H0	DLGAP4	0	0	2500600	NaN	NaN	0	1	0.37390098	0.37390098
P11532	DMD	0	106070000	0	NaN	NaN	NaN	0.37390098	0.37390098	1
P31689	DNAJA1	0	0	9753800	NaN	NaN	0	1	0.37390098	0.37390098
P25685	DNAJB1	26782000	0	55098000	0	2.0572772	0	0.1189159	0.01249186	3.59E-05
Q9UBS4	DNAJB11	76304000	386860000	68600000	5.06998301	0.89903545	5.63935852	0.0053095	0.11684595	0.01595399
Q8IXB1	DNAJC10	0	37323000	0	NaN	NaN	NaN	1.32E-08	1	1.32E-08
Q99543	DNAJC2	0	0	4717400	NaN	NaN	0	1	0.33315367	0.48946416
Q13217	DNAJC3	0	163910000	209810000	NaN	NaN	0.78123063	0.37390098	0.0001712	0.06727973
Q99615	DNAJC7	11640000	0	20435000	0	1.75558424	0	1	0.37390098	0.37390098
O75937	DNAJC8	64726000	32107000	45371000	0.49604487	0.70097023	0.70765465	0.11637831	0.77112573	0.11692002
Q8WXX5	DNAJC9	48531000	24638000	34682000	0.50767553	0.71463603	0.7103973	1.76E-06	0.05809431	0.11702868
O00115	DNASE2	20491000	556480000	608510016	27.1572895	29.6964531	0.91449606	0.11647889	3.40E-06	0.34157658
O00429	DNM1L	12512000	0	10369000	0	0.82872444	0	0.37390098	0.88935202	0.37390098

P50570	DNM2	4640700	5179500	7896300	1.11610317	1.70153213	0.65594012	1	0.37390098	0.37390098
P26358	DNMT1	35276000	14888000	47005000	0.42204332	1.33249235	0.31673226	0.37390098	0.37390098	1
Q9ULA0	DNPEP	0	0	2983500	NaN	NaN	0	1	0.37390098	0.37390098
Q9BU89	DOHH	28430000	0	0	0	0	NaN	0.37390098	0.37390098	1
Q9NY33	DPP3	313529984	113880000	234980000	0.36321884	0.74946582	0.48463699	0.12283671	0.336624	0.37390098
P27487	DPP4	0	83704000	55630000	NaN	NaN	1.50465572	0.00363787	0.00062387	0.71335405
Q9UHL4	DPP7	0	18166000	0	NaN	NaN	NaN	0.37390098	1	0.37390098
Q6W0C5	DPPA3	76726000	0	0	0	0	NaN	0.74808747	0.49498254	1
Q16555	DPYSL2	211890000	170440000	361440000	0.80437964	1.70579076	0.47155821	0.63229269	0.02891765	0.02613635
Q14195	DPYSL3	26241000	0	0	0	0	NaN	0.37390098	0.37390098	1
Q08554	DSC1	77581000	36259000	5421600	0.46736959	0.06988309	6.68787813	0.24448225	0.00259688	0.00032437
Q02487	DSC2	0	88859000	73263000	NaN	NaN	1.21287692	1	0.12026047	0.12026047
Q02413	DSG1	165170000	36516000	8463000	0.22108132	0.05123812	4.31478214	0.00318215	0.00318215	1
Q14126	DSG2	70189000	544280000	645760000	7.75449133	9.20030212	0.84285182	1.55E-06	2.07E-05	0.00555863
P15924	DSP	153240000	98126000	29148000	0.64034194	0.19021143	3.36647463	0.05883959	0.02615047	0.00021691
P60981	DSTN	77812000	70598000	95869000	0.90728939	1.23205936	0.73640072	0.1334939	0.00144231	0.00013526
P23919	DTYMK	8797400	0	0	0	0	NaN	1	0.1164882	0.1164882
P51452	DUSP3	2640900	0	2929300	0	1.10920525	0	1	0.37390098	0.37390098
P33316	DUT	117950000	57828000	177490000	0.49027553	1.50479019	0.3258099	0.48448753	0.89080799	0.45588219
Q14204	DYNC1H1	53201000	20712000	48797000	0.38931599	0.91721958	0.42445233	0.37390098	0.37390098	1
Q13409	DYNC1I2	11756000	6657700	11316000	0.56632358	0.96257228	0.58834392	0.11734103	0.41475794	0.00022277
Q96FJ2	DYNLL2	22711000	12089000	23541000	0.53229713	1.03654611	0.5135296	0.37390098	0.34337831	0.14334619
Q9NP97	DYNLRB1	59675000	0	0	0	0	NaN	1	0.37390098	0.37390098
P51808	DYNLT3	0	0	5506100	NaN	NaN	0	0.37390098	0.37390098	1
Q13011	ECH1	3765400	10788000	12982000	2.86503434	3.44770813	0.83099675	1	0.14445983	0.14445983
Q9NTX5	ECHDC1	11580000	9591700	20356000	0.82829881	1.7578584	0.47119769	0.83866245	0.39400274	0.28640288
Q16610	ECM1	0	76505000	46440000	NaN	NaN	1.64739454	7.75E-05	4.62E-08	0.0079836
Q5VYK3	ECM29	0	0	10749000	NaN	NaN	0	1	0.37390098	0.37390098
O60869	EDF1	140350000	0	94245000	0	0.67149985	0	0.37390098	0.37390098	1
Q15075	EEA1	84040000	25187000	34291000	0.29970253	0.40803188	0.73450762	0.11787491	0.11787491	1
Q5VTE0	EEF1A1P5	7454199808	1497500032	2531300096	0.20089346	0.33958039	0.59159327	0.00108118	0.00175997	0.40842581
Q05639	EEF1A2	12442000	0	0	0	0	NaN	0.37390098	0.37390098	1
P24534	EEF1B2	15464000	19222000	37390000	1.243016	2.41787386	0.51409465	1	4.05E-05	4.05E-05

P29692	EEF1D	62703000	97558000	186810000	1.55587447	2.97928333	0.52223116	0.37390097	0.37390097	0.64220947
O43324	EEF1E1	15240000	4483100	9113300	0.29416665	0.59798557	0.49192938	0.00450345	0.14629242	0.00019443
P26641	EEF1G	1724199936	841560000	1117200000	0.48808724	0.64795268	0.75327605	0.0483979	0.98248565	0.21009749
P13639	EEF2	4012600064	2074700032	4682599936	0.51704633	1.16697395	0.44306582	0.00459339	0.0131088	2.81E-05
Q12805	EFEMP1	0	0	2752600	NaN	NaN	0	1	0.37390098	0.37390098
Q96C19	EFHD2	24599000	13557000	17134000	0.55111998	0.69653237	0.79123378	0.37390098	0.37390098	1
P98172	EFNB1	0	0	851900	NaN	NaN	0	0.37390098	1	0.37390098
Q7Z2Z2	EFTUD1	0	0	726720	NaN	NaN	0	1	0.37390098	0.37390098
Q15029	EFTUD2	0	5654400	5276100	NaN	NaN	1.07170069	1	0.37390098	0.37390098
P41567	EIF1	0	0	3688000	NaN	NaN	0	1	0.37390098	0.37390098
P47813	EIF1AX	79101000	16256000	50363000	0.20550941	0.63669235	0.32277665	0.00140561	0.10632206	0.01300835
Q9BY44	EIF2A	65545000	53890000	85526000	0.82218325	1.30484402	0.63010079	0.37390098	0.78721291	0.37390098
Q14232	EIF2B1	52403000	26192000	37013000	0.49981871	0.7063145	0.70764327	0.67934895	0.35385492	0.48473868
P05198	EIF2S1	139680000	48886000	83346000	0.34998569	0.59669244	0.58654284	0.02420911	0.7452358	0.02656343
P20042	EIF2S2	20546000	10711000	13433000	0.52131802	0.6538012	0.79736471	0.11705073	0.28522944	0.74503201
P41091	EIF2S3	318800000	112730000	184200000	0.35360727	0.57779169	0.61199784	0.0092266	0.14606719	0.0446551
Q14152	EIF3A	0	0	4420300	NaN	NaN	0	1	0.37390098	0.37390098
P55884	EIF3B	58031000	29544000	47843000	0.50910717	0.82443869	0.6175198	0.03536936	0.00965257	0.22518425
Q99613	EIF3C	12702000	38179000	29925000	3.00574708	2.35592818	1.27582288	0.37390097	0.12063856	0.29949278
O15371	EIF3D	0	0	2149900	NaN	NaN	0	1	0.37390098	0.37390098
P60228	EIF3E	8860400	9927600	11902000	1.12044609	1.3432802	0.83411193	1	0.37390098	0.37390098
O00303	EIF3F	0	4984300	0	NaN	NaN	NaN	1	0.37390098	0.37390098
O75821	EIF3G	92952000	51014000	60633000	0.54882091	0.65230441	0.84135699	5.92E-05	0.01015211	3.56E-07
O15372	EIF3H	5196700	0	2163900	0	0.41639888	0	0.37390098	0.92914319	0.37390098
Q13347	EIF3I	17656000	7227200	13292000	0.40933394	0.75283188	0.54372555	0.30604243	0.56191188	0.73291463
O75822	EIF3J	80070000	40846000	67999000	0.51012862	0.84924442	0.6006853	0.00099728	0.22133447	0.0002057
Q9UBQ5	EIF3K	12091000	0	0	0	0	NaN	0.2660197	0.32797295	1
Q9Y262	EIF3L	8085600	6780700	7719100	0.83861434	0.95467252	0.87843144	1	0.11820129	0.11820129
P60842	EIF4A1	694600000	230870000	589080000	0.33237836	0.84808522	0.39191622	0.00159346	0.92013562	5.85E-05
Q14240	EIF4A2	39280000	42483000	79796000	1.08154273	2.03146648	0.53239512	1	0.15601207	0.15601207
P38919	EIF4A3	110860000	52253000	143870000	0.47134224	1.29776299	0.36319596	0.02879792	0.02111313	0.00310542
P23588	EIF4B	151500000	30189000	68899000	0.19926733	0.45477888	0.4381631	4.82E-05	0.01370593	0.11637923
P06730	EIF4E	14082000	0	0	0	0	NaN	0.37390098	0.37390098	1

Q04637	EIF4G1	59616000	63141000	103040000	1.0591284	1.7283951	0.61278147	0.13664114	0.69963712	0.12273209
Q15056	EIF4H	20893000	0	20335000	0	0.97329247	0	1	0.37390098	0.37390098
P55010	EIF5	0	13763000	46955000	NaN	NaN	0.29311043	1	0.37390098	0.37390098
P63241	EIF5A	1167699968	252800000	787440000	0.21649396	0.67435133	0.32104033	0.00265521	0.61509657	0.00021954
O60841	EIF5B	14342000	7660400	9785200	0.53412354	0.68227583	0.78285575	1	0.37390098	0.37390098
P56537	EIF6	238790000	98159000	222090000	0.41106829	0.93006408	0.44197848	0.02208492	0.34739983	0.02491906
Q15717	ELAVL1	57709000	22699000	40414000	0.39333552	0.70030671	0.56166178	0.37390098	0.78916448	0.37390098
Q9HC35	EML4	0	0	15363000	NaN	NaN	0	1	6.13E-08	6.13E-08
P06733	ENO1	4041299968	2752699904	2884400128	0.68114221	0.71373075	0.95434052	0.81173164	0.36413595	0.59475958
P11171	EPB41	8605000	0	0	0	0	NaN	0.37390098	0.37390098	1
O43491	EPB41L2	29760000	69629000	66484000	2.33968425	2.23400545	1.04730463	0.37390097	0.37390097	0.86128747
P16422	EPCAM	46467000	487190016	257120000	10.4846449	5.53338909	1.89479625	0.00025759	0.11623459	0.01278091
P29317	EPHA2	19890000	20873000	12489000	1.04942179	0.62790346	1.67131078	1	0.37390098	0.37390098
P29323	EPHB2	8994100	7603500	0	0.84538752	0	NaN	0.37390098	0.37390098	1
P54760	EPHB4	0	38010000	42897000	NaN	NaN	0.88607597	0.1161206	0.11746206	0.6506505
P07814	EPRS	29078000	17295000	22841000	0.59477955	0.78550792	0.757191	0.37390098	0.11774249	0.24785739
Q9UBC2	EPS15L1	0	0	1480800	NaN	NaN	0	1	0.37390098	0.37390098
Q9NZ08	ERAP1	0	259430000	78694000	NaN	NaN	3.29669356	0.00146206	8.52E-06	0.00639356
Q6P179	ERAP2	0	136410000	19518000	NaN	NaN	6.98893309	2.35E-05	1	2.35E-05
P84090	ERH	319980000	98773000	176360000	0.30868492	0.55115944	0.56006461	0.37390098	0.98490345	0.17026988
Q96HE7	ERO1L	10680000	175080000	14391000	16.393259	1.34747195	12.1659374	0.00051314	0.1162729	0.00110133
P30040	ERP29	26632000	748659968	98772000	28.1112938	3.70877147	7.57967806	0.07085177	0.00105262	0.1095274
Q9BS26	ERP44	44163000	659649984	290560000	14.9367113	6.57926321	2.27027106	0.00301239	0.00110512	0.0187137
Q9H9K5	ERVMER34-1	56601000	0	0	0	0	NaN	2.11E-05	2.11E-05	1
P10768	ESD	113330000	249770000	323240000	2.20391774	2.85220146	0.77270758	0.9987896	0.68150573	0.70652813
P62495	ETF1	362289984	231120000	361560000	0.63794202	0.99798506	0.63923001	0.01175928	0.76424557	0.08689666
P13804	ETFA	0	0	1244900	NaN	NaN	0	0.37390098	1	0.37390098
O95571	ETHE1	0	2823900	4918100	NaN	NaN	0.57418513	1	0.37390098	0.37390098
Q01844	EWSR1	33734000	17599000	19492000	0.52169919	0.57781464	0.90288323	0.53970271	0.28963926	0.28499478
Q9NQT5	EXOSC3	0	0	1194400	NaN	NaN	0	1	0.37390098	0.37390098
Q16394	EXT1	0	18992000	26719000	NaN	NaN	0.71080506	0.11833327	0.37390098	0.69096076
Q93063	EXT2	0	115660000	79108000	NaN	NaN	1.46205187	0.00079104	0.00032543	0.40209198
P15311	EZR	1722499968	1320400000	2057500032	0.76656026	1.19448483	0.64174968	0.45314255	0.066286	0.13668859

Q9Y624	F11R	0	29597000	13588000	NaN	NaN	2.17817187	0.0002366	1	0.0002366
P00734	F2	1412600	0	0	0	0	NaN	0.37390098	0.37390098	1
Q01469	FABP5	499609984	314049984	680040000	0.62859029	1.36114168	0.46181104	0.13007915	0.05402507	0.02600538
Q9H098	FAM107B	0	0	2524000	NaN	NaN	0	1	0.37390098	0.37390098
Q96TA1	FAM129B	0	5155700	8434000	NaN	NaN	0.61129951	1	0.37390098	0.37390098
Q9GZU8	FAM192A	8195800	0	0	0	0	NaN	1	0.37390098	0.37390098
Q9BTY7	FAM203A	0	0	13870000	NaN	NaN	0	1	0.37390098	0.37390098
Q92520	FAM3C	52794000	253700000	127460000	4.80547047	2.41428947	1.99042833	0.00374087	0.04485051	0.01080075
Q9NUQ9	FAM49B	8651300	10208000	13852000	1.17993832	1.6011467	0.73693329	1	0.11624146	0.11624146
Q9Y285	FARSA	4680800	2485600	2582600	0.53102034	0.55174327	0.96244097	0.72995269	0.99699491	0.17617184
Q9NSD9	FARSB	5668500	3849600	3954500	0.67912143	0.69762725	0.97347325	0.37390098	0.37390098	1
P49327	FASN	337369984	248050000	371230016	0.73524618	1.10036469	0.6681841	0.01201489	0.24513973	0.01935684
Q14517	FAT1	0	50736000	51636000	NaN	NaN	0.98257029	1	0.37390098	0.37390098
P09467	FBP1	0	0	507290	NaN	NaN	0	0.37390098	1	0.37390098
P14324	FDPS	71076000	79147000	116720000	1.11355448	1.64218581	0.6780929	0.64738142	0.06285939	0.09340279
P39748	FEN1	52181000	23161000	60021000	0.44385886	1.15024626	0.3858816	1.72E-06	0.01212906	1.68E-05
Q9UHY8	FEZ2	0	0	66960000	NaN	NaN	0	1	0.37390098	0.37390098
O95750	FGF19	78627000	0	0	0	0	NaN	0.37390098	0.37390098	1
P22455	FGFR4	0	13482000	0	NaN	NaN	NaN	0.37390098	1	0.37390098
P02679	FGG	0	0	6767400	NaN	NaN	0	1	0.37390098	0.37390098
P07954	FH	32518000	31803000	22580000	0.9780122	0.69438463	1.40845883	0.35731259	0.74991387	0.00102611
Q13642	FHL1	6134400	0	0	0	0	NaN	0.93217224	0.29123402	1
Q96AY3	FKBP10	0	27117000	0	NaN	NaN	NaN	0.00038428	1	0.00038428
P62942	FKBP1A	473160000	133340000	155480000	0.28180742	0.32859921	0.85760224	0.22305715	0.63315511	0.01471891
P26885	FKBP2	0	45247000	0	NaN	NaN	NaN	0.04800972	1	0.04800972
Q00688	FKBP3	49999000	27804000	51578000	0.55609113	1.03158069	0.53906703	0.16685517	0.84861296	0.52283192
Q02790	FKBP4	384710016	147730000	248400000	0.38400352	0.64568114	0.59472626	0.01234467	0.15655729	0.00146956
Q13451	FKBP5	0	0	3553700	NaN	NaN	0	0.37390098	0.37390098	1
O95302	FKBP9	0	5861500	0	NaN	NaN	NaN	0.56023139	1	0.20238224
P21333	FLNA	512969984	282830016	405750016	0.55135781	0.79098201	0.69705486	0.00185797	0.03659681	0.00041682
O75369	FLNB	254210000	421100000	1153600000	1.65650451	4.53798056	0.36503121	0.04303143	0.00242055	0.00834466
P02751	FN1	240400000	310489984	208900000	1.29155564	0.86896837	1.48630917	0.37390098	1	0.37390098
Q86XX4	FRAS1	8793700	11011000	5998400	1.25214648	0.68212467	1.83565617	0.37390098	1	0.37390098

Q16658	FSCN1	300550016	130880000	49101000	0.43546829	0.16337048	2.66552615	0.02355265	0.00073427	0.01412452
P02794	FTH1	0	0	8282300	NaN	NaN	0	1	0.37390098	0.37390098
Q96AE4	FUBP1	447780000	140530000	163830000	0.31383714	0.36587164	0.85777938	0.02193959	0.0339742	0.078851
P04066	FUCA1	0	5565800	3106600	NaN	NaN	1.791605	1	0.37390098	0.37390098
Q9BTY2	FUCA2	35134000	300030016	223080000	8.53959179	6.34940529	1.34494364	0.01821941	0.00632811	0.14236861
P35637	FUS	104220000	23392000	19638000	0.22444828	0.18842833	1.19115996	0.39261129	0.50013494	0.78916377
Q9BYC5	FUT8	0	0	3833200	NaN	NaN	0	1	0.37390098	0.37390098
Q16595	FXN	13702000	0	0	0	0	NaN	0.37390098	0.37390098	1
Q13283	G3BP1	44352000	30188000	48507000	0.68064576	1.09368241	0.62234318	0.11889905	0.36427081	0.00110241
Q9UN86	G3BP2	0	5611000	7105100	NaN	NaN	0.78971446	1	0.37390098	0.37390098
P11413	G6PD	58325000	23547000	30118000	0.40372053	0.51638234	0.78182483	0.00608895	0.00060425	0.04564205
P10253	GAA	4249500	28176000	38520000	6.63042688	9.06459618	0.73146415	0.02444682	0.0005708	0.42324951
P60520	GABARAPL2	0	4553200	0	NaN	NaN	NaN	0.37390098	0.37390098	1
P22466	GAL	0	4050800	4596600	NaN	NaN	0.88126004	1	0.37390098	0.37390098
P34059	GALNS	0	2457900	0	NaN	NaN	NaN	1	0.37390098	0.37390098
Q10472	GALNT1	5985000	168810000	31025000	28.205513	5.18379259	5.44109583	0.00918946	0.00113866	0.09405769
Q8IXK2	GALNT12	0	19934000	11488000	NaN	NaN	1.73520195	1	0.37390098	0.37390098
Q10471	GALNT2	27886000	133220000	71120000	4.77730751	2.55038381	1.87317204	0.37390098	0.37390098	0.71784341
Q8N4A0	GALNT4	0	9587900	6340200	NaN	NaN	1.51223934	0.12828426	1	0.12828426
Q7Z7M9	GALNT5	0	24800000	8216800	NaN	NaN	3.0182066	0.12327444	1	0.12327444
Q8NCL4	GALNT6	0	69134000	23724000	NaN	NaN	2.9140954	0.116395	1	0.116395
Q86SF2	GALNT7	56215000	47057000	34964000	0.83708972	0.62196922	1.34587002	0.11612811	0.11612811	1
Q14697	GANAB	131600000	2564400128	605920000	19.4863224	4.6042552	4.23224211	0.00968087	0.00064603	0.03915435
P04406	GAPDH	732590016	898700032	1246400000	1.22674346	1.70136094	0.72103661	0.12963225	0.00717778	0.13453007
P41250	GARS	167980000	142100000	269220000	0.84593403	1.60269082	0.52782112	0.60997552	0.00162068	0.00057887
P22102	GART	66978000	9214700	13605000	0.13757801	0.2031264	0.67730248	0.11629809	0.11629809	1
P04062	GBA	0	151750000	269270016	NaN	NaN	0.56356072	0.00190263	0.00092771	0.06692269
P02774	GC	0	0	172700	NaN	NaN	0	1	0.37390098	0.37390098
P48506	GCLC	34092000	36888000	68665000	1.08201337	2.0141089	0.5372169	0.54906499	0.0115387	0.0133994
P48507	GCLM	6120500	0	7966000	0	1.30152762	0	1	0.37390098	0.37390098
Q02742	GCNT1	17551000	63514000	77393000	3.6188252	4.40960646	0.82066852	0.12392631	0.00015863	0.16419631
O95395	GCNT3	0	352390016	254220000	NaN	NaN	1.38616168	0.00084	7.00E-05	0.67708999
P23434	GCSH	43331000	7887600	0	0.18203133	0	NaN	0.37390098	0.37390098	1

Q9Y2T3	GDA	12341000	12022000	33048000	0.97415119	2.67790294	0.36377391	0.37390098	0.12011748	0.30258456
Q99988	GDF15	494200000	1772000000	919449984	3.58559299	1.8604815	1.92723918	0.00426497	0.09759589	0.00169574
P31150	GDI1	20735000	29058000	37114000	1.40139866	1.78992045	0.78293908	0.50702649	0.12731126	0.59168512
P50395	GDI2	716990016	577969984	705939968	0.80610603	0.98458827	0.81872398	0.73048556	0.14001578	0.00638122
Q06210	GFPT1	49897000	38074000	63875000	0.76305187	1.28013706	0.59607047	0.1904747	0.10300311	0.00579663
O60609	GFRA3	1473300	0	0	0	0	NaN	0.37390098	0.37390098	1
O75223	GGCT	151690000	90805000	120480000	0.5986222	0.79425144	0.75369358	0.03056456	0.14137505	0.07627328
Q92820	GGH	459220000	326380000	683459968	0.71072686	1.48830616	0.47754076	0.08245282	0.01002554	0.00358996
O14908	GIPC1	2345000	2094100	5440800	0.89300638	2.32017064	0.38488826	1	0.11625472	0.11625472
P06280	GLA	15309000	140070000	136260000	9.14951992	8.90064621	1.02796125	0.00047744	0.01079803	0.8858667
P16278	GLB1	0	146090000	116590000	NaN	NaN	1.25302339	6.26E-06	2.26E-05	0.78538841
Q92896	GLG1	295180000	426790016	327169984	1.4458636	1.10837448	1.30449009	0.00065128	0.21417367	0.00791662
Q04760	GLO1	97038000	146830000	304120000	1.51311862	3.13402987	0.48280284	0.42747686	0.00566572	0.01989071
Q9HC38	GLOD4	138930000	64505000	131590000	0.46429858	0.94716763	0.49019682	0.72826576	0.1939307	0.00172186
O76003	GLRX3	141890000	95720000	133910000	0.6746071	0.94375926	0.71480846	0.03864997	0.98216796	0.07225839
P00367	GLUD1	8624700	7889900	8130900	0.91480285	0.94274586	0.97035998	0.90509784	0.37390098	0.37390098
P15104	GLUL	11609000	12069000	0	1.03962445	0	NaN	0.37390098	1	0.37390098
P17900	GM2A	0	486760000	547470016	NaN	NaN	0.88910806	2.32E-05	0.00061534	0.05891546
O60547	GMDS	41295000	26895000	45418000	0.65128952	1.09984255	0.59216613	0.37390098	1	0.37390098
P60983	GMFB	10492000	7787900	9351100	0.74227029	0.89126003	0.83283252	1	0.37390098	0.37390098
Q9P2T1	GMPR2	0	0	4627100	NaN	NaN	0	1	0.11613387	0.11613387
P49915	GMPS	31193000	19871000	30617000	0.63703394	0.9815343	0.64901853	0.0297818	0.66245472	0.03022525
P08754	GNAI3	44661000	31329000	11821000	0.7014845	0.26468283	2.65028334	0.55126709	0.11617639	0.11664219
P62873	GNB1	40393000	22125000	17659000	0.54774344	0.43717971	1.25290215	0.37390098	0.37390098	1
P63244	GNB2L1	231580000	136940000	285260000	0.59132916	1.23179889	0.48005328	0.00191386	0.14876398	0.03230022
P46926	GNPDA1	112430000	77676000	134690000	0.69088322	1.19798982	0.57670206	0.33027929	0.81158459	0.67036372
Q96EK6	GNPNAT1	12207000	12208000	15078000	1.0000819	1.23519289	0.80965644	0.37390098	1.88E-05	0.03309516
P15586	GNS	32991000	576769984	610400000	17.4826469	18.5020161	0.94490498	3.06E-05	4.20E-05	0.00170493
Q8NBJ4	GOLM1	136290000	331449984	170300000	2.43194652	1.2495414	1.94627118	0.00047287	0.00599422	0.00340132
Q9H8Y8	GORASP2	10699000	0	0	0	0	NaN	0.37390098	0.37390098	1
P17174	GOT1	211950000	279800000	387630016	1.32012272	1.82887483	0.72182232	0.00465033	0.00022981	0.00027044
P00505	GOT2	90925000	24296000	20267000	0.26720923	0.22289799	1.19879603	4.09E-06	4.09E-06	1
O75487	GPC4	0	10572000	9289100	NaN	NaN	1.13810813	0.37390098	1	0.37390098

P06744	GPI	937929984	650169984	1103299968	0.69319671	1.17631376	0.58929574	0.09637553	0.03083864	0.00255802
Q86SQ4	GPR126	0	114690000	56732000	NaN	NaN	2.02161026	0.0003783	0.37390098	0.03987844
Q13098	GPS1	0	0	4643600	NaN	NaN	0	1	0.37390098	0.37390098
P62993	GRB2	35377000	28343000	35217000	0.80117023	0.99547726	0.80481017	0.68746793	0.14229748	0.45412514
O43424	GRID2	60903000	0	0	0	0	NaN	0.42302507	0.17017575	1
P28799	GRN	130740000	940289984	786540032	7.19206047	6.01606274	1.19547629	0.00021118	0.00086413	0.34788862
Q96QA5	GSDMA	4946100	2926800	0	0.59173894	0	NaN	0.97073233	0.74534553	0.55573213
P06396	GSN	335600000	708270016	654609984	2.11045885	1.95056605	1.08197248	0.00313437	0.00178966	0.49009329
P15170	GSPT1	137190000	56891000	88523000	0.41468766	0.64525843	0.64266914	0.00387642	0.00387642	1
P00390	GSR	115080000	115910000	177690000	1.0072124	1.5440563	0.65231586	0.3562279	0.00115966	0.02491268
P48637	GSS	364620000	163080000	183320000	0.44726017	0.50277001	0.88959199	0.01777179	0.01409077	0.76453114
P78417	GSTO1	80425000	39803000	96104000	0.4949083	1.19495177	0.41416591	0.12638567	0.1511586	8.80E-06
P09211	GSTP1	582960000	178350000	92384000	0.30593866	0.158474	1.93052912	0.05668044	0.01908123	0.32857892
P78347	GTF2I	14614000	15244000	15334000	1.0431093	1.04926777	0.99413067	0.37390098	0.37390098	1
P08236	GUSB	0	5528000	6748200	NaN	NaN	0.81918138	0.11679506	1	0.11679506
Q4G148	GXYLT1	0	126870000	88567000	NaN	NaN	1.43247485	0.37390098	0.37390098	0.96922821
Q71UI9	H2AFV	326580000	373700000	326460000	1.14428318	0.99963254	1.14470375	0.46099749	0.53607047	0.40697235
Q14520	HABP2	24337000	18886000	20423000	0.77602005	0.83917493	0.92474169	1	0.37390098	0.37390098
P12081	HARS	196170000	82482000	145540000	0.42046183	0.74190754	0.5667308	0.01956577	0.21676427	0.00279019
O14929	HAT1	0	0	4308400	NaN	NaN	0	1	0.37390098	0.37390098
P69905	HBA1	0	0	200200000	NaN	NaN	0	0.69986242	0.12207063	0.11659358
P51610	HCFC1	0	0	9486500	NaN	NaN	0	1	0.37390098	0.37390098
Q13547	HDAC1	0	3743900	0	NaN	NaN	NaN	1	0.37390098	0.37390098
P51858	HDGF	155000000	137920000	168070000	0.88980645	1.08432257	0.82061046	0.70104086	0.49276662	0.28533688
Q00341	HDLBP	7940100	6189600	12439000	0.77953678	1.56660497	0.49759626	1	0.00671375	0.00671375
Q9NRV9	HEBP1	11718000	8331000	8382400	0.71095753	0.71534389	0.99386811	1	0.37390098	0.37390098
Q9Y5Z4	HEBP2	29471000	15295000	17216000	0.51898479	0.58416748	0.88841778	0.23639539	0.30161312	0.34478351
P06865	HEXA	130640000	1016000000	628040000	7.77709723	4.80740976	1.61773133	0.00054179	8.05E-05	0.01077019
P07686	HEXB	47434000	2658099968	1964400000	56.0378609	41.4133339	1.35313582	0.0013677	1.89E-05	0.05651182
O14964	HGS	0	0	7295400	NaN	NaN	0	1	0.28372633	0.54474443
P49773	HINT1	167990000	146520000	270990016	0.87219477	1.61313188	0.5406841	0.11624198	1	0.11624198
P16401	HIST1H1B	196380000	44163000	26056000	0.22488542	0.13268153	1.69492626	0.00174796	1	0.00174796
P16403	HIST1H1C	0	10015000	0	NaN	NaN	NaN	0.37390098	1	0.37390098

P16402	HIST1H1D	0	0	1613000	NaN	NaN	0	1	0.37390098	0.37390098
P10412	HIST1H1E	364929984	197600000	127510000	0.54147375	0.34940949	1.54968238	0.05192491	0.01652193	0.06429178
P62805	HIST1H4A	701950016	1032800000	753600000	1.47132981	1.07358074	1.37048829	0.05028385	0.73053718	0.16965225
Q71DI3	HIST2H3A	74750000	145440000	0	1.94568563	0	NaN	0.70266157	0.69484258	0.29434052
Q8N257	HIST3H2BB	215040000	420640000	231460000	1.95610118	1.07635784	1.81733346	0.896366	0.97144395	0.3577629
P19367	HK1	38518000	20582000	19776000	0.53434759	0.51342231	1.04075646	0.99122125	0.94736749	0.90175968
P52789	HK2	9301000	0	0	0	0	NaN	0.37390098	0.37390098	1
P05534	HLA-A	71661000	15920000	9910300	0.22215711	0.13829419	1.60640955	0.37390098	0.37390098	1
P10316	HLA-A	18069000	0	0	0	0	NaN	0.08556473	0.37024045	1
P17096	HMGA1	55568000	20969000	20097000	0.37735748	0.36166498	1.04338956	0.11831667	0.30779195	0.12160838
P09429	HMGB1	181670000	91586000	320169984	0.50413388	1.7623713	0.28605428	0.90701044	0.06381535	0.04708039
P26583	HMGB2	124850000	64374000	359089984	0.51561075	2.87617135	0.17926984	0.00144871	0.0648166	0.01892043
O15347	HMGB3	0	0	49440000	NaN	NaN	0	1	0.37390098	0.37390098
Q01581	HMGCS1	131360000	23799000	47411000	0.18117388	0.36092418	0.50197214	0.022393	0.26638255	0.30980983
P82970	HMGN5	1625600	0	2114500	0	1.30075049	0	1	0.37390098	0.37390098
P30519	HMOX2	0	0	1507200	NaN	NaN	0	0.37390098	1	0.37390098
Q9UK76	HN1	150350000	59596000	119290000	0.39638177	0.79341537	0.49958923	7.00E-06	0.18125212	0.11664986
Q9H910	HN1L	47345000	19420000	40742000	0.4101806	0.86053437	0.47665799	0.134387	0.88832641	0.11767366
P09651	HNRNPA1	1769799936	657649984	1157600000	0.37159565	0.65408522	0.56811506	0.00085078	0.02179067	0.0581741
P22626	HNRNPA2B1	1650200064	342889984	956940032	0.20778692	0.57989335	0.35831919	0.00011258	0.00617286	0.01645459
P51991	HNRNPA3	66120000	28016000	115940000	0.42371446	1.75347853	0.24164222	0.00260527	0.04011298	2.85E-06
Q99729	HNRNPAB	34715000	0	13488000	0	0.3885352	0	1	0.37390098	0.37390098
P07910	HNRNPC	134310000	84172000	149680000	0.62669945	1.11443675	0.56234634	0.212356	0.10616125	0.00758014
Q14103	HNRNPD	552449984	171970000	435489984	0.31128609	0.78828853	0.39488852	4.00E-05	0.00169793	2.14E-05
P52597	HNRNPF	222700000	123820000	147240000	0.55599463	0.6611585	0.84093994	0.03657754	0.23691653	0.03286924
P31943	HNRNPH1	659660032	198660000	471910016	0.30115512	0.71538365	0.42097008	0.02978136	0.15727173	0.48960778
P31942	HNRNPH3	72946000	0	15794000	0	0.21651633	0	0.37390098	0.37390098	1
P61978	HNRNPK	635329984	216510000	543430016	0.34078354	0.85535079	0.39841378	0.01118699	0.55179399	0.00020812
P14866	HNRNPL	206070000	97555000	190110000	0.47340709	0.92255062	0.51315027	0.37390098	1.80E-06	0.00424414
P52272	HNRNPM	31596000	8349800	28814000	0.26426762	0.91195089	0.28978273	1	0.37390098	0.37390098
O43390	HNRNPR	26627000	21470000	32239000	0.80632442	1.21076345	0.66596359	1	0.11660262	0.11660262
Q00839	HNRNPU	206200000	124960000	165560000	0.6060136	0.80290979	0.75477171	0.11709742	0.81707627	0.13593455
O14979	HNRPDL	45117000	13631000	47808000	0.30212557	1.05964494	0.28511965	0.09996432	0.73456115	0.00199

P00492	HPRT1	243470000	45685000	123530000	0.18764119	0.50737256	0.36982918	0.00524336	0.00859374	0.03168464
Q9Y251	HPSE	16618000	18739000	25021000	1.12763274	1.50565648	0.74893087	0.37390098	1.00E-06	0.04478564
P02790	HPX	191000000	38372000	145710000	0.20090052	0.76287961	0.263345	0.31568164	0.52758217	0.22144632
P04196	HRG	0	0	664570	NaN	NaN	0	1	0.37390098	0.37390098
P52758	HRSP12	0	0	15166000	NaN	NaN	0	1	0.56641477	0.74711186
O14792	HS3ST1	0	53496000	27980000	NaN	NaN	1.91193712	0.37390098	0.37390098	0.60554981
O75506	HSBP1	0	0	2995600	NaN	NaN	0	1	0.38761806	0.0612182
P51659	HSD17B4	5508900	8533700	7527200	1.54907513	1.3663708	1.13371503	1	0.11649209	0.11649209
P07900	HSP90AA1	2461299968	3085499904	7247200256	1.25360584	2.94446039	0.42575061	0.00497231	8.60E-06	1.63E-05
P08238	HSP90AB1	1383399936	1532099968	4102000128	1.10748887	2.96515846	0.3735007	0.04257565	7.77E-05	0.00010845
P14625	HSP90B1	100800000	5339400192	245520000	52.9702415	2.43571424	21.7473125	0.01434094	2.42E-05	0.01648783
P48723	HSPA13	6047400	29491000	23040000	4.87664127	3.80990171	1.27999127	0.37390098	1	0.37390098
P08107	HSPA1A	1886099968	303780000	1817900032	0.16106251	0.96384078	0.1671049	0.5457086	0.03132704	0.33779764
P54652	HSPA2	0	0	17451000	NaN	NaN	0	1	0.37390098	0.37390098
P34932	HSPA4	696849984	270640000	417440000	0.38837627	0.59903854	0.64833272	0.00784559	0.18829902	0.00549698
O95757	HSPA4L	12761000	13342000	18691000	1.04552937	1.46469712	0.7138195	0.37390098	0.75368696	0.37390098
P11021	HSPA5	291520000	6738200064	518020000	23.1140232	1.77696216	13.0076056	0.04317493	0.12220549	0.04664922
P17066	HSPA6	487409984	601929984	1540899968	1.23495626	3.16140413	0.39063534	0.37390098	0.00032688	0.07913956
P11142	HSPA8	7702600192	2456600064	5725899776	0.31893128	0.74337232	0.42903301	0.00147229	0.93664658	0.00023514
P38646	HSPA9	34214000	44498000	44683000	1.30057871	1.30598581	0.99585974	0.94012052	0.49350709	0.39356911
P04792	HSPB1	44214000	77476000	0	1.75229561	0	NaN	0.11746304	0.11746304	1
P10809	HSPD1	147420000	135140000	147390000	0.9167006	0.99979651	0.91688716	0.37756929	0.02106527	0.02256992
P61604	HSPE1	170040000	199870000	208380000	1.17542934	1.22547638	0.95916116	0.89685929	0.34135154	0.4222174
P98160	HSPG2	116970000	1645299968	316270016	14.066	2.70385575	5.20220041	0.00330594	0.01227374	0.00410015
Q92598	HSPH1	729689984	24855000	25946000	0.03406241	0.03555756	0.95795113	2.41E-05	2.41E-05	1
P28221	HTR1D	0	0	2861900	NaN	NaN	0	1	0.37390098	0.37390098
Q9Y4L1	HYOU1	69085000	1058000000	85629000	15.3144674	1.2394731	12.3556271	0.00029265	0.0032805	0.00039719
P41252	IARS	16404000	11524000	23920000	0.70251161	1.4581809	0.48177257	0.24845701	0.30457646	0.13851526
P14735	IDE	57486000	60505000	52404000	1.05251718	0.91159588	1.15458739	0.30166769	0.98075914	0.29614061
O75874	IDH1	31371000	157800000	183840000	5.03012323	5.86018944	0.8583551	0.12461558	1.31E-05	0.04381832
Q13907	IDI1	13368000	14448000	32122000	1.08078992	2.40290236	0.4497852	1	0.37390098	0.37390098
P35475	IDUA	0	12463000	0	NaN	NaN	NaN	0.46347424	1	0.85978109
P78318	IGBP1	7249400	0	0	0	0	NaN	0.37390098	0.37390098	1

P01344	IGF2	53591000	39358000	28449000	0.73441434	0.53085405	1.38345814	0.37390098	0.37390098	1
P11717	IGF2R	0	2718200	0	NaN	NaN	NaN	0.90197057	1	0.11730083
P35858	IGFALS	2783200	0	962420	0	0.3457962	0	0.37390098	0.37390098	1
P08833	IGFBP1	41525000	0	0	0	0	NaN	0.37390098	0.37390098	1
P18065	IGFBP2	485430016	2606899968	5315500032	5.3702898	10.9500856	0.49043363	0.00588239	9.63E-05	0.00666344
P17936	IGFBP3	33913000	781880000	406470016	23.0554657	11.9856701	1.92358589	0.00393396	5.07E-05	0.04749602
P22692	IGFBP4	152300000	219000000	811969984	1.43795145	5.33138514	0.26971442	0.38873672	0.00044468	0.00055587
P24592	IGFBP6	125610000	171670000	212590000	1.36669052	1.69246078	0.80751681	0.37390098	0.70619565	0.37390098
Q969P0	IGSF8	0	55935000	22020000	NaN	NaN	2.5401907	0.00038	0.11658	0.01565
P27930	IL1R2	0	0	7448700	NaN	NaN	0	1	0.37390098	0.37390098
Q12905	ILF2	206050000	96239000	153220000	0.46706626	0.74360591	0.62810993	0.13897684	0.4920077	0.64117408
Q12906	ILF3	440449984	166410000	305489984	0.37781817	0.69358611	0.54473144	0.00063348	0.00566868	0.00369888
P29218	IMPA1	89600000	29669000	74008000	0.33112723	0.82598215	0.4008891	0.00023107	0.32495159	0.00312621
Q9NX62	IMPAD1	0	28621000	63924000	NaN	NaN	0.4477348	1	0.1162161	0.1162161
P12268	IMPDH2	36340000	13878000	23532000	0.38189322	0.64755088	0.58975011	0.11866172	0.3943271	0.00036751
Q27J81	INF2	17313000	0	0	0	0	NaN	0.25753406	0.61970794	1
P09529	INHBB	25620000	0	0	0	0	NaN	0.37390098	0.37390098	1
O00410	IPO5	154670000	52401000	123480000	0.33879226	0.79834485	0.42436832	0.00922844	0.24057762	0.01171695
O95373	IPO7	18314000	0	6703900	0	0.36605328	0	0.37390098	0.37390098	1
Q96P70	IPO9	3205500	2244400	3789900	0.70017159	1.18231165	0.59220558	1	0.37390098	0.37390098
P46940	IQGAP1	193400000	249210000	399649984	1.28857291	2.06644249	0.62357062	0.01038036	0.00011092	0.00060399
Q7Z5L9	IRF2BP2	5886100	0	0	0	0	NaN	0.28003383	0.55837011	1
P05161	ISG15	210950000	24892000	72894000	0.11799952	0.34555107	0.34148216	0.00264053	0.02194175	0.00012645
Q9H0X4	ITFG3	0	2466700	0	NaN	NaN	NaN	0.37390098	1	0.37390098
P23229	ITGA6	28271000	80003000	39650000	2.82986093	1.40249729	2.01773024	0.00034246	0.12466727	0.0223986
P05556	ITGB1	0	19327000	6807000	NaN	NaN	2.83928299	0.37390098	1	0.37390098
P19827	ITIH1	15612000	4176600	7675600	0.26752499	0.49164745	0.54413986	0.30734894	0.45720741	0.12811273
P19823	ITIH2	13511000	0	5554300	0	0.41109467	0	0.37390098	0.4428421	0.37390098
Q14624	ITIH4	4908900	0	1449500	0	0.29528001	0	0.13976501	0.13976501	1
P78504	JAG1	95994000	59427000	51412000	0.61906993	0.53557515	1.1558975	0.03183473	0.04554183	0.60488623
P14923	JUP	37731000	33158000	36648000	0.87879992	0.97129679	0.90476972	0.17194371	0.18444572	0.3966217
Q15046	KARS	510910016	172910000	213140000	0.33843532	0.41717717	0.81125081	0.0001805	0.0002159	0.01575634
Q8IYT4	KATNAL2	0	0	70577000	NaN	NaN	0	1	0.88124144	0.74359101

Q92945	KHSRP	160340000	35217000	126840000	0.21963951	0.79106897	0.27764902	2.75E-05	0.18893836	2.79E-05
Q8IZA0	KIAA0319L	0	0	6867100	NaN	NaN	0	1	0.37390098	0.37390098
Q8WUJ3	KIAA1199	308710016	1179200000	476560000	3.81976604	1.54371405	2.47439981	6.03E-05	0.00183065	0.00019087
P33176	KIF5B	15295000	12953000	20864000	0.84687805	1.36410594	0.62083012	0.00026767	0.00026767	1
Q92876	KLK6	28657000	31090000	0	1.08490074	0	NaN	0.0228162	0.0002705	0.37390098
P03952	KLKB1	229440	0	0	0	0	NaN	0.88511318	0.48778278	1
P01042	KNG1	19028000	4761300	5821900	0.25022599	0.30596489	0.81782579	0.37390098	0.6048286	0.37390098
P52292	KPNA2	8326900	5249300	8068100	0.63040268	0.96891999	0.65062404	1	0.37390098	0.37390098
O00505	KPNA3	30133000	7207700	12663000	0.23919623	0.42023695	0.56919372	0.37390098	0.37390098	1
O00629	KPNA4	23622000	16204000	20183000	0.68597072	0.85441536	0.80285388	1	0.37390098	0.37390098
Q14974	KPNB1	193960000	100960000	213850000	0.52051967	1.10254693	0.47210661	0.43354461	0.15830268	0.00176701
P05783	KRT18	1600300032	799710016	903980032	0.49972504	0.56488162	0.88465452	0.00548574	0.00738682	0.07629978
Q86UP2	KTN1	3020000	2839600	3419500	0.94026488	1.13228476	0.83041382	0.37390098	0.37390098	1
O00515	LAD1	0	0	1815100	NaN	NaN	0	1	0.37390098	0.37390098
Q16787	LAMA3	32413000	630040000	304449984	19.4378796	9.39283562	2.06943679	0.00015711	3.09E-05	0.00072229
O15230	LAMA5	14146000	364169984	47741000	25.7436714	3.37487626	7.62803411	0.00018247	1.50E-06	0.0004865
P07942	LAMB1	18854000	3399000064	815689984	180.280045	43.2634964	4.16702461	0.0043938	7.63E-05	0.02474086
Q13751	LAMB3	0	47181000	24638000	NaN	NaN	1.91496873	0.11875408	1	0.11875408
P11047	LAMC1	14786000	914800000	355360000	61.8693352	24.0335445	2.57429075	2.41E-05	0.00013209	0.00039065
Q13753	LAMC2	0	44683000	26503000	NaN	NaN	1.68596005	7.00E-05	0.12093	0.02203
O43813	LANCL1	5492400	0	0	0	0	NaN	1	0.37390098	0.37390098
Q9NS86	LANCL2	0	0	1897700	NaN	NaN	0	1	0.37390098	0.37390098
P28838	LAP3	45655000	27919000	34740000	0.61152118	0.76092434	0.80365574	0.06193923	0.48010284	0.00890303
Q9P2J5	LARS	56125000	52357000	88280000	0.93286413	1.57291758	0.59307885	0.2236539	0.00200674	0.00181389
Q14847	LASP1	99649000	0	61014000	0	0.61228913	0	0.0051605	0.07743817	0.13603598
P06239	LCK	0	0	6759100	NaN	NaN	0	1	0.37390098	0.37390098
P13796	LCP1	684320000	52109000	84134000	0.07614712	0.12294541	0.61935723	0.00657156	0.00736529	0.37390098
P00338	LDHA	4339999744	1515399936	1836400000	0.34917051	0.42313367	0.82520145	0.00524399	0.01109958	0.17445758
P07195	LDHB	3307899904	1078499968	1446899968	0.32603768	0.43740743	0.74538666	2.48E-05	0.00043254	0.12663516
P01130	LDLR	47531000	331809984	474200000	6.98091745	9.97664642	0.69972581	8.94E-05	0.00504386	0.1204358
Q32P28	LEPRE1	10070000	76081000	15007000	7.55521345	1.49026811	5.06970072	0.00023648	1	0.00023648
Q8NES3	LFNG	0	0	12258000	NaN	NaN	0	1	0.37390098	0.37390098
P09382	LGALS1	707040000	0	6667200	0	0.00942974	0	9.23E-05	9.23E-05	1

P17931	LGALS3	516750016	511380000	1392000000	0.98960811	2.69375896	0.36737069	0.11022737	0.00440122	0.00627956
Q08380	LGALS3BP	680670016	2290299904	1222499968	3.3647728	1.79602444	1.873456	0.00217547	0.00083061	0.01864213
P56470	LGALS4	0	92771000	171250000	NaN	NaN	0.5417285	0.37390098	0.37390098	0.68820405
Q99538	LGMN	320769984	5764700160	3963899904	17.9714451	12.3574524	1.45430017	0.00505461	0.01375796	0.19866696
P48059	LIMS1	19846000	0	0	0	0	NaN	0.37390098	0.37390098	1
Q9NUP9	LIN7C	35769000	16256000	32201000	0.45447174	0.90024883	0.50482905	1	0.11611927	0.11611927
P38571	LIPA	12561000	449940000	296660000	35.8203964	23.6175461	1.51668572	0.37390097	0.00017139	0.32627881
Q12907	LMAN2	48017000	139180000	120290000	2.89855671	2.50515437	1.15703714	0.24026346	0.15818281	0.83560187
P02545	LMNA	1057699968	2012400000	2948900096	1.902619	2.78803086	0.68242395	0.00068338	2.83E-05	0.00060614
P20700	LMNB1	38222000	29703000	41419000	0.77711791	1.08364296	0.71713465	0.82752591	0.22427656	0.07443593
Q03252	LMNB2	33903000	24789000	44896000	0.73117423	1.32424855	0.55214274	0.03984699	0.02659595	0.00747034
Q8WWI1	LMO7	24106000	4780800	15647000	0.19832407	0.64909154	0.30554101	1	0.00076883	0.00076883
Q08397	LOXL1	0	20727000	0	NaN	NaN	NaN	0.12606999	1	0.12606999
O94910	LPHN1	0	0	1502800	NaN	NaN	0	1	0.37390098	0.37390098
O75581	LRP6	7416800	15648000	18925000	2.10980487	2.55163956	0.82684278	0.37390097	0.03213642	0.24400066
Q14114	LRP8	0	3338300	0	NaN	NaN	NaN	0.37390098	1	0.37390098
P30533	LRPAP1	0	95510000	7038600	NaN	NaN	13.5694599	0.37390098	1	0.37390098
Q8N1G4	LRRC47	25533000	16476000	16964000	0.64528257	0.66439509	0.97123319	0.12215819	0.32532403	0.37390098
Q32MZ4	LRRFIP1	15993000	10642000	25193000	0.66541612	1.5752517	0.42241892	1	0.00059342	0.00059342
A6NIV6	LRRIQ4	0	0	0	NaN	NaN	NaN	1	1	1
Q9Y333	LSM2	0	0	2392800	NaN	NaN	0	1	0.37390098	0.37390098
Q86X29	LSR	17079000	322420000	241860000	18.8781548	14.1612511	1.3330853	8.02E-07	0.00033918	0.00257305
P09960	LTA4H	122940000	99216000	129620000	0.80702782	1.05433547	0.76543742	0.16596366	0.62680036	0.00535465
Q14766	LTBP1	0	129940000	85195000	NaN	NaN	1.52520692	0.12221	0	0.68985999
Q8N2S1	LTBP4	16408000	32014000	33326000	1.95112145	2.03108239	0.96063131	1	0.37390098	0.37390098
Q9Y383	LUC7L2	35460000	16606000	15874000	0.46830231	0.44765934	1.04611313	0.00264866	0.02484995	0.37390098
P51884	LUM	116370000	27823000	44041000	0.23909083	0.37845665	0.63175225	0.29591304	0.3492505	0.37390098
Q9NX58	LYAR	7632600	0	2324000	0	0.30448341	0	0.11852887	0.11852887	1
P07948	LYN	11064000	20985000	7182200	1.89669192	0.64915037	2.92180657	0.37390098	1	0.37390098
O95274	LYPD3	0	0	6212800	NaN	NaN	0	1	0.37390098	0.37390098
O75608	LYPLA1	8879100	10674000	24871000	1.20214891	2.80107212	0.42917454	0.21747412	0.24453761	0.35939446
P61626	LYZ	27060000	2618899968	477889984	96.7812271	17.6603832	5.48013163	6.17E-06	0.11955766	0.00048168
Q9UPN3	MACF1	0	0	2882800	NaN	NaN	0	1	0.56367338	0.33046591

Q96A72	MAGOHB	44102000	29896000	42180000	0.67788309	0.95641923	0.70877194	0.58872318	0.85674608	0.70989597
P33908	MAN1A1	82381000	0	22387000	0	0.27174956	0	0.00014277	0.00014277	1
O60476	MAN1A2	0	0	17026000	NaN	NaN	0	1	0.37390098	0.37390098
Q16706	MAN2A1	32497000	161630000	135780000	4.97369003	4.17823172	1.19038153	0.0007745	4.11E-05	0.16273102
O00754	MAN2B1	0	36292000	26980000	NaN	NaN	1.34514451	1.25E-05	0.14123745	0.13891625
Q9Y2E5	MAN2B2	0	8160600	7093700	NaN	NaN	1.15040112	1	0.37390098	0.37390098
O00462	MANBA	0	66335000	53241000	NaN	NaN	1.2459383	0.11804894	7.18E-05	0.7851162
P55145	MANF	55684000	1243699968	116920000	22.3349609	2.09970546	10.637188	0.00540133	0.07959756	0.00741773
P46821	MAP1B	19098000	0	0	0	0	NaN	0.11720661	0.11720661	1
Q02750	MAP2K1	647689984	512270016	0	0.79091853	0	NaN	1	0.37390098	0.37390098
P27816	MAP4	75263000	27487000	63038000	0.36521265	0.83756959	0.43603858	3.02E-05	0.02445298	8.71E-05
P28482	MAPK1	7681900	9466800	13822000	1.23235142	1.79929447	0.68490809	0.37390098	0.94979733	0.37390098
Q8TD08	MAPK15	0	0	19001000	NaN	NaN	0	1	0.06184072	0.58654588
Q15691	MAPRE1	104400000	45544000	97991000	0.4362452	0.93861109	0.46477738	0.37390098	0.91176438	0.37390098
P29966	MARCKS	328720000	69605000	153260000	0.21174556	0.46623266	0.45416287	0.00012331	0.00382127	0.00012594
P49006	MARCKSL1	18900000	8256000	9264800	0.43682539	0.49020106	0.89111477	0.98170257	0.37390098	0.37390098
P56192	MARS	43049000	0	21605000	0	0.50186998	0	0.11719013	0.2833254	0.37390098
P31153	MAT2A	51925000	33456000	47858000	0.64431393	0.9216755	0.69906807	0.37390098	0.11652978	0.24619019
Q9NZL9	MAT2B	54875000	26493000	61710000	0.48278815	1.12455583	0.42931452	0.37390098	0.64768183	0.1261756
P43243	MATR3	0	0	6019700	NaN	NaN	0	1	0.37390098	0.37390098
Q14703	MBTPS1	11287000	22842000	22877000	2.02374411	2.02684498	0.99847007	0.37390098	1	0.37390098
P49736	MCM2	17072000	13124000	30215000	0.76874417	1.76985705	0.4343538	1	8.29E-05	8.29E-05
P33991	MCM4	25504000	7466700	18118000	0.29276586	0.71039838	0.41211504	0.37390098	0.92574555	0.37390098
Q14566	MCM6	87309000	26890000	60907000	0.30798659	0.69760275	0.44149277	0.0007817	0.18131547	0.12023061
P40925	MDH1	285560000	244660000	319700000	0.85677266	1.11955452	0.76527995	0.16326715	0.3933706	0.18987994
P40926	MDH2	437449984	422040000	409649984	0.96477318	0.93644989	1.03024542	0.61775255	0.90839505	0.56425995
P21741	MDK	0	0	2341400	NaN	NaN	0	1	0.37390098	0.37390098
P48163	ME1	29140000	50112000	82269000	1.71969795	2.82323265	0.60912371	0.11685919	0.00013874	0.05758445
Q14696	MESDC2	7346600	157940000	12416000	21.4983807	1.69003344	12.7206831	0.0005487	1	0.0005487
P08581	MET	120200000	476750016	316220000	3.96630621	2.63078213	1.507653	0.02652897	1.31E-05	0.0735203
P50579	METAP2	0	0	2828000	NaN	NaN	0	1	0.37390098	0.37390098
P55001	MFAP2	0	0	17632000	NaN	NaN	0	0.37390098	1	0.37390098
Q08431	MFGE8	0	0	2428300	NaN	NaN	0	1	0.37390098	0.37390098

P26572	MGAT1	5627800	22761000	17481000	4.04438686	3.10618711	1.30204225	1.89E-06	1	1.89E-06
Q09328	MGAT5	4326300	21814000	7636400	5.04218388	1.76511109	2.85658169	0.37390098	0.37390098	1
Q9BRT3	MIEN1	0	0	7124600	NaN	NaN	0	1	0.37390098	0.37390098
P14174	MIF	354190016	312720000	406609984	0.88291591	1.14799953	0.76909083	0.13691102	0.14009662	0.89048177
Q9UNW1	MINPP1	14598000	91663000	49563000	6.27914762	3.39519119	1.849424	0.00213908	0.00573144	0.02399131
P46013	MKI67	14113000	9140300	22042000	0.64765108	1.56182241	0.41467652	1	0.37390098	0.37390098
Q14165	MLEC	1199100	5696200	6072800	4.75039625	5.06446505	0.93798578	1	0.37390098	0.37390098
Q8NB16	MLKL	0	0	854700	NaN	NaN	0	1	0.37390098	0.37390098
P03956	MMP1	0	486169984	0	NaN	NaN	NaN	0.00251001	1	0.00251001
P39900	MMP12	25663000	0	0	0	0	NaN	0.57004923	0.7355445	1
Q9H8S9	MOB1A	852330	0	0	0	0	NaN	0.07012462	0.12843823	1
Q15014	MORF4L2	8963500	3798700	4574100	0.4237965	0.5103029	0.83048034	1	0.37390098	0.37390098
O95297	MPZL1	0	0	4583600	NaN	NaN	0	0.37390098	1	0.37390098
Q13421	MSLN	6701400	0	0	0	0	NaN	0.37390098	0.37390098	1
P26038	MSN	691049984	10756000	5317500	0.01556472	0.00769481	2.02275515	1.76E-05	1.76E-05	1
Q13126	MTAP	0	0	54356000	NaN	NaN	0	1	0.11793209	0.11793209
Q86UE4	MTDH	19853000	11143000	14917000	0.56127536	0.75137258	0.74700004	1	0.37390098	0.37390098
P11586	MTHFD1	150920000	109430000	233210000	0.72508615	1.54525578	0.46923375	0.00749918	0.12311667	0.00039229
P58546	MTPN	33160000	13190000	28372000	0.39776841	0.85560918	0.46489498	0.1532225	0.5490576	0.11700131
P98088	MUC5AC	0	213780000	8286000	NaN	NaN	25.8001442	0.00026898	1	0.00026898
P53602	MVD	0	5777400	5043600	NaN	NaN	1.14549136	1	0.37390098	0.37390098
Q7Z406	MYH14	0	0	750240	NaN	NaN	0	0.37390098	1	0.37390098
Q9Y2K3	MYH15	11741000	0	0	0	0	NaN	0.18098207	0.72648728	1
P35579	MYH9	251490000	353180000	198020000	1.40435004	0.78738719	1.78355718	0.02900806	0.93141586	0.04493656
P19105	MYL12A	11094000	17834000	16686000	1.6075356	1.50405622	1.06880021	4.79E-05	0.00109604	0.09439074
P60660	MYL6	219820000	200550000	238150000	0.91233736	1.08338642	0.8421163	0.64185119	0.24538748	0.02153753
Q9NZM1	MYOF	0	29989000	32910000	NaN	NaN	0.91124278	1	0.37390098	0.37390098
Q9BXJ9	NAA15	14776000	16042000	30868000	1.08567953	2.08906341	0.51969677	1	0.37390098	0.37390098
Q14CX7	NAA25	9106900	0	19936000	0	2.18910933	0	1	0.37390098	0.37390098
O95777	NAA38	6931100	3398800	6607000	0.4903695	0.95323974	0.51442409	1	0.37390098	0.37390098
Q9GZZ1	NAA50	12764000	11668000	23219000	0.91413349	1.81910062	0.50251949	1	0.11893349	0.11893349
Q13765	NACA	160400000	44529000	111080000	0.27761221	0.69251871	0.40087324	0.0021591	0.02097525	0.0568708
P17050	NAGA	0	50013000	35538000	NaN	NaN	1.40731049	0.00014198	9.86E-06	0.08385951

P54802	NAGLU	0	31082000	34230000	NaN	NaN	0.90803391	1	0.12035929	0.12035929
P43490	NAMPT	24125000	13578000	23212000	0.56281865	0.96215546	0.58495605	1	0.37390098	0.37390098
Q9NR45	NANS	8736800	14023000	43023000	1.60504985	4.92434311	0.32594195	1	0.37390098	0.37390098
Q99733	NAP1L4	69946000	108060000	151350000	1.54490602	2.16381216	0.71397424	0.37390097	0.37390097	0.92759109
P54920	NAPA	18964000	22344000	34087000	1.17823243	1.79745829	0.65549916	6.38E-05	0.09575489	0.00027243
O43776	NARS	16470000	21484000	50998000	1.30443227	3.09641767	0.42127141	1	0.0002391	0.0002391
P49321	NASP	215170000	132640000	153420000	0.6164428	0.71301764	0.86455482	0.0836594	0.70592433	0.05358033
P19338	NCL	202970000	133100000	214200000	0.65576196	1.05532837	0.62138188	0.00255603	0.47683874	0.00034402
Q68D85	NCR3LG1	0	0	21182000	NaN	NaN	0	1	0.37390098	0.37390098
Q15843	NEDD8	9836400	7570100	9500000	0.76960069	0.96580052	0.79685265	1	0.37390098	0.37390098
Q9UMX5	NENF	6172600	88897000	10549000	14.4018726	1.70900428	8.42705441	0.11711991	1	0.11711991
P48681	NES	21513000	0	0	0	0	NaN	0.37390098	0.37390098	1
Q99519	NEU1	30220000	162530000	152860000	5.37822628	5.05823946	1.06326056	0.00115371	6.90E-05	0.11012436
Q9UMS0	NFU1	25251000	8498700	9994800	0.33656886	0.395818	0.85031217	0.00011554	0.00011554	1
Q8NBF2	NHLRC2	25552000	26008000	11977000	1.01784599	0.46873042	2.17149544	1	0.11650027	0.11650027
P55769	NHP2L1	18009000	12143000	14499000	0.67427397	0.80509746	0.83750606	0.37390098	0.99940634	0.37390098
P14543	NID1	51162000	0	0	0	0	NaN	1.60E-05	1.60E-05	1
Q9BYT8	NLN	18774000	6650600	19838000	0.35424525	1.05667412	0.33524549	0.00015878	0.23132724	0.00018868
P15531	NME1	3617200128	1493799936	2803399936	0.41297132	0.77501929	0.53285295	0.00504367	0.21482293	0.17552859
P22392	NME2	405280000	183710000	424710016	0.45329157	1.04794216	0.43255398	1	0.37390098	0.37390098
O60361	NME2P1	9498700	0	0	0	0	NaN	0.37390098	0.37390098	1
P30419	NMT1	0	0	2877800	NaN	NaN	0	1	0.37390098	0.37390098
Q15155	NOMO1	2550500	10591000	3825600	4.15251923	1.49994123	2.76845455	0.00234749	1	0.00234749
Q15233	NONO	89766000	42013000	68042000	0.46802798	0.75799298	0.61745685	0.00389932	0.36926982	2.65E-05
P46087	NOP2	12280000	0	24487000	0	1.99405539	0	1	0.37390098	0.37390098
Q6P988	NOTUM	338860000	0	0	0	0	NaN	0.00012	0.00012	1
P48745	NOV	0	40663000	129620000	NaN	NaN	0.31370932	1	0.00323	0.00323
P61916	NPC2	24578000	893990016	999400000	36.3735886	40.6623802	0.89452672	2.68E-05	0.00032083	0.17535941
P55786	NPEPPS	96939000	71632000	134080000	0.73893893	1.38313782	0.53424823	1.15E-05	0.00605165	0.00018336
Q8TAT6	NPLOC4	7131600	0	6436700	0	0.90256041	0	1	0.37390098	0.37390098
P06748	NPM1	96509000	134120000	128320000	1.38971496	1.3296169	1.04519951	0.37390098	0.37390098	0.72753626
Q6UXI9	NPNT	0	13068000	9848400	NaN	NaN	1.3269161	0.37390098	0.37390098	0.92859262
P15559	NQO1	163620000	208620000	283310016	1.27502751	1.73151219	0.73636651	0.14284132	0.00109044	0.10596793

Q86WQ0	NR2C2AP	0	0	10243000	NaN	NaN	0	1	0.37390098	0.37390098
O43847	NRD1	0	6408100	7574200	NaN	NaN	0.84604317	1	0.37390098	0.37390098
O14786	NRP1	0	39186000	51264000	NaN	NaN	0.76439607	0.11657	0.00047	0.26447999
O60462	NRP2	0	3981500	8509800	NaN	NaN	0.46787232	1	0.37390098	0.37390098
Q9UNZ2	NSFL1C	136460000	29175000	25165000	0.21379891	0.18441302	1.15934825	0.0068819	0.00162473	0.95734048
Q08J23	NSUN2	2434700	0	0	0	0	NaN	0.37390098	0.37390098	1
Q5TFE4	NT5DC1	20731000	33293000	44367000	1.60595238	2.14012837	0.75040007	0.80162486	0.00614347	0.17474075
P21589	NT5E	0	7625700	0	NaN	NaN	NaN	0.37390098	1	0.37390098
Q02818	NUCB1	71307000	1128899968	1238300032	15.8315449	17.365757	0.91165304	0.00148585	0.02007052	0.22765519
P80303	NUCB2	0	7539300	2026500	NaN	NaN	3.72035527	0.37390098	1	0.37390098
Q9Y266	NUDC	468460000	252490000	291240000	0.53897876	0.62169665	0.86694825	0.08598687	0.3686448	0.08916093
Q96RS6	NUDCD1	0	0	7795900	NaN	NaN	0	1	0.37390098	0.37390098
P36639	NUDT1	0	0	1403200	NaN	NaN	0	1	0.37390098	0.37390098
O43809	NUDT21	53294000	23084000	28026000	0.43314445	0.52587533	0.82366371	0.13142346	0.44589204	0.37390098
Q9NZJ9	NUDT4	19571000	0	22132000	0	1.13085687	0	0.37390098	0.4915767	0.12419999
Q9UKK9	NUDT5	28816000	35770000	49340000	1.24132431	1.7122432	0.72496963	0.26802224	0.00094274	0.0047873
Q9BW91	NUDT9	5256800	13098000	6666600	2.49162984	1.26818597	1.96471965	0.37390098	1	0.37390098
Q14980	NUMA1	50322000	34515000	34331000	0.68588293	0.68222648	1.00535953	0.37390098	0.37390098	1
Q8NFH4	NUP37	3411800	0	3421400	0	1.00281382	0	1	0.37390098	0.37390098
P61970	NUTF2	231660000	0	0	0	0	NaN	1	0.37390098	0.37390098
Q86UD1	OAF	24645000	25187000	21197000	1.02199233	0.8600933	1.18823421	0.3316395	0.19972454	0.67617655
P04181	OAT	0	2510100	0	NaN	NaN	NaN	0.99043196	1	0.14987722
Q04671	OCA2	3799500	0	0	0	0	NaN	0.37390098	0.37390098	1
Q16625	OCLN	0	0	12847000	NaN	NaN	0	1	0.37390098	0.37390098
Q9NTK5	OLA1	98373000	53136000	110350000	0.5401482	1.12175083	0.48152244	0.12242296	0.77807224	0.1167949
P22059	OSBP	9410900	0	6667200	0	0.70845509	0	1	0.37390098	0.37390098
Q96FW1	OTUB1	66942000	36510000	39360000	0.54539752	0.58797169	0.92759144	0.00013875	0.44465727	0.00029517
Q8N573	OXR1	8895100	0	5721800	0	0.64325303	0	0.37390098	0.37390098	1
P13674	P4HA1	0	172250000	0	NaN	NaN	NaN	6.00E-05	1	6.00E-05
P07237	P4HB	194140000	1693799936	319150016	8.72463131	1.64391685	5.30722189	0.00164757	0.06116297	0.00202734
Q9UQ80	PA2G4	1136400000	425300000	808760000	0.37425202	0.71168602	0.52586675	0.00089666	0.05753196	0.00328548
P11940	PABPC1	171320000	62154000	126530000	0.36279476	0.73855942	0.49121946	0.00059637	0.0776193	0.01563745
Q86U42	PABPN1	45095000	24281000	25944000	0.53844106	0.57531875	0.93590039	0.00197356	0.72843486	0.1219798

Q9UNF0	PACSIN2	34147000	31454000	32154000	0.92113507	0.94163471	0.97822976	0.65459794	0.39950231	0.58101755
P43034	PAFAH1B1	180970000	81084000	194970000	0.44805217	1.07736087	0.41587937	0.01951307	0.31638551	0.00539345
Q15102	PAFAH1B3	41177000	21022000	81846000	0.51052773	1.98766303	0.25684822	0.1198831	0.10133202	0.00556803
P22234	PAICS	421769984	126610000	220810000	0.30018732	0.52353179	0.57338887	0.00026801	0.00126965	0.00092005
Q9H074	PAIP1	16410000	5883200	19758000	0.35851312	1.20402193	0.29776293	0.12793963	0.45474857	0.37390098
Q13153	PAK1	0	0	3104500	NaN	NaN	0	1	0.3575556	0.14659511
Q13177	PAK2	48749000	4149900	7136600	0.0851279	0.1463948	0.5814954	0.37390098	0.37390098	1
P19021	PAM	181790000	245060000	138710000	1.34803891	0.76302326	1.76670754	0.03631053	0.50652874	0.02097039
O95340	PAPSS2	0	0	4803100	NaN	NaN	0	1	0.37390098	0.37390098
Q99497	PARK7	246300000	172190000	190470000	0.69910675	0.7733252	0.90402687	0.04359432	0.19527934	0.26323316
P09874	PARP1	144250000	52875000	61560000	0.36655113	0.42675909	0.85891813	0.0018981	0.00805653	0.04802636
Q9NVD7	PARVA	0	0	392110	NaN	NaN	0	0.37390098	0.37390098	1
Q9BVG4	PBDC1	23146000	0	0	0	0	NaN	0.11939703	0.11939703	1
Q96KB5	PBK	7407800	7636400	23535000	1.03085935	3.17705655	0.32446992	1	9.45E-05	9.45E-05
P61457	PCBD1	179540000	119890000	111310000	0.66776204	0.61997324	1.07708204	0.84824318	0.93159407	0.41549551
Q15365	PCBP1	0	0	44182000	NaN	NaN	0	1	0.37390098	0.37390098
Q15366	PCBP2	313110016	84149000	200270000	0.26875219	0.63961542	0.42017776	0.01430119	0.16906619	0.44346121
Q08174	PCDH1	0	16013000	17080000	NaN	NaN	0.93752927	0.1178803	0.37390098	0.52420133
Q9Y5H3	PCDHGA10	0	38158000	15262000	NaN	NaN	2.50019646	0.37390098	0.37390098	0.65994197
Q5JVF3	PCID2	0	0	2528400	NaN	NaN	0	1	0.37390098	0.37390098
P22061	PCMT1	160740000	72692000	126120000	0.45223343	0.78462112	0.57637173	0.01090129	0.00529566	0.05750129
P12004	PCNA	612009984	323969984	580800000	0.5293541	0.94900411	0.55779958	0.0191813	0.85037595	0.00201534
Q8WW12	PCNP	25821000	12952000	19716000	0.50160724	0.76356453	0.65692836	0.13047732	0.59121603	0.37390098
Q9UHG2	PCSK1N	28173000	0	0	0	0	NaN	0.37390098	0.37390098	1
Q8NBP7	PCSK9	565600000	2.1024E+10	1.0521E+10	37.1711464	18.6014843	1.99828911	0.00113463	0.0046492	0.0127935
Q9UHG3	PCYOX1	0	38240000	0	NaN	NaN	NaN	0.11814912	1	0.11814912
Q13442	PDAP1	35862000	9040400	16600000	0.25208858	0.4628855	0.54460239	0.00617778	0.37344509	0.11844831
Q9BUL8	PDCD10	9102000	5615200	0	0.61691934	0	NaN	0.37390098	0.37390098	1
Q53EL6	PDCD4	0	0	5141300	NaN	NaN	0	1	0.37390098	0.37390098
O14737	PDCD5	28211000	21386000	51811000	0.75807309	1.8365531	0.4127695	1	0.37390098	0.37390098
Q8WUM4	PDCD6IP	310990016	869009984	823880000	2.79433393	2.64921689	1.05477738	1.78E-05	0.00019516	0.29907298
P04085	PDGFA	34377000	0	0	0	0	NaN	0.37390098	0.37390098	1
Q15198	PDGFRL	0	37517000	0	NaN	NaN	NaN	0.37390098	1	0.37390098

P30101	PDIA3	488689984	8580199936	979529984	17.5575523	2.00439954	8.75950718	1.73E-07	0.00290776	3.88E-07
P13667	PDIA4	103260000	4772400128	178490000	46.2173157	1.72854924	26.7376328	0.00883555	0.00011375	0.00980508
Q14554	PDIA5	0	10988000	0	NaN	NaN	NaN	0.37390098	1	0.37390098
Q15084	PDIA6	5153700	183740000	7215200	35.6520576	1.40000391	25.465683	0.06243306	1	0.06243306
O00151	PDLIM1	16460000	22998000	64620000	1.39720535	3.92588091	0.355896	1	0.11690132	0.11690132
Q96HC4	PDLIM5	23750000	0	0	0	0	NaN	0.37390098	0.37390098	1
O00764	PDXK	0	0	6432900	NaN	NaN	0	1	0.37390098	0.37390098
Q15121	PEA15	0	0	1135000	NaN	NaN	0	1	0.37390098	0.37390098
P30086	PEBP1	355640000	381430016	407409984	1.07251716	1.14556849	0.93623137	0.13909696	0.17539732	0.79030728
P12955	PEPD	93693000	43766000	129190000	0.46712133	1.378865	0.33877236	0.37390098	0.3000423	0.11933922
O15067	PFAS	100340000	14717000	24405000	0.14667132	0.24322304	0.60303217	0.00475572	0.00475572	1
O60925	PFDN1	21465000	15137000	23535000	0.70519447	1.09643602	0.64316976	1	0.37390098	0.37390098
Q9UHV9	PFDN2	0	0	6399300	NaN	NaN	0	1	0.37390098	0.37390098
Q9NQP4	PFDN4	328840	0	0	0	0	NaN	0.37390098	0.37390098	1
Q99471	PFDN5	0	0	11075000	NaN	NaN	0	1	0.37390098	0.37390098
O15212	PFDN6	3226500	0	0	0	0	NaN	0.37390098	0.37390098	1
Q01813	PFKP	20295000	23155000	36104000	1.14092135	1.77896035	0.64134169	0.39531854	0.04963307	0.32351631
P35080	PFN2	12224000	0	0	0	0	NaN	0.37390098	0.37390098	1
P18669	PGAM1	920880000	382640000	536670016	0.4155156	0.58277953	0.71298933	0.41436005	0.29469511	0.64619625
P52209	PGD	283980000	158130000	184780000	0.556835	0.65067965	0.85577446	0.24620481	0.14211129	0.87217587
P00558	PGK1	937640000	570289984	727660032	0.60821849	0.7760548	0.78373134	0.07417645	0.10333254	0.36772186
O95336	PGLS	15969000	11718000	15595000	0.73379672	0.97657961	0.75139469	0.37390098	0.12069213	0.34626389
P36871	PGM1	17489000	29756000	31041000	1.70141232	1.77488708	0.95860314	0.12815697	0.00026123	0.43559855
Q96G03	PGM2	32816000	24750000	39572000	0.75420529	1.20587516	0.62544221	0.37390098	0.14449129	0.37345886
O95394	PGM3	23012000	13844000	28866000	0.60159916	1.25438905	0.47959536	1	0.37390098	0.37390098
A6NDG6	PGP	0	0	12399000	NaN	NaN	0	1	0.40682489	0.20964785
Q7RTV0	PHF5A	31755000	21014000	24140000	0.66175407	0.76019526	0.87050539	1	0.37390098	0.37390098
O43175	PHGDH	31560000	152570000	202730000	4.83428383	6.42363739	0.7525773	2.61E-05	1.17E-05	0.00273996
Q96FC7	PHYHIPL	40515000	0	0	0	0	NaN	0.11636227	0.11636227	1
Q9H5I5	PIEZO2	0	0	142020000	NaN	NaN	0	1	0.37390098	0.37390098
Q9Y2I7	PIKFYVE	13569000	0	0	0	0	NaN	0.62886488	0.76613432	1
Q9Y237	PIN4	18339000	24889000	20546000	1.35716236	1.12034464	1.21137929	0.37390098	0.37390098	1
O00625	PIR	0	0	3933700	NaN	NaN	0	1	0.37390098	0.37390098

Q9GZP4	PITHD1	5595900	5564800	7340300	0.99444234	1.31172824	0.75811613	0.37390098	0.41251847	0.11613382
P48739	PITPNB	44379000	21063000	25596000	0.47461638	0.57675928	0.82290202	0.04761934	0.00353456	0.09988476
P14618	PKM	3983600128	6261100032	8126499840	1.57171905	2.03998876	0.7704547	0.02847942	2.75E-06	0.36939156
Q99959	PKP2	0	2067500	0	NaN	NaN	NaN	1	0.37390098	0.37390098
Q8NCC3	PLA2G15	0	0	6136500	NaN	NaN	0	1	0.37390098	0.37390098
Q68DD2	PLA2G4F	0	102280000	0	NaN	NaN	NaN	0.33847278	1	0.20133963
Q03405	PLAUR	0	49148000	45437000	NaN	NaN	1.0816735	1	0.37390098	0.37390098
Q6P4A8	PLBD1	23200000	53681000	39869000	2.3138361	1.71849144	1.34643459	0.00034659	1.41E-05	0.0527971
Q8NHP8	PLBD2	9107900	251020000	211420000	27.5606899	23.2128143	1.18730485	0.04952252	0.00016428	0.73339278
Q8IV08	PLD3	0	18774000	34675000	NaN	NaN	0.54142755	1	0.37390098	0.37390098
Q15149	PLEC	328689984	214290000	393220000	0.65195173	1.19632483	0.54496211	0.00018584	0.00728554	0.00039216
P00747	PLG	3680300	0	3094900	0	0.8409369	0	0.37390098	0.37390098	1
O60664	PLIN3	11130000	0	0	0	0	NaN	0.37390098	0.37390098	1
Q02809	PLOD1	33043000	1108800000	720179968	33.5562744	21.7952347	1.53961515	0.00029508	0.00020791	0.07560463
O60568	PLOD3	98664000	634409984	356000000	6.4300046	3.60820556	1.78205049	0.00365643	3.94E-05	0.18969725
Q04941	PLP2	0	14791000	0	NaN	NaN	NaN	0.37390098	1	0.37390098
O43660	PLRG1	21268000	12736000	21781000	0.59883392	1.02412069	0.58472979	0.37390098	0.86185253	0.37390098
Q14651	PLS1	10190000	23200000	32714000	2.27674198	3.21040225	0.70917648	0.37390097	0.00131042	0.05692803
P13797	PLS3	54941000	16252000	20615000	0.29580823	0.37522069	0.78835797	0.15676765	0.77027875	0.11881045
P55058	PLTP	0	0	1264600	NaN	NaN	0	1	0.03163997	0.52922052
O15031	PLXNB2	1604600	11414000	5863700	7.11329937	3.65430641	1.94655252	0.11728208	1	0.11728208
Q9ULL4	PLXNB3	0	0	99158000	NaN	NaN	0	1	0.84169853	0.24271774
O15305	PMM2	24680000	26476000	24813000	1.07277143	1.00538898	1.06702137	0.37390098	1	0.37390098
P00491	PNP	82825000	31142000	55757000	0.37599757	0.67319047	0.55853075	0.82176828	0.32981387	0.23882455
Q9NVS9	PNPO	6705400	13668000	26792000	2.03835726	3.99558568	0.51015228	0.37390097	0.1171624	0.26188859
O00592	PODXL	0	29426000	31601000	NaN	NaN	0.93117309	1	0.37390098	0.37390098
Q9H488	POFUT1	0	85377000	0	NaN	NaN	NaN	0.37390098	1	0.37390098
Q8NBL1	POGLUT1	0	17242000	0	NaN	NaN	NaN	0.37390098	1	0.37390098
Q9Y2S0	POLR1D	8841700	0	0	0	0	NaN	0.69835967	0.0104382	1
P19388	POLR2E	0	0	4370300	NaN	NaN	0	1	0.37390098	0.37390098
P62487	POLR2G	0	0	0	NaN	NaN	NaN	1	1	1
P52434	POLR2H	0	0	1763900	NaN	NaN	0	1	0.37390098	0.37390098
Q15181	PPA1	286280000	269670016	495260000	0.94197994	1.72998464	0.5445019	0.03422081	0.09406335	0.02797605

Q9H2U2	PPA2	22026000	22398000	34566000	1.0168891	1.56932712	0.64797777	0.11649009	0.12406206	0.47534931
Q06203	PPAT	9389600	5802500	8702300	0.61797094	0.92680198	0.66677773	1	0.37390098	0.37390098
Q9HAB8	PPCS	0	0	1845600	NaN	NaN	0	0.37390098	0.37390098	1
P62937	PPIA	3212100096	2237499904	4053799936	0.69658476	1.26204038	0.55195123	0.03927574	0.33954197	0.0242899
P23284	PPIB	446110016	594140032	397990016	1.33182406	0.89213425	1.49285161	0.00062864	0.84292102	0.01284273
Q08752	PPID	43010000	12205000	34076000	0.28377122	0.79228085	0.35816997	0.00085117	0.23940337	0.11827666
Q9UNP9	PPIE	0	0	2682900	NaN	NaN	0	0.37390098	0.37390098	1
P30405	PPIF	25119000	4642200	4236900	0.18480831	0.16867311	1.09565961	0.37390098	0.37390098	1
Q9Y3C6	PPIL1	20455000	10151000	17085000	0.49626008	0.83524811	0.59414691	1	0.13343062	0.13343062
Q8WUA2	PPIL4	5025300	0	0	0	0	NaN	0.37390098	0.37390098	1
P35813	PPM1A	0	0	5765600	NaN	NaN	0	1	0.37390098	0.37390098
P49593	PPM1F	10488000	0	0	0	0	NaN	0.56987607	0.06599133	1
O15355	PPM1G	14687000	27054000	27652000	1.8420372	1.88275349	0.97837406	1	0.37390098	0.37390098
P62136	PPP1CA	269750016	130650000	235140000	0.48433733	0.87169594	0.55562645	1	0.37390098	0.37390098
P62140	PPP1CB	0	0	3485100	NaN	NaN	0	1	0.30541313	0.40625268
P36873	PPP1CC	0	0	17347000	NaN	NaN	0	0.86637962	0.37390098	0.37390098
Q9UD71	PPP1R1B	0	0	69251000	NaN	NaN	0	1	0.37390098	0.37390098
Q15435	PPP1R7	9244800	8972900	10931000	0.97058886	1.18239439	0.82086724	0.12088022	0.12088022	1
P67775	PPP2CA	0	0	296320	NaN	NaN	0	1	0.37390098	0.37390098
P30153	PPP2R1A	165820000	110010000	273270016	0.66343021	1.6479919	0.40256888	0.04759059	0.02709346	0.01289098
P63151	PPP2R2A	41861000	26539000	61366000	0.63397914	1.46594679	0.43247074	4.23E-08	0.01006149	3.44E-05
Q15257	PPP2R4	6257500	5823100	11589000	0.9305793	1.85201752	0.50246787	1	0.37390098	0.37390098
Q14738	PPP2R5D	0	0	3070600	NaN	NaN	0	1	0.37390098	0.37390098
P60510	PPP4C	5172100	0	4676100	0	0.90410084	0	1	0.11738515	0.11738515
Q9NY27	PPP4R2	0	0	9337400	NaN	NaN	0	1	0.37390098	0.37390098
P53041	PPP5C	23712000	0	28739000	0	1.2120024	0	1	0.11620434	0.11620434
P50897	PPT1	11655000	167840000	95269000	14.4006863	8.17408848	1.76174831	0.00760454	0.0036946	0.77400368
P42785	PRCP	14472000	37752000	17958000	2.6086235	1.24087894	2.10223866	0.123078	0.37390098	0.49919116
Q06830	PRDX1	3395699968	2915800064	4037900032	0.85867423	1.1891216	0.72210807	0.61042124	0.00298385	0.04497255
P32119	PRDX2	469969984	369020000	646110016	0.78519911	1.37478995	0.57114112	0.01349364	0.03148237	0.00247059
P30048	PRDX3	48579000	28130000	35052000	0.5790568	0.72154635	0.80252194	0.37390098	0.8440538	0.37390098
Q13162	PRDX4	28640000	674369984	70999000	23.5464382	2.47901535	9.49830246	0.00323359	0.18694711	0.0046351
P30044	PRDX5	0	0	46520000	NaN	NaN	0	1	0.37390098	0.37390098

P30041	PRDX6	387790016	314950016	435840000	0.81216639	1.12390721	0.72262758	0.04550919	0.26193956	0.01501501
P48147	PREP	13453000	15792000	22987000	1.1738646	1.70868957	0.686997	0.37390098	0.37390098	1
Q92954	PRG4	0	0	1657500	NaN	NaN	0	1	0.37390098	0.37390098
P54619	PRKAG1	0	0	2167300	NaN	NaN	0	1	0.35685807	0.65148336
P14314	PRKCSH	101300000	2363200000	339940000	23.3287258	3.35577488	6.95181513	6.11E-05	0.00017568	0.00010995
P78527	PRKDC	17874000	12431000	31087000	0.69547945	1.73923016	0.39987776	0.37390098	0.37299421	0.11764872
Q99873	PRMT1	216690000	102660000	216170000	0.47376436	0.99760026	0.474904	0.13609667	0.01376206	0.00076629
O14744	PRMT5	43664000	34175000	55664000	0.78268141	1.27482593	0.61395156	0.48565191	0.8762548	0.58843625
Q9NVM4	PRMT7	7038400	0	0	0	0	NaN	0.37390098	0.37390098	1
P04156	PRNP	91612000	28832000	40095000	0.3147186	0.43766099	0.71909213	0.00817182	0.00817182	1
Q9UNN8	PROCR	0	0	5799400	NaN	NaN	0	0.37390098	1	0.37390098
O43490	PROM1	9927600	35246000	11142000	3.55030417	1.12232566	3.16334581	0.00010872	1	0.00010872
P07225	PROS1	11934000	87078000	74404000	7.29663134	6.23462391	1.1703403	0.37390097	0.00026051	0.11389463
O94903	PROSC	55773000	46279000	112060000	0.82977426	2.00921583	0.4129841	0.39348876	0.45454797	0.32238621
Q9UMS4	PRPF19	50608000	30156000	45855000	0.59587419	0.90608203	0.65763819	0.94579846	0.83258379	0.78565681
P60891	PRPS1	0	0	10413000	NaN	NaN	0	1	0.37390098	0.37390098
P21108	PRPS1L1	0	0	20672000	NaN	NaN	0	1	0.37390098	0.37390098
Q9Y520	PRRC2C	7989600	5390000	5514500	0.67462701	0.69020975	0.97742313	0.37390098	0.37390098	1
P07478	PRSS2	5671100	392200000	5121700	69.1576614	0.90312284	76.5761337	0.17928898	1	0.17928898
Q9GZN4	PRSS22	0	173540000	112340000	NaN	NaN	1.54477477	0.37390098	0.37390098	0.62433738
O95084	PRSS23	0	0	6315700	NaN	NaN	0	1	0.37390098	0.37390098
P07602	PSAP	448340000	5831099904	4292499968	13.0059776	9.57420731	1.35843909	6.32E-05	0.00793091	0.03592673
Q6NUJ1	PSAPL1	0	0	6413200	NaN	NaN	0	1	0.80983084	0.82050443
Q9Y617	PSAT1	587870016	141510000	472060000	0.24071647	0.80300063	0.29977122	3.26E-05	0.04488631	0.01238136
O75475	PSIP1	0	0	4248300	NaN	NaN	0	1	0.37390098	0.37390098
P25786	PSMA1	97590000	93728000	113860000	0.96042627	1.16671789	0.8231864	0.92655754	0.27047256	0.22808443
P25787	PSMA2	0	0	6490200	NaN	NaN	0	1	0.37390098	0.37390098
P25788	PSMA3	111820000	85241000	100610000	0.7623055	0.89974958	0.84724182	0.98429286	0.19984467	0.18832222
P25789	PSMA4	90307000	57423000	75248000	0.63586432	0.83324659	0.76311666	0.04273316	0.66521782	0.02918825
P28066	PSMA5	167340000	91844000	130420000	0.54884666	0.77937132	0.70421714	0.01210534	0.35536066	0.03724096
P60900	PSMA6	308649984	157080000	225080000	0.50892597	0.7292403	0.69788522	0.0006322	0.48457271	0.00766484
O14818	PSMA7	521760000	341590016	414350016	0.654688	0.79413909	0.82439965	0.01779914	0.00962972	0.94346297
P20618	PSMB1	250220000	156260000	207990000	0.62449044	0.83122849	0.75128615	0.01294817	0.25557756	0.01796365

P49721	PSMB2	88437000	64184000	73315000	0.72575957	0.82900822	0.8754552	0.03923639	0.21438628	0.42154813
P49720	PSMB3	67286000	37766000	63955000	0.56127572	0.95049489	0.59050894	0.37390098	0.89246237	0.37390098
P28070	PSMB4	84477000	61767000	95412000	0.7311694	1.12944353	0.64737141	0.5152263	0.3300353	0.67483222
P28074	PSMB5	286540000	160590000	314640000	0.56044531	1.09806657	0.51039284	0.04188133	0.9663282	0.00254857
P28072	PSMB6	368900000	196910000	246040000	0.5337761	0.66695583	0.80031705	0.02488448	0.20187035	0.02465094
Q99436	PSMB7	32489000	37472000	53704000	1.15337503	1.65299022	0.69775063	0.11785074	0.11533193	0.00013044
P28062	PSMB8	0	23864000	21433000	NaN	NaN	1.11342323	0.00107021	0.37390098	0.07369059
P35998	PSMC2	28140000	9390000	12303000	0.33368871	0.43720683	0.76322848	0.37390098	0.37390098	1
P17980	PSMC3	2591400	1268200	4104500	0.48938799	1.58389282	0.30897796	1	0.37390098	0.37390098
P62195	PSMC5	58078000	0	0	0	0	NaN	0.13763039	0.13763039	1
P62333	PSMC6	9866600	8495000	9371900	0.86098552	0.94986117	0.90643305	0.37390098	0.98202676	0.37390098
Q99460	PSMD1	23661000	7875200	9450700	0.33283463	0.39942098	0.83329278	0.12253611	0.3427045	0.37390098
O00231	PSMD11	18826000	6092200	8077500	0.32360566	0.42906088	0.75421852	0.11630911	0.11630911	1
O00232	PSMD12	8425300	4481000	5015200	0.53185052	0.59525478	0.89348382	0.00011205	0.1702361	0.00052136
Q9UNM6	PSMD13	59473000	19244000	25312000	0.32357541	0.42560491	0.76027179	0.00038437	0.00038437	1
O00487	PSMD14	4522000	0	0	0	0	NaN	0.13903068	0.30606809	1
Q13200	PSMD2	31938000	15827000	14610000	0.49555388	0.45744881	1.08329916	0.11772002	0.23430827	0.37390098
O43242	PSMD3	63266000	21226000	39018000	0.33550406	0.61672938	0.54400533	0.00364739	0.02108334	0.02443943
P55036	PSMD4	40803000	8172300	19738000	0.20028675	0.48373893	0.4140389	0.37390098	0.62337816	0.37390098
Q16401	PSMD5	0	0	12338000	NaN	NaN	0	1	0.78554374	0.61143255
Q15008	PSMD6	90916000	32850000	38024000	0.36132255	0.4182322	0.86392802	1.33E-05	1.33E-05	1
P48556	PSMD8	36333000	0	0	0	0	NaN	0.41352591	0.35910127	1
O00233	PSMD9	26933000	59150000	57214000	2.1961906	2.12430859	1.03383791	0.0076234	0.00799101	0.16104379
Q06323	PSME1	95818000	113280000	168690000	1.18224132	1.76052511	0.67152768	0.07893224	0.0011476	0.001956
Q9UL46	PSME2	25339000	40352000	60108000	1.5924859	2.37215352	0.67132497	0.37390097	0.11612656	0.42620432
P61289	PSME3	101750000	27716000	49823000	0.27239311	0.48966095	0.55628926	0.00273061	0.01627307	0.00827626
Q92530	PSMF1	8928400	0	0	0	0	NaN	0.37390098	0.37390098	1
O95456	PSMG1	0	0	659560	NaN	NaN	0	0.37390098	0.37390098	1
Q8WXF1	PSPC1	40561000	15756000	45633000	0.38845196	1.12504625	0.34527645	1	0.00010546	0.00010546
P78330	PSPH	167010000	62461000	142900000	0.37399557	0.85563737	0.43709588	0.00010989	0.65013802	0.00474228
P26599	PTBP1	168170000	56911000	105260000	0.33841351	0.62591428	0.54067069	0.01465225	0.21543027	0.41238084
Q15185	PTGES3	67077000	63893000	280760000	0.95253217	4.18563747	0.22757159	1	0.13353321	0.13353321
Q9P2B2	PTGFRN	14884000	138910000	40882000	9.33284092	2.74670792	3.39782786	6.01E-07	0.12302157	0.00134222

Q13308	PTK7	102230000	0	0	0	0	NaN	0.00014	0.00014	1
P06454	PTMA	16771000	0	64367000	0	3.8379941	0	1	0.00528477	0.00528477
P20962	PTMS	82375000	0	0	0	0	NaN	1	0.37390098	0.37390098
Q06124	PTPN11	13605000	14174000	14109000	1.04182291	1.03704524	1.00460696	1	0.37390098	0.37390098
P10586	PTPRF	17501000	51097000	49981000	2.91966176	2.85589385	1.0223285	0.37390097	0.00113444	0.19660054
P23471	PTPRZ1	0	0	9813300	NaN	NaN	0	0.37390098	1	0.37390098
Q6GMV3	PTRHD1	22182000	3751500	6085100	0.16912362	0.27432603	0.61650586	0.37390098	0.37390098	1
Q9UHX1	PUF60	121340000	52491000	75823000	0.43259436	0.62488049	0.69228333	0.00294289	0.40430528	0.11798262
Q96PZ0	PUS7	0	0	5801200	NaN	NaN	0	1	0.37390098	0.37390098
P15151	PVR	0	9636900	0	NaN	NaN	NaN	0.37390098	0.37390098	0.72509009
Q15223	PVRL1	57810000	20455000	0	0.35383153	0	NaN	0.37390098	0.37390098	1
Q92692	PVRL2	0	0	1669800	NaN	NaN	0	1	0.88204294	0.03477364
P11216	PYGB	158670000	412009984	514240000	2.59664702	3.24094033	0.80120176	0.00327085	5.67E-05	0.02462
P06737	PYGL	37978000	33516000	57452000	0.8825109	1.51277053	0.58337396	0.59295213	0.03971928	0.06567125
P47897	QARS	17169000	19849000	67135000	1.15609527	3.91024518	0.29565799	1	3.32E-06	3.32E-06
P09417	QDPR	31711000	12159000	13503000	0.38343161	0.42581439	0.90046656	0.00223605	0.02879429	0.32371911
Q16769	QPCT	24685000	0	0	0	0	NaN	7.74E-06	7.74E-06	1
O00391	QSOX1	129920000	650870016	299670016	5.00977516	2.30657339	2.17195582	0.00417144	0.02877949	0.03594544
Q6ZRP7	QSOX2	0	6822800	7876600	NaN	NaN	0.8662113	1	0.37390098	0.37390098
P62491	RAB11A	40562000	30707000	34029000	0.75703859	0.83893794	0.90237737	0.49541989	0.61273474	0.80339491
P61106	RAB14	4872600	5141900	8867100	1.05526829	1.81978822	0.57988518	1	0.37390098	0.37390098
P61019	RAB2A	11484000	12013000	17943000	1.04606414	1.56243467	0.66950899	0.37390098	0.00037879	0.05155813
P51148	RAB5C	13923000	14652000	20663000	1.05235946	1.48409104	0.70909357	6.95E-08	0.00232403	0.10881611
Q9NRW1	RAB6B	44123000	0	38477000	0	0.8720395	0	1	0.37390098	0.37390098
P51149	RAB7A	31295000	31319000	49497000	1.00076687	1.58162642	0.63274544	0.52790564	0.83157825	0.75760269
Q92696	RABGGTA	0	0	8370600	NaN	NaN	0	1	0.11623767	0.11623767
P53611	RABGGTB	0	0	4737900	NaN	NaN	0	1	0.37390098	0.37390098
P63000	RAC1	88922000	83471000	117280000	0.93869907	1.31890869	0.7117241	0.49905038	0.11636129	0.37390098
P54725	RAD23A	0	17917000	0	NaN	NaN	NaN	1	0.37390098	0.37390098
P54727	RAD23B	57932000	26554000	71415000	0.45836499	1.23273838	0.37182665	1	0.0003089	0.0003089
P78406	RAE1	0	0	5696200	NaN	NaN	0	0.37390098	0.37390098	1
P11233	RALA	6226500	8904100	0	1.43003297	0	NaN	0.37390098	1	0.37390098
P62826	RAN	765840000	542700032	1073799936	0.70863372	1.40212047	0.50540143	0.02788981	0.00647793	0.00215617

P43487	RANBP1	51815000	10146000	30431000	0.19581202	0.58730096	0.33341002	1	0.01166592	0.01166592
Q9H6Z4	RANBP3	0	0	5702700	NaN	NaN	0	1	0.37390098	0.37390098
P46060	RANGAP1	17636000	20561000	40522000	1.16585398	2.29768658	0.50740337	1	7.17E-05	7.17E-05
Q9HD47	RANGRF	0	0	2441400	NaN	NaN	0	1	0.36495355	0.1573851
P61224	RAP1B	5690800	10685000	6289000	1.87759185	1.10511708	1.69899821	0.37390098	1	0.37390098
P49788	RARRES1	0	470980000	256060000	NaN	NaN	1.83933449	0.00243	0.11876	0.11159
Q99969	RARRES2	16617000	0	0	0	0	NaN	0.37390098	0.37390098	1
P54136	RARS	35716000	34764000	66499000	0.97334528	1.86188269	0.52277476	0.8324995	0.01334196	0.03784527
Q09028	RBBP4	99900000	86890000	130760000	0.86976975	1.30890894	0.66449982	0.25420907	0.96208584	0.37445003
Q15291	RBBP5	0	0	1898200	NaN	NaN	0	1	0.37390098	0.37390098
Q16576	RBBP7	53107000	36222000	65027000	0.68205696	1.2244525	0.55703014	0.1177027	0.53384501	0.37390098
Q9NTZ6	RBM12	14097000	0	11013000	0	0.78123003	0	0.11788207	0.11788207	1
P98179	RBM3	56666000	14764000	14575000	0.26054424	0.25720891	1.01296747	0.37390098	0.37390098	1
Q9BQ04	RBM4B	1773200	0	0	0	0	NaN	0.926503	0.39636508	1
Q9Y5S9	RBM8A	104900000	66968000	79902000	0.63839847	0.76169688	0.83812672	0.00438556	0.17793404	0.37390098
P38159	RBMX	453110016	142950000	225710000	0.31548628	0.49813509	0.63333482	0.00425566	0.10330122	0.21810457
P09455	RBP1	61996000	0	0	0	0	NaN	0.00889785	0.00889785	1
P02753	RBP4	37237000	55804000	19383000	1.49861693	0.52053064	2.87901759	2.53E-05	1	2.53E-05
P18754	RCC1	52274000	59091000	103060000	1.130409	1.97153461	0.57336503	0.41842148	0.0541909	0.04270803
Q9P258	RCC2	118410000	91337000	160760000	0.77136225	1.35765564	0.56815749	0.1475603	0.01747964	0.02588584
Q15293	RCN1	83534000	924129984	117710000	11.0629206	1.40912688	7.85090446	0.00773361	0.00127376	0.01045326
P35241	RDX	9181100	0	0	0	0	NaN	0.00172215	0.00172215	1
P46063	RECQL	829070	0	0	0	0	NaN	0.37390098	0.37390098	1
P05451	REG1A	0	0	13770000	NaN	NaN	0	1	0.37390098	0.37390098
Q9BYZ8	REG4	11922000	1.5697E+10	4405300224	1316.6416	369.510162	3.56320786	0.0516848	0.03004984	0.12218269
Q9Y3B8	REXO2	0	0	11644000	NaN	NaN	0	1	0.37390098	0.37390098
P61586	RHOA	24464000	18012000	15684000	0.73626554	0.64110529	1.14843154	0.37390098	0.37390098	1
P34096	RNASE4	0	50446000	34044000	NaN	NaN	1.48178828	0.00013	0.12157	0.36833
O00584	RNASET2	0	237880000	66852000	NaN	NaN	3.55830789	4.34E-05	6.42E-06	0.00016749
P13489	RNH1	23730000	11398000	37580000	0.48032027	1.5836494	0.30329964	0.00648396	0.0844488	0.00519989
O43148	RNMT	7469300	0	3486800	0	0.4668175	0	0.37390098	0.37390098	1
Q9H4A4	RNPEP	12752000	28857000	24526000	2.26293921	1.92330611	1.17658806	0.37390097	0.37390097	0.83244771
Q9Y6N7	ROBO1	56464000	12616000	11158000	0.2234344	0.19761264	1.13066852	5.98E-05	5.98E-05	1

O75116	ROCK2	0	43573000	67508000	NaN	NaN	0.6454494	1	0.37390098	0.37390098
O75695	RP2	0	0	3663300	NaN	NaN	0	1	0.27244875	0.73449594
P27694	RPA1	16523000	15618000	22523000	0.94522786	1.36313021	0.69342452	0.37390098	0.10427648	1.73E-05
P35244	RPA3	0	0	13740000	NaN	NaN	0	1	0.41709736	0.56406724
P62906	RPL10A	7414100	0	75434000	0	10.1743975	0	1	0.37390098	0.37390098
P62913	RPL11	12912000	14844000	9288600	1.14962828	0.71937734	1.59808803	0.37390098	1	0.37390098
P30050	RPL12	134240000	113700000	306190016	0.84699047	2.28091478	0.37133804	0.00507419	0.10980549	0.05097647
P26373	RPL13	8294300	12987000	5419800	1.56577408	0.65343672	2.39621377	0.37390098	1	0.37390098
Q6NVV1	RPL13A	0	67598000	0	NaN	NaN	NaN	1	0.37390098	0.37390098
Q07020	RPL18	0	0	4758800	NaN	NaN	0	1	0.37390098	0.37390098
P84098	RPL19	0	48267000	341320000	NaN	NaN	0.14141275	1	0.37390098	0.37390098
Q6P5R6	RPL22L1	0	0	34586000	NaN	NaN	0	1	0.37390098	0.37390098
P62750	RPL23A	0	10543000	9711400	NaN	NaN	1.08563137	0.37390098	1	0.37390098
P83731	RPL24	0	0	2704600	NaN	NaN	0	1	0.04030157	0.94044709
P46776	RPL27A	0	10479000	0	NaN	NaN	NaN	0.37390098	1	0.37390098
P62888	RPL30	23357000	16023000	17377000	0.68600422	0.74397397	0.92208093	1	0.37390098	0.37390098
P42766	RPL35	0	0	2571900	NaN	NaN	0	1	0.37390098	0.37390098
Q02878	RPL6	30043000	44129000	22150000	1.46886134	0.73727655	1.99227989	0.37390098	1	0.37390098
P18124	RPL7	57558000	112120000	51557000	1.9479481	0.89573997	2.17468047	0.39239153	0.37390098	0.12239096
P62424	RPL7A	0	35265000	0	NaN	NaN	NaN	1	0.37390098	0.37390098
P62917	RPL8	0	0	12259000	NaN	NaN	0	1	0.69159311	0.06922211
P05388	RPLP0	148430000	70102000	160720000	0.47228998	1.08280003	0.43617472	0.02137949	0.41218686	0.01494651
P05387	RPLP2	105730000	62965000	115150000	0.59552634	1.08909488	0.54680848	0.11956284	0.72431087	0.27181232
P78346	RPP30	0	0	499260000	NaN	NaN	0	1	0.98265058	0.2642172
Q9NQG5	RPRD1B	8223100	4260200	6055600	0.51807714	0.7364133	0.7035141	1	0.37390098	0.37390098
P46783	RPS10	46188000	28908000	24504000	0.62587684	0.53052741	1.17972577	0.37390098	0.63546079	0.37390098
P62280	RPS11	8810600	10999000	0	1.24838269	0	NaN	0.37390098	1	0.37390098
P25398	RPS12	146420000	45164000	101040000	0.30845514	0.69006968	0.44699129	0.00661242	0.03922459	0.26613203
P62263	RPS14	27961000	12627000	9430600	0.45159328	0.33727694	1.33893919	0.12494145	0.12494145	1
P62244	RPS15A	24785000	0	12016000	0	0.48480937	0	1	0.37390098	0.37390098
P62269	RPS18	5971300	19382000	18128000	3.24585938	3.03585482	1.06917477	0.37390097	0.12323273	0.7160154
P39019	RPS19	18345000	18705000	33051000	1.01962388	1.80163527	0.56594354	1	0.37390098	0.37390098
P60866	RPS20	60981000	23703000	82757000	0.38869485	1.35709488	0.28641686	0.20292708	0.80512953	0.18137273

P63220	RPS21	246150000	55680000	119820000	0.22620353	0.48677635	0.46469703	0.00947502	0.06950229	0.01207179
P62851	RPS25	23856000	25632000	10639000	1.07444668	0.44596747	2.40924907	1	0.37390098	0.37390098
P42677	RPS27	20900000	11490000	11297000	0.54976076	0.54052633	1.01708424	0.37390098	0.37390098	1
P62857	RPS28	20118000	7412600	12577000	0.3684561	0.62516153	0.58937746	0.20745318	0.47843477	0.24047489
P23396	RPS3	77361000	56785000	67741000	0.73402619	0.87564796	0.83826631	0.0072253	0.15339293	0.00840781
P62701	RPS4X	25103000	0	0	0	0	NaN	0.12682673	0.58049572	1
P51812	RPS6KA3	5208000	0	7145400	0	1.37200463	0	1	0.37390098	0.37390098
P62241	RPS8	0	13142000	11333000	NaN	NaN	1.15962231	0.37390098	0.11900762	0.64364249
P46781	RPS9	13494000	15829000	14626000	1.17303991	1.08388913	1.08225083	0.37390098	1	0.37390098
P08865	RPSA	144680000	96238000	229490000	0.6651783	1.58619022	0.41935596	0.00210347	0.00041185	2.95E-05
Q9P2E9	RRBP1	41252000	83707000	62902000	2.02916217	1.52482307	1.33075261	0.11925778	0.37390097	0.38589922
P23921	RRM1	336440000	176420000	279740000	0.52437282	0.83147067	0.63065702	0.12083194	0.32194617	0.37390098
P31350	RRM2	11470000	9183700	16433000	0.80067134	1.43269396	0.5588572	1	0.37390098	0.37390098
O43818	RRP9	0	0	2693600	NaN	NaN	0	1	0.37390098	0.37390098
Q15404	RSU1	0	0	37138000	NaN	NaN	0	1	0.37390098	0.37390098
Q96T51	RUFY1	0	0	1091500	NaN	NaN	0	1	0.37390098	0.37390098
Q9Y265	RUVBL1	48939000	16209000	28360000	0.33120823	0.57949692	0.57154441	0.12581928	0.12581928	1
Q9Y230	RUVBL2	43650000	24199000	29690000	0.55438715	0.68018329	0.81505555	0.0146356	0.0464806	0.04280801
P31949	S100A11	164510000	465140000	882540032	2.82742691	5.36465883	0.52704692	0.37390097	0.11614528	0.40418264
Q99584	S100A13	29457000	0	0	0	0	NaN	0.37390098	0.37390098	1
Q9HCY8	S100A14	0	55564000	65828000	NaN	NaN	0.84407848	0.00012	0.00111	0.13449
Q96FQ6	S100A16	11897000	11375000	22468000	0.95612341	1.88854337	0.50627559	1	0.37390098	0.37390098
P33764	S100A3	14038000	0	0	0	0	NaN	0.37390098	0.37390098	1
P26447	S100A4	186060000	0	0	0	0	NaN	0.12552842	0.12552842	1
P06703	S100A6	296590016	1515200000	628040000	5.10873556	2.11753583	2.41258526	0.1860873	0.00532289	0.69743633
P06702	S100A9	5056300	265930000	4351700	52.5937958	0.86064911	61.1094513	0.02011796	1	0.02011796
P25815	S100P	18131000	133390000	263020000	7.35701275	14.5066462	0.50714773	1	0.11723614	0.11723614
Q9UBE0	SAE1	1828100	0	0	0	0	NaN	0.9467113	0.34789151	1
P82979	SARNP	115590000	28111000	136490000	0.24319577	1.18081152	0.20595647	0.00365241	0.0023057	0.0006017
P49591	SARS	43780000	16142000	38951000	0.36870718	0.88969851	0.4144181	0.37390098	0.2988041	0.11612496
Q9Y3A5	SBDS	66509000	46065000	100700000	0.69261301	1.51408076	0.45744786	1	3.55E-06	3.55E-06
Q6UWP8	SBSN	45549000	43083000	0	0.94586051	0	NaN	0.55307966	0.02114413	8.90E-05
Q8WTV0	SCARB1	0	6673300	0	NaN	NaN	NaN	0.37390098	1	0.37390098

P22307	SCP2	0	40712000	28726000	NaN	NaN	1.41725266	1	0.37390098	0.37390098
Q9HB40	SCPEP1	0	170690000	130230000	NaN	NaN	1.3106811	6.93E-05	0.11669547	0.26840094
Q12765	SCRN1	9559300	0	0	0	0	NaN	0.37390098	0.37390098	1
P18827	SDC1	0	0	6270200	NaN	NaN	0	1	0.37390098	0.37390098
P31431	SDC4	69591000	340009984	324710016	4.88583279	4.665977	1.0471189	0.0109396	0.01343558	0.84282488
O00560	SDCBP	15757000	129160000	111810000	8.19699192	7.09589386	1.1551739	0.11673261	0.12446739	0.66870391
Q9HCN8	SDF2L1	0	19395000	0	NaN	NaN	NaN	0.37390098	1	0.37390098
Q9BRK5	SDF4	92562000	898310016	341689984	9.7049551	3.69147158	2.62902069	0.00464001	0.010732	0.01288618
P55735	SEC13	0	0	6350800	NaN	NaN	0	1	0.37390098	0.37390098
Q15437	SEC23B	29871000	28465000	30293000	0.95293093	1.01412737	0.93965602	1	0.37390098	0.37390098
P53992	SEC24C	10452000	9481000	10865000	0.90709913	1.03951395	0.8726185	0.37390098	0.75064158	0.37390098
O94979	SEC31A	0	0	2243200	NaN	NaN	0	0.37390098	0.37390098	1
Q14563	SEMA3A	51192000	8566500	5999400	0.16734061	0.11719409	1.4278928	0.37390098	0.37390098	1
Q9NPR2	SEMA4B	0	8349500	18217000	NaN	NaN	0.45833561	1	0.37390098	0.37390098
Q9NTN9	SEMA4G	0	0	2690300	NaN	NaN	0	1	0.30154926	0.05365916
P49903	SEPHS1	0	0	2804700	NaN	NaN	0	1	0.37390098	0.37390098
Q9NVA2	SEPTIN11	29532000	39775000	62842000	1.34684408	2.12792897	0.6329366	0.37390098	0.83587486	0.37390098
Q15019	SEPTIN2	24766000	24233000	32040000	0.97847855	1.29370916	0.75633585	0.37390098	0.83219868	0.37390098
Q16181	SEPTIN7	60914000	69027000	105410000	1.13318777	1.73047245	0.65484297	0.81107759	0.80636561	0.99464661
Q9UHD8	SEPTIN9	22399000	15423000	14361000	0.68855751	0.64114469	1.07395029	0.11990477	0.66481841	0.12737572
Q8NC51	SERBP1	165460000	100070000	132760000	0.60479873	0.80236918	0.75376618	0.00557544	0.00288111	0.03935665
Q86VE9	SERINC5	0	0	44096000	NaN	NaN	0	1	0.37390098	0.37390098
P01009	SERPINA1	158110000	4340000	0	0.02744924	0	NaN	0.02001458	0.02001458	1
Q9UK55	SERPINA10	487700	0	0	0	0	NaN	0.37390098	0.37390098	1
P01011	SERPINA3	12754000	19416000	7687500	1.5223459	0.60275209	2.52565861	0.22551811	0.59030223	0.37390098
P30740	SERPINB1	214600000	324049984	508720000	1.51001859	2.37054992	0.63699085	0.17394854	0.01437683	0.05337016
P29508	SERPINB3	0	3578000	0	NaN	NaN	NaN	0.37390098	1	0.37390098
P48594	SERPINB4	30839000	411150016	0	13.3321447	0	NaN	0.37390098	1	0.37390098
P36952	SERPINB5	0	60585000	85499000	NaN	NaN	0.70860475	0.00414	0.00029	0.64002001
P35237	SERPINB6	9555200	17606000	26079000	1.84255695	2.72929931	0.67510259	1	0.11622849	0.11622849
P01008	SERPINC1	43390000	37209000	31536000	0.85754782	0.72680342	1.17988968	0.37390098	0.67119277	0.37390098
P05121	SERPINE1	22899000	0	0	0	0	NaN	0.12819979	0.12819979	1
P36955	SERPINF1	265690000	126910000	74230000	0.47766194	0.27938575	1.70968616	0.12803312	0.07677179	0.54281348

P50454	SERPINH1	0	0	8297500	NaN	NaN	0	1	0.37390098	0.37390098
Q99574	SERPINI1	148390000	2159200	1584400	0.01455085	0.01067727	1.36278713	1.95E-07	1.95E-07	1
Q01105	SET	127040000	267490000	545670016	2.1055572	4.29526138	0.49020469	0.00062649	0.00066942	0.00341772
Q15637	SF1	52424000	9299900	15291000	0.17739776	0.29167938	0.60819435	0.37390098	0.37390098	1
Q15459	SF3A1	26431000	16854000	23285000	0.63766032	0.8809731	0.72381359	0.14943299	0.86371493	0.11914762
Q12874	SF3A3	8367300	5250200	7662000	0.62746644	0.91570759	0.68522578	0.37390098	0.37390098	1
Q13435	SF3B2	109240000	75962000	82484000	0.69536799	0.7550714	0.92093015	0.93310702	0.68086874	0.44259521
Q15393	SF3B3	98719000	41184000	50763000	0.41718411	0.51421714	0.81129956	0.00810933	0.00539751	0.29582736
Q15427	SF3B4	0	0	7804000	NaN	NaN	0	1	0.37390098	0.37390098
Q9BWJ5	SF3B5	10712000	4620700	4480700	0.43135735	0.4182879	1.03124511	0.37390098	0.37390098	1
P31947	SFN	96410000	156590000	142660000	1.62420917	1.47972202	1.09764481	0.14623863	0.23867539	0.70233685
P23246	SFPQ	386260000	110160000	201280000	0.28519651	0.52109981	0.5472973	0.1900733	0.51801854	0.03700437
O43556	SGCE	0	0	6825900	NaN	NaN	0	1	0.37390098	0.37390098
O43765	SGTA	105530000	43644000	96097000	0.4135696	0.91061312	0.45416611	1.77E-05	0.12739736	0.00020527
O75368	SH3BGRL	6768000	0	0	0	0	NaN	0.37390098	0.37390098	1
Q9UJC5	SH3BGRL2	0	0	2345300	NaN	NaN	0	1	0.37390098	0.37390098
Q9H299	SH3BGRL3	0	0	10802000	NaN	NaN	0	1	0.37390098	0.37390098
Q99962	SH3GL2	0	0	6992600	NaN	NaN	0	1	0.37390098	0.37390098
Q96B97	SH3KBP1	0	0	5417500	NaN	NaN	0	1	0.12224498	0.12224498
P34896	SHMT1	6062400	9677000	31391000	1.59623253	5.17798233	0.30827308	1	0.00015182	0.00015182
P34897	SHMT2	0	0	9903300	NaN	NaN	0	1	0.37390098	0.37390098
Q9HAT2	SIAE	0	892230016	565350016	NaN	NaN	1.57819045	0.00098067	1.03E-06	0.04891939
Q9H173	SIL1	0	141080000	58483000	NaN	NaN	2.41232491	1.18E-05	0.37390098	0.03714428
P42285	SKIV2L2	14416000	6500500	9621500	0.45092258	0.66741812	0.67562228	0.37390098	0.37390098	1
P63208	SKP1	28400000	13406000	24389000	0.47204226	0.85876763	0.54967403	1	0.37390098	0.37390098
P55011	SLC12A2	0	31322000	12813000	NaN	NaN	2.44454861	1	0.37390098	0.37390098
P53985	SLC16A1	6752500	8495800	4558400	1.25817108	0.6750685	1.86376798	0.12660731	1	0.12660731
Q15758	SLC1A5	44525000	34890000	18146000	0.78360474	0.40754631	1.92273784	0.9934355	0.37390098	0.37390098
Q00325	SLC25A3	0	5117800	0	NaN	NaN	NaN	0.37390098	1	0.37390098
P11166	SLC2A1	227110000	175070000	0	0.77085996	0	NaN	0.37390098	0.37390098	1
Q9ULF5	SLC39A10	22139000	438800000	373689984	19.8202267	16.8792629	1.17423534	0.00024835	0.00199903	0.98485637
Q15043	SLC39A14	0	9762000	0	NaN	NaN	NaN	0.37390098	1	0.37390098
P08195	SLC3A2	28191000	69839000	36367000	2.47735095	1.29002166	1.9203949	0.00393027	0.04371782	0.00875629

Q01650	SLC7A5	0	0	2008300	NaN	NaN	0	1	0.37390098	0.37390098
O14745	SLC9A3R1	70964000	48806000	44551000	0.68775719	0.62779719	1.09550858	0.02284466	0.00633133	0.48007548
Q9H2G2	SLK	5641300	12315000	84194000	2.18300748	14.9245739	0.14626934	1	1.19E-05	1.19E-05
P03973	SLPI	0	0	64346000	NaN	NaN	0	1	0.12007917	0.12007917
Q9NWH9	SLTM	5644900	0	0	0	0	NaN	0.37390098	0.37390098	1
Q14683	SMC1A	34441000	17166000	27647000	0.49841759	0.80273509	0.6208992	0.12294279	0.87463677	0.11733725
O95347	SMC2	0	0	974750	NaN	NaN	0	0.37390098	0.37390098	1
Q9UQE7	SMC3	5856700	3992400	7078200	0.6816808	1.20856452	0.56404173	1	0.37390098	0.37390098
Q9NTJ3	SMC4	3703600	0	0	0	0	NaN	0.65646863	0.33782414	1
Q6IN85	SMEK1	0	0	6413400	NaN	NaN	0	1	0.37390098	0.37390098
Q9H4F8	SMOC1	0	730110016	290550016	NaN	NaN	2.51285481	0.00010747	1.75E-05	0.00127525
Q92484	SMPDL3A	0	87805000	70016000	NaN	NaN	1.25407052	1	0.11840029	0.11840029
P52788	SMS	30456000	20420000	21489000	0.67047542	0.70557523	0.95025361	0.1577422	0.1577422	1
Q2TAY7	SMU1	4320000	3676700	4882100	0.85108799	1.13011575	0.75309807	0.37390098	0.54980087	0.1168203
Q16533	SNAPC1	1773500	0	0	0	0	NaN	0.97885287	0.81960237	1
Q7KZF4	SND1	336649984	151560000	373049984	0.45020053	1.10812414	0.40627265	0.01054401	0.1157656	0.00115665
O75643	SNRNP200	0	10206000	11879000	NaN	NaN	0.85916322	0.121654	0.11727814	0.85652852
P08621	SNRNP70	0	5755000	0	NaN	NaN	NaN	0.37390098	0.37390098	1
P09012	SNRPA	36187000	13207000	17102000	0.36496532	0.47260067	0.77224886	0.1161797	0.42071122	0.37390098
P09661	SNRPA1	73577000	0	48867000	0	0.66416138	0	0.12569463	0.39371973	0.37390098
P62314	SNRPD1	0	4844500	0	NaN	NaN	NaN	1	0.37390098	0.37390098
P62316	SNRPD2	64194000	45931000	57844000	0.71550298	0.90108109	0.7940495	0.16062436	0.48087367	0.12434819
P62318	SNRPD3	82340000	0	39353000	0	0.47793296	0	0.11633606	0.11633606	1
P62306	SNRPF	69943000	23572000	33286000	0.3370173	0.47590181	0.70816559	0.12873688	0.28167871	0.37390098
Q13596	SNX1	0	0	4957500	NaN	NaN	0	1	0.37390098	0.37390098
O60749	SNX2	21134000	18815000	32282000	0.8902716	1.52749121	0.58283252	1	0.11631464	0.11631464
O60493	SNX3	24346000	8921800	27855000	0.36645856	1.14413047	0.32029438	0.37390098	0.71375579	0.11653224
Q9Y5X3	SNX5	0	0	22142000	NaN	NaN	0	1	0.37390098	0.37390098
Q9UNH7	SNX6	0	5973500	9962400	NaN	NaN	0.59960449	1	0.00044	0.00044
P00441	SOD1	552390016	459270016	754510016	0.83142346	1.36590087	0.60869968	0.14944796	0.25876498	0.87205106
P04179	SOD2	9385100	13443000	22148000	1.43237686	2.35991096	0.60696226	1	0.37390098	0.37390098
P08294	SOD3	0	2872600064	1845900032	NaN	NaN	1.55620563	0.37390098	0.11681303	0.67467409
Q00796	SORD	18742000	0	0	0	0	NaN	0.1161783	0.1161783	1

Q99523	SORT1	0	12212000	15313000	NaN	NaN	0.79749233	1	0.12579116	0.12579116
O60271	SPAG9	5982000	6110900	4721500	1.02154803	0.78928453	1.29427087	1	0.11893032	0.11893032
O60575	SPINK4	0	152000000	0	NaN	NaN	NaN	0.16204691	1	0.44578516
O43278	SPINT1	55828000	317800000	317929984	5.6924839	5.6948123	0.99959117	1.22E-07	0.00013349	0.06068078
O43291	SPINT2	0	377340000	278520000	NaN	NaN	1.35480392	0.00281202	3.79E-05	0.0340057
Q9HCB6	SPON1	31535000	714289984	222800000	22.6507053	7.06516552	3.20596933	9.63E-06	3.05E-05	0.00013043
P35270	SPR	4728900	0	0	0	0	NaN	1	0.37390098	0.37390098
Q13813	SPTAN1	749390016	587129984	1236099968	0.78347719	1.64947486	0.47498584	0.12599073	0.00082854	0.00155114
Q01082	SPTBN1	228690000	203340000	418529984	0.88915128	1.83011925	0.48584333	0.62574393	0.00168503	0.00350867
P30626	SRI	23267000	21311000	31377000	0.91593242	1.34856236	0.67919177	1	0.37390098	0.37390098
P37108	SRP14	0	0	34153000	NaN	NaN	0	1	0.37390098	0.37390098
P49458	SRP9	12532000	4822400	11525000	0.3848069	0.91964573	0.41842949	0.1161864	0.24289136	0.37390098
Q9UH36	SRRD	45482000	0	0	0	0	NaN	0.10678542	0.91642189	1
Q9UQ35	SRRM2	7431700	8284100	6574600	1.11469781	0.88466972	1.26001585	0.11766137	1	0.11766137
Q9BXP5	SRRT	6614000	9953100	11894000	1.50485337	1.79830658	0.83681691	1	0.37390098	0.37390098
Q07955	SRSF1	34917000	27919000	27235000	0.79958189	0.77999258	1.02511477	0.25565255	0.171239	0.45088264
Q16629	SRSF7	0	0	1825100	NaN	NaN	0	1	0.37390098	0.37390098
Q13242	SRSF9	3929400	0	4296900	0	1.09352577	0	1	0.12137386	0.12137386
P05455	SSB	73092000	33606000	96795000	0.45977673	1.32428992	0.34718734	0.00083321	0.06848014	0.00714845
Q08945	SSRP1	10532000	21272000	22636000	2.0197494	2.14925933	0.93974203	1	0.37390098	0.37390098
Q9NP77	SSU72	0	0	2565300	NaN	NaN	0	1	0.37390098	0.37390098
P50502	ST13	100210000	81350000	199190000	0.81179523	1.98772573	0.40840402	0.01543409	0.00093033	0.00059301
Q9Y5Y6	ST14	0	37985000	35393000	NaN	NaN	1.0732348	1	0.00017	0.00017
Q11206	ST3GAL4	11471000	23432000	50236000	2.0427165	4.37939167	0.46643841	0.37390097	0.11681802	0.77327436
P15907	ST6GAL1	1251400	0	0	0	0	NaN	1	0.37390098	0.37390098
O75886	STAM2	0	0	10984000	NaN	NaN	0	1	0.37390098	0.37390098
O76061	STC2	112690000	0	0	0	0	NaN	0.00143493	0.00143493	1
P31948	STIP1	1133900032	497230016	1069699968	0.4385131	0.94338119	0.46483129	0.00252541	0.34127527	0.00128388
Q9Y6E0	STK24	5063800	3662300	11765000	0.72323155	2.32335401	0.31128773	1	0.37390098	0.37390098
P16949	STMN1	96839000	42490000	150130000	0.43876949	1.55030513	0.28302139	0.00266676	0.01553552	0.00082638
Q93045	STMN2	0	0	70477000	NaN	NaN	0	1	0.37390098	0.37390098
Q9Y3F4	STRAP	320929984	120250000	242120000	0.37469232	0.75443244	0.49665454	0.05833052	0.62031025	0.03025807
Q13033	STRN3	6989000	6844800	7527600	0.97936755	1.07706392	0.90929377	1	0.37390098	0.37390098

O15400	STX7	15072000	0	22379000	0	1.4848063	0	1	0.37390098	0.37390098
P53999	SUB1	35995000	8361100	13851000	0.23228504	0.38480344	0.60364592	0.11611814	0.11611814	1
Q9Y2Z0	SUGT1	126170000	38725000	123030000	0.30692717	0.97511292	0.31476063	3.46E-05	0.08440576	4.14E-05
Q8IWU5	SULF2	0	102910000	95372000	NaN	NaN	1.0790379	1.65E-05	5.48E-05	0.01980491
Q8NBK3	SUMF1	0	8280800	0	NaN	NaN	NaN	0.11642519	1	0.11642519
P63165	SUMO1	106780000	42411000	79066000	0.39718112	0.740457	0.53639996	0.20169342	0.77583784	0.23240642
Q6EEV6	SUMO4	0	82313000	0	NaN	NaN	NaN	1	0.37390098	0.37390098
Q9Y5B9	SUPT16H	12079000	16234000	14817000	1.34398544	1.22667444	1.09563339	0.37390098	0.11869231	0.37121832
O00267	SUPT5H	0	0	2885400	NaN	NaN	0	1	0.37390098	0.37390098
Q9UH65	SWAP70	6724300	3220100	16728000	0.47887513	2.48769379	0.19249761	1	0.11620655	0.11620655
Q15431	SYCP1	0	3504800	0	NaN	NaN	NaN	0.37390098	1	0.37390098
O60506	SYNCRIP	606569984	116660000	186070000	0.19232735	0.30675769	0.62696832	0.00488036	0.02192809	0.36551541
Q8NF91	SYNE1	406400000	0	0	0	0	NaN	1	0.37390098	0.37390098
Q01995	TAGLN	3291000	0	0	0	0	NaN	0.37390098	0.37390098	1
P37802	TAGLN2	289270016	225520000	481600000	0.77961761	1.66488051	0.46827242	0.05775152	0.09731507	0.02231721
P37837	TALDO1	447649984	315670016	393860000	0.70517153	0.87983918	0.80147773	0.00375112	0.64603728	0.00402701
P26639	TARS	407460000	143360000	256970000	0.35183823	0.63066316	0.55788612	0.00040791	0.04594642	0.0005084
Q6P1N9	TATDN1	12910000	0	22598000	0	1.75042605	0	1	0.11654283	0.11654283
O14907	TAX1BP3	0	0	2208500	NaN	NaN	0	1	0.37390098	0.37390098
O75347	TBCA	250350000	116050000	208930000	0.46355101	0.83455163	0.55544919	0.08477448	0.49079853	0.09360715
Q99426	TBCB	11878000	9380100	41868000	0.78970367	3.52483582	0.22403984	1	0.37390098	0.37390098
Q9BZK7	TBL1XR1	31752000	20849000	24707000	0.65662003	0.77812421	0.8438499	0.37390098	0.89517564	0.37390098
P23193	TCEA1	49518000	40151000	100810000	0.81083643	2.03582525	0.3982839	0.37390098	0.02242393	0.00143096
Q15369	TCEB1	33343000	32318000	48310000	0.9692589	1.44887984	0.66897124	1	0.37390098	0.37390098
Q15370	TCEB2	27696000	17141000	33838000	0.61889803	1.22176492	0.50656068	0.86564505	0.01627091	0.01091479
P17987	TCP1	687929984	241840000	584910016	0.35154739	0.85024643	0.41346532	0.00080938	0.34297198	0.00068317
Q9UGI8	TES	13553000	9652700	7984800	0.7122187	0.58915371	1.20888436	0.37390098	0.37390098	1
P02787	TF	0	7311000	0	NaN	NaN	NaN	0.37390098	1	0.37390098
P04155	TFF1	16468000	840510016	88369000	51.0389862	5.36610413	9.51136684	0.00339892	0.00022056	0.00897391
Q03403	TFF2	0	1353699968	78289000	NaN	NaN	17.2910614	0.00059498	1	0.00059498
Q07654	TFF3	93156000	2404999936	299390016	25.8169079	3.21385646	8.03299999	2.88E-05	0.0023683	4.59E-05
Q92734	TFG	65402000	59553000	57509000	0.91056848	0.87931561	1.03554225	1	0.37390098	0.37390098
P02786	TFRC	255580000	1023299968	946320000	4.00383425	3.7026372	1.08134663	0.01353259	0.00017363	0.21925573

P01137	TGFB1	30233000	34087000	0	1.12747657	0	NaN	0.37390098	1	0.37390098
Q15582	TGFBI	522950016	3408900096	674070016	6.51859617	1.28897595	5.05718994	0.00050198	0.0028884	0.00066876
Q08188	TGM3	1561900	0	0	0	0	NaN	0.45444727	0.90442324	1
O43493	TGOLN2	0	5641100	4391000	NaN	NaN	1.28469598	0.37390098	0.00088046	0.13069843
P35442	THBS2	137790000	1.0686E+10	2945900032	77.5527954	21.3796368	3.62741446	0.00596291	3.21E-06	0.0149905
P52888	THOP1	28739000	14939000	22337000	0.51981628	0.77723652	0.66880065	0.11611986	0.40686008	0.37390098
Q9BV44	THUMPD3	0	0	1251500	NaN	NaN	0	1	0.37390098	0.37390098
Q01085	TIAL1	27690000	16174000	27042000	0.58410978	0.97659802	0.59810662	0.37390098	0.53282523	0.12712418
O60220	TIMM8A	0	0	7813400	NaN	NaN	0	1	0.12242359	0.33365434
P01033	TIMP1	1207100032	5221300224	1912000000	4.32549095	1.58396149	2.73080564	0.00119231	0.11383477	0.00166388
P16035	TIMP2	304769984	19483000	14778000	0.0639269	0.04848903	1.31837869	0.00010558	0.00010558	1
Q99727	TIMP4	0	0	8705600	NaN	NaN	0	1	0.37390098	0.37390098
Q9GZM7	TINAGL1	0	934270016	471369984	NaN	NaN	1.98203123	6.64E-07	3.26E-06	4.15E-05
P29401	TKT	731590016	563980032	757449984	0.77089632	1.03534758	0.74457723	0.06321426	0.21954474	0.03796056
Q9Y490	TLN1	100200000	65061000	139090000	0.64931136	1.38812375	0.46776187	0.0105772	0.00163826	0.00023478
P82094	TMF1	0	0	5696799744	NaN	NaN	0	1	0.37390098	0.37390098
Q9NYL9	TMOD3	8955400	8334800	7624400	0.93070102	0.85137457	1.09317458	1	0.37390098	0.37390098
P42166	TMPO	40953000	31964000	40869000	0.78050447	0.99794888	0.78210866	0.68080229	0.34390044	0.08381534
P63313	TMSB10	214940000	0	119400000	0	0.55550385	0	0.0022525	0.06634837	0.00150438
P62328	TMSB4X	3899399936	283960000	890129984	0.07282146	0.22827359	0.3190096	0.00465504	0.0155144	0.00118615
P24821	TNC	2010800000	198640000	317560000	0.09878656	0.1579272	0.62551957	0.00036127	0.00039009	0.17006332
O00300	TNFRSF11B	2605500	12099000	5375700	4.64363861	2.06321239	2.25068355	1.42E-05	1	1.42E-05
Q9C0C2	TNKS1BP1	8730100	0	0	0	0	NaN	0.37390098	0.37390098	1
Q92973	TNPO1	13383000	11255000	20433000	0.84099233	1.52678776	0.55082464	0.00090285	0.68435156	5.45E-05
O75674	TOM1L1	0	17843000	5355400	NaN	NaN	3.33177733	0.11807915	1	0.11807915
P11387	TOP1	14781000	4662500	6618900	0.31543875	0.44779783	0.70442218	1	0.37390098	0.37390098
O14657	TOR1B	0	22891000	24531000	NaN	NaN	0.93314582	4.00E-05	0.1164	0.23025
Q9H497	TOR3A	0	27269000	17262000	NaN	NaN	1.57971263	1	0.37390098	0.37390098
Q53FA7	TP53I3	0	0	8799500	NaN	NaN	0	1	0.37390098	0.37390098
P55327	TPD52	9085300	8919300	25093000	0.98172873	2.76193404	0.35544974	1	0.11742619	0.11742619
O43399	TPD52L2	54313000	30373000	51933000	0.55922157	0.95617992	0.58484972	2.82E-06	0.44542131	0.00041332
P60174	TPI1	3605100032	1801200000	2451899904	0.49962553	0.68011981	0.73461401	0.00364942	0.00395646	0.03008654
P06753	TPM3	434689984	131350000	175250000	0.30216938	0.4031609	0.74950069	0.00656874	0.0114571	0.22834367

P67936	TPM4	271129984	106360000	228150000	0.39228415	0.84147829	0.46618453	0.02657738	0.11427665	0.02076739
O14773	TPP1	32709000	267280000	214710000	8.17145157	6.56424856	1.24484193	0.00321777	0.00086635	0.06730745
P29144	TPP2	41423000	11129000	17277000	0.26866716	0.41708714	0.64415121	4.02E-05	0.02539811	0.37390098
P12270	TPR	26501000	27218000	23702000	1.02705562	0.89438134	1.14834189	0.37390098	0.37390098	1
P13693	TPT1	243560000	81306000	126710000	0.33382329	0.52024144	0.64166993	0.00048577	0.12460868	0.98779154
Q12931	TRAP1	11097000	16692000	6457500	1.50419033	0.58191401	2.58490133	0.8132593	0.34874997	0.6469543
Q13263	TRIM28	110960000	25451000	38052000	0.22937094	0.3429344	0.66884792	0.00036275	0.00036275	1
Q9UI30	TRMT112	0	0	2874600	NaN	NaN	0	1	0.37390098	0.37390098
Q9UJA5	TRMT6	12699000	0	0	0	0	NaN	1	0.37390098	0.37390098
Q96FX7	TRMT61A	0	0	0	NaN	NaN	NaN	1	1	1
Q99816	TSG101	25206000	44649000	35543000	1.77136397	1.41010082	1.25619674	0.37390098	1	0.37390098
Q15631	TSN	104240000	38243000	45241000	0.36687452	0.43400806	0.8453173	0.06554432	0.18847847	0.19103213
Q99598	TSNAX	29957000	17114000	18593000	0.57128549	0.62065625	0.92045391	0.37390098	0.37390098	1
O43657	TSPAN6	18290000	121380000	56504000	6.63641357	3.08933854	2.14816642	0.11634397	0.11664874	0.42904827
P19075	TSPAN8	0	61556000	0	NaN	NaN	NaN	0.37390098	1	0.37390098
Q13630	TSTA3	0	0	27043000	NaN	NaN	0	1	0.37390098	0.37390098
Q99614	TTC1	9140200	5917200	7835400	0.6473819	0.85724604	0.75518799	0.11695235	0.44322678	0.37390098
Q96AE7	TTC17	0	27979000	0	NaN	NaN	NaN	0.37390098	1	0.37390098
Q14166	TTLL12	10317000	8860000	11702000	0.85877675	1.13424444	0.75713551	1	0.11630639	0.11630639
Q6EMB2	TTLL5	38498000	13948000	23868000	0.36230454	0.61998028	0.58438075	0.86791593	0.9735868	0.24644426
P68366	TUBA4A	0	0	8490900	NaN	NaN	0	1	0.37390098	0.37390098
P07437	TUBB	85542000	32126000	50268000	0.3755582	0.58764118	0.63909447	0.37390098	0.37390098	1
P68371	TUBB4B	253390000	180290000	388480000	0.71151191	1.53313076	0.46409082	0.00409121	0.00523458	0.00154325
Q12792	TWF1	65209000	49228000	91869000	0.75492644	1.40883923	0.53584999	5.28E-05	0.00050192	0.02121736
Q6IBS0	TWF2	20167000	10119000	17303000	0.5017603	0.85798579	0.58481187	0.37390098	0.64511049	0.12444281
P40222	TXLNA	31635000	25579000	22733000	0.80856645	0.71860284	1.1251924	0.81437522	0.39650393	0.19567747
P10599	TXN	439760000	425380000	722609984	0.96730036	1.6431917	0.58867162	0.27380183	0.03825794	0.07823176
O95881	TXNDC12	0	47750000	8194100	NaN	NaN	5.82736349	0.37390098	1	0.37390098
Q9P2K2	TXNDC16	0	3687000	1438400	NaN	NaN	2.56326485	0.37390098	1	0.37390098
Q9BRA2	TXNDC17	305089984	133520000	153730000	0.43764138	0.50388414	0.86853576	0.17601806	0.23799464	0.839279
Q8NBS9	TXNDC5	99727000	629030016	129260000	6.30751991	1.29613841	4.86639357	0.00020286	0.00320999	0.00031578
O43396	TXNL1	5281000	6230800	7446000	1.17985225	1.40996027	0.83679831	1	0.1189566	0.1189566
P83876	TXNL4A	0	0	1045300	NaN	NaN	0	1	0.37390098	0.37390098

Q16881	TXNRD1	31115000	24258000	35528000	0.77962399	1.14182866	0.68278539	1	0.00144245	0.00144245
Q3KQV9	UAP1L1	0	0	911930	NaN	NaN	0	1	0.37390098	0.37390098
P22314	UBA1	404230016	210130000	342830016	0.51982778	0.84810627	0.61292768	0.00030467	0.00825176	0.00043437
Q9UBT2	UBA2	86239000	19675000	47305000	0.22814503	0.54853374	0.41591799	0.00284482	0.12660679	0.14782836
Q8TBC4	UBA3	5192200	2945400	9708200	0.56727397	1.86976624	0.30339301	0.37390098	0.37390098	1
P62987	UBA52	324520000	586819968	454630016	1.80827057	1.40093064	1.29076385	0.04715862	0.02706868	0.71377587
A0AVT1	UBA6	15386000	0	23808000	0	1.54738069	0	1	0.37390098	0.37390098
Q14157	UBAP2L	0	0	1580100	NaN	NaN	0	1	0.37390098	0.37390098
P63279	UBE2I	107210000	24554000	30109000	0.22902714	0.28084135	0.81550366	2.15E-07	0.0019839	0.1170997
P61086	UBE2K	26620000	13632000	17545000	0.51209617	0.65909094	0.77697349	0.99068648	0.95474082	0.91654921
P68036	UBE2L3	39472000	15168000	30342000	0.3842724	0.76869678	0.49990112	0.00085581	0.24772064	0.00126745
P61081	UBE2M	59296000	33522000	74701000	0.56533325	1.25979829	0.44874901	0.15974031	0.00641656	0.01669785
Q5JXB2	UBE2NL	1434499968	1267600000	1754599936	0.88365287	1.22314394	0.72244388	0.29332143	0.09233025	0.06834376
Q9NPD8	UBE2T	10182000	10340000	16369000	1.01551759	1.60764098	0.63168186	1	0.37390098	0.37390098
Q13404	UBE2V1	0	0	6915600	NaN	NaN	0	1	0.37390098	0.37390098
Q15819	UBE2V2	115700000	66913000	100130000	0.57833189	0.86542785	0.66826129	0.00417832	0.09570122	0.01929634
O14562	UBFD1	25068000	4976400	4449400	0.19851604	0.17749321	1.11844289	1	0.37390098	0.37390098
Q8WVY7	UBLCP1	21879000	8460200	12715000	0.38668129	0.58115089	0.6653716	0.12908374	0.9521963	4.68E-05
Q9UMX0	UBQLN1	21391000	0	0	0	0	NaN	1	0.37390098	0.37390098
Q9NRR5	UBQLN4	9175100	6560800	6917600	0.71506578	0.75395364	0.94842142	0.19126017	0.98264337	0.71509492
O95071	UBR5	0	0	1299100	NaN	NaN	0	1	0.37390098	0.37390098
O94888	UBXN7	4847200	0	0	0	0	NaN	1	0.37390098	0.37390098
P15374	UCHL3	81158000	13501000	48518000	0.16635452	0.59782153	0.27826786	0.13088848	0.13088848	1
Q9Y5K5	UCHL5	0	0	6021400	NaN	NaN	0	1	0.37390098	0.37390098
Q92890	UFD1L	6821900	0	0	0	0	NaN	0.32083964	0.97020215	1
O60701	UGDH	58483000	46472000	89372000	0.79462409	1.52817059	0.51998389	0.75729316	0.4858492	0.36880028
Q9NYU2	UGGT1	0	219090000	30012000	NaN	NaN	7.30007982	0.00205008	1	0.00205008
Q16851	UGP2	36210000	14643000	26169000	0.40439105	0.72270089	0.55955517	0.00180192	0.04662802	0.37390098
Q9BZM5	ULBP2	14901000	157210000	321969984	10.5502987	21.6072731	0.48827532	0.15836425	0.00049703	0.28327537
Q9BZM4	ULBP3	0	22660000	23258000	NaN	NaN	0.9742884	1	0.37390098	0.37390098
P11172	UMPS	8310500	5114500	0	0.61542624	0	NaN	0.37390098	0.37390098	1
Q9H3U1	UNC45A	3408000	3558900	6877400	1.04427814	2.01801634	0.51747751	1	0.37390098	0.37390098
Q92900	UPF1	0	0	4853200	NaN	NaN	0	0.37390098	0.37390098	1

Q16831	UPP1	5073000	0	2669000	0	0.5261187	0	1	0.37390098	0.37390098
P06132	UROD	16456000	7487800	6809500	0.45501944	0.41380045	1.09961081	0.37390098	0.37390098	1
O60763	USO1	26335000	36290000	30265000	1.37801409	1.14923108	1.19907486	0.13920678	0.78814977	0.11631161
P54578	USP14	24139000	7948000	14126000	0.32925969	0.58519411	0.56265044	0.11619032	0.3516964	0.37390098
Q9P275	USP36	25207000	0	0	0	0	NaN	0.60969496	0.31093654	1
P45974	USP5	87622000	33054000	63499000	0.37723404	0.7246924	0.52054363	0.13626556	0.66942316	0.13240209
Q93008	USP9X	0	0	5695400	NaN	NaN	0	1	0.22591653	0.34008929
Q8NBZ7	UXS1	0	0	28717000	NaN	NaN	0	1	0.37390098	0.37390098
P26640	VARS	20961000	19510000	36616000	0.93077618	1.74686325	0.5328272	1	0.37390098	0.37390098
Q6EMK4	VASN	18866000	7146200	0	0.37878725	0	NaN	0.37390098	0.37390098	1
Q99536	VAT1	43137000	32985000	85599000	0.76465678	1.98435223	0.38534328	1	0.37390098	0.37390098
P61758	VBP1	8987900	7054500	11545000	0.78488857	1.28450477	0.61104375	1	0.37390098	0.37390098
P18206	VCL	610240000	504700000	865260032	0.82705164	1.41790116	0.58329284	0.05890666	0.00148216	0.00355126
P55072	VCP	527929984	204340000	454510016	0.38705891	0.8609286	0.44958305	0.00110161	0.59535253	0.00118112
P15692	VEGFA	16453000	98876000	102730000	6.00960302	6.24384594	0.96248418	0.11641381	0.00143376	0.23583223
O15240	VGF	0	50050000	216110000	NaN	NaN	0.23159502	1	3.00E-05	3.00E-05
P09327	VIL1	114310000	27172000	50536000	0.23770449	0.44209605	0.5376761	5.44E-05	0.00032142	4.62E-07
P08670	VIM	379060000	44037000	85002000	0.11617422	0.22424418	0.51807016	0.00114505	0.00173534	0.01313016
O95497	VNN1	0	0	48037000	NaN	NaN	0	1	0.37390098	0.37390098
Q9BRG1	VPS25	0	0	2457700	NaN	NaN	0	1	0.07937776	0.08651212
O75436	VPS26A	43081000	28484000	40801000	0.66117316	0.94707644	0.69812012	0.1176517	0.31199679	0.00010527
Q4G0F5	VPS26B	15964000	3827300	0	0.23974568	0	NaN	1	0.37390098	0.37390098
Q9UBQ0	VPS29	21760000	29175000	71193000	1.34076285	3.2717371	0.40980151	1	0.1161229	0.1161229
Q96QK1	VPS35	108860000	95757000	166550000	0.87963438	1.52994668	0.57494444	1	0.00089316	0.00089316
P62760	VSNL1	13308000	0	0	0	0	NaN	0.37390098	0.37390098	1
Q9NP79	VTA1	16666000	9298200	13846000	0.55791432	0.83079326	0.67154413	0.12327582	0.93830425	0.12666601
Q5GFL6	VWA2	8100300	0	0	0	0	NaN	0.37390098	0.37390098	1
P23381	WARS	60027000	35114000	52825000	0.58497012	0.88002068	0.66472316	0.04993789	0.8999759	0.08399446
O75083	WDR1	51061000	37735000	83429000	0.73901802	1.63390851	0.45230076	0.86123562	0.31848422	0.2495286
P61964	WDR5	0	0	4982900	NaN	NaN	0	0.37390098	0.37390098	1
Q9BRP8	WIBG	0	0	2498700	NaN	NaN	0	1	0.37390098	0.37390098
O00308	WWP2	0	0	1936900	NaN	NaN	0	1	0.37390098	0.37390098
Q9NQW7	XPNPEP1	0	0	3108200	NaN	NaN	0	1	0.37390098	0.37390098

O14980	XPO1	27328000	19823000	40088000	0.72537327	1.46692038	0.49448714	1	0.37390098	0.37390098
Q9C0E2	XPO4	0	0	3020300	NaN	NaN	0	1	0.37390098	0.37390098
P13010	XRCC5	177440000	69617000	106910000	0.39234108	0.60251355	0.65117389	0.00527515	0.0167196	0.03203987
P12956	XRCC6	499710016	167170000	242160000	0.33453402	0.48460105	0.69032872	0.00023573	0.00058907	0.00186041
Q9H0D6	XRN2	33901000	0	32746000	0	0.96593022	0	0.11896923	0.11896923	1
P54577	YARS	166580000	50141000	98719000	0.30100253	0.59262216	0.50791639	0.00226491	0.0139296	0.01102586
P67809	YBX1	51707000	25187000	49207000	0.48711005	0.95165062	0.51185805	0.22862999	0.94033515	0.23881646
O15498	YKT6	12751000	0	1959000	0	0.15363501	0	0.77777487	0.95618677	0.37551278
Q9Y5A9	YTHDF2	4583500	5503700	7947300	1.20076358	1.73389328	0.69252449	1	0.13150106	0.13150106
P31946	YWHAB	478340000	350929984	597560000	0.73364133	1.24923694	0.58727157	0.9145025	0.00660263	0.00296079
P62258	YWHAE	2363899904	736499968	1327500032	0.31156141	0.56157202	0.55480224	0.01006957	0.40268877	0.01741111
P61981	YWHAG	427060000	282369984	496110016	0.6611951	1.1616869	0.56916809	0.06730789	0.10903398	0.02313374
Q04917	YWHAH	192540000	112190000	269420000	0.5826841	1.39929366	0.41641304	0.03384934	0.00459619	9.85E-05
P27348	YWHAQ	169470000	87188000	164680000	0.51447451	0.97173542	0.52943891	0.01528157	0.9279741	0.00408124
P63104	YWHAZ	1068499968	854300032	2239399936	0.79953212	2.09583521	0.38148615	0.83273178	0.00163202	0.00098444
Q8WU90	ZC3H15	0	0	2882700	NaN	NaN	0	1	0.19051452	0.13968961
O43670	ZNF207	42146000	12914000	17359000	0.30641106	0.41187775	0.74393684	0.37390098	0.37390098	1
O75312	ZNF259	24051000	0	12940000	0	0.53802335	0	0.37390098	0.37390098	1
A6NL28		0	0	18335000	NaN	NaN	0	1	0.58861411	0.45952225
Q0VD67		0	12557000	0	NaN	NaN	NaN	0.14817709	1	0.79239231
Q6ZN80		0	0	3128100	NaN	NaN	0	1	0.40733621	0.65222967
Q6ZS92		42348000	0	0	0	0	NaN	0.37390098	1	0.37390098
