# Supplementary material for: A prognostic six‐gene expression risk‐score derived from proteomic profiling of the metastatic colorectal cancer secretome
Source: J Pathol Clin Res. 2022 Sep 22;8(6):495–508. doi: 10.1002/cjp2.294 (PMC9535096; doi:10.1002/cjp2.294)
Supplement: Supplementary file 4 — Table S2. Up‐ and down‐regulated proteins in KM12L4 and/or KM12SM compared with SW620 [file CJP2-8-495-s009.pdf]

A prognostic six-gene expression risk-score derived from proteomic profiling of the metastatic colorectal cancer secretome

J Robles *et al.* *J Pathol Clin Res* DOI: https://doi.org/10.1002/cjp2.294

Table S2. Up and down-regulated proteins in KM12L4 and/or KM12SM compared with SW620.

Protein IDs	Protein name	Gene names	KM12L4 vs SW620		KM12SM vs SW620		KM12L4 vs KM12SM	
			Ratio	p-value	Ratio	p-value	Ratio	p-value
Q9BYZ8	Regenerating islet-derived protein 4	REG4	1316.64	0.00517	369.51	0.03005	3.56	0.12218
P07942	Laminin subunit beta-1	LAMB1	180.28	0.00439	43.26	0.00008	4.17	0.02474
Q9H3G5	Probable serine carboxypeptidase CPVL	CPVL	65.36	0.00003	58.46	0.00009	1.12	0.00733
Q06481	Amyloid-like protein 2	APLP2	52.94	0.01917	50.73	0.00003	1.04	0.39511
P12830	Cadherin-1	CDH1	52.11	0.00100	46.70	0.00008	1.12	0.69348
P07686	Beta-hexosaminidase subunit beta	HEXB	56.04	0.00137	41.41	0.00002	1.35	0.05651
Q6YHK3	CD109 antigen	CD109	44.88	0.00015	45.97	0.00012	0.98	0.25060
P61626	Lysozyme C	LYZ	96.78	0.00001	17.66	0.11956	5.48	0.00048
P35442	Thrombospondin-2	THBS2	77.55	0.00596	21.38	0.00000	3.63	0.01499
P11047	Laminin subunit gamma-1	LAMC1	61.87	0.00002	24.03	0.00013	2.57	0.00039
P61916	Epididymal secretory protein E1	NPC2	36.37	0.00003	40.66	0.00032	0.89	0.17536
O43852	Calumenin	CALU	44.32	0.10604	21.15	0.03587	2.10	0.82576
P38571	Lysosomal acid lipase/cholesteryl ester hydrolase	LIPA	35.82	0.37390	23.62	0.00017	1.52	0.32628
O00115	Deoxyribonuclease-2-alpha	DNASE2	27.16	0.11648	29.70	0.00000	0.91	0.34158
Q02809	Procollagen-lysine,2-oxoglutarate 5-dioxygenase 1	PLOD1	33.56	0.00030	21.80	0.00021	1.54	0.07560
Q8NBP7	Proprotein convertase subtilisin/kexin type 9	PCSK9	37.17	0.00113	18.60	0.00465	2.00	0.01279
A8K2U0	Alpha-2-macroglobulin-like protein 1	A2ML1	31.58	0.00082	21.22	0.00056	1.49	0.06829
Q8NHP8	Putative phospholipase B-like 2	PLBD2	27.56	0.04952	23.21	0.00016	1.19	0.73339
Q13510	Acid ceramidase	ASAH1	26.57	0.00007	23.03	0.00012	1.15	0.03763
P10909	Clusterin	CLU	18.85	0.00024	24.86	0.00003	0.76	0.01768
P29279	Connective tissue growth factor	CTGF	19.98	0.01617	21.67	0.00032	0.92	0.68979
P27797	Calreticulin	CALR	61.39	0.00002	6.55	0.00366	9.37	0.00003
Q9ULF5	Zinc transporter ZIP10	SLC39A10	19.82	0.00025	16.88	0.00200	1.17	0.98486
P15586	N-acetylglucosamine-6-sulfatase	GNS	17.48	0.00003	18.50	0.00004	0.94	0.00170
P61769	Beta-2-microglobulin	B2M	21.59	0.01187	13.47	0.14492	1.60	0.27077
P17936	Insulin-like growth factor-binding protein 3	IGFBP3	23.06	0.00393	11.99	0.00005	1.92	0.04750
Q02818	Nucleobindin-1	NUCB1	15.83	0.00149	17.37	0.02007	0.91	0.22766
P04155	Trefoil factor 1	TFF1	51.04	0.00340	5.37	0.00022	9.51	0.00897
Q86X29	Lipolysis-stimulated lipoprotein receptor	LSR	18.88	0.00000	14.16	0.00034	1.33	0.00257
Q9BZM5	NKG2D ligand 2	ULBP2	10.55	0.15836	21.61	0.00050	0.49	0.28328
Q99538	Legumain	LGMN	17.97	0.00505	12.36	0.01376	1.45	0.19867
P07858	Cathepsin B	CTSB	13.63	0.00038	14.31	0.06843	0.95	0.21650
Q16787	Laminin subunit alpha-3	LAMA3	19.44	0.00016	9.39	0.00003	2.07	0.00072
Q9HCB6	Spondin-1	SPON1	22.65	0.00001	7.07	0.00003	3.21	0.00013
P10619	Lysosomal protective protein	CTSA	16.33	0.00566	9.19	0.00736	1.78	0.15672
Q10472	Polypeptide N-acetylgalactosaminyltransferase 1	GALNT1	28.21	0.00919	5.18	0.00114	5.44	0.09406

Q14118	Alpha-dystroglycan	DAG1	10.97	0.00501	13.02	0.12115	0.84	0.32492
P53634	Dipeptidyl peptidase 1	CTSC	13.42	0.00074	10.07	0.01114	1.33	0.05048
Q96HE7	ERO1-like protein alpha	ERO1L	16.39	0.000513	1.35	0.116273	12.17	0.001101
O94985	Calsyntenin-1	CLSTN1	15.44	0.00001	8.73	0.00025	1.77	0.00058
P14625	Endoplasmin	HSP90B1	52.97	0.01434	2.44	0.00002	21.75	0.01649
P07602	Prosaposin	PSAP	13.01	0.00006	9.57	0.00793	1.36	0.03593
P16070	CD44 antigen	CD44	33.79	0.00000	3.62	0.13055	9.34	0.00447
P50897	Palmitoyl-protein thioesterase 1	PPT1	14.40	0.00760	8.17	0.00369	1.76	0.77400
P07339	Cathepsin D	CTSD	12.37	0.00106	8.82	0.00644	1.40	0.17399
P25815	Protein S100-P	S100P	7.36	1.00000	14.51	0.11724	0.51	0.11724
P30040	Endoplasmic reticulum resident protein 29	ERP29	28.11	0.07085	3.71	0.00105	7.58	0.10953
Q969H8	Myeloid-derived growth factor	C19orf10	25.90	0.01245	4.00	0.37390	6.48	0.02512
Q9BS26	Endoplasmic reticulum resident protein 44	ERP44	14.94	0.00301	6.58	0.00111	2.27	0.01871
Q14697	Neutral alpha-glucosidase AB	GANAB	19.49	0.00968	4.60	0.00065	4.23	0.03915
O15230	Laminin subunit alpha-5	LAMA5	25.74	0.00018	3.37	0.00000	7.63	0.00049
Q07654	Trefoil factor 3	TFF3	25.82	0.00003	3.21	0.00237	8.03	0.00005
P06280	Alpha-galactosidase A	GLA	9.15	0.00048	8.90	0.01080	1.03	0.88587
P13667	Protein disulfide-isomerase A4	PDIA4	46.22	0.00884	1.73	0.00011	26.74	0.00981
P14314	Glucosidase 2 subunit beta	PRKCSH	23.33	0.00006	3.36	0.00018	6.95	0.00011
Q14126	Desmoglein-2	DSG2	7.75	0.00000	9.20	0.00002	0.84	0.00556
P01130	Low-density lipoprotein receptor	LDLR	6.98	0.00009	9.98	0.00504	0.70	0.12044
P21926	CD9 antigen	CD9	9.93	0.00151	6.54	0.16933	1.52	0.06820
P10253	70 kDa lysosomal alpha-glucosidase	GAA	6.63	0.02445	9.06	0.00057	0.73	0.42325
P18065	Insulin-like growth factor-binding protein 2	IGFBP2	5.37	0.00588	10.95	0.00010	0.49	0.00666
Q13162	Peroxiredoxin-4	PRDX4	23.55	0.00323	2.48	0.18695	9.50	0.00464
P16422	Epithelial cell adhesion molecule	EPCAM	10.48	0.00026	5.53	0.11623	1.89	0.01278
Q9BTY2	Plasma alpha-L-fucosidase	FUCA2	8.54	0.01822	6.35	0.00633	1.34	0.14237
O14773	Tripeptidyl-peptidase 1	TPP1	8.17	0.00322	6.56	0.00087	1.24	0.06731
P55145	Mesencephalic astrocyte-derived neurotrophic factor	MANF	22.33	0.00540	2.10	0.07960	10.64	0.00742
P07225	Vitamin K-dependent protein S	PROS1	7.30	0.37390	6.23	0.00026	1.17	0.11389
P28799	Acrogranin	GRN	7.19	0.00021	6.02	0.00086	1.20	0.34789
P11021	78 kDa glucose-regulated protein	HSPA5	23.11	0.04317	1.78	0.12221	13.01	0.04665
P98160	Basement membrane-specific heparan sulfate proteoglycan c	HSPG2	14.07	0.00331	2.70	0.01227	5.20	0.00410
P15692	Vascular endothelial growth factor A	VEGFA	6.01	0.11641	6.24	0.00143	0.96	0.23583
P06865	Beta-hexosaminidase subunit alpha	HEXA	7.78	0.00054	4.81	0.00008	1.62	0.01077
Q14696	LDLR chaperone MESD	MESDC2	21.50	0.00055	1.69	1.00000	12.72	0.00055
Q9BRK5	45 kDa calcium-binding protein	SDF4	9.70	0.00464	3.69	0.01073	2.63	0.01289
P30101	Protein disulfide-isomerase A3	PDIA3	17.56	0.00000	2.00	0.00291	8.76	0.00000
Q9H2G2	STE20-like serine/threonine-protein kinase	SLK	2.18	1.00000	14.92	0.00001	0.15	0.00001

O43278	Kunitz-type protease inhibitor 1	SPINT1	5.69	0.00000	5.69	0.00013	1.00	0.06068
O43175	D-3-phosphoglycerate dehydrogenase	PHGDH	4.83	0.00003	6.42	0.00001	0.75	0.00274
O75874	Isocitrate dehydrogenase [NADP] cytoplasmic	IDH1	5.03	0.12462	5.86	0.00001	0.86	0.04382
O14672	Disintegrin and metalloproteinase domain-containing protein	ADAM10	7.98	0.00005	3.61	0.00005	2.21	0.02862
Q99519	Sialidase-1	NEU1	5.38	0.00115	5.06	0.00007	1.06	0.11012
Q9P2B2	Prostaglandin F2 receptor negative regulator	PTGFRN	9.33	0.00000	2.75	0.12302	3.40	0.00134
P39687	Acidic leucine-rich nuclear phosphoprotein 32 family member	ANP32A	4.56	0.00555	5.57	0.00113	0.82	0.03440
Q9UMX5	Neudesin	NENF	14.40	0.11712	1.71	1.00000	8.43	0.11712
O60568	Procollagen-lysine,2-oxoglutarate 5-dioxygenase 3	PLOD3	6.43	0.00366	3.61	0.00004	1.78	0.18970
Q9UNW1	Multiple inositol polyphosphate phosphatase 1	MINPP1	6.28	0.00214	3.40	0.00573	1.85	0.02399
O43657	Tetraspanin-6	TSPAN6	6.64	0.11634	3.09	0.11665	2.15	0.42905
P13497	Bone morphogenetic protein 1	BMP1	7.52	0.01822	2.08	0.01227	3.62	0.11693
P07237	Protein disulfide-isomerase	P4HB	8.72	0.00165	1.64	0.06116	5.31	0.00203
O00391	Sulfhydryl oxidase 1	QSOX1	5.01	0.00417	2.31	0.02878	2.17	0.03595
O00468	Agrin	AGRN	5.92	0.00108	1.88	0.00052	3.14	0.00333
P06703	Protein S100-A6	S100A6	5.11	0.18609	2.12	0.00532	2.41	0.69744
Q09328	Alpha-1,6-mannosylglycoprotein 6-beta-N-acetylglucosaminy	MGAT5	5.04	0.01822	1.77	0.01073	2.86	0.15344
P34896	Serine hydroxymethyltransferase, cytosolic	SHMT1	1.60	1.00000	5.18	0.00015	0.31	0.00015
O95994	Anterior gradient protein 2 homolog	AGR2	NaN	0.02034	NaN	0.37390	10.29	0.02103
P36952	Serpin B5	SERPINB5	NaN	0.00414	NaN	0.00029	0.71	0.64002
O95395	Beta-1,3-galactosyl-O-glycosyl-glycoprotein beta-1,6-N-acetyl	GCNT3	NaN	0.00084	NaN	0.00007	1.39	0.67709
Q13740	CD166 antigen	ALCAM	NaN	0.37390	NaN	0.00000	0.56	0.00714
P00352	Retinal dehydrogenase 1	ALDH1A1	NaN	0.00690	NaN	0.00004	0.81	0.55544
P49788	Retinoic acid receptor responder protein 1	RARRES1	NaN	0.00243	NaN	0.11876	1.84	0.11159
Q9HCY8	Protein S100-A14	S100A14	NaN	0.00012	NaN	0.00111	0.84	0.13449
P03950	Angiogenin	ANG	NaN	0.00000	NaN	0.00008	1.87	0.00122
P34096	Ribonuclease 4	RNASE4	NaN	0.00013	NaN	0.12157	1.48	0.36833
Q08397	Lysyl oxidase homolog 1	LOXL1	NaN	0.12607	NaN	1.00000	NaN	0.12607
Q13753	Laminin subunit gamma-2	LAMC2	NaN	0.00007	NaN	0.12093	1.69	0.02203
P22223	Cadherin-3	CDH3	NaN	0.12529	NaN	0.11617	1.53	0.91117
Q9Y5Y6	Suppressor of tumorigenicity 14 protein	ST14	NaN	1.00000	NaN	0.00017	1.07	0.00017
P32929	Cystathionine gamma-lyase	CTH	NaN	1.00000	NaN	0.00013	0.10	0.00013
P48745	Protein NOV homolog	NOV	NaN	1.00000	NaN	0.00323	0.31	0.00323
Q14766	Latent-transforming growth factor beta-binding protein 1	LTBP1	NaN	0.12221	NaN	0.00000	1.53	0.68986
O15551	Claudin-3	CLDN3	NaN	0.12222	NaN	1.00000	2.75	0.12222
O14786	Neuropilin-1	NRP1	NaN	0.11657	NaN	0.00047	0.76	0.26448
P09341	GRO-alpha(4-73)	CXCL1	NaN	0.03406	NaN	0.11911	2.58	0.06224
P00918	Carbonic anhydrase 2	CA2	NaN	0.11633	NaN	0.00001	0.92	0.15820
Q969P0	Immunoglobulin superfamily member 8	IGSF8	NaN	0.00038	NaN	0.11658	2.54	0.01565

P13674	Prolyl 4-hydroxylase subunit alpha-1	P4HA1	NaN	0.00006	NaN	1.00000	NaN	0.00006
O14657	Torsin-1B	TOR1B	NaN	0.00004	NaN	0.11640	0.93	0.23025
P03956	22 kDa interstitial collagenase	MMP1	NaN	0.00092	NaN	1.00000	NaN	0.00092
O15240	Antimicrobial peptide VGF[554-577]	VGF	NaN	1.00000	NaN	0.00003	0.23	0.00003
Q9UNH7	Sorting nexin-6	SNX6	NaN	1.00000	NaN	0.00044	0.60	0.00044

Protein IDs	Protein name	Gene names	KM12L4 vs SW620		KM12SM vs SW620		KM12L4 vs KM12SM	
			Ratio	p-value	Ratio	p-value	Ratio	p-value
P26038	Moesin	MSN	0.02	0.00002	0.01	0.00002	2.02	1.00000
Q99574	Neuroserpin	SERPINI1	0.01	0.00000	0.01	0.00000	1.36	1.00000
Q92598	Heat shock protein 105 kDa	HSPH1	0.03	0.00002	0.04	0.00002	0.96	1.00000
P16035	Metalloproteinase inhibitor 2	TIMP2	0.06	0.00011	0.05	0.00011	1.32	1.00000
P13796	Plastin-2	LCP1	0.08	0.00657	0.12	0.00737	0.62	0.37390
Q02413	Desmoglein-1	DSG1	0.22	0.00318	0.05	0.00318	4.31	1.00000
P24821	Tenascin	TNC	0.10	0.00036	0.16	0.00039	0.63	0.17006
P62328	Hematopoietic system regulatory peptide	TMSB4X	0.07	0.00466	0.23	0.01551	0.32	0.00119
P08670	Vimentin	VIM	0.12	0.00115	0.22	0.00174	0.52	0.01313
P16401	Histone H1.5	HIST1H1B	0.22	0.00175	0.13	1.00000	1.69	0.00175
P25311	Zinc-alpha-2-glycoprotein	AZGP1	0.19	0.05665	0.17	0.07344	1.17	0.36248
Q08554	Desmocollin-1	DSC1	0.47	0.24448	0.07	0.00260	6.69	0.00032
O15067	Phosphoribosylformylglycinamidine synthase	PFAS	0.15	0.00476	0.24	0.00476	0.60	1.00000
Q9UNZ2	NSFL1 cofactor p47	NSFL1C	0.21	0.00688	0.18	0.00162	1.16	0.95734
P05161	Ubiquitin-like protein ISG15	ISG15	0.12	0.00264	0.35	0.02194	0.34	0.00013
Q9Y6N7	Roundabout homolog 1	ROBO1	0.22	0.00006	0.20	0.00006	1.13	1.00000
Q96IU4	Alpha/beta hydrolase domain-containing protein 14B	ABHD14B	0.20	0.00510	0.24	0.00510	0.86	1.00000
P09211	Glutathione S-transferase P	GSTP1	0.31	0.05668	0.16	0.01908	1.93	0.32858
O60506	Heterogeneous nuclear ribonucleoprotein Q	SYNCRIP	0.19	0.00488	0.31	0.02193	0.63	0.36552
Q04828	Aldo-keto reductase family 1 member C1	AKR1C1	0.19	0.00019	0.34	0.00164	0.55	0.37390
Q01581	Hydroxymethylglutaryl-CoA synthase, cytoplasmic	HMGCS1	0.18	0.02239	0.36	0.26638	0.50	0.30981
Q16658	Fascin	FSCN1	0.44	0.02355	0.16	0.00073	2.67	0.01412
P23588	Eukaryotic translation initiation factor 4B	EIF4B	0.20	0.00005	0.45	0.01371	0.44	0.11638
P00492	Hypoxanthine-guanine phosphoribosyltransferase	HPRT1	0.19	0.00524	0.51	0.00859	0.37	0.03168
P15374	Ubiquitin carboxyl-terminal hydrolase isozyme L3	UCHL3	0.17	0.13089	0.60	0.13089	0.28	1.00000
P43487	Ran-specific GTPase-activating protein	RANBP1	0.20	1.00000	0.59	0.01167	0.33	0.01167
P15924	Desmoplakin	DSP	0.64	0.05884	0.19	0.02615	3.37	0.00022
Q8WWI1	LIM domain only protein 7	LMO7	0.20	1.00000	0.65	0.00077	0.31	0.00077
Q13308	Inactive tyrosine-protein kinase 7	PTK7	0.00	0.00014	0.00	0.00014	NaN	1
Q6P988	Palmitoleoyl-protein carboxylesterase NOTUM	NOTUM	0.00	0.00012	0.00	0.00012	NaN	1
Q9H9K5	Endogenous retrovirus group MER34 member 1 Env po	ERVMER34-1	0.00	0.00002	0.00	0.00002	NaN	1
P09455	Retinol-binding protein 1	RBP1	0.00	0.00944	0.00	0.00944	NaN	1
Q9H9S4	Calcium-binding protein 39-like	CAB39L	0.00	0.0003	0.00	0.0003	NaN	1
Q16769	Glutaminyl-peptide cyclotransferase	QPCT	0.00	0.00002	0.00	0.00002	NaN	1
