# Supplementary material for: A prognostic six‐gene expression risk‐score derived from proteomic profiling of the metastatic colorectal cancer secretome
Source: J Pathol Clin Res. 2022 Sep 22;8(6):495–508. doi: 10.1002/cjp2.294 (PMC9535096; doi:10.1002/cjp2.294)
Supplement: Supplementary file 5 — Table S3. List of gene alterations between KM12SM and SW620 cells [file CJP2-8-495-s004.pdf]

A prognostic six-gene expression risk-score derived from proteomic profiling of the metastatic colorectal cancer secretome

J Robles *et al.* *J Pathol Clin Res* DOI: https://doi.org/10.1002/cjp2.294

Table S3. List of gene alterations between KM12SM and SW620 cells

ID	Gene Symbol	Group	KM12SM	SW620	FC	P-val	FDR P-val
TC0800008663.hg.1	MAL2	Multiple_Complex	12.16	3.74	343.47	3.75E-18	8.04E-14
TC0700009411.hg.1	MGAM2	Multiple_Complex	11.13	3.26	233.79	6.38E-16	1.95E-12
TC0100015477.hg.1	REG4	Coding	12.15	4.56	192.66	4.08E-17	4.37E-13
TC0700010355.hg.1	AGR2	Multiple_Complex	11.58	4.46	138.91	1.80E-15	3.85E-12
TC1200012601.hg.1	SLCO1B3	Multiple_Complex	10.81	3.83	126.65	1.60E-16	6.85E-13
TC1200008133.hg.1	LYZ	Multiple_Complex	12.68	5.81	116.83	9.19E-17	6.42E-13
TC1800007543.hg.1	SERPINB5	Multiple_Complex	10.13	3.29	114.54	1.20E-16	6.42E-13
TC0200009707.hg.1	KCNJ3	Multiple_Complex	10.35	4.11	75.66	5.34E-16	1.91E-12
TC0600013998.hg.1	THBS2	Multiple_Complex	11.4	5.23	72.15	9.54E-13	2.05E-10
TC0100017084.hg.1	CTSE	Multiple_Complex	11.04	4.95	67.98	1.54E-14	1.14E-11
TC0900012245.hg.1	ALDH1A1	Multiple_Complex	10.52	4.6	60.41	1.75E-15	3.85E-12
TC0200014764.hg.1	DPP4	Multiple_Complex	9.55	3.73	56.4	2.63E-15	4.51E-12
TC0100010149.hg.1	RAB25	Multiple_Complex	9.96	4.15	55.82	4.29E-14	2.42E-11
TC0600009439.hg.1	TMEM200A	Coding	9.23	3.44	55.26	9.25E-16	2.48E-12
TC0400007868.hg.1	PARM1	Multiple_Complex	9.16	3.46	52.06	3.12E-14	1.91E-11
TC1200011460.hg.1	KITLG	Multiple_Complex	10.42	4.86	47.01	5.64E-15	6.05E-12
TC0400008331.hg.1	NPNT	Multiple_Complex	9.69	4.14	46.78	3.97E-13	1.14E-10
TC1300007752.hg.1	CLDN10	Multiple_Complex	10.56	5.04	45.93	3.82E-15	4.88E-12
TC0100010887.hg.1	C1orf21	Multiple_Complex	9.63	4.19	43.42	2.73E-15	4.51E-12
TC0300008242.hg.1	ALCAM	Multiple_Complex	11.3	5.93	41.46	1.43E-13	5.28E-11
TC1500007409.hg.1	GCNT3	Multiple_Complex	10.56	5.18	41.46	3.87E-15	4.88E-12
TC0600008319.hg.1	FAM83B	Multiple_Complex	8.95	3.65	39.5	1.33E-14	1.05E-11
TC0400011279.hg.1	ABCG2	Multiple_Complex	9.16	3.95	37.03	2.37E-14	1.56E-11
TC1900009639.hg.1	AP1M2	Multiple_Complex	10.35	5.15	36.75	3.74E-15	4.88E-12
TC0900009569.hg.1	NFIB	Multiple_Complex	10.65	5.5	35.48	1.48E-14	1.13E-11
TC0100018239.hg.1	GIPC2	Coding	9.58	4.43	35.34	5.50E-15	6.05E-12
TC0100015476.hg.1	HMGCS2	Multiple_Complex	9.47	4.34	34.95	2.07E-14	1.43E-11
TC1200006736.hg.1	A2ML1	Multiple_Complex	10.07	4.95	34.86	5.11E-12	7.17E-10
TC0800011129.hg.1	CDH17	Coding	10.56	5.56	32.06	5.95E-15	6.08E-12
TC1500010864.hg.1	GATM	Multiple_Complex	9.52	4.57	30.91	4.63E-14	2.52E-11
TC0300013884.hg.1	C3orf58	Multiple_Complex	8.14	3.23	30.09	8.37E-14	3.58E-11
TC1900011741.hg.1	CEACAM5	Multiple_Complex	11.57	6.74	28.46	1.10E-13	4.31E-11
TC1100010514.hg.1	ELF5	Multiple_Complex	9.55	4.77	27.58	8.37E-13	1.85E-10
TC1000011740.hg.1	COL17A1	Multiple_Complex	10.49	5.74	26.91	5.69E-13	1.39E-10
TC2100008251.hg.1	TFF1	Multiple_Complex	10.65	5.92	26.54	5.54E-13	1.37E-10
TC0200014809.hg.1	GALNT3	Multiple_Complex	8.58	3.86	26.33	1.29E-12	2.51E-10
TC1700006899.hg.1	MAP2K4	Multiple_Complex	9.02	4.34	25.61	1.28E-13	4.89E-11
TC0600008697.hg.1	NT5E	Multiple_Complex	10.83	6.16	25.54	3.03E-12	4.84E-10

TC0900006522.hg.1	SLC1A1	Multiple_Complex	8.68	4	25.49	1.27E-12	2.49E-10
TC0200008680.hg.1	SLC9A2	Multiple_Complex	7.67	3.06	24.41	1.26E-12	2.49E-10
TC0600012293.hg.1	MB21D1	Multiple_Complex	8.42	3.83	23.93	3.38E-13	1.03E-10
TC0700010358.hg.1	AGR3	Multiple_Complex	7.92	3.36	23.67	4.23E-12	6.22E-10
TC1100012681.hg.1	SIAE	Multiple_Complex	10.22	5.65	23.63	5.27E-13	1.33E-10
TC0100012006.hg.1	KCNK1	Multiple_Complex	10.07	5.51	23.5	1.90E-13	6.69E-11
TC0700010437.hg.1	RAPGEF5	Multiple_Complex	8.21	3.67	23.33	1.78E-13	6.37E-11
TC0700010965.hg.1	IGFBP3	Multiple_Complex	10.24	5.7	23.18	3.91E-13	1.13E-10
TC0300007645.hg.1	PTPRG	Multiple_Complex	8.16	3.64	22.88	5.42E-13	1.35E-10
TC0100010939.hg.1	PLA2G4A	Multiple_Complex	7.93	3.43	22.64	7.22E-14	3.23E-11
TC1800007298.hg.1	LIPG	Multiple_Complex	9.19	4.69	22.62	3.04E-10	2.03E-08
TC1100006911.hg.1	SPON1	Multiple_Complex	9	4.51	22.43	1.15E-11	1.40E-09
TC0300008316.hg.1	PVRL3	Multiple_Complex	7.97	3.52	21.88	5.33E-14	2.54E-11
TC1400010777.hg.1	TC2N	Multiple_Complex	10.59	6.17	21.46	1.44E-11	1.68E-09
TC0900007488.hg.1	PIP5K1B	Multiple_Complex	9.23	4.81	21.29	1.48E-11	1.70E-09
TC0500007826.hg.1	IQGAP2	Multiple_Complex	9.35	4.99	20.6	2.94E-13	9.26E-11
TC0800011532.hg.1	TRPS1	Multiple_Complex	8.25	3.89	20.59	1.10E-13	4.31E-11
TC1800009268.hg.1	DSC2	Multiple_Complex	9.99	5.65	20.25	1.33E-12	2.55E-10
TC0400008879.hg.1	GAB1	Multiple_Complex	7.54	3.24	19.58	3.61E-13	1.06E-10
TC1200011002.hg.1	LRIG3	Multiple_Complex	7.99	3.71	19.44	5.13E-14	2.54E-11
TC0600009783.hg.1	UST	Multiple_Complex	8.22	3.95	19.36	4.63E-13	1.26E-10
TC0100016678.hg.1	FAM129A	Multiple_Complex	7.82	3.55	19.23	8.76E-14	3.61E-11
TC0300010930.hg.1	SLC6A20	Multiple_Complex	10.14	5.89	19.08	7.33E-13	1.71E-10
TC0500007761.hg.1	TMEM171	Coding	8.91	4.71	18.47	4.64E-11	4.43E-09
TC0300013068.hg.1	MECOM	Multiple_Complex	9.28	5.13	17.74	4.17E-13	1.16E-10
TC0700007905.hg.1	AUTS2	Multiple_Complex	8.65	4.53	17.29	2.40E-12	4.08E-10
TC0300012945.hg.1	RARRES1	Multiple_Complex	8.75	4.65	17.25	2.37E-11	2.56E-09
TC0100014988.hg.1	F3	Multiple_Complex	10.31	6.23	16.99	1.95E-12	3.51E-10
TC0900011203.hg.1	PTGR1	Multiple_Complex	8.61	4.53	16.96	1.19E-11	1.45E-09
TC1900007836.hg.1	FXYD3	Multiple_Complex	10.75	6.66	16.95	2.20E-11	2.41E-09
TC0800011601.hg.1	SAMD12	Multiple_Complex	8.56	4.48	16.89	9.47E-13	2.05E-10
TC1900010625.hg.1	LGALS4	Multiple_Complex	10.14	6.08	16.64	1.19E-12	2.42E-10
TC1200011251.hg.1	TSPAN8	Multiple_Complex	7.75	3.7	16.58	4.99E-11	4.67E-09
TC0400008020.hg.1	CDS1	Multiple_Complex	8.23	4.19	16.49	2.64E-11	2.82E-09
TC1800007013.hg.1	DSG3	Multiple_Complex	7.78	3.75	16.36	5.80E-12	7.83E-10
TC0100013293.hg.1	ID3	Multiple_Complex	9.31	5.29	16.27	5.42E-12	7.46E-10
TC0600008981.hg.1	AIM1	Multiple_Complex	8.15	4.14	16.17	6.68E-13	1.59E-10
TC0700008857.hg.1	MDFIC	Multiple_Complex	7.74	3.73	16.14	1.12E-12	2.30E-10
TC0900010866.hg.1	FBP1	Multiple_Complex	8.84	4.86	15.77	7.64E-13	1.76E-10
TC0200014610.hg.1	NMI	Multiple_Complex	8.44	4.46	15.75	3.44E-12	5.31E-10

TC1100013022.hg.1	FADS2	Multiple_Complex	11.68	7.74	15.35	1.62E-13	5.88E-11
TC0600011957.hg.1	ADGRF1	Multiple_Complex	8	4.07	15.26	5.06E-13	1.31E-10
TC1100012318.hg.1	IL18	Multiple_Complex	7.61	3.68	15.2	4.72E-13	1.27E-10
TC1000008495.hg.1	PLCE1	Multiple_Complex	7.9	3.98	15.07	2.76E-13	8.97E-11
TC0700010568.hg.1	HOXA13	Multiple_Complex	9.25	5.33	15.06	1.31E-12	2.54E-10
TC0X00009069.hg.1	ARHGAP6	Multiple_Complex	8.07	4.16	15.04	3.23E-12	5.10E-10
TC0400008618.hg.1	FGF2	Multiple_Complex	8.38	4.49	14.9	4.15E-11	4.09E-09
TC1600011418.hg.1	CDH3	Multiple_Complex	9.73	5.86	14.67	5.47E-12	7.47E-10
TC0800009735.hg.1	MTUS1	Multiple_Complex	10.37	6.51	14.5	2.57E-11	2.76E-09
TC0400010558.hg.1	ATP8A1	Multiple_Complex	7.31	3.46	14.36	5.95E-12	7.98E-10
TC0800008416.hg.1	GRHL2	Multiple_Complex	9.27	5.44	14.19	1.47E-12	2.69E-10
TC1000011368.hg.1	ANKRD22	Multiple_Complex	8.94	5.12	14.12	8.29E-13	1.85E-10
TC1400008981.hg.1	FOXA1	Multiple_Complex	9.59	5.8	13.9	1.92E-10	1.41E-08
TC0900008776.hg.1	MVB12B	Multiple_Complex	8.48	4.71	13.64	8.56E-13	1.87E-10
TC1700007752.hg.1	PPP1R1B	Multiple_Complex	9.89	6.14	13.49	3.77E-12	5.69E-10
TC1800008317.hg.1	ZNF521	Multiple_Complex	7.69	3.94	13.47	4.82E-12	6.85E-10
TC0X00009341.hg.1	DMD	Multiple_Complex	7.13	3.44	12.96	3.55E-09	1.64E-07
TC1800009222.hg.1	RNF125	Multiple_Complex	7.19	3.5	12.94	4.26E-13	1.17E-10
TC1000008354.hg.1	PAPSS2	Multiple_Complex	9.34	5.64	12.94	3.57E-11	3.58E-09
TC0900010565.hg.1	RASEF	Multiple_Complex	8.4	4.7	12.93	3.37E-11	3.46E-09
TC0400009543.hg.1	TLR3	Multiple_Complex	7.45	3.77	12.9	2.36E-12	4.05E-10
TC0400010242.hg.1	PPARGC1A	Multiple_Complex	8.93	5.29	12.48	1.86E-11	2.07E-09
TC0500006816.hg.1	FAM105A	Multiple_Complex	6.81	3.17	12.46	7.23E-11	6.32E-09
TC1600010861.hg.1	FA2H	Multiple_Complex	8.87	5.25	12.32	1.30E-11	1.55E-09
TC0800012362.hg.1	KHDRBS3	Multiple_Complex	8.67	5.06	12.19	2.32E-12	4.03E-10
TC1200011567.hg.1	TMCC3	Multiple_Complex	8.73	5.14	12.08	6.54E-09	2.76E-07
TC1900006603.hg.1	ZNF556	Coding	6.97	3.38	12.01	6.88E-10	4.02E-08
TC0500008884.hg.1	PCDHB13	Coding	8.26	4.69	11.88	1.10E-12	2.29E-10
TC0900008337.hg.1	ZNF462	Multiple_Complex	8.23	4.67	11.74	9.18E-12	1.18E-09
TC0100014340.hg.1	TACSTD2	Coding	8.45	4.9	11.72	1.33E-07	3.62E-06
TC1500009709.hg.1	CA12	Multiple_Complex	9.61	6.07	11.68	1.05E-11	1.31E-09
TC0600009669.hg.1	ADGRG6	Multiple_Complex	9.13	5.58	11.65	5.04E-12	7.11E-10
TC0800011764.hg.1	FAM84B	Multiple_Complex	9.57	6.04	11.59	1.58E-10	1.21E-08
TC0300013417.hg.1	LIPH	Multiple_Complex	8.82	5.31	11.44	1.27E-11	1.52E-09
TC0100017449.hg.1	CAPN8	Multiple_Complex	9.15	5.63	11.43	2.04E-10	1.48E-08
TC1600008209.hg.1	CDH1	Multiple_Complex	9.28	5.76	11.42	2.99E-11	3.13E-09
TC1300008249.hg.1	GJB2	Coding	9.24	5.74	11.3	2.33E-12	4.03E-10
TC1100009196.hg.1	TMPRSS4	Multiple_Complex	7.82	4.35	11.12	8.35E-10	4.76E-08
TC1400010617.hg.1	PRKCH	Multiple_Complex	8.54	5.07	11.05	8.06E-11	6.94E-09
TC0600014191.hg.1	SAMD5	Multiple_Complex	7.16	3.72	10.87	9.57E-12	1.22E-09

TC1200007182.hg.1	FAR2	Multiple_Complex	8.04	4.6	10.87	2.70E-10	1.86E-08
TC0100015872.hg.1	S100A14	Multiple_Complex	9.33	5.89	10.85	1.47E-12	2.69E-10
TC1000012050.hg.1	FGFR2	Multiple_Complex	9.26	5.82	10.84	7.22E-11	6.32E-09
TC0700008938.hg.1	PTPRZ1	Multiple_Complex	7.4	3.98	10.68	4.50E-10	2.82E-08
TC0100013272.hg.1	HTR1D	Coding	7.3	3.89	10.62	4.31E-12	6.24E-10
TC0200013595.hg.1	MGAT4A	Multiple_Complex	7.13	3.72	10.6	2.64E-12	4.32E-10
TC1700012346.hg.1	CLDN7	Multiple_Complex	10.87	7.47	10.59	3.79E-10	2.43E-08
TC0600011464.hg.1	SLC44A4	Multiple_Complex	8.03	4.63	10.55	1.48E-11	1.70E-09
TC0700009352.hg.1	TBXAS1	Multiple_Complex	8.21	4.81	10.55	2.23E-10	1.58E-08
TC0100014768.hg.1	MCOLN2	Multiple_Complex	6.78	3.39	10.52	9.51E-12	1.21E-09
TC0600011943.hg.1	ENPP5	Multiple_Complex	8.28	4.88	10.5	4.05E-12	6.04E-10
TC1800007360.hg.1	RAB27B	Multiple_Complex	8.09	4.7	10.46	2.11E-12	3.73E-10
TC0300012943.hg.1	LXN	Multiple_Complex	6.5	3.13	10.38	1.85E-10	1.37E-08
TC0600009843.hg.1	PLEKHG1	Multiple_Complex	7.83	4.46	10.36	1.30E-11	1.55E-09
TC0900006968.hg.1	SPINK4	Coding	7.39	4.03	10.29	6.85E-09	2.87E-07
TC0100011406.hg.1	CD55	Multiple_Complex	11.52	8.18	10.16	5.31E-12	7.40E-10
TC1000009884.hg.1	FAM171A1	Multiple_Complex	9.87	6.54	10.03	4.42E-11	4.27E-09
TC1700009528.hg.1	CXCL16	Multiple_Complex	9.12	5.8	10.01	3.11E-12	4.94E-10
TC1200009399.hg.1	PIWIL1	Multiple_Complex	9.25	5.94	9.92	3.08E-08	1.03E-06
TC0700007596.hg.1	EGFR	Multiple_Complex	8.14	4.84	9.89	2.00E-09	1.03E-07
TC1800008781.hg.1	ATP8B1	Multiple_Complex	10.5	7.2	9.85	3.22E-11	3.36E-09
TC0300006758.hg.1	PLCL2	Multiple_Complex	6.73	3.43	9.83	2.97E-12	4.80E-10
TC0100014769.hg.1	MCOLN3	Multiple_Complex	6.34	3.05	9.78	4.29E-12	6.24E-10
TC2200007120.hg.1	SLC5A1	Multiple_Complex	8.27	4.99	9.73	1.94E-09	9.99E-08
TC1000008926.hg.1	ACSL5	Multiple_Complex	9.86	6.58	9.66	2.74E-08	9.26E-07
TC0700006933.hg.1	STK31	Multiple_Complex	6.91	3.65	9.6	2.61E-12	4.30E-10
TC0X00010840.hg.1	HS6ST2	Coding	7.35	4.09	9.57	2.18E-10	1.56E-08
TC1200008016.hg.1	TBC1D30	Multiple_Complex	7.93	4.67	9.55	3.00E-09	1.44E-07
TC1100007216.hg.1	PRRG4	Multiple_Complex	8.98	5.73	9.51	1.52E-11	1.74E-09
TC0700008574.hg.1	MUC3A	Multiple_Complex	8.27	5.02	9.5	2.31E-10	1.63E-08
TC0200015194.hg.1	TFPI	Multiple_Complex	8.65	5.4	9.5	1.54E-09	8.19E-08
TC1600008609.hg.1	HSD17B2	Coding	9.64	6.39	9.5	2.74E-10	1.88E-08
TC1800006852.hg.1	GATA6	Coding	9.41	6.18	9.35	9.71E-10	5.40E-08
TC0400012245.hg.1	FAM198B	Multiple_Complex	9.37	6.16	9.3	2.11E-10	1.53E-08
TC0200009902.hg.1	NOSTRIN	Multiple_Complex	7.22	4.01	9.22	3.57E-11	3.58E-09
TC0600014366.hg.1	PDE10A	Multiple_Complex	8	4.81	9.1	4.71E-11	4.47E-09
TC1000012475.hg.1	FFAR4	Multiple_Complex	8.44	5.25	9.09	1.24E-10	9.69E-09
TC0100013278.hg.1	TCEA3	Multiple_Complex	8.82	5.64	9.05	1.46E-11	1.69E-09
TC0800012323.hg.1	CA13	Multiple_Complex	8.23	5.06	9	3.72E-12	5.66E-10
TC0300009459.hg.1	GPR160	Multiple_Complex	9.08	5.94	8.8	1.03E-10	8.37E-09

TC0200010897.hg.1	SGPP2	Multiple_Complex	9.33	6.19	8.8	1.82E-11	2.04E-09
TC0300012323.hg.1	MGLL	Multiple_Complex	7.99	4.86	8.73	8.32E-10	4.76E-08
TC1900008485.hg.1	FUT2	Multiple_Complex	8.1	4.97	8.73	3.41E-10	2.22E-08
TC1700010967.hg.1	PRR15L	Coding	7.5	4.38	8.71	9.24E-08	2.66E-06
TC1100007273.hg.1	CD44	Multiple_Complex	10.36	7.24	8.7	5.79E-12	7.83E-10
TC1600009147.hg.1	PRSS22	Multiple_Complex	8.07	4.95	8.69	3.22E-06	5.20E-05
TC0X00011259.hg.1	STS	Multiple_Complex	8.48	5.36	8.68	4.12E-10	2.61E-08
TC0400011192.hg.1	PLAC8	Multiple_Complex	11.52	8.4	8.67	2.18E-09	1.10E-07
TC1100010400.hg.1	LGR4	Multiple_Complex	9.45	6.34	8.6	4.27E-11	4.16E-09
TC0300013949.hg.1	SATB1	Multiple_Complex	10.03	6.95	8.51	3.13E-10	2.09E-08
TC0400012178.hg.1	DCHS2	Multiple_Complex	7.86	4.78	8.45	5.95E-09	2.53E-07
TC0200016699.hg.1	TRABD2A	Multiple_Complex	9.29	6.22	8.44	4.34E-11	4.21E-09
TC2100007208.hg.1	MX1	Multiple_Complex	9.45	6.38	8.39	1.05E-10	8.50E-09
TC0400007799.hg.1	SLC4A4	Multiple_Complex	7.23	4.19	8.26	5.82E-11	5.31E-09
TC0900010543.hg.1	TLE1	Multiple_Complex	9.05	6.01	8.23	2.79E-09	1.35E-07
TC1400006529.hg.1	ANG	Multiple_Complex	8.39	5.37	8.14	4.62E-11	4.42E-09
TC0900007752.hg.1	NTRK2	Multiple_Complex	9.06	6.03	8.13	3.48E-11	3.52E-09
TC0900010969.hg.1	CORO2A	Multiple_Complex	8.96	5.94	8.1	2.00E-11	2.21E-09
TC1500008304.hg.1	GDPGP1	Multiple_Complex	8.57	5.55	8.1	8.21E-07	1.64E-05
TC0700008745.hg.1	PRKAR2B	Multiple_Complex	7.34	4.35	7.94	2.91E-11	3.07E-09
TC0X00006704.hg.1	REPS2	Multiple_Complex	6.89	3.94	7.73	5.75E-11	5.27E-09
TC0400012829.hg.1	ARHGEF38	Multiple_Complex	8.47	5.52	7.73	8.95E-11	7.55E-09
TC1200011246.hg.1	PTPRR	Multiple_Complex	7.45	4.5	7.73	3.39E-11	3.47E-09
TC1400009361.hg.1	TMEM30B	Multiple_Complex	8.57	5.62	7.72	1.59E-10	1.21E-08
TC1100010897.hg.1	UBE2L6	Multiple_Complex	9.05	6.1	7.72	2.94E-11	3.09E-09
TC1100012350.hg.1	DRD2	Multiple_Complex	7.74	4.79	7.71	1.36E-10	1.05E-08
TC0100010867.hg.1	LAMC2	Multiple_Complex	9.98	7.06	7.54	5.06E-07	1.09E-05
TC0900009776.hg.1	MOB3B	Multiple_Complex	8.15	5.25	7.47	3.08E-09	1.46E-07
TC1900008432.hg.1	EHD2	Multiple_Complex	8.91	6.01	7.46	6.30E-07	1.31E-05
TC1500007829.hg.1	LOXL1	Multiple_Complex	9.08	6.19	7.42	9.76E-10	5.41E-08
TC1200011474.hg.1	ATP2B1	Multiple_Complex	9.96	7.07	7.39	5.56E-10	3.38E-08
TC1900009439.hg.1	CD70	Multiple_Complex	7.96	5.08	7.38	8.84E-10	4.99E-08
TC0800010926.hg.1	PAG1	Multiple_Complex	8.52	5.64	7.37	1.42E-08	5.33E-07
TC1100007445.hg.1	CREB3L1	Multiple_Complex	8.13	5.25	7.37	1.13E-11	1.40E-09
TC0800010252.hg.1	ANK1	Multiple_Complex	8.91	6.04	7.31	4.01E-10	2.57E-08
TC0100017500.hg.1	TMEM63A	Multiple_Complex	8.95	6.09	7.29	4.76E-11	4.49E-09
TC1600007107.hg.1	TMC5	Multiple_Complex	8.6	5.75	7.2	1.85E-10	1.37E-08
TC1800009229.hg.1	SMAD4	Multiple_Complex	8.29	5.45	7.15	2.72E-11	2.89E-09
TC0400010750.hg.1	NMU	Multiple_Complex	7.45	4.62	7.14	2.59E-10	1.80E-08
TC1700006911.hg.1	ARHGAP44	Multiple_Complex	7.05	4.22	7.11	4.53E-11	4.36E-09

TC0500008156.hg.1	ERAP2	Multiple_Complex	7.75	4.92	7.11	2.32E-09	1.16E-07
TC0400012150.hg.1	TMEM154	Multiple_Complex	7.25	4.44	7.01	1.32E-09	7.18E-08
TC0500007848.hg.1	PDE8B	Multiple_Complex	7.9	5.09	6.99	1.33E-10	1.03E-08
TC0100014531.hg.1	WLS	Multiple_Complex	10.39	7.59	6.94	1.12E-10	8.96E-09
TC0200016651.hg.1	CYP1B1	Multiple_Complex	7.51	4.73	6.89	3.17E-10	2.11E-08
TC0800011211.hg.1	NIPAL2	Multiple_Complex	6.98	4.2	6.87	1.00E-10	8.19E-09
TC0400011203.hg.1	HPSE	Multiple_Complex	6.95	4.18	6.82	3.11E-10	2.08E-08
TC1200012708.hg.1	OAS1	Multiple_Complex	9.37	6.6	6.81	6.09E-08	1.84E-06
TC0900012209.hg.1	TTC39B	Multiple_Complex	6.91	4.15	6.81	1.20E-08	4.61E-07
TC0600014110.hg.1	PSMB9	Multiple_Complex	7.95	5.18	6.79	4.71E-07	1.02E-05
TC0X00010607.hg.1	KLHL13	Multiple_Complex	7.93	5.17	6.78	2.32E-10	1.63E-08
TC0400007330.hg.1	NSUN7	Multiple_Complex	6.49	3.75	6.7	9.69E-11	8.02E-09
TC0900007680.hg.1	TLE4	Multiple_Complex	6.57	3.83	6.67	5.36E-11	4.98E-09
TC1700010036.hg.1	ALDH3A1	Multiple_Complex	10.63	7.89	6.66	8.65E-11	7.36E-09
TC0200010236.hg.1	GULP1	Multiple_Complex	7.87	5.15	6.59	5.58E-11	5.16E-09
TC1800006897.hg.1	LAMA3	Multiple_Complex	9.93	7.22	6.54	2.18E-10	1.56E-08
TC1400007259.hg.1	PELI2	Multiple_Complex	7.66	4.96	6.5	1.83E-09	9.52E-08
TC0100012008.hg.1	KCNK1	Unassigned	6.7	4	6.5	1.41E-09	7.52E-08
TC0400011144.hg.1	ANTXR2	Multiple_Complex	7.87	5.17	6.49	3.15E-06	5.11E-05
TC1200006896.hg.1	GPRC5A	Multiple_Complex	11.22	8.53	6.44	5.88E-11	5.35E-09
TC0300008561.hg.1	PARP14	Multiple_Complex	9.88	7.19	6.43	8.11E-09	3.28E-07
TC0X00009007.hg.1	PUDP	Multiple_Complex	7.53	4.84	6.43	4.83E-09	2.10E-07
TC0900009978.hg.1	GNE	Multiple_Complex	8.67	5.99	6.42	2.24E-10	1.58E-08
TC1200012016.hg.1	TBX3	Multiple_Complex	9.39	6.72	6.4	2.87E-10	1.94E-08
TC0200016743.hg.1	ITGB6	Multiple_Complex	6.2	3.53	6.39	2.02E-10	1.47E-08
TC0600011351.hg.1	TRIM31	Multiple_Complex	6.78	4.11	6.38	1.92E-10	1.41E-08
TC0600010707.hg.1	NRN1	Multiple_Complex	8.23	5.56	6.36	2.26E-07	5.56E-06
TC0400009033.hg.1	TRIM2	Multiple_Complex	10.81	8.14	6.35	2.60E-10	1.80E-08
TC0600008655.hg.1	TPBG	Multiple_Complex	8.21	5.55	6.32	2.81E-10	1.92E-08
TC1900011742.hg.1	CEACAM6	Coding	9.22	6.57	6.29	2.37E-08	8.23E-07
TC0100009324.hg.1	FAM102B	Multiple_Complex	9.74	7.1	6.25	1.75E-10	1.31E-08
TC0500011648.hg.1	EFNA5	Multiple_Complex	9.28	6.64	6.24	3.40E-10	2.22E-08
TC2000007209.hg.1	PROCR	Coding	9.07	6.43	6.21	9.84E-09	3.88E-07
TC0400011994.hg.1	INPP4B	Multiple_Complex	6.62	4.02	6.09	1.91E-09	9.83E-08
TC1700010209.hg.1	TLCD1	Multiple_Complex	9.33	6.74	6.04	9.07E-10	5.08E-08
TC0400010704.hg.1	LNX1	Multiple_Complex	6.39	3.8	6.01	2.07E-09	1.05E-07
TC0300008371.hg.1	GRAMD1C	Multiple_Complex	6.14	3.55	6	2.95E-10	1.99E-08
TC0800008002.hg.1	HNF4G	Multiple_Complex	9.15	6.57	5.97	5.25E-07	1.12E-05
TC0700006735.hg.1	SCIN	Multiple_Complex	6.08	3.5	5.97	6.58E-10	3.91E-08
TC0100009621.hg.1	PHGDH	Multiple_Complex	10.65	8.08	5.95	1.81E-08	6.49E-07

TC1100009524.hg.1	ST14	Multiple_Complex	10.12	7.55	5.94	3.37E-10	2.22E-08
TC2000007239.hg.1	EPB41L1	Multiple_Complex	9.46	6.89	5.91	4.59E-09	2.01E-07
TC1000008955.hg.1	PLEKHS1	Coding	6.15	3.59	5.88	4.12E-09	1.86E-07
TC0100016476.hg.1	KIAA0040	Multiple_Complex	9.34	6.78	5.87	1.72E-09	9.03E-08
TC1200006555.hg.1	CCND2	Multiple_Complex	10.07	7.52	5.86	4.84E-10	3.01E-08
TC1200009136.hg.1	CABP1	Multiple_Complex	7.98	5.43	5.85	6.21E-10	3.71E-08
TC0700011052.hg.1	DDC	Multiple_Complex	6.45	3.9	5.85	8.23E-08	2.42E-06
TC0300012728.hg.1	PLSCR1	Multiple_Complex	9.39	6.85	5.8	5.50E-10	3.36E-08
TC0600007862.hg.1	PIM1	Multiple_Complex	8.52	5.99	5.79	7.25E-11	6.32E-09
TC1600008646.hg.1	ATP2C2	Multiple_Complex	8.58	6.07	5.7	2.68E-09	1.31E-07
TC0200015538.hg.1	KLF7	Multiple_Complex	8.28	5.77	5.7	1.06E-10	8.53E-09
TC1800007506.hg.1	TNFRSF11A	Multiple_Complex	7.88	5.37	5.7	3.50E-07	8.02E-06
TC1900011908.hg.1	PDE4C	Multiple_Complex	7.19	4.68	5.67	5.56E-08	1.71E-06
TC1300008359.hg.1	PARP4	Multiple_Complex	9.81	7.31	5.66	1.10E-08	4.28E-07
TC0100014852.hg.1	GBP1	Multiple_Complex	6.12	3.63	5.62	1.48E-09	7.88E-08
TC0100008517.hg.1	NFIA	Multiple_Complex	9.32	6.83	5.61	1.72E-10	1.30E-08
TC1200006862.hg.1	BCL2L14	Multiple_Complex	7.78	5.3	5.6	1.15E-09	6.34E-08
TC0300011550.hg.1	PDZRN3	Multiple_Complex	8.59	6.11	5.59	7.38E-10	4.28E-08
TC0100010543.hg.1	ATP1B1	Multiple_Complex	12.2	9.72	5.59	2.61E-10	1.80E-08
TC1200009137.hg.1	MLEC	Multiple_Complex	12.15	9.67	5.56	1.37E-09	7.37E-08
TC0200007237.hg.1	CRIM1	Multiple_Complex	8.46	5.99	5.55	4.95E-06	7.47E-05
TC0500011146.hg.1	ENC1	Multiple_Complex	8.3	5.83	5.54	4.46E-09	1.98E-07
TC0100008697.hg.1	CTH	Multiple_Complex	8.12	5.67	5.49	3.07E-09	1.46E-07
TC2100008527.hg.1	SLC37A1	Multiple_Complex	9.81	7.35	5.49	3.77E-09	1.74E-07
TC0400011712.hg.1	PDE5A	Multiple_Complex	5.99	3.54	5.49	3.03E-09	1.45E-07
TC0700009369.hg.1	RAB19	Multiple_Complex	7.67	5.21	5.48	8.29E-10	4.75E-08
TC1300008253.hg.1	CRYL1	Multiple_Complex	8.06	5.62	5.44	9.32E-10	5.20E-08
TC0300013336.hg.1	LAMP3	Multiple_Complex	7.2	4.76	5.43	2.30E-08	8.04E-07
TC1900009970.hg.1	BST2	Multiple_Complex	6.27	3.83	5.42	2.96E-10	1.99E-08
TC1200008353.hg.1	NTS	Multiple_Complex	5.8	3.36	5.42	1.28E-08	4.87E-07
TC0400011978.hg.1	TBC1D9	Multiple_Complex	6.37	3.94	5.42	2.24E-10	1.58E-08
TC0400012990.hg.1	DDX60L	Multiple_Complex	6.94	4.5	5.41	2.39E-09	1.19E-07
TC0100018366.hg.1	CHRM3	NonCoding	7.71	5.28	5.4	1.79E-08	6.43E-07
TC0400011685.hg.1	PRSS12	Multiple_Complex	8.13	5.7	5.39	8.99E-10	5.06E-08
TC0700006795.hg.1	AHR	Multiple_Complex	12.58	10.14	5.39	2.21E-10	1.57E-08
TC0400012792.hg.1	NIPAL1	Multiple_Complex	8.47	6.04	5.39	3.70E-08	1.20E-06
TC0400007853.hg.1	EREG	Multiple_Complex	9.78	7.35	5.36	2.19E-09	1.11E-07
TC0400012818.hg.1	CCNG2	Multiple_Complex	9.01	6.59	5.36	1.26E-07	3.44E-06
TC0100015629.hg.1	FMO5	Multiple_Complex	6.34	3.92	5.35	4.51E-08	1.42E-06
TC1200010006.hg.1	PLBD1	Multiple_Complex	11.09	8.7	5.26	6.62E-10	3.91E-08

TC0200014834.hg.1	STK39	Multiple_Complex	9.97	7.58	5.26	4.58E-10	2.86E-08
TC0700011626.hg.1	SEMA3C	Multiple_Complex	6.81	4.42	5.24	5.10E-09	2.22E-07
TC0100017748.hg.1	SIPA1L2	Multiple_Complex	7.95	5.56	5.24	1.28E-08	4.87E-07
TC0300007383.hg.1	DAG1	Multiple_Complex	10.58	8.22	5.12	1.82E-06	3.20E-05
TC0X00011338.hg.1	ARSD	Multiple_Complex	7.27	4.92	5.1	2.60E-09	1.27E-07
TC1600009749.hg.1	ERN2	Multiple_Complex	8.18	5.84	5.07	5.58E-10	3.38E-08
TC0800008674.hg.1	DEPTOR	Multiple_Complex	7.8	5.46	5.07	3.13E-09	1.48E-07
TC0400010258.hg.1	LGI2	Coding	6.89	4.55	5.06	6.99E-09	2.91E-07
TC1200010182.hg.1	BHLHE41	Multiple_Complex	10.24	7.9	5.05	4.08E-10	2.59E-08
TC0100014849.hg.1	GBP3	Multiple_Complex	8.39	6.05	5.05	2.51E-09	1.24E-07
TC0700009560.hg.1	CNTNAP2	Multiple_Complex	8.49	6.16	5.03	6.82E-08	2.05E-06
TC2200007242.hg.1	APOL1	Multiple_Complex	6.37	4.04	5.02	1.95E-08	6.92E-07
TC0100015752.hg.1	CTSS	Multiple_Complex	6.57	4.25	5	2.04E-09	1.04E-07
TC1100008257.hg.1	ANO1	Multiple_Complex	9.32	7	5	6.69E-09	2.82E-07
TC0500007231.hg.1	PTGER4	Multiple_Complex	7.06	4.75	4.98	2.12E-08	7.46E-07
TC0600012747.hg.1	CD24	Multiple_Complex	12.02	9.71	4.96	5.91E-10	3.55E-08
TC0500007415.hg.1	GPX8	Multiple_Complex	7.58	5.28	4.91	3.82E-09	1.75E-07
TSUnmapped00000263.hg.1	BCL2L14	NonCoding	5.63	3.34	4.88	2.02E-09	1.03E-07
TC1000008875.hg.1	ADD3	Multiple_Complex	8.79	6.51	4.87	1.33E-08	5.05E-07
TC1900010864.hg.1	KCNN4	Multiple_Complex	9.2	6.92	4.86	1.07E-07	3.01E-06
TC0200014772.hg.1	IFIH1	Multiple_Complex	7.7	5.42	4.86	7.00E-10	4.08E-08
TC0300013103.hg.1	RPL22L1	Multiple_Complex	11.4	9.12	4.85	2.68E-09	1.31E-07
TC0600008146.hg.1	RUNX2	Multiple_Complex	8.4	6.12	4.85	3.75E-10	2.41E-08
TC0X00008056.hg.1	CLDN2	Coding	7.24	4.97	4.82	5.53E-09	2.37E-07
TC1700012281.hg.1	EPN3	Multiple_Complex	8.06	5.8	4.81	1.21E-06	2.26E-05
TC1000007990.hg.1	DDIT4	Multiple_Complex	10.86	8.59	4.81	7.60E-09	3.09E-07
TC0700007002.hg.1	HOXA-AS3	Multiple_Complex	8.69	6.42	4.81	4.33E-09	1.93E-07
TC0100014776.hg.1	DDAH1	Multiple_Complex	10.49	8.22	4.8	2.36E-08	8.23E-07
TC1200008921.hg.1	OAS2	Multiple_Complex	6.52	4.27	4.77	6.20E-09	2.63E-07
TC0200015578.hg.1	IDH1	Multiple_Complex	10.25	8	4.76	1.31E-08	4.98E-07
TC0X00006957.hg.1	XK	Multiple_Complex	7.19	4.94	4.75	9.80E-09	3.88E-07
TC2000007015.hg.1	PYGB	Multiple_Complex	11.01	8.76	4.75	6.64E-10	3.91E-08
TC1500007062.hg.1	CKMT1B	Multiple_Complex	10.41	8.16	4.73	1.55E-08	5.69E-07
TC1400010584.hg.1	IRF9	Multiple_Complex	8.84	6.6	4.73	8.42E-08	2.47E-06
TC1500010792.hg.1	MCTP2	Multiple_Complex	6.97	4.73	4.73	1.52E-08	5.64E-07
TC2100008249.hg.1	TFF3	Multiple_Complex	9.58	7.34	4.72	2.06E-06	3.56E-05
TC1400009697.hg.1	NPC2	Multiple_Complex	9.46	7.23	4.69	1.95E-07	4.95E-06
TC0500013261.hg.1	GALNT10	Multiple_Complex	9.02	6.79	4.69	2.24E-08	7.87E-07
TC0100013445.hg.1	IFI6	Multiple_Complex	10.59	8.37	4.68	1.95E-05	0.0002
TC1200011245.hg.1	PTPRB	Multiple_Complex	6.13	3.91	4.68	6.78E-06	9.74E-05

TC0100009109.hg.1	SLC44A3	Multiple_Complex	8.64	6.42	4.65	1.98E-09	1.02E-07
TC1400007566.hg.1	SMOC1	Multiple_Complex	8.55	6.34	4.64	1.21E-07	3.33E-06
TC1200012111.hg.1	TAOK3	Multiple_Complex	7.48	5.26	4.63	1.20E-07	3.32E-06
TC0500008568.hg.1	SLC12A2	Multiple_Complex	11.62	9.41	4.63	5.42E-09	2.33E-07
TC0100011205.hg.1	ELF3	Multiple_Complex	8.98	6.77	4.62	3.89E-09	1.78E-07
TC1600011517.hg.1	DOC2A	Multiple_Complex	8.33	6.12	4.62	4.74E-09	2.07E-07
TC1500007510.hg.1	TPM1	Multiple_Complex	9.88	7.68	4.6	1.66E-09	8.73E-08
TC0100007645.hg.1	TINAGL1	Multiple_Complex	9.89	7.7	4.58	1.52E-08	5.61E-07
TC1400008054.hg.1	OTUB2	Coding	8.44	6.24	4.57	4.00E-09	1.82E-07
TC0200009626.hg.1	LYPD6	Multiple_Complex	6.68	4.49	4.56	3.95E-08	1.27E-06
TC1800007829.hg.1	HSBP1L1	Multiple_Complex	9.38	7.19	4.56	1.91E-08	6.79E-07
TC0800011595.hg.1	EXT1	NonCoding	7.01	4.82	4.56	8.17E-08	2.41E-06
TC0100013182.hg.1	CAMK2N1	Multiple_Complex	10.84	8.65	4.55	1.54E-09	8.19E-08
TC0400009431.hg.1	TENM3	Multiple_Complex	7.57	5.39	4.55	2.42E-08	8.38E-07
TC0800007460.hg.1	HGSNAT	Multiple_Complex	9.25	7.07	4.54	1.74E-06	3.08E-05
TC1400007133.hg.1	ABHD12B	Multiple_Complex	9.74	7.56	4.53	5.33E-09	2.29E-07
TC0300009353.hg.1	PPM1L	Multiple_Complex	7.87	5.69	4.52	1.26E-07	3.44E-06
TC1100011128.hg.1	ATL3	Multiple_Complex	9.23	7.06	4.52	9.87E-08	2.81E-06
TC0600008539.hg.1	CD109	Multiple_Complex	9.75	7.57	4.51	3.24E-09	1.52E-07
TC0100007638.hg.1	SERINC2	Multiple_Complex	9.33	7.17	4.47	1.08E-07	3.05E-06
TC0400012947.hg.1	GPRIN3	Coding	6.01	3.85	4.46	2.64E-08	8.97E-07
TC1000011524.hg.1	PIK3AP1	Multiple_Complex	9.43	7.27	4.46	4.32E-08	1.38E-06
TC0200015314.hg.1	STK17B	Multiple_Complex	9.25	7.1	4.46	4.29E-08	1.37E-06
TC0100008696.hg.1	HHLA3	Multiple_Complex	8.74	6.58	4.45	1.98E-05	0.0002
TC0600012299.hg.1	SLC17A5	Multiple_Complex	8.66	6.51	4.45	1.00E-08	3.92E-07
TC1100007729.hg.1	DTX4	Multiple_Complex	7.02	4.87	4.45	1.37E-08	5.16E-07
TC0300014061.hg.1	PLSCR2	Multiple_Complex	7.46	5.31	4.44	2.94E-07	6.94E-06
TC1000008800.hg.1	GSTO1	Multiple_Complex	9.38	7.23	4.43	2.82E-09	1.36E-07
TC0900010591.hg.1	SLC28A3	Multiple_Complex	8.56	6.42	4.41	2.16E-07	5.37E-06
TC1200008322.hg.1	TMTC2	Multiple_Complex	8.68	6.55	4.36	1.63E-09	8.63E-08
TC0200011624.hg.1	CMPK2	Multiple_Complex	6.5	4.39	4.34	4.66E-08	1.46E-06
TC0100014772.hg.1	SYDE2	Multiple_Complex	7.5	5.38	4.34	3.46E-09	1.61E-07
TC0400007901.hg.1	SHROOM3	Multiple_Complex	9.91	7.8	4.32	2.15E-07	5.35E-06
TC0800007080.hg.1	BNIP3L	Multiple_Complex	10.89	8.78	4.31	1.23E-09	6.73E-08
TC0100012921.hg.1	DHRS3	Multiple_Complex	9.61	7.51	4.31	3.34E-07	7.68E-06
TC0X00010207.hg.1	POF1B	Multiple_Complex	10.58	8.48	4.31	3.39E-09	1.58E-07
TC0100017420.hg.1	DUSP10	Multiple_Complex	8.11	6.01	4.3	1.02E-08	4.01E-07
TC1400007430.hg.1	SYNE2	Multiple_Complex	7.93	5.82	4.3	2.65E-08	9.01E-07
TC0700006844.hg.1	ITGB8	Multiple_Complex	7.58	5.48	4.29	5.73E-07	1.21E-05
TC0300008558.hg.1	DTX3L	Multiple_Complex	8.59	6.49	4.28	5.12E-09	2.22E-07

TC0200014792.hg.1	COBLL1	Multiple_Complex	7.04	4.94	4.26	1.91E-06	3.34E-05
TC0100017172.hg.1	IRF6	Multiple_Complex	8.09	6	4.26	5.60E-08	1.72E-06
TC0900009779.hg.1	C9orf72	Multiple_Complex	5.65	3.56	4.26	6.38E-06	9.27E-05
TC0400011920.hg.1	SLC7A11	Multiple_Complex	11.14	9.05	4.26	3.07E-09	1.46E-07
TC0X00006446.hg.1	CSF2RA	Multiple_Complex	5.63	3.54	4.26	4.29E-08	1.37E-06
TC0700010348.hg.1	SOSTDC1	Coding	7.7	5.62	4.25	2.32E-08	8.10E-07
TC2000006674.hg.1	PLCB4	Multiple_Complex	7.54	5.46	4.25	1.10E-08	4.28E-07
TC1700010358.hg.1	MYO1D	Multiple_Complex	9.7	7.61	4.25	2.40E-09	1.20E-07
TC1500007067.hg.1	CKMT1A	Multiple_Complex	10.47	8.4	4.22	5.65E-09	2.42E-07
TC0600010095.hg.1	MAP3K4	Multiple_Complex	8.5	6.43	4.19	7.08E-09	2.94E-07
TC0400012366.hg.1	DDX60	Multiple_Complex	6.96	4.89	4.19	1.80E-08	6.46E-07
TC1600009580.hg.1	XYLT1	Multiple_Complex	7.92	5.85	4.18	3.65E-09	1.68E-07
TC0700011834.hg.1	PON3	Multiple_Complex	8.36	6.3	4.18	1.55E-08	5.68E-07
TC0400007232.hg.1	C4orf19	Coding	6.88	4.82	4.16	2.51E-07	6.06E-06
TC1800008301.hg.1	OSBPL1A	Multiple_Complex	9.58	7.52	4.15	2.47E-08	8.50E-07
TC0400009001.hg.1	FAM160A1	Multiple_Complex	5.64	3.59	4.15	1.70E-07	4.42E-06
TC0400012078.hg.1	NR3C2	Multiple_Complex	5.68	3.63	4.15	6.34E-09	2.68E-07
TC1900008993.hg.1	ZNF304	Multiple_Complex	5.63	3.57	4.15	1.30E-07	3.55E-06
TC1900006652.hg.1	TJP3	Multiple_Complex	6.87	4.82	4.14	2.02E-05	0.0002
TC0800010002.hg.1	DUSP4	Multiple_Complex	11.1	9.05	4.14	4.12E-09	1.86E-07
TC0100017167.hg.1	LAMB3	Multiple_Complex	9.15	7.1	4.13	1.89E-09	9.78E-08
TC0700013531.hg.1	HOXA6	Multiple_Complex	9.13	7.09	4.12	3.28E-07	7.60E-06
TC0600009606.hg.1	ARFGEF3	Multiple_Complex	8.14	6.1	4.11	1.46E-08	5.43E-07
TC1900008141.hg.1	CYP2S1	Multiple_Complex	11.19	9.15	4.11	1.16E-07	3.23E-06
TC0900007552.hg.1	GDA	Multiple_Complex	7.82	5.78	4.1	2.12E-06	3.65E-05
TC0200007821.hg.1	SLC1A4	Multiple_Complex	7.86	5.83	4.1	2.67E-08	9.03E-07
TSUnmapped00000083.hg.1	BCL2L14	NonCoding	5.34	3.31	4.09	5.52E-07	1.17E-05
TC2100008521.hg.1	B3GALT5	Multiple_Complex	7.89	5.86	4.09	2.50E-09	1.23E-07
TC0800008667.hg.1	NOV	Multiple_Complex	7.59	5.56	4.09	3.38E-06	5.41E-05
TC0100015265.hg.1	DENND2D	Multiple_Complex	7.47	5.44	4.08	6.71E-08	2.02E-06
TC2000009317.hg.1	SULF2	Multiple_Complex	10.8	8.78	4.08	1.76E-09	9.19E-08
TC0100015194.hg.1	SORT1	Multiple_Complex	10.48	8.45	4.08	2.37E-06	4.02E-05
TC0100013314.hg.1	IL22RA1	Multiple_Complex	8.1	6.08	4.06	1.85E-07	4.72E-06
TC0500011470.hg.1	FAM172A	Multiple_Complex	6.47	4.45	4.05	3.84E-06	6.04E-05
TC0200009619.hg.1	LYPD6B	Multiple_Complex	7.89	5.87	4.05	1.36E-06	2.50E-05
TC1500010783.hg.1	SEMA4B	Multiple_Complex	9.27	7.25	4.04	1.09E-07	3.07E-06
TC0400012952.hg.1	BDH2	Multiple_Complex	8.14	6.13	4.02	4.23E-08	1.36E-06
TC0900010607.hg.1	AGTPBP1	Multiple_Complex	7.72	5.71	4.02	6.86E-09	2.87E-07
TC0300007517.hg.1	CACNA1D	Multiple_Complex	7.33	5.33	4	3.21E-07	7.46E-06
TC1000010446.hg.1	ZNF239	Multiple_Complex	6.2	4.2	3.99	9.82E-09	3.88E-07

TC0800012322.hg.1	CA13	NonCoding	4.85	2.86	3.99	9.48E-09	3.80E-07
TC1600007147.hg.1	TMEM159	Multiple_Complex	7.71	5.71	3.98	3.70E-08	1.20E-06
TC0300010664.hg.1	GLB1	Multiple_Complex	10.28	8.29	3.98	1.92E-07	4.90E-06
TC0800008263.hg.1	ESRP1	Multiple_Complex	10.54	8.56	3.97	4.27E-09	1.92E-07
TC0800010558.hg.1	TOX	Multiple_Complex	7.06	5.08	3.96	4.51E-09	1.99E-07
TC1000011485.hg.1	PDLIM1	Multiple_Complex	9.49	7.5	3.96	3.08E-09	1.46E-07
TC0800012206.hg.1	DGAT1	Multiple_Complex	10.23	8.25	3.95	1.25E-08	4.79E-07
TC1900010851.hg.1	ETHE1	Multiple_Complex	9.21	7.23	3.94	1.08E-07	3.05E-06
TC1500010725.hg.1	CAPN3	Multiple_Complex	5.6	3.63	3.92	1.72E-07	4.46E-06
TC1900009076.hg.1	PLPP2	Multiple_Complex	10.74	8.77	3.9	6.95E-09	2.90E-07
TC1900008470.hg.1	GRIN2D	Multiple_Complex	7.69	5.73	3.9	1.62E-08	5.91E-07
TC0100007361.hg.1	GRHL3	Multiple_Complex	7.6	5.64	3.9	7.32E-09	3.01E-07
TC0900008312.hg.1	SLC44A1	Multiple_Complex	10.11	8.15	3.87	2.41E-08	8.35E-07
TC1300008892.hg.1	LPAR6	Multiple_Complex	6.35	4.4	3.87	4.19E-07	9.24E-06
TC0900007807.hg.1	DAPK1	Multiple_Complex	9.6	7.65	3.86	1.54E-08	5.68E-07
TC0600009626.hg.1	ABRACL	Multiple_Complex	8.93	6.98	3.85	2.54E-07	6.12E-06
TC1700012216.hg.1	LGALS9	Multiple_Complex	8.16	6.22	3.85	4.09E-08	1.31E-06
TC1900007012.hg.1	LDLR	Multiple_Complex	10.7	8.76	3.84	7.05E-06	0.0001
TC1700012110.hg.1	SECTM1	Multiple_Complex	7.57	5.63	3.83	1.80E-07	4.65E-06
TC0200016403.hg.1	LPIN1	Multiple_Complex	8.59	6.66	3.82	1.06E-08	4.13E-07
TC1300007135.hg.1	ITM2B	Multiple_Complex	10.97	9.04	3.8	1.15E-08	4.44E-07
TC0600014361.hg.1	CNKSR3	Multiple_Complex	7.1	5.17	3.8	4.71E-07	1.02E-05
TC1100009504.hg.1	BARX2	Multiple_Complex	6.97	5.04	3.8	1.53E-08	5.66E-07
TC2100008250.hg.1	TFF2	Multiple_Complex	8.05	6.13	3.79	5.94E-06	8.73E-05
TC0600011507.hg.1	PSMB8	Multiple_Complex	9.05	7.12	3.79	2.06E-08	7.29E-07
TC0500013248.hg.1	PCDHB15	Coding	6.5	4.58	3.78	2.34E-07	5.71E-06
TC1700010200.hg.1	ALDOC	Multiple_Complex	9.02	7.1	3.77	9.60E-09	3.83E-07
TC0800008371.hg.1	SPAG1	Multiple_Complex	7.71	5.8	3.76	1.71E-08	6.15E-07
TC1900008755.hg.1	ZNF331	Multiple_Complex	7.51	5.6	3.75	5.47E-08	1.68E-06
TC0100009195.hg.1	SLC35A3	Multiple_Complex	10.77	8.86	3.74	3.23E-08	1.07E-06
TC1100010030.hg.1	STK33	Multiple_Complex	7.15	5.25	3.73	4.08E-06	6.35E-05
TC0100008773.hg.1	MSH4	Multiple_Complex	4.77	2.88	3.71	2.79E-08	9.40E-07
TC1400007495.hg.1	PLEKHH1	Multiple_Complex	8.77	6.88	3.71	2.49E-08	8.57E-07
TC1000012011.hg.1	RGS10	Multiple_Complex	8.16	6.28	3.7	2.07E-07	5.17E-06
TC0400008345.hg.1	SGMS2	Multiple_Complex	7.77	5.88	3.7	1.16E-05	0.0002
TC0400012922.hg.1	TLR6	Multiple_Complex	6.45	4.57	3.68	3.48E-08	1.14E-06
TC0700010257.hg.1	ICA1	Multiple_Complex	8.96	7.08	3.68	2.36E-08	8.23E-07
TC0Y00006444.hg.1	CSF2RA	Multiple_Complex	5.82	3.94	3.68	7.49E-08	2.22E-06
TC1100009453.hg.1	ST3GAL4	Multiple_Complex	9.45	7.57	3.67	1.23E-08	4.72E-07
TC0600009333.hg.1	SMPDL3A	Multiple_Complex	7.75	5.87	3.67	9.77E-09	3.88E-07

TC0300008361.hg.1	SIDT1	Multiple_Complex	7.54	5.67	3.67	1.20E-06	2.25E-05
TC0600010210.hg.1	UNC93A	Multiple_Complex	7.69	5.81	3.67	2.61E-07	6.27E-06
TC0800007114.hg.1	SCARA3	Multiple_Complex	6.12	4.24	3.66	1.78E-06	3.15E-05
TC1500008003.hg.1	TMED3	Multiple_Complex	8.62	6.75	3.66	2.95E-07	6.94E-06
TC0700013443.hg.1	CFTR	Multiple_Complex	5.67	3.8	3.66	1.49E-08	5.54E-07
TC0300006622.hg.1	PPARG	Multiple_Complex	10.39	8.52	3.66	1.00E-08	3.92E-07
TC1500010862.hg.1	ELL3	Multiple_Complex	9.07	7.2	3.65	2.18E-06	3.74E-05
TC0100007370.hg.1	RCAN3	Multiple_Complex	8.28	6.42	3.64	1.03E-08	4.03E-07
TC1900008057.hg.1	ZFP36	Coding	8.91	7.05	3.63	1.33E-06	2.46E-05
TC1900006804.hg.1	TNFSF9	Multiple_Complex	9.66	7.81	3.62	1.01E-06	1.95E-05
TC0600014192.hg.1	RP11-307P5.1	NonCoding	4.92	3.06	3.62	3.51E-08	1.15E-06
TC0100008033.hg.1	ERMAP	Multiple_Complex	7.14	5.28	3.62	4.47E-08	1.41E-06
TC0400010256.hg.1	CCDC149	Multiple_Complex	5.95	4.1	3.62	1.83E-08	6.53E-07
TC0700012886.hg.1	EPHA1	Multiple_Complex	6.42	4.56	3.61	1.68E-08	6.06E-07
TC0300013334.hg.1	MCCC1	Multiple_Complex	9.39	7.55	3.6	8.54E-08	2.49E-06
TC1500006972.hg.1	SPINT1	Multiple_Complex	9.82	7.98	3.6	1.48E-08	5.50E-07
TC1200012863.hg.1	CCDC92	NonCoding	7.34	5.5	3.6	5.04E-08	1.57E-06
TC2100007056.hg.1	SIM2	Multiple_Complex	7.98	6.14	3.59	1.36E-06	2.50E-05
TC0400008137.hg.1	CCSER1	Multiple_Complex	5.12	3.28	3.59	3.35E-08	1.10E-06
TC0300011023.hg.1	SHISA5	Multiple_Complex	11.61	9.77	3.58	3.19E-08	1.06E-06
TC0100015598.hg.1	TXNIP	Multiple_Complex	11.32	9.48	3.57	1.24E-07	3.40E-06
TC0300011936.hg.1	CD47	Multiple_Complex	9.5	7.67	3.56	4.80E-08	1.50E-06
TC0700012016.hg.1	EPHB4	Multiple_Complex	8.68	6.85	3.56	1.18E-08	4.55E-07
TC0600013179.hg.1	OR2A4	Coding	6.58	4.75	3.54	3.81E-07	8.56E-06
TC1900009431.hg.1	SLC25A23	Multiple_Complex	8.59	6.76	3.54	9.57E-09	3.82E-07
TC1400010763.hg.1	TMEM229B	Coding	7.21	5.39	3.54	2.43E-06	4.10E-05
TC0900006758.hg.1	DENND4C	Multiple_Complex	9.1	7.27	3.54	3.80E-08	1.23E-06
TC1600008712.hg.1	IRF8	Multiple_Complex	6.6	4.78	3.53	1.98E-07	5.01E-06
TC1300008660.hg.1	PROSER1	Multiple_Complex	10.14	8.32	3.53	3.23E-07	7.50E-06
TC0900008148.hg.1	TDRD7	Multiple_Complex	8.06	6.25	3.52	1.37E-06	2.52E-05
TC1700010686.hg.1	JUP	Multiple_Complex	9.43	7.61	3.52	8.72E-07	1.73E-05
TC0800011579.hg.1	EXT1	Multiple_Complex	9.54	7.73	3.49	2.60E-08	8.89E-07
TC1000006802.hg.1	CAMK1D	Multiple_Complex	7.01	5.21	3.49	2.19E-07	5.42E-06
TC1900008544.hg.1	RCN3	Multiple_Complex	8.16	6.36	3.49	2.51E-08	8.61E-07
TC0X00011382.hg.1	ACSL4	Multiple_Complex	9.85	8.05	3.49	1.47E-07	3.92E-06
TC1300010040.hg.1	NEK5	Multiple_Complex	6.85	5.05	3.49	7.27E-07	1.48E-05
TC0900007492.hg.1	PIP5K1B	Unassigned	5.58	3.78	3.48	1.30E-06	2.40E-05
TC0400011440.hg.1	PPP3CA	Multiple_Complex	10.35	8.56	3.47	4.65E-08	1.46E-06
TC0500012294.hg.1	PCDH1	Multiple_Complex	7.74	5.95	3.47	5.22E-07	1.12E-05
TC0300013471.hg.1	BCL6	Multiple_Complex	10.21	8.42	3.47	2.02E-07	5.09E-06

TC0400008813.hg.1	MGST2	Multiple_Complex	8.12	6.32	3.47	1.90E-07	4.85E-06
TC1800007014.hg.1	DSG2	Multiple_Complex	10.57	8.78	3.46	3.87E-08	1.25E-06
TC0100013636.hg.1	RNF19B	Multiple_Complex	8.67	6.88	3.45	2.22E-07	5.47E-06
TC0400011180.hg.1	SCD5	Coding	8.93	7.15	3.44	6.62E-08	1.99E-06
TC0200009470.hg.1	HNMT	Multiple_Complex	6.91	5.13	3.44	1.71E-08	6.15E-07
TSUnmapped00000243.hg.1	MANSC1	Coding	9.5	7.72	3.43	5.47E-08	1.68E-06
TC1900012056.hg.1	ZNF772	Multiple_Complex	5.51	3.73	3.43	8.56E-05	0.0008
TC0500011875.hg.1	PPIC	Multiple_Complex	8.66	6.89	3.43	2.69E-07	6.46E-06
TC0100008816.hg.1	IFI44	Multiple_Complex	5.93	4.16	3.42	6.86E-08	2.06E-06
TC0200010445.hg.1	CASP10	Multiple_Complex	5.38	3.61	3.42	5.91E-08	1.79E-06
TC1200012602.hg.1	SLCO1B7	Multiple_Complex	4.79	3.02	3.41	1.37E-07	3.68E-06
TC0800008184.hg.1	DECR1	Multiple_Complex	9.45	7.68	3.41	2.00E-07	5.04E-06
TC0400007556.hg.1	SRD5A3	Multiple_Complex	9.78	8.03	3.38	7.21E-07	1.47E-05
TSUnmapped00000248.hg.1	BCL2L14	Coding	6.48	4.72	3.38	5.87E-07	1.23E-05
TC0800012026.hg.1	SLC45A4	Multiple_Complex	7.58	5.82	3.38	5.53E-06	8.23E-05
TC1300009394.hg.1	SLITRK6	Coding	5.86	4.12	3.35	1.63E-08	5.92E-07
TC0600008569.hg.1	MYO6	Multiple_Complex	8.24	6.49	3.35	1.27E-07	3.48E-06
TC0200011449.hg.1	SH3YL1	Multiple_Complex	6.95	5.21	3.34	1.03E-07	2.93E-06
TC1200007037.hg.1	PDE3A	Multiple_Complex	5.89	4.15	3.33	1.09E-07	3.07E-06
TC1300009271.hg.1	TBC1D4	Multiple_Complex	7.74	6	3.33	6.50E-07	1.35E-05
TC1000007226.hg.1	CCDC7	Multiple_Complex	6.45	4.72	3.32	4.35E-06	6.70E-05
TC0100016366.hg.1	KIFAP3	Multiple_Complex	8.97	7.24	3.32	1.57E-07	4.15E-06
TC1400009141.hg.1	NIN	Multiple_Complex	9.34	7.61	3.31	2.36E-06	4.02E-05
TC1300008573.hg.1	STARD13	Multiple_Complex	6.4	4.68	3.31	9.06E-07	1.78E-05
TC0100008417.hg.1	PCSK9	Multiple_Complex	8.22	6.5	3.31	1.95E-05	0.0002
TC0700012636.hg.1	PODXL	Multiple_Complex	9.63	7.91	3.31	2.83E-08	9.50E-07
TC0300012236.hg.1	MUC13	Multiple_Complex	9.88	8.15	3.3	5.85E-08	1.78E-06
TC0800011683.hg.1	FBXO32	Multiple_Complex	7.47	5.75	3.3	1.88E-06	3.29E-05
TC0600009459.hg.1	ENPP1	Multiple_Complex	7.69	5.97	3.29	2.45E-08	8.47E-07
TC1500007196.hg.1	SLC27A2	Multiple_Complex	9.63	7.91	3.29	2.03E-07	5.10E-06
TC1300006989.hg.1	AKAP11	Multiple_Complex	8.81	7.09	3.29	4.42E-06	6.77E-05
TC0200010745.hg.1	IGFBP2	Multiple_Complex	8.56	6.85	3.28	4.19E-08	1.34E-06
TC0X00006709.hg.1	NHS	Multiple_Complex	7.09	5.39	3.27	6.67E-07	1.38E-05
TC0400008483.hg.1	UGT8	Multiple_Complex	9.23	7.52	3.27	2.45E-07	5.94E-06
TC0300010521.hg.1	THRB	Multiple_Complex	6.76	5.05	3.27	9.49E-07	1.85E-05
TC1900011281.hg.1	ZNF615	Multiple_Complex	6.81	5.11	3.26	2.42E-07	5.88E-06
TC0100011203.hg.1	RNPEP	Multiple_Complex	10.34	8.64	3.26	7.93E-06	0.0001
TC0200007200.hg.1	LTBP1	Multiple_Complex	7.79	6.09	3.25	8.34E-07	1.66E-05
TC1300008487.hg.1	SLC7A1	Multiple_Complex	10.47	8.77	3.25	2.31E-07	5.65E-06
TC0800012312.hg.1	SGK3	Multiple_Complex	7.46	5.76	3.25	8.50E-08	2.48E-06

TC1600009105.hg.1	ABCA3	Multiple_Complex	8.61	6.91	3.25	1.69E-06	3.01E-05
TC0100018302.hg.1	EFNA4	Multiple_Complex	7.16	5.46	3.24	0.0002	0.0013
TC0100018479.hg.1	NOTCH2NL	Multiple_Complex	9.4	7.71	3.24	3.38E-06	5.41E-05
TC0500013381.hg.1	PRELID2	Multiple_Complex	7.98	6.29	3.24	2.42E-07	5.87E-06
TC1100007943.hg.1	PLCB3	Multiple_Complex	9.17	7.48	3.24	2.37E-08	8.23E-07
TC1900007061.hg.1	ZNF844	Coding	6.95	5.25	3.24	1.45E-07	3.88E-06
TC0500012642.hg.1	RNF145	Multiple_Complex	8.57	6.88	3.23	2.23E-07	5.48E-06
TC1900006977.hg.1	ICAM1	Multiple_Complex	8.65	6.96	3.23	6.43E-05	0.0007
TC1600010006.hg.1	PRSS8	Multiple_Complex	8.31	6.62	3.23	6.29E-06	9.17E-05
TC0500013219.hg.1	HSD17B4	Multiple_Complex	9.91	8.22	3.23	1.26E-06	2.35E-05
TC1000006754.hg.1	CELF2	Multiple_Complex	7.5	5.81	3.23	4.06E-07	9.01E-06
TC0600013160.hg.1	SAMD3	Multiple_Complex	4.53	2.84	3.22	7.05E-07	1.45E-05
TC1200011591.hg.1	NTN4	Multiple_Complex	8.13	6.44	3.22	1.53E-05	0.0002
TC0800007102.hg.1	PTK2B	Multiple_Complex	7.48	5.79	3.22	1.11E-05	0.0001
TC1200009964.hg.1	MANSC1	Coding	9.69	8.01	3.21	7.11E-07	1.45E-05
TC0100015160.hg.1	SLC25A24	Multiple_Complex	9.36	7.69	3.19	1.87E-07	4.79E-06
TC0900006655.hg.1	LURAP1L	Multiple_Complex	7.98	6.3	3.19	3.12E-08	1.04E-06
TC0300011757.hg.1	PROS1	Multiple_Complex	7.26	5.59	3.19	4.24E-06	6.56E-05
TC0200007348.hg.1	PKDCC	Multiple_Complex	8.87	7.2	3.19	1.01E-07	2.87E-06
TC2200007672.hg.1	GRAMD4	Multiple_Complex	6.32	4.64	3.19	2.42E-07	5.87E-06
TC0400008119.hg.1	TIGD2	Multiple_Complex	6.55	4.88	3.19	1.58E-07	4.18E-06
TC1200008629.hg.1	DRAM1	Multiple_Complex	6.85	5.18	3.18	3.49E-06	5.54E-05
TC1700007127.hg.1	LGALS9C	Multiple_Complex	7.94	6.27	3.18	7.14E-07	1.46E-05
TC1400010012.hg.1	LGMN	Multiple_Complex	10.39	8.72	3.18	8.70E-07	1.72E-05
TC1400010071.hg.1	CLMN	Multiple_Complex	6.62	4.95	3.18	5.87E-08	1.79E-06
TC1000008585.hg.1	MARVELD1	Multiple_Complex	7.61	5.95	3.18	5.40E-08	1.67E-06
TC1100011833.hg.1	SYTL2	Multiple_Complex	7.88	6.22	3.17	1.24E-06	2.32E-05
TC0100008126.hg.1	AKR1A1	Multiple_Complex	8.92	7.26	3.17	4.30E-06	6.64E-05
TC1900011113.hg.1	GYS1	Multiple_Complex	9.37	7.71	3.16	3.58E-07	8.17E-06
TC0600010991.hg.1	MBOAT1	Multiple_Complex	9.3	7.64	3.15	1.81E-07	4.66E-06
TC1100012955.hg.1	TRIM34	Multiple_Complex	5.46	3.8	3.15	0.0003	0.0026
TC1500007977.hg.1	HYKK	Multiple_Complex	7.5	5.84	3.15	9.85E-06	0.0001
TC1700008263.hg.1	ABCC3	Multiple_Complex	8.11	6.46	3.15	1.07E-06	2.03E-05
TC0300012191.hg.1	PARP9	Multiple_Complex	8.39	6.74	3.14	1.30E-07	3.54E-06
TC1200006445.hg.1	B4GALNT3	Multiple_Complex	7.76	6.11	3.14	1.04E-06	1.98E-05
TC0900006473.hg.1	SMARCA2	Multiple_Complex	7.7	6.05	3.14	4.40E-06	6.75E-05
TC0800012313.hg.1	SGK3	Multiple_Complex	7.13	5.48	3.14	7.12E-08	2.12E-06
TC0500013247.hg.1	PCDHB14	Coding	8.29	6.64	3.13	1.45E-07	3.88E-06
TC0300006791.hg.1	KAT2B	Multiple_Complex	7.61	5.96	3.13	1.02E-06	1.96E-05
TC1200008933.hg.1	TPCN1	Multiple_Complex	9.45	7.8	3.13	9.32E-08	2.68E-06

TC0400010282.hg.1	SEL1L3	Multiple_Complex	10.55	8.9	3.13	4.43E-08	1.40E-06
TC0800006913.hg.1	SH2D4A	Multiple_Complex	8.8	7.16	3.12	1.57E-07	4.16E-06
TC0400011027.hg.1	BTC	Coding	6.42	4.78	3.11	4.15E-07	9.18E-06
TC1800007508.hg.1	TNFRSF11A	Unassigned	6.04	4.4	3.11	1.44E-06	2.63E-05
TC1300008688.hg.1	FOXO1	Multiple_Complex	6.78	5.14	3.11	5.98E-05	0.0006
TC0100010196.hg.1	NTRK1	Multiple_Complex	7.4	5.76	3.11	4.47E-08	1.41E-06
TC0900007077.hg.1	UNC13B	Multiple_Complex	9.29	7.66	3.11	7.79E-08	2.31E-06
TC0900010485.hg.1	GNAQ	Multiple_Complex	10	8.37	3.1	1.76E-07	4.55E-06
TC1400010390.hg.1	AHNAK2	Multiple_Complex	7.46	5.82	3.1	8.06E-08	2.38E-06
TC0600014318.hg.1	FAM46A	Coding	8.37	6.74	3.09	7.40E-07	1.50E-05
TC0500012238.hg.1	HBEGF	Multiple_Complex	7.84	6.21	3.09	2.52E-06	4.23E-05
TC1000011445.hg.1	MYOF	Multiple_Complex	10.4	8.78	3.09	1.98E-07	5.01E-06
TC0X00006955.hg.1	LANCL3	Coding	5.8	4.18	3.08	5.75E-06	8.50E-05
TC0200015364.hg.1	SATB2	Multiple_Complex	8.78	7.15	3.08	3.53E-08	1.15E-06
TC0100007845.hg.1	MANEAL	Coding	9.25	7.63	3.08	4.65E-08	1.46E-06
TC0100009558.hg.1	PTGFRN	Multiple_Complex	9.49	7.88	3.07	1.35E-07	3.64E-06
TC0600008571.hg.1	MYO6	Unassigned	6.32	4.7	3.07	0.0006	0.0043
TC0500010869.hg.1	ELOVL7	Multiple_Complex	7.65	6.04	3.07	2.02E-07	5.08E-06
TC0600013794.hg.1	AGPAT4	Multiple_Complex	6.03	4.42	3.06	1.93E-06	3.36E-05
TC1400009787.hg.1	SPTLC2	Multiple_Complex	10.52	8.9	3.06	7.92E-07	1.59E-05
TC0300013084.hg.1	LRRC31	Multiple_Complex	5.63	4.01	3.06	6.58E-08	1.98E-06
TC1900006968.hg.1	C19orf66	Multiple_Complex	6.83	5.22	3.06	7.12E-07	1.45E-05
TC0900007006.hg.1	PRSS3	Multiple_Complex	10.67	9.06	3.06	3.18E-07	7.41E-06
TC0400012874.hg.1	CYP4V2	Multiple_Complex	7.83	6.22	3.05	1.23E-05	0.0002
TC0800007185.hg.1	RBPMS	Multiple_Complex	9.54	7.94	3.04	1.36E-07	3.67E-06
TC1000009589.hg.1	PITRM1	Multiple_Complex	9.43	7.83	3.04	3.51E-07	8.02E-06
TC0700009807.hg.1	INSIG1	Multiple_Complex	8.31	6.71	3.03	4.45E-07	9.74E-06
TC1200008574.hg.1	APAF1	Multiple_Complex	8.38	6.78	3.03	2.26E-06	3.85E-05
TC0X00008228.hg.1	DOCK11	Multiple_Complex	7.49	5.9	3.03	3.92E-06	6.15E-05
TC0600014371.hg.1	RNASET2	Multiple_Complex	8.17	6.58	3.01	1.31E-06	2.42E-05
TC1200011470.hg.1	DUSP6	Multiple_Complex	10.64	9.05	3.01	4.72E-08	1.48E-06
TC0100016296.hg.1	GPA33	Multiple_Complex	7.15	5.57	3	1.36E-05	0.0002
TC2200008482.hg.1	EIF4ENIF1	Multiple_Complex	8.21	6.62	3	1.07E-06	2.04E-05
TC0100016963.hg.1	KDM5B	Multiple_Complex	9.14	7.56	3	1.11E-05	0.0001
TC0100012089.hg.1	GPR137B	Multiple_Complex	7.34	5.76	3	1.87E-07	4.77E-06
TC1200006730.hg.1	RIMKLB	Multiple_Complex	7.51	5.93	3	1.94E-07	4.94E-06
TC0900006762.hg.1	ACER2	Multiple_Complex	6.76	5.18	2.99	5.25E-05	0.0006
TC1300009622.hg.1	ZIC5	Coding	7.63	6.05	2.99	3.36E-06	5.38E-05
TC0100011022.hg.1	RGS2	Multiple_Complex	8.78	7.2	2.98	1.36E-07	3.68E-06
TC0100014191.hg.1	RAB3B	Multiple_Complex	6.33	4.75	2.98	4.73E-07	1.03E-05

TC1800007080.hg.1	GALNT1		Multiple_Complex	9.18	7.61	2.98	1.82E-06	3.20E-05
TC1400010776.hg.1	CATSPERB		Multiple_Complex	6.11	4.54	2.97	4.14E-07	9.16E-06
TC1200011557.hg.1	CEP83		Multiple_Complex	6.85	5.28	2.97	3.45E-05	0.0004
TC1500010858.hg.1	CATSPER2		Multiple_Complex	5.37	3.8	2.96	6.37E-05	0.0006
TC0100008618.hg.1	AK4		Multiple_Complex	9.1	7.53	2.96	1.16E-06	2.19E-05
TC0600013165.hg.1	EPB41L2		Multiple_Complex	10.36	8.8	2.95	5.75E-07	1.21E-05
TSUnmapped00000589.hg.1	BCL2L14		Coding	6.76	5.2	2.95	5.98E-08	1.81E-06
TC1400006718.hg.1	PSME1		Multiple_Complex	9.51	7.95	2.95	1.44E-05	0.0002
TC1800008331.hg.1	SS18		Multiple_Complex	9.97	8.41	2.95	2.80E-07	6.65E-06
TC0800009239.hg.1	CYC1		Multiple_Complex	10.97	9.42	2.94	4.12E-07	9.12E-06
TC1500010723.hg.1	CHAC1		Multiple_Complex	10.11	8.56	2.94	1.32E-05	0.0002
TC0500011935.hg.1		mar-03	Multiple_Complex	8.58	7.02	2.94	1.81E-07	4.66E-06
TC1900011469.hg.1	TMEM238		Multiple_Complex	8.94	7.39	2.94	0.0011	0.0068
TC1100006760.hg.1	PPFIBP2		Multiple_Complex	8.07	6.52	2.94	1.39E-05	0.0002
TC1600011060.hg.1	COTL1		Multiple_Complex	10.54	8.99	2.93	5.76E-06	8.50E-05
TC1100007899.hg.1	RARRES3		Multiple_Complex	7.93	6.38	2.93	1.70E-06	3.01E-05
TC1200012206.hg.1	KDM2B		Multiple_Complex	8.05	6.5	2.93	3.31E-07	7.62E-06
TC0500013245.hg.1	PCDHB9		Multiple_Complex	7.53	5.98	2.93	3.29E-07	7.61E-06
TC0600008757.hg.1	PNRC1		Multiple_Complex	9.11	7.56	2.92	0.0004	0.0031
TC1100011126.hg.1	PLA2G16		Multiple_Complex	10.39	8.85	2.92	1.09E-05	0.0001
TC1200006850.hg.1	ETV6		Multiple_Complex	9.33	7.79	2.92	1.27E-06	2.37E-05
TC1200012181.hg.1	SPPL3		Multiple_Complex	8.89	7.35	2.92	5.10E-07	1.10E-05
TC0100017733.hg.1	TSNAX		NonCoding	4.83	3.29	2.91	3.12E-05	0.0004
TC0500009076.hg.1	SLC26A2		Multiple_Complex	6.68	5.14	2.91	1.94E-05	0.0002
TC0100008536.hg.1	INADL		Multiple_Complex	8.59	7.05	2.91	1.73E-07	4.50E-06
TC0100015401.hg.1	IGSF3		Multiple_Complex	7.18	5.64	2.91	1.52E-07	4.04E-06
TC0500007881.hg.1	JMY		Multiple_Complex	8.59	7.06	2.9	6.48E-06	9.39E-05
TC1500010749.hg.1	FAM81A		Multiple_Complex	6.8	5.27	2.9	7.32E-06	0.0001
TC0300010282.hg.1	TIMP4		Multiple_Complex	7.73	6.2	2.9	5.48E-07	1.16E-05
TC0700013500.hg.1	ADAP1		Multiple_Complex	9.52	7.99	2.9	2.86E-07	6.79E-06
TC1400006943.hg.1	MIPOL1		Multiple_Complex	4.3	2.77	2.89	2.86E-06	4.74E-05
TC0300011899.hg.1	CBLB		Multiple_Complex	8.18	6.65	2.89	1.87E-06	3.28E-05
TC0900012141.hg.1	GCNT1		Multiple_Complex	7.3	5.77	2.89	0.0003	0.0026
TC1900009754.hg.1	DNASE2		Multiple_Complex	9.01	7.48	2.89	2.83E-06	4.70E-05
TC1600009943.hg.1	SEZ6L2		Multiple_Complex	7.11	5.58	2.89	7.35E-06	0.0001
TC1200010795.hg.1	RARG		Multiple_Complex	7.43	5.9	2.89	1.94E-07	4.94E-06
TC0500007834.hg.1	F2RL1		Multiple_Complex	9.52	7.99	2.88	2.85E-07	6.76E-06
TC0600011817.hg.1	C6orf132		Multiple_Complex	7.89	6.36	2.88	0.0001	0.0011
TC0400010500.hg.1	RBM47		Multiple_Complex	9.96	8.44	2.88	4.05E-06	6.32E-05
TC0700008367.hg.1	PEG10		Multiple_Complex	8.79	7.27	2.88	3.01E-07	7.06E-06

TC1200011471.hg.1	POC1B	Multiple_Complex	9.14	7.62	2.88	1.62E-07	4.24E-06
TC0800011064.hg.1	CALB1	Multiple_Complex	10.29	8.76	2.88	1.05E-07	2.96E-06
TC0300013838.hg.1	ABHD14A	Multiple_Complex	7.46	5.94	2.87	2.12E-05	0.0003
TC1400009980.hg.1	CCDC88C	Multiple_Complex	8.38	6.86	2.87	7.93E-06	0.0001
TC1500008043.hg.1	ABHD17C	Multiple_Complex	9.77	8.25	2.87	4.34E-07	9.54E-06
TC0200011138.hg.1	UGT1A1	Multiple_Complex	6.01	4.48	2.87	9.27E-07	1.81E-05
TC0600011953.hg.1	PLA2G7	Multiple_Complex	5.03	3.5	2.87	1.76E-07	4.55E-06
TC1900007384.hg.1	GDF15	Multiple_Complex	11.41	9.88	2.87	6.20E-05	0.0006
TC0500007154.hg.1	SLC1A3	Multiple_Complex	6.73	5.21	2.87	6.30E-07	1.31E-05
TC1900008113.hg.1	LTBP4	Multiple_Complex	6.94	5.42	2.87	2.55E-05	0.0003
TC0100018285.hg.1	NBPF19	Coding	7.96	6.44	2.86	7.73E-07	1.56E-05
TC1100009657.hg.1	IFITM3	Multiple_Complex	11.19	9.68	2.86	0.0002	0.0016
TC2100007140.hg.1	ETS2	Multiple_Complex	11.47	9.95	2.86	2.05E-07	5.13E-06
TC0100015483.hg.1	NOTCH2	Multiple_Complex	9.44	7.93	2.86	6.67E-07	1.38E-05
TSUnmapped00000486.hg.1	MLXIP	Coding	9.21	7.7	2.86	1.01E-06	1.94E-05
TC0200008663.hg.1	IL1R2	Multiple_Complex	8.55	7.04	2.85	9.69E-07	1.88E-05
TC1200008920.hg.1	OAS3	Multiple_Complex	9.03	7.52	2.85	2.77E-06	4.61E-05
TC0900008197.hg.1	GALNT12	Multiple_Complex	8.15	6.64	2.85	1.50E-07	4.00E-06
TC1500007463.hg.1	C2CD4A	Coding	7.2	5.69	2.85	7.19E-06	0.0001
TC0100014276.hg.1	DHCR24	Multiple_Complex	10.43	8.92	2.85	3.66E-07	8.28E-06
TC1700010590.hg.1	GSDMB	Multiple_Complex	7.82	6.32	2.85	1.29E-06	2.40E-05
TC1300007137.hg.1	RB1	Multiple_Complex	10.25	8.74	2.84	3.31E-07	7.62E-06
TC0100010755.hg.1	RALGPS2	Multiple_Complex	7.34	5.84	2.84	6.15E-06	8.98E-05
TC1500007520.hg.1	APH1B	Multiple_Complex	7.06	5.55	2.84	4.72E-07	1.03E-05
TC0100013153.hg.1	TMCO4	Multiple_Complex	7.61	6.1	2.83	1.74E-06	3.08E-05
TC1900007844.hg.1	LSR	Multiple_Complex	10.42	8.92	2.83	2.97E-05	0.0003
TC0900011257.hg.1	WDR31	Multiple_Complex	6.78	5.28	2.83	4.63E-07	1.01E-05
TC0800009158.hg.1	THEM6	Multiple_Complex	8.4	6.9	2.82	5.20E-06	7.80E-05
TC0100012816.hg.1	CLSTN1	Multiple_Complex	10.26	8.77	2.82	3.33E-07	7.66E-06
TSUnmapped00000103.hg.1	MLXIP	NonCoding	10.24	8.75	2.82	1.82E-07	4.68E-06
TC1100011614.hg.1	KCNE3	Coding	8.23	6.73	2.82	8.24E-07	1.64E-05
TC1400010774.hg.1	FOXN3	Multiple_Complex	6.06	4.57	2.81	7.20E-07	1.47E-05
TC0100007747.hg.1	GJB3	Multiple_Complex	7.36	5.87	2.8	1.87E-06	3.28E-05
TC1000010273.hg.1	NRP1	Multiple_Complex	8.81	7.32	2.8	5.51E-06	8.21E-05
TC0400010058.hg.1	HS3ST1	Coding	7.98	6.49	2.8	5.07E-05	0.0005
TC0900006736.hg.1	CNTLN	Multiple_Complex	7.76	6.28	2.8	9.51E-07	1.85E-05
TC1400010015.hg.1	ITPK1	Multiple_Complex	10.16	8.68	2.8	2.94E-07	6.94E-06
TC2000007817.hg.1	FAM210B	Multiple_Complex	8.2	6.72	2.8	1.33E-05	0.0002
TC0500008994.hg.1	SH3RF2	Multiple_Complex	7.3	5.82	2.8	3.14E-07	7.33E-06
TC1000008388.hg.1	FAS	Multiple_Complex	7.43	5.95	2.79	1.37E-05	0.0002

TC0100014009.hg.1	TESK2	Multiple_Complex	5.94	4.46	2.78	7.85E-07	1.57E-05
TC1900011915.hg.1	ZNF737	Multiple_Complex	9.4	7.93	2.78	1.05E-05	0.0001
TC0500009706.hg.1	SQSTM1	Multiple_Complex	11.43	9.95	2.78	1.40E-05	0.0002
TC1600011359.hg.1	ITPRIPL2	Multiple_Complex	6.1	4.62	2.78	7.41E-07	1.50E-05
TC0X00010851.hg.1	GPC3	Multiple_Complex	5.77	4.3	2.77	2.03E-07	5.10E-06
TC1200007816.hg.1	DGKA	Multiple_Complex	5.92	4.46	2.77	3.90E-06	6.12E-05
TC0100015822.hg.1	S100A11	Multiple_Complex	12.42	10.95	2.76	6.29E-06	9.17E-05
TC0400011538.hg.1	PAPSS1	Multiple_Complex	8.96	7.49	2.76	6.39E-06	9.29E-05
TC0X00011339.hg.1	ARSE	Multiple_Complex	5.96	4.49	2.76	7.22E-06	0.0001
TC2100008314.hg.1	CSTB	Multiple_Complex	11.45	9.99	2.76	7.42E-07	1.50E-05
TC0900011120.hg.1	KLF4	Multiple_Complex	7.99	6.53	2.76	9.80E-07	1.89E-05
TC0100008145.hg.1	TSPAN1	Multiple_Complex	8.4	6.94	2.76	3.29E-07	7.61E-06
TC0100010672.hg.1	RABGAP1L	Multiple_Complex	8.36	6.9	2.75	2.62E-06	4.38E-05
TC0600008120.hg.1	TMEM63B	Multiple_Complex	8.23	6.77	2.75	3.59E-07	8.17E-06
TC0400011650.hg.1	ARSJ	Multiple_Complex	8.31	6.85	2.75	4.97E-07	1.07E-05
TC0500010480.hg.1	PRLR	Multiple_Complex	5.9	4.44	2.75	7.05E-06	0.0001
TC0200008797.hg.1	SULT1C2	Multiple_Complex	7.59	6.13	2.75	6.31E-06	9.18E-05
TC0500009083.hg.1	CDX1	Multiple_Complex	7.53	6.07	2.75	3.05E-06	4.99E-05
TC0100017078.hg.1	RAB29	Multiple_Complex	8.25	6.8	2.74	1.52E-06	2.75E-05
TC0400007422.hg.1	ATP10D	Multiple_Complex	6.05	4.59	2.74	0.0001	0.0011
TC0100007469.hg.1	RPS6KA1	Multiple_Complex	9.63	8.17	2.74	6.53E-05	0.0007
TC0500011705.hg.1	NREP	Multiple_Complex	8.3	6.85	2.74	5.36E-07	1.14E-05
TC0800007119.hg.1	ESCO2	Multiple_Complex	8.81	7.36	2.74	4.32E-06	6.67E-05
TC0300010989.hg.1	CSPG5	Multiple_Complex	5.96	4.51	2.74	1.48E-07	3.95E-06
TC0700012439.hg.1	AASS	Multiple_Complex	6.9	5.45	2.74	3.80E-07	8.53E-06
TC1500007645.hg.1	MAP2K5	Multiple_Complex	6.71	5.26	2.73	5.48E-07	1.16E-05
TC0400012903.hg.1	PROM1	Multiple_Complex	10.3	8.86	2.73	3.61E-07	8.21E-06
TC1500010422.hg.1	IDH2	Multiple_Complex	9.45	8.01	2.72	9.25E-07	1.81E-05
TC1900011668.hg.1	ZNF763	Coding	6.94	5.49	2.72	1.36E-06	2.50E-05
TC0900011259.hg.1	HDHD3	Multiple_Complex	8.06	6.62	2.71	9.04E-06	0.0001
TC0100008591.hg.1	PGM1	Multiple_Complex	8.24	6.8	2.71	2.38E-07	5.80E-06
TC0300009789.hg.1	ST6GAL1	Multiple_Complex	8.02	6.58	2.71	0.0004	0.003
TC0300011485.hg.1	FOXP1	Multiple_Complex	9.58	8.14	2.71	3.87E-07	8.67E-06
TC1000009714.hg.1	PRKCQ	Multiple_Complex	6.93	5.5	2.71	4.63E-07	1.01E-05
TC0900008847.hg.1	LCN2	Multiple_Complex	6.46	5.03	2.7	0.0001	0.0011
TC0500007641.hg.1	PIK3R1	Multiple_Complex	9.92	8.48	2.7	1.32E-06	2.44E-05
TC0200007068.hg.1	KRTCAP3	Multiple_Complex	6.09	4.65	2.7	8.02E-06	0.0001
TC1800006937.hg.1	TAF4B	Multiple_Complex	7.78	6.34	2.7	3.65E-06	5.78E-05
TC1900007839.hg.1	FXYD5	Multiple_Complex	10.14	8.71	2.7	1.10E-06	2.08E-05
TC0X00007507.hg.1	EFNB1	Multiple_Complex	8.3	6.87	2.69	9.73E-07	1.88E-05

TC0100009341.hg.1	KIAA1324	Multiple_Complex	6.83	5.4	2.69	4.75E-06	7.22E-05
TC0200011251.hg.1	MLPH	Multiple_Complex	7.35	5.92	2.69	5.89E-06	8.67E-05
TC0100018356.hg.1	RHOU	NonCoding	7.78	6.36	2.69	5.29E-07	1.13E-05
TC1200012855.hg.1	VSIG10	Multiple_Complex	7.81	6.39	2.69	1.40E-05	0.0002
TC0100008607.hg.1	RAVER2	Multiple_Complex	9.58	8.16	2.68	2.38E-06	4.03E-05
TC1900007502.hg.1	ZNF85	Multiple_Complex	7.99	6.57	2.68	3.15E-07	7.35E-06
TC0600009364.hg.1	NCOA7	Multiple_Complex	8.69	7.27	2.68	2.32E-05	0.0003
TC1300009898.hg.1	GRTP1	Multiple_Complex	7.13	5.72	2.67	8.99E-07	1.77E-05
TC1300008760.hg.1	EPSTI1	Multiple_Complex	5.21	3.79	2.67	3.25E-07	7.53E-06
TC1400010714.hg.1	HAUS4	Multiple_Complex	6.66	5.24	2.67	3.70E-07	8.36E-06
TC1100010003.hg.1	CYB5R2	Multiple_Complex	8.67	7.26	2.66	7.61E-06	0.0001
TC0600013431.hg.1	PLAGL1	Multiple_Complex	6.73	5.32	2.66	1.46E-06	2.66E-05
TC0700010565.hg.1	HOXA11	Coding	7.93	6.52	2.65	8.32E-06	0.0001
TC0100010855.hg.1	NPL	Multiple_Complex	6.85	5.45	2.65	7.62E-06	0.0001
TC1000008795.hg.1	SLK	Multiple_Complex	11.2	9.79	2.65	1.97E-06	3.42E-05
TC0700009083.hg.1	AHCYL2	Multiple_Complex	8.39	6.99	2.65	6.88E-06	9.83E-05
TC0400010125.hg.1	FGFBP1	Multiple_Complex	6.23	4.83	2.64	4.54E-05	0.0005
TC1000012056.hg.1	ATE1	Multiple_Complex	9.5	8.1	2.64	8.30E-06	0.0001
TC0400011578.hg.1	CASP6	Multiple_Complex	8.19	6.79	2.64	9.07E-07	1.78E-05
TC0100015864.hg.1	S100A6	Multiple_Complex	10.88	9.48	2.64	8.21E-07	1.64E-05
TC1500009036.hg.1	MEIS2	Multiple_Complex	8.74	7.34	2.64	1.46E-06	2.65E-05
TC0900009912.hg.1	SIGMAR1	Multiple_Complex	11.42	10.03	2.63	0.0045	0.0223
TC0400012763.hg.1	S100P	Multiple_Complex	8.71	7.32	2.63	3.75E-07	8.46E-06
TC0100013305.hg.1	FUCA1	Coding	7.74	6.34	2.63	4.44E-07	9.73E-06
TC1100007262.hg.1	EHF	Multiple_Complex	11.6	10.21	2.63	6.07E-07	1.27E-05
TC0800008916.hg.1	EFR3A	Multiple_Complex	9.22	7.83	2.63	8.13E-07	1.62E-05
TC1900011791.hg.1	ZNF613	Multiple_Complex	8.43	7.04	2.62	2.92E-06	4.81E-05
TC1600009642.hg.1	GDE1	Multiple_Complex	9.74	8.35	2.62	3.93E-06	6.16E-05
TC0400008271.hg.1	NFKB1	Multiple_Complex	8.7	7.31	2.62	1.48E-06	2.68E-05
TC0600007847.hg.1	CDKN1A	Multiple_Complex	7.38	5.99	2.62	4.42E-05	0.0005
TC0800011061.hg.1	NBN	Multiple_Complex	9.1	7.71	2.62	1.24E-05	0.0002
TC1600011362.hg.1	ACSM3	Multiple_Complex	6.27	4.88	2.61	9.26E-06	0.0001
TC1800008234.hg.1	ABHD3	Multiple_Complex	8.36	6.97	2.61	1.76E-06	3.11E-05
TC0300012367.hg.1	KIAA1257	Multiple_Complex	6.59	5.21	2.61	2.24E-06	3.82E-05
TC1400008056.hg.1	IFI27	Multiple_Complex	7.13	5.74	2.61	7.82E-07	1.57E-05
TC0300008088.hg.1	CRYBG3	Multiple_Complex	7.12	5.74	2.61	1.33E-05	0.0002
TC0400007264.hg.1	KLF3	Multiple_Complex	10.2	8.82	2.6	3.89E-06	6.11E-05
TC0100018355.hg.1	RHOU	Multiple_Complex	7.82	6.44	2.6	4.25E-07	9.36E-06
TC0100008938.hg.1	LMO4	Multiple_Complex	9.11	7.72	2.6	5.09E-06	7.67E-05
TC0600008109.hg.1	VEGFA	Multiple_Complex	11.21	9.83	2.6	2.92E-07	6.89E-06

TC0900009825.hg.1	DDX58	Multiple_Complex	7.15	5.77	2.6	9.44E-07	1.84E-05
TC0500008646.hg.1	CCNI2	Multiple_Complex	7.17	5.79	2.6	2.67E-05	0.0003
TC0100016839.hg.1	DENND1B	Multiple_Complex	7.25	5.87	2.6	2.07E-05	0.0002
TC0600013326.hg.1	PERP	Coding	11.32	9.94	2.59	5.28E-06	7.91E-05
TC0200016494.hg.1	CNNM4	Multiple_Complex	8.98	7.61	2.59	3.97E-07	8.84E-06
TC0600008303.hg.1	LRRC1	Multiple_Complex	8.87	7.5	2.59	7.16E-07	1.46E-05
TC0600011059.hg.1	DCDC2	Coding	7.34	5.97	2.59	2.04E-07	5.11E-06
TC0400007857.hg.1	AREG	Multiple_Complex	7.85	6.48	2.58	5.77E-07	1.21E-05
TC0200016115.hg.1	ARL4C	Multiple_Complex	8.01	6.64	2.58	1.23E-06	2.30E-05
TC0400011299.hg.1	FAM13A	Multiple_Complex	7.3	5.93	2.58	8.13E-07	1.62E-05
TC0300007596.hg.1	FLNB	Multiple_Complex	8.94	7.58	2.58	6.36E-06	9.25E-05
TC0400012621.hg.1	ACSL1	Multiple_Complex	9.05	7.69	2.58	5.23E-07	1.12E-05
TC2000008318.hg.1	FERMT1	Multiple_Complex	11.2	9.83	2.58	1.92E-06	3.34E-05
TC0300007380.hg.1	TCTA	Multiple_Complex	8.64	7.27	2.58	0.0002	0.0016
TC0100008815.hg.1	IFI44L	Multiple_Complex	4.72	3.36	2.57	1.81E-06	3.20E-05
TC0100014910.hg.1	TGFBR3	Multiple_Complex	8.35	6.98	2.57	2.06E-06	3.56E-05
TC0700010604.hg.1	CPVL	Multiple_Complex	8.74	7.37	2.57	8.46E-07	1.68E-05
TC1600008301.hg.1	CALB2	Multiple_Complex	6.35	4.99	2.57	5.13E-07	1.10E-05
TC2000007283.hg.1	SRC	Multiple_Complex	8.8	7.44	2.57	3.28E-05	0.0004
TC0700006783.hg.1	TSPAN13	Multiple_Complex	10.44	9.08	2.57	2.67E-06	4.45E-05
TC1500009921.hg.1	UACA	Multiple_Complex	9.13	7.77	2.56	4.72E-05	0.0005
TC0200011878.hg.1	OSR1	Multiple_Complex	6.08	4.72	2.56	1.46E-05	0.0002
TC1900006520.hg.1	ATP5D	Multiple_Complex	9.75	8.39	2.56	0.0032	0.0168
TC0600009534.hg.1	MYB	Multiple_Complex	9.98	8.63	2.56	8.00E-07	1.60E-05
TC0300013846.hg.1	GXYLT2	Multiple_Complex	8.26	6.9	2.56	4.40E-06	6.75E-05
TC1900006991.hg.1	SLC44A2	Multiple_Complex	8.82	7.47	2.55	4.43E-05	0.0005
TC0800012363.hg.1	KHDRBS3	NonCoding	5.35	4	2.55	0.0007	0.0047
TC2000008587.hg.1	RALGAPA2	Multiple_Complex	8.91	7.56	2.55	2.20E-06	3.76E-05
TC2000009987.hg.1	OVOL2	Multiple_Complex	7.22	5.88	2.55	2.99E-06	4.91E-05
TC0800007738.hg.1	SDCBP	Multiple_Complex	10.99	9.64	2.54	3.62E-07	8.21E-06
TC0800007688.hg.1	LYN	Multiple_Complex	9.92	8.57	2.54	1.08E-06	2.05E-05
TC0600007231.hg.1	LRRC16A	Multiple_Complex	9.76	8.41	2.53	4.77E-07	1.03E-05
TC0400011087.hg.1	CCNI	Multiple_Complex	11.99	10.65	2.53	5.20E-07	1.11E-05
TC0400012606.hg.1	IRF2	Multiple_Complex	7.38	6.05	2.53	2.12E-05	0.0003
TC0400012933.hg.1	NAAA	Multiple_Complex	8.12	6.79	2.52	3.13E-06	5.08E-05
TC1500007318.hg.1	TEX9	Multiple_Complex	8.77	7.43	2.52	8.64E-06	0.0001
TC2100008256.hg.1	RSPH1	Multiple_Complex	5.83	4.5	2.52	6.66E-06	9.59E-05
TC1900010695.hg.1	CNTD2	Multiple_Complex	8.77	7.44	2.52	1.15E-05	0.0002
TC1900010538.hg.1	ZNF790	Coding	8.42	7.09	2.51	7.27E-06	0.0001
TC0700011503.hg.1	CLDN3	Coding	8.4	7.07	2.51	3.17E-05	0.0004

TC1600006870.hg.1	ATF7IP2	Multiple_Complex	7.1	5.77	2.51	1.81E-05	0.0002
TC1300009004.hg.1	ATP7B	Multiple_Complex	6.44	5.11	2.51	2.99E-06	4.91E-05
TC0X00010837.hg.1	MBNL3	Multiple_Complex	8.38	7.05	2.51	9.97E-07	1.92E-05
TC0800012160.hg.1	FAM83H	Multiple_Complex	9.08	7.75	2.51	6.27E-07	1.31E-05
TC0300007669.hg.1	SYNPR	Multiple_Complex	6.88	5.56	2.51	1.29E-06	2.40E-05
TC0400008380.hg.1	CCDC109B	Multiple_Complex	9.06	7.74	2.51	2.49E-06	4.19E-05
TC0900009855.hg.1	BAG1	Multiple_Complex	7.47	6.14	2.51	9.46E-06	0.0001
TC0300007055.hg.1	CTDSPL	Multiple_Complex	9.85	8.53	2.51	2.37E-06	4.02E-05
TC0300013380.hg.1	ALG3	Multiple_Complex	11.04	9.72	2.5	2.59E-06	4.33E-05
TC0400007311.hg.1	RHOH	Multiple_Complex	6.25	4.93	2.5	0.0004	0.0031
TC2000008722.hg.1	ACSS1	Multiple_Complex	8.88	7.56	2.5	1.57E-05	0.0002
TC1000009863.hg.1	FAM107B	Multiple_Complex	6.96	5.64	2.5	6.41E-06	9.31E-05
TC1300008280.hg.1	SKA3	Multiple_Complex	8.8	7.48	2.5	3.10E-06	5.05E-05
TC0700010558.hg.1	HOXA2	Multiple_Complex	6.54	5.22	2.5	5.06E-07	1.09E-05
TC0800009213.hg.1	FAM83H-AS1	NonCoding	9.46	8.14	2.49	1.55E-05	0.0002
TC0900008763.hg.1	PBX3	Multiple_Complex	7.01	5.69	2.49	4.14E-06	6.43E-05
TC1600008739.hg.1	FOXL1	Coding	6.91	5.6	2.49	5.33E-07	1.14E-05
TC1300008713.hg.1	KBTBD6	Multiple_Complex	6.72	5.4	2.49	9.72E-07	1.88E-05
TC1100010290.hg.1	E2F8	Multiple_Complex	8.42	7.1	2.49	4.96E-05	0.0005
TC1500006870.hg.1	C15orf41	Multiple_Complex	9.32	8.01	2.48	1.47E-06	2.67E-05
TC0900006538.hg.1	JAK2	Multiple_Complex	6.36	5.05	2.48	6.09E-07	1.27E-05
TC1200012083.hg.1	FBXO21	Multiple_Complex	9.12	7.81	2.48	1.01E-05	0.0001
TC1300008633.hg.1	SUPT20H	Multiple_Complex	10.33	9.02	2.48	1.31E-05	0.0002
TC1100007876.hg.1	TMEM179B	Multiple_Complex	11.44	10.13	2.48	0.0014	0.0084
TC1200008726.hg.1	TCP11L2	Multiple_Complex	8.4	7.1	2.47	7.38E-06	0.0001
TC1200009071.hg.1	PEBP1	Multiple_Complex	10.39	9.08	2.47	0.0004	0.0031
TC0700011797.hg.1	SAMD9L	Multiple_Complex	6.05	4.74	2.47	1.57E-05	0.0002
TC1300008873.hg.1	SUCLA2	Multiple_Complex	10.14	8.84	2.47	5.11E-06	7.69E-05
TC0400008609.hg.1	KIAA1109	Multiple_Complex	7.52	6.22	2.47	2.58E-05	0.0003
TC0800006873.hg.1	PDGFRL	Multiple_Complex	6.93	5.63	2.46	0.0015	0.009
TC1700010625.hg.1	TNS4	Multiple_Complex	10.46	9.16	2.46	5.57E-07	1.18E-05
TC1200010265.hg.1	CAPRIN2	Multiple_Complex	9.42	8.12	2.45	1.07E-05	0.0001
TC0100017368.hg.1	BPNT1	Multiple_Complex	10.13	8.84	2.45	8.73E-07	1.73E-05
TC1300006474.hg.1	MPHOSPH8	Multiple_Complex	6.61	5.32	2.45	0.0001	0.0011
TC1200012634.hg.1	KRT7	Multiple_Complex	9.69	8.4	2.45	1.64E-06	2.94E-05
TC0700011054.hg.1	GRB10	Multiple_Complex	7.64	6.35	2.45	5.71E-07	1.20E-05
TC1900006552.hg.1	ONECUT3	Coding	8.01	6.72	2.45	1.37E-05	0.0002
TC0300007161.hg.1	VIPR1	Multiple_Complex	7.58	6.29	2.45	1.82E-06	3.20E-05
TC0800011611.hg.1	TNFRSF11B	Multiple_Complex	6.7	5.41	2.44	0.0003	0.0024
TC1800006902.hg.1	TTC39C	Multiple_Complex	8.3	7.01	2.44	6.50E-07	1.35E-05

TC0100012490.hg.1	C1orf233	Multiple_Complex	7.61	6.32	2.44	3.08E-06	5.02E-05
TC0700012461.hg.1	WASL	Multiple_Complex	10.07	8.78	2.44	1.97E-06	3.42E-05
TC0400012956.hg.1	ELOVL6	Multiple_Complex	9.52	8.23	2.44	1.22E-06	2.29E-05
TC0100006884.hg.1	VPS13D	Multiple_Complex	7.37	6.09	2.44	8.34E-06	0.0001
TC0800009529.hg.1	PPP1R3B	Coding	7.14	5.85	2.44	2.03E-06	3.51E-05
TC1900007049.hg.1	ZNF440	Multiple_Complex	7.42	6.14	2.43	2.22E-05	0.0003
TC0300007647.hg.1	PTPRG	NonCoding	4.84	3.56	2.43	9.30E-07	1.82E-05
TC1900007688.hg.1	CCNE1	Multiple_Complex	7.84	6.56	2.43	5.46E-07	1.16E-05
TC1300008632.hg.1	ALG5	Multiple_Complex	6.83	5.56	2.42	1.06E-05	0.0001
TSUnmapped00000502.hg.1	MLXIP	NonCoding	9.92	8.65	2.42	1.90E-06	3.32E-05
TC1200010050.hg.1	LMO3	Multiple_Complex	7.01	5.74	2.42	6.04E-06	8.85E-05
TC0500013243.hg.1	PCDHB8	Coding	7.54	6.26	2.42	2.08E-06	3.59E-05
TC0800008715.hg.1	ZHX2	Coding	7.16	5.9	2.41	5.16E-06	7.76E-05
TC1100012949.hg.1	IFITM1	Multiple_Complex	9.12	7.85	2.41	0.0178	0.0659
TC1500010800.hg.1	ALDH1A3	Multiple_Complex	8.56	7.29	2.41	0.0001	0.001
TC0400010418.hg.1	RELL1	Multiple_Complex	8.62	7.35	2.4	7.71E-06	0.0001
TC0100017029.hg.1	PIK3C2B	Multiple_Complex	7.83	6.56	2.4	1.17E-06	2.21E-05
TC0100008017.hg.1	RIMKLA	Multiple_Complex	8.8	7.54	2.4	1.74E-05	0.0002
TC0700010728.hg.1	HERPUD2	Multiple_Complex	9.25	7.99	2.4	9.69E-06	0.0001
TC1000008904.hg.1	PDCD4	Multiple_Complex	9.4	8.14	2.4	1.78E-05	0.0002
TC0400012961.hg.1	NUDT6	Multiple_Complex	7.18	5.92	2.4	3.77E-05	0.0004
TC1100009016.hg.1	COLCA2	Coding	6.63	5.37	2.4	1.68E-06	3.00E-05
TC1000008400.hg.1	IFIT1	Coding	6.92	5.66	2.4	2.00E-05	0.0002
TC1300008386.hg.1	MTMR6	Multiple_Complex	8.66	7.4	2.39	3.57E-05	0.0004
TC1200012859.hg.1	RHOF	Multiple_Complex	9.73	8.48	2.39	0.0001	0.0013
TC1000007024.hg.1	KIAA1217	Multiple_Complex	7.42	6.17	2.39	2.92E-06	4.82E-05
TC0200010219.hg.1	FAM171B	Multiple_Complex	6.08	4.82	2.39	3.19E-06	5.16E-05
TC1000008663.hg.1	SEMA4G	Multiple_Complex	7.72	6.46	2.39	8.68E-06	0.0001
TC1900011940.hg.1	ECH1	Multiple_Complex	12.54	11.29	2.38	0.0005	0.0034
TC1200008814.hg.1	ANKRD13A	Multiple_Complex	8.55	7.3	2.38	3.08E-05	0.0003
TC0100009480.hg.1	HIPK1	Multiple_Complex	10.46	9.21	2.38	2.46E-05	0.0003
TC0700011953.hg.1	CYP3A5	Multiple_Complex	7.51	6.26	2.38	2.61E-06	4.36E-05
TC1700007189.hg.1	ALDH3A2	Multiple_Complex	10.28	9.03	2.38	3.74E-06	5.91E-05
TC1100011032.hg.1	CYB561A3	Multiple_Complex	9.04	7.79	2.38	0.0002	0.0014
TC1700007451.hg.1	CPD	Multiple_Complex	9.65	8.4	2.38	1.82E-05	0.0002
TC0200012404.hg.1	ZFP36L2	Multiple_Complex	10.62	9.37	2.38	2.98E-06	4.91E-05
TC0600010542.hg.1	SERPINB6	Multiple_Complex	9.42	8.17	2.38	1.56E-05	0.0002
TC1100009514.hg.1	TMEM45B	Multiple_Complex	10.82	9.57	2.38	7.07E-05	0.0007
TC0100008625.hg.1	PDE4B	Multiple_Complex	4.73	3.48	2.37	1.11E-05	0.0001
TC0100014275.hg.1	TTC22	Multiple_Complex	7.57	6.33	2.37	5.18E-06	7.79E-05

TC0400008668.hg.1	LARP1B	Multiple_Complex	8.54	7.3	2.37	2.79E-06	4.63E-05
TC0200013261.hg.1	RETSAT	Multiple_Complex	8.99	7.74	2.37	7.49E-07	1.51E-05
TC0100013634.hg.1	TMEM54	Multiple_Complex	8.98	7.73	2.37	7.32E-06	0.0001
TC1200012698.hg.1	CHPT1	Multiple_Complex	9.05	7.8	2.37	8.28E-06	0.0001
TC0800008478.hg.1	FZD6	Coding	9.5	8.26	2.37	8.99E-06	0.0001
TC2000007509.hg.1	CTSA	Multiple_Complex	11.21	9.97	2.36	4.66E-05	0.0005
TC0500008855.hg.1	SLC35A4	Multiple_Complex	10.11	8.88	2.36	8.88E-05	0.0009
TC1100010639.hg.1	TP53I11	Multiple_Complex	8.16	6.92	2.35	1.41E-05	0.0002
TC1200011770.hg.1	ALDH1L2	Multiple_Complex	6.6	5.36	2.35	7.98E-06	0.0001
TC0200007609.hg.1	SPTBN1	Multiple_Complex	11.5	10.27	2.35	4.80E-05	0.0005
TC1400010730.hg.1	EGLN3	Multiple_Complex	7.82	6.58	2.35	0.0001	0.001
TC1900010360.hg.1	ANKRD27	Multiple_Complex	9.04	7.81	2.35	0.0002	0.0015
TC1000009841.hg.1	PHYH	Multiple_Complex	8.7	7.47	2.35	1.57E-06	2.83E-05
TC1200007430.hg.1	SCAF11	NonCoding	6.89	5.66	2.35	0.0002	0.0016
TC0300010643.hg.1	CMTM6	Multiple_Complex	9.26	8.03	2.35	0.0002	0.0018
TC1900011398.hg.1	TMC4	Multiple_Complex	7.73	6.5	2.34	2.54E-05	0.0003
TC1700012276.hg.1	PNPO	Multiple_Complex	9.14	7.91	2.34	3.90E-05	0.0004
TC0100016629.hg.1	RNASEL	Coding	5.44	4.22	2.34	4.24E-05	0.0005
TC0100013315.hg.1	IFNLR1	Multiple_Complex	7.76	6.54	2.34	1.11E-05	0.0001
TC0800006447.hg.1	FBXO25	Multiple_Complex	7.6	6.38	2.34	1.26E-05	0.0002
TC0900009673.hg.1	MLLT3	Multiple_Complex	9.37	8.15	2.34	6.97E-06	9.93E-05
TC1700007481.hg.1	RAB11FIP4	Multiple_Complex	6.83	5.61	2.33	6.64E-05	0.0007
TC0600014162.hg.1	TSTD3	Multiple_Complex	6.4	5.18	2.33	0.0008	0.0055
TC0200010273.hg.1	NAB1	Multiple_Complex	9.7	8.48	2.33	3.47E-06	5.52E-05
TC0600013361.hg.1	CITED2	Coding	8.84	7.62	2.32	3.18E-06	5.14E-05
TC0300006993.hg.1	CRTAP	Multiple_Complex	10.33	9.11	2.32	0.0002	0.0016
TC0300013751.hg.1	BDH1	Multiple_Complex	9.01	7.8	2.32	0.0002	0.0018
TC0100008594.hg.1	ROR1	Multiple_Complex	8.38	7.17	2.32	2.11E-05	0.0003
TC1100006831.hg.1	ADM	Coding	8.45	7.24	2.32	8.94E-06	0.0001
TC0800007086.hg.1	DPYSL2	Multiple_Complex	10.91	9.69	2.31	7.08E-07	1.45E-05
TC1500008312.hg.1	IQGAP1	Multiple_Complex	10.76	9.55	2.31	3.43E-06	5.47E-05
TC0900008487.hg.1	BSPRY	Multiple_Complex	7.45	6.24	2.31	4.36E-06	6.70E-05
TC0X00007053.hg.1	MAOA	Multiple_Complex	7.06	5.85	2.31	1.61E-06	2.89E-05
TC1200010341.hg.1	PKP2	Multiple_Complex	11.59	10.39	2.31	1.70E-06	3.01E-05
TC2100008545.hg.1	NRIP1	Multiple_Complex	9.02	7.82	2.3	1.54E-05	0.0002
TC0900009711.hg.1	IFNE	Multiple_Complex	5.97	4.77	2.3	0.0013	0.0081
TC1200011099.hg.1	GNS	Multiple_Complex	11	9.8	2.3	3.12E-06	5.08E-05
TC0600010002.hg.1	SYNJ2	Multiple_Complex	8.43	7.23	2.3	2.22E-05	0.0003
TC1200009912.hg.1	STYK1	Coding	5.62	4.42	2.3	9.47E-05	0.0009
TC1200009196.hg.1	MLXIP	Multiple_Complex	7.95	6.75	2.29	1.21E-05	0.0002

TC1800007734.hg.1	ZNF236	Multiple_Complex	8.94	7.75	2.29	1.78E-06	3.14E-05
TC0500007681.hg.1	OCLN	Multiple_Complex	10.05	8.86	2.29	8.98E-06	0.0001
TC1800007518.hg.1	ZCCHC2	Multiple_Complex	7.34	6.14	2.29	3.92E-05	0.0004
TC0100018367.hg.1	CHRM3	Multiple_Complex	7.16	5.97	2.29	8.36E-06	0.0001
TC0100009908.hg.1	ECM1	Multiple_Complex	6.75	5.55	2.29	3.63E-05	0.0004
TC0400012458.hg.1	HPGD	Multiple_Complex	7.15	5.95	2.29	1.63E-06	2.92E-05
TC1900009459.hg.1	INSR	Multiple_Complex	7.03	5.83	2.29	3.02E-06	4.94E-05
TC1300008463.hg.1	FLT1	Multiple_Complex	5.58	4.39	2.29	7.86E-06	0.0001
TC0300008826.hg.1	NEK11	Multiple_Complex	4.56	3.37	2.29	9.25E-05	0.0009
TC1000010065.hg.1	ARHGAP21	Multiple_Complex	8.56	7.37	2.29	4.10E-06	6.37E-05
TC1800007620.hg.1	SOCS6	Multiple_Complex	7.33	6.14	2.28	4.00E-06	6.25E-05
TC0400011791.hg.1	ANKRD50	Multiple_Complex	6.46	5.27	2.28	0.0035	0.0179
TC1500010106.hg.1	TSPAN3	Multiple_Complex	10.9	9.71	2.28	4.32E-05	0.0005
TC1900006819.hg.1	TRIP10	Multiple_Complex	10.18	8.99	2.28	4.51E-06	6.90E-05
TC0600007012.hg.1	CD83	Multiple_Complex	6.76	5.57	2.28	0.0006	0.0044
TC1200012877.hg.1	ZNF605	Multiple_Complex	7.12	5.93	2.28	4.66E-06	7.08E-05
TC2200006947.hg.1	ASPHD2	Multiple_Complex	6.44	5.25	2.28	3.66E-06	5.79E-05
TC0400009258.hg.1	PALLD	Multiple_Complex	8.73	7.55	2.28	4.79E-06	7.26E-05
TC1500010724.hg.1	GANC	Multiple_Complex	7.57	6.39	2.27	2.06E-05	0.0002
TC1800009029.hg.1	CYB5A	Multiple_Complex	9.15	7.97	2.27	1.55E-05	0.0002
TC1600008199.hg.1	ZFP90	Multiple_Complex	8.37	7.18	2.27	2.18E-05	0.0003
TC1400007227.hg.1	LGALS3	Multiple_Complex	10.92	9.74	2.27	0.0002	0.0018
TC1100011122.hg.1	HRASLS5	Multiple_Complex	6.69	5.51	2.26	4.36E-05	0.0005
TC1500010752.hg.1	TLN2	NonCoding	6.58	5.4	2.26	6.02E-06	8.83E-05
TC1500009669.hg.1	VPS13C	Multiple_Complex	7.85	6.68	2.26	0.0003	0.0027
TC1500010720.hg.1	IVD	Multiple_Complex	8.85	7.68	2.26	0.0032	0.0168
TC1300006987.hg.1	DGKH	Multiple_Complex	7.53	6.35	2.26	2.37E-05	0.0003
TC0200007048.hg.1	MAPRE3	Multiple_Complex	7.3	6.13	2.26	4.88E-06	7.37E-05
TC1900011866.hg.1	RAB3D	Multiple_Complex	7.65	6.48	2.26	2.91E-06	4.81E-05
TC1200006610.hg.1	PLEKHG6	Multiple_Complex	7.1	5.93	2.25	0.0002	0.0016
TC0X00009492.hg.1	NDP	Multiple_Complex	5.47	4.3	2.25	0.0004	0.0033
TC1200011767.hg.1	SLC41A2	Multiple_Complex	7.36	6.19	2.25	1.88E-05	0.0002
TC1600008709.hg.1	COX4I1	Multiple_Complex	9.44	8.26	2.25	4.93E-06	7.45E-05
TC1500006766.hg.1	CHRNA7	Multiple_Complex	6.63	5.46	2.25	0.0018	0.0105
TC2200007495.hg.1	SREBF2	Multiple_Complex	9.34	8.17	2.25	8.84E-06	0.0001
TC1200012110.hg.1	PEBP1	NonCoding	6.92	5.75	2.25	6.12E-06	8.95E-05
TC0300007067.hg.1	MYD88	Multiple_Complex	9.73	8.57	2.25	0.0003	0.0023
TC0300012073.hg.1	LSAMP	Multiple_Complex	7.53	6.36	2.24	1.25E-05	0.0002
TC0600011508.hg.1	TAP1	Multiple_Complex	6.68	5.52	2.24	2.91E-06	4.81E-05
TC1800008418.hg.1	GAREM1	Multiple_Complex	8.01	6.84	2.24	4.57E-06	6.96E-05

TC1900010856.hg.1	PLAUR	Multiple_Complex	7.46	6.3	2.24	2.16E-05	0.0003
TC1800007523.hg.1	PHLPP1	Multiple_Complex	8.47	7.31	2.24	0.0001	0.001
TC1200012864.hg.1	CCDC92	Multiple_Complex	7.1	5.94	2.24	1.39E-05	0.0002
TC1100011983.hg.1	VSTM5	Multiple_Complex	6.07	4.91	2.23	6.51E-05	0.0007
TC0800009828.hg.1	HR	Multiple_Complex	7.3	6.14	2.23	5.69E-06	8.42E-05
TC1500006965.hg.1	RAD51	Multiple_Complex	7.17	6.01	2.23	0.0005	0.0038
TC0800009201.hg.1	GSDMD	Multiple_Complex	7.33	6.17	2.23	6.24E-05	0.0006
TC1900010005.hg.1	RAB3A	Multiple_Complex	7.66	6.5	2.23	1.08E-05	0.0001
TC2000007281.hg.1	MANBAL	Multiple_Complex	10.48	9.32	2.23	0.0011	0.0069
TC0800009752.hg.1	ASAH1	Multiple_Complex	10.31	9.15	2.23	3.09E-06	5.03E-05
TC2200007660.hg.1	TTC38	Multiple_Complex	8.86	7.7	2.23	2.06E-06	3.56E-05
TC0800011881.hg.1	NDRG1	Multiple_Complex	7.52	6.37	2.23	1.42E-05	0.0002
TC0300007479.hg.1	PHF7	Multiple_Complex	5.53	4.37	2.23	3.29E-06	5.29E-05
TC0900012054.hg.1	NRARP	Coding	7.73	6.57	2.23	9.50E-06	0.0001
TC0600011234.hg.1	HIST1H4L	Coding	8.46	7.3	2.23	2.22E-05	0.0003
TC0400011744.hg.1	ANXA5	Multiple_Complex	11.28	10.13	2.23	1.30E-05	0.0002
TC0100010112.hg.1	SLC50A1	Multiple_Complex	8.25	7.1	2.22	3.74E-05	0.0004
TC0300009033.hg.1	PLS1	Multiple_Complex	10.32	9.17	2.22	6.91E-06	9.86E-05
TC1700012249.hg.1	MIR4728	Multiple_Complex	7.95	6.8	2.22	2.51E-06	4.21E-05
TC1900008498.hg.1	NUCB1	Multiple_Complex	9.33	8.18	2.22	5.89E-05	0.0006
TC1200012835.hg.1	NT5DC3	Multiple_Complex	9	7.85	2.22	4.56E-05	0.0005
TC1200012234.hg.1	CLIP1	Multiple_Complex	8.81	7.66	2.22	2.86E-05	0.0003
TC1600011518.hg.1	DOC2A	Multiple_Complex	5.63	4.48	2.22	2.01E-06	3.48E-05
TC0600011876.hg.1	YIPF3	Multiple_Complex	9.72	8.57	2.22	1.17E-05	0.0002
TC1100010992.hg.1	TCN1	Multiple_Complex	4.57	3.42	2.21	1.15E-05	0.0002
TC0500011219.hg.1	AP3B1	Multiple_Complex	8.91	7.76	2.21	0.0001	0.0013
TC0800012173.hg.1	EPPK1	Coding	6.6	5.45	2.21	1.90E-05	0.0002
TC1700010408.hg.1	CCT6B	Multiple_Complex	4.47	3.33	2.21	0.0002	0.0014
TC0900011072.hg.1	ABCA1	Multiple_Complex	6.27	5.12	2.21	3.61E-06	5.71E-05
TC0600009228.hg.1	NT5DC1	Multiple_Complex	9.42	8.27	2.21	4.21E-06	6.52E-05
TC1100012352.hg.1	TMPRSS5	Multiple_Complex	6.91	5.77	2.21	0.0002	0.0015
TC0100013920.hg.1	C1orf210	Coding	7.66	6.52	2.2	6.64E-05	0.0007
TC1900010078.hg.1	ZNF14	Multiple_Complex	4.79	3.65	2.2	3.47E-06	5.52E-05
TC0500008921.hg.1	NDFIP1	Multiple_Complex	10.13	8.99	2.2	0.0004	0.0028
TC0200011022.hg.1	SP140L	Multiple_Complex	6.33	5.2	2.2	8.49E-06	0.0001
TC1500009702.hg.1	RPS27L	Multiple_Complex	9.12	7.98	2.2	9.39E-05	0.0009
TC0X00006816.hg.1	PDK3	Multiple_Complex	7.73	6.59	2.2	9.27E-05	0.0009
TC1200011791.hg.1	CKAP4	Multiple_Complex	9.73	8.59	2.2	5.77E-06	8.50E-05
TC0900009003.hg.1	POMT1	Multiple_Complex	9.2	8.06	2.2	2.62E-05	0.0003
TC1500008309.hg.1	ZNF774	Multiple_Complex	7.72	6.59	2.19	7.27E-06	0.0001

TC1600007374.hg.1	NFATC2IP	Multiple_Complex	8.47	7.34	2.19	0.0001	0.0012
TC2200007963.hg.1	DGCR2	Multiple_Complex	8.37	7.24	2.19	2.08E-05	0.0003
TC1000011596.hg.1	DNMBP	Multiple_Complex	9.28	8.15	2.19	2.74E-05	0.0003
TC1600011347.hg.1	ALG1	Multiple_Complex	9.4	8.27	2.19	0.0035	0.018
TC1500008893.hg.1	MTMR10	Multiple_Complex	7.65	6.52	2.19	0.0001	0.0012
TC1100011867.hg.1	FZD4	Coding	7.02	5.89	2.18	2.52E-05	0.0003
TC1500009438.hg.1	LYSMD2	Coding	5.57	4.44	2.18	7.50E-06	0.0001
TC0700008565.hg.1	SLC12A9	Multiple_Complex	7.01	5.89	2.18	0.0014	0.0085
TC0700013481.hg.1	PRSS1	Multiple_Complex	4.9	3.78	2.18	3.90E-05	0.0004
TSUnmapped00000714.hg.1	MANSC1	Coding	4.18	3.05	2.18	3.33E-05	0.0004
TC1400009711.hg.1	NEK9	Multiple_Complex	8.72	7.6	2.18	1.22E-05	0.0002
TC1200008792.hg.1	UNG	Multiple_Complex	8.59	7.47	2.18	3.39E-05	0.0004
TC1900011067.hg.1	TMEM143	Multiple_Complex	5.9	4.77	2.18	0.0223	0.0784
TC0600013275.hg.1	MAP7	Multiple_Complex	7.93	6.81	2.18	6.59E-06	9.51E-05
TC1000010117.hg.1	ACBD5	Multiple_Complex	10.05	8.93	2.18	7.82E-06	0.0001
TC2000006642.hg.1	BMP2	Coding	6.7	5.57	2.18	8.38E-05	0.0008
TSUnmapped00000639.hg.1	SERTAD4	Coding	4.93	3.81	2.17	3.09E-06	5.03E-05
TC1900006616.hg.1	GNA11	Multiple_Complex	9.3	8.18	2.17	8.04E-06	0.0001
TC1800009290.hg.1	MYO5B	Multiple_Complex	8.95	7.84	2.17	5.96E-06	8.74E-05
TC0800009997.hg.1	KIF13B	Multiple_Complex	9.13	8.02	2.17	0.0002	0.0019
TC0100015716.hg.1	MTMR11	Multiple_Complex	7.52	6.4	2.17	0.0006	0.0041
TC1900010375.hg.1	RHPN2	Multiple_Complex	9.42	8.3	2.17	3.57E-06	5.66E-05
TC0400008105.hg.1	HERC6	Multiple_Complex	8.33	7.22	2.16	0.0003	0.0021
TC0200013265.hg.1	CAPG	Multiple_Complex	8.26	7.15	2.16	1.75E-05	0.0002
TC1200011435.hg.1	RASSF9	Coding	4.28	3.17	2.16	8.66E-06	0.0001
TC1400007595.hg.1	SIPA1L1	Multiple_Complex	7.32	6.21	2.16	5.32E-05	0.0006
TC0500008871.hg.1	PCDHA9	Multiple_Complex	6.88	5.77	2.16	1.81E-05	0.0002
TC0100013281.hg.1	ASAP3	Multiple_Complex	8.48	7.36	2.16	0.001	0.0064
TC0400011464.hg.1	MANBA	Multiple_Complex	7.89	6.78	2.16	2.15E-05	0.0003
TC1600008150.hg.1	ELMO3	Multiple_Complex	7.51	6.4	2.16	0.0002	0.0018
TC1900008752.hg.1	ZNF813	Multiple_Complex	8.22	7.11	2.16	7.01E-06	9.99E-05
TC0700012797.hg.1	PARP12	Multiple_Complex	9.07	7.96	2.16	9.76E-06	0.0001
TC0100007486.hg.1	SFN	Coding	11.19	10.08	2.16	4.43E-06	6.79E-05
TC0800007367.hg.1	ADAM9	Multiple_Complex	9.91	8.8	2.16	2.67E-06	4.44E-05
TC0100014794.hg.1	ODF2L	Multiple_Complex	7.95	6.84	2.16	0.0001	0.0013
TC0200016497.hg.1	C2orf15	Multiple_Complex	6.57	5.46	2.15	7.95E-05	0.0008
TC1000008397.hg.1	IFIT3	Coding	7.39	6.29	2.15	1.64E-05	0.0002
TC0100018299.hg.1	SLC27A3	Multiple_Complex	6.75	5.64	2.15	1.14E-05	0.0002
TC1200011936.hg.1	ATXN2	Multiple_Complex	10.18	9.08	2.15	4.41E-06	6.77E-05
TC1200012561.hg.1	ZNF891	Multiple_Complex	6.46	5.36	2.15	9.05E-05	0.0009

TC1000006652.hg.1	PFKFB3	Multiple_Complex	8.05	6.95	2.15	1.21E-05	0.0002
TC1200012736.hg.1	ZNF10	Multiple_Complex	6.34	5.24	2.15	1.89E-05	0.0002
TC1700007599.hg.1	AP2B1	Multiple_Complex	11.04	9.94	2.14	0.0003	0.0027
TC1300006799.hg.1	FRY	Multiple_Complex	4.6	3.5	2.14	4.83E-05	0.0005
TC0700011796.hg.1	SAMD9	Coding	7.37	6.27	2.14	5.14E-05	0.0005
TC1900007290.hg.1	TMEM38A	Multiple_Complex	6.1	5	2.14	1.18E-05	0.0002
TC0600011349.hg.1	RNF39	Coding	6.36	5.26	2.14	0.0029	0.0155
TC0800010138.hg.1	RAB11FIP1	Multiple_Complex	8.31	7.21	2.14	1.33E-05	0.0002
TC1300006923.hg.1	NHLRC3	Multiple_Complex	7.68	6.58	2.14	1.42E-05	0.0002
TC1900011794.hg.1	ZNF701	Multiple_Complex	9.38	8.28	2.14	3.61E-06	5.71E-05
TC1200009172.hg.1	ORAI1	Multiple_Complex	7.5	6.41	2.14	0.0002	0.0016
TC1100007257.hg.1	CAT	Multiple_Complex	9.87	8.78	2.14	1.29E-05	0.0002
TC0800008782.hg.1	ZNF572	Coding	6.14	5.04	2.14	0.0001	0.0013
TC0300013555.hg.1	MB21D2	Coding	7.54	6.44	2.14	0.0009	0.0061
TC0700008567.hg.1	TRIP6	Multiple_Complex	8.95	7.86	2.14	0.0002	0.0014
TC0300013123.hg.1	PLD1	Multiple_Complex	8.27	7.18	2.13	8.40E-06	0.0001
TC0600009753.hg.1	STXBP5	Multiple_Complex	7.39	6.3	2.13	4.42E-05	0.0005
TC2000006861.hg.1	RIN2	Multiple_Complex	8.44	7.35	2.13	1.21E-05	0.0002
TC1100008005.hg.1	CAPN1	Multiple_Complex	9.93	8.84	2.13	0.0089	0.0381
TC1600009331.hg.1	C16orf72	NonCoding	4.57	3.48	2.13	0.0065	0.0296
TC0800009619.hg.1	CTSB	Multiple_Complex	11.02	9.93	2.13	0.0002	0.0015
TC0300011759.hg.1	STX19	Coding	4.59	3.5	2.13	6.30E-05	0.0006
TC0100015715.hg.1	SF3B4	Multiple_Complex	11.19	10.1	2.13	1.06E-05	0.0001
TC1900011399.hg.1	MBOAT7	Multiple_Complex	9.06	7.97	2.13	0.0001	0.0012
TC1600009654.hg.1	GP2	Multiple_Complex	6.13	5.04	2.13	0.0034	0.0176
TC1700009850.hg.1	PMP22	Multiple_Complex	8.62	7.53	2.13	2.18E-05	0.0003
TC1400008767.hg.1	STXBP6	Multiple_Complex	8.38	7.29	2.12	0.0028	0.015
TC0900008178.hg.1	NANS	Multiple_Complex	9.27	8.18	2.12	5.45E-06	8.13E-05
TC1900011796.hg.1	NDUFA3	NonCoding	7.1	6.02	2.12	6.57E-06	9.48E-05
TC0800009208.hg.1	ZNF623	Coding	8.08	7	2.12	3.37E-05	0.0004
TC0300012720.hg.1	PLSCR4	Multiple_Complex	5.4	4.31	2.11	0.0014	0.0086
TC1200012043.hg.1	MED13L	Multiple_Complex	9.62	8.54	2.11	2.41E-05	0.0003
TC0300006968.hg.1	CMTM7	Multiple_Complex	9.5	8.42	2.11	0.0005	0.0035
TC0600012713.hg.1	PREP	Multiple_Complex	10.11	9.03	2.11	1.57E-05	0.0002
TC1800009245.hg.1	SERPINB8	Multiple_Complex	7.07	5.99	2.11	8.57E-05	0.0008
TC0800012444.hg.1	PABPC1	Multiple_Complex	12.14	11.06	2.11	0.0004	0.0031
TC1700008920.hg.1	ITGB4	Multiple_Complex	8.88	7.8	2.11	0.0003	0.0024
TC2100007355.hg.1	PFKL	Multiple_Complex	8.85	7.77	2.11	7.06E-05	0.0007
TC1000012542.hg.1	ZNF438	Multiple_Complex	7.43	6.36	2.11	6.02E-05	0.0006
TC0100013287.hg.1	E2F2	Multiple_Complex	7.23	6.16	2.11	6.26E-06	9.14E-05

TC1600010593.hg.1	CMTM4	Multiple_Complex	9.7	8.63	2.11	9.06E-05	0.0009
TC0500013109.hg.1	ZFP62	Multiple_Complex	9.22	8.15	2.11	6.72E-05	0.0007
TC1500010047.hg.1	SCAMP2	Multiple_Complex	9.49	8.41	2.11	0.0002	0.0016
TC1700007890.hg.1	ATP6V0A1	Multiple_Complex	9.92	8.85	2.1	1.26E-05	0.0002
TC1500007084.hg.1	CASC4	Multiple_Complex	9.37	8.29	2.1	0.0001	0.0012
TC0200015645.hg.1	ABCA12	Multiple_Complex	4.99	3.92	2.1	4.21E-06	6.52E-05
TC0500008698.hg.1	JADE2	Multiple_Complex	8.8	7.73	2.1	0.0224	0.0789
TC0100007691.hg.1	KIAA1522	Multiple_Complex	8.04	6.97	2.1	6.77E-06	9.73E-05
TC0100010454.hg.1	MGST3	Multiple_Complex	10.01	8.94	2.1	0.0002	0.0015
TC0400011695.hg.1	SEC24D	Multiple_Complex	8.33	7.26	2.1	2.90E-05	0.0003
TC1800008458.hg.1	ZNF24	Coding	9.04	7.97	2.1	0.0004	0.003
TC1700008342.hg.1	PCTP	Multiple_Complex	8.13	7.06	2.1	2.36E-05	0.0003
TC0400012780.hg.1	PI4K2B	Multiple_Complex	8.79	7.72	2.1	4.19E-06	6.49E-05
TC0500010628.hg.1	ANXA2R	Coding	6.32	5.25	2.1	3.81E-05	0.0004
TC0100016322.hg.1	GPR161	Multiple_Complex	6.02	4.95	2.1	9.34E-05	0.0009
TC0800009242.hg.1	HGH1	Multiple_Complex	8.92	7.85	2.09	0.0038	0.019
TC1500009952.hg.1	PKM	Multiple_Complex	12.63	11.56	2.09	0.0003	0.0023
TC0900008474.hg.1	SNX30	Multiple_Complex	8.01	6.94	2.09	0.0001	0.0011
TC1600009194.hg.1	ZNF597	Coding	6.81	5.75	2.09	3.50E-05	0.0004
TC1700008228.hg.1	ITGA3	Multiple_Complex	6.69	5.63	2.09	0.0001	0.001
TC0800010749.hg.1	NCOA2	Multiple_Complex	8.68	7.62	2.09	1.53E-05	0.0002
TC1000007462.hg.1	ZNF22	Multiple_Complex	7.48	6.42	2.09	0.0001	0.0011
TC1100007233.hg.1	KIAA1549L	Multiple_Complex	6.19	5.13	2.09	0.0001	0.0011
TC0900012189.hg.1	TMEM141	Multiple_Complex	8.8	7.74	2.09	8.00E-05	0.0008
TC0200012307.hg.1	SOS1	Multiple_Complex	9.06	8	2.09	0.0002	0.0017
TC0900011300.hg.1	TNFSF15	Coding	6.39	5.33	2.08	0.0002	0.002
TC1900011683.hg.1	HSH2D	Multiple_Complex	7.72	6.66	2.08	0.001	0.0064
TC1900012055.hg.1	VN1R1	Multiple_Complex	5.45	4.39	2.08	0.0021	0.0116
TC0900012246.hg.1	ALDH1A1	NonCoding	4.58	3.53	2.08	0.0001	0.0011
TC0300013839.hg.1	ACY1	Multiple_Complex	8.35	7.3	2.08	0.0066	0.03
TC1900012027.hg.1	ZNF320	NonCoding	4.08	3.03	2.08	5.70E-05	0.0006
TC2000010009.hg.1	BLCAP	NonCoding	6.67	5.62	2.08	2.07E-05	0.0002
TC0100007444.hg.1	CNKSR1	Multiple_Complex	7.44	6.38	2.08	5.60E-05	0.0006
TC1300008443.hg.1	LNX2	Multiple_Complex	7.69	6.63	2.08	3.60E-05	0.0004
TC1200010757.hg.1	KRT6C	Multiple_Complex	6.85	5.79	2.08	0.0029	0.0153
TC1400007975.hg.1	C14orf159	Multiple_Complex	7.29	6.24	2.07	3.93E-05	0.0004
TC1000009276.hg.1	DOCK1	Multiple_Complex	7.41	6.36	2.07	3.32E-05	0.0004
TC1000008881.hg.1	MXI1	Multiple_Complex	9.36	8.31	2.07	2.37E-05	0.0003
TC1700006999.hg.1	ADORA2B	Multiple_Complex	8.41	7.35	2.07	0.0002	0.0014
TC0600010331.hg.1	FAM120B	Multiple_Complex	6.26	5.21	2.07	0.0002	0.0019

TC0100013692.hg.1	KIAA0319L	Multiple_Complex	9.56	8.51	2.07	4.43E-05	0.0005
TC1400009481.hg.1	PLEK2	Multiple_Complex	10.55	9.5	2.07	1.33E-05	0.0002
TC0100010152.hg.1	LMNA	Multiple_Complex	10.64	9.59	2.07	0.0004	0.0031
TC0900011209.hg.1	C9orf84	Multiple_Complex	3.68	2.63	2.07	3.40E-05	0.0004
TC1200006888.hg.1	CDKN1B	Multiple_Complex	9.28	8.23	2.06	1.74E-05	0.0002
TC0100015728.hg.1	APH1A	Multiple_Complex	10.14	9.1	2.06	2.14E-05	0.0003
TC1600009486.hg.1	PLA2G10	Multiple_Complex	6.59	5.55	2.06	9.34E-05	0.0009
TC1400008118.hg.1	AK7	Multiple_Complex	7.81	6.77	2.06	4.27E-06	6.60E-05
TC2000007830.hg.1	TFAP2C	Coding	5.56	4.52	2.06	4.04E-06	6.31E-05
TC1900011233.hg.1	KLK10	Multiple_Complex	7.81	6.77	2.06	1.74E-05	0.0002
TC0800009252.hg.1	SLC52A2	Multiple_Complex	9.31	8.27	2.06	0.0053	0.0253
TC0400008053.hg.1	AFF1	Multiple_Complex	9.99	8.95	2.06	5.23E-05	0.0005
TC0300008994.hg.1	PXYLP1	Multiple_Complex	5.78	4.74	2.06	0.0002	0.0016
TC1900007331.hg.1	COLGALT1	Multiple_Complex	10.78	9.74	2.06	0.0001	0.0012
TC1200011870.hg.1	KCTD10	Multiple_Complex	8.25	7.21	2.05	0.0001	0.0011
TC0800011597.hg.1	EXT1	NonCoding	6.46	5.42	2.05	1.16E-05	0.0002
TC1400009134.hg.1	SAV1	Multiple_Complex	9.33	8.3	2.05	1.55E-05	0.0002
TC1600009731.hg.1	COG7	Multiple_Complex	8.98	7.95	2.05	0.0002	0.0015
TC1100006966.hg.1	NCR3LG1	Multiple_Complex	9.07	8.03	2.05	2.68E-05	0.0003
TC1800008405.hg.1	TRAPPC8	Multiple_Complex	8.54	7.51	2.05	3.89E-05	0.0004
TC2000009216.hg.1	SLPI	Multiple_Complex	6.83	5.8	2.05	0.0001	0.001
TC2100008568.hg.1	TMPRSS2	Multiple_Complex	8.82	7.78	2.05	4.45E-05	0.0005
TC0300009855.hg.1	IL1RAP	Multiple_Complex	6.51	5.48	2.05	3.76E-05	0.0004
TC1400009668.hg.1	PNMA1	Coding	10.44	9.4	2.05	0.0012	0.0073
TC1700006645.hg.1	PLD2	Multiple_Complex	7.3	6.27	2.05	0.0002	0.0015
TC1000008733.hg.1	MFSD13A	Multiple_Complex	9.47	8.44	2.05	2.86E-05	0.0003
TC0900010366.hg.1	KLF9	Multiple_Complex	7.29	6.25	2.05	4.02E-05	0.0004
TC0100015784.hg.1	SELENBP1	Multiple_Complex	6.38	5.35	2.04	0.0004	0.0033
TC0100007542.hg.1	SMPDL3B	Multiple_Complex	8.08	7.05	2.04	8.36E-05	0.0008
TC0800007057.hg.1	DOCK5	Multiple_Complex	9.65	8.62	2.04	5.10E-05	0.0005
TC0500013239.hg.1	PCDHB3	Coding	5.79	4.76	2.04	0.001	0.0063
TC0400011208.hg.1	FAM175A	Multiple_Complex	8.3	7.27	2.04	0.0005	0.0037
TC1200008803.hg.1	MVK	Multiple_Complex	7.64	6.61	2.04	6.88E-05	0.0007
TC0800006760.hg.1	FDFT1	Multiple_Complex	11.02	9.99	2.04	1.75E-05	0.0002
TC1300008735.hg.1	VWA8	Multiple_Complex	9.39	8.37	2.03	2.94E-05	0.0003
TC1100011514.hg.1	DHCR7	Multiple_Complex	11.05	10.03	2.03	1.69E-05	0.0002
TC1600011318.hg.1	DECR2	Multiple_Complex	9.07	8.05	2.03	0.0003	0.0023
TC0900008490.hg.1	C9orf43	Multiple_Complex	5.52	4.49	2.03	8.89E-05	0.0009
TC1200011671.hg.1	UHRF1BP1L	Multiple_Complex	9.09	8.07	2.03	3.86E-05	0.0004
TC0100015921.hg.1	ADAR	Multiple_Complex	9.41	8.39	2.03	6.15E-05	0.0006

TC1900009048.hg.1	ZNF584	Multiple_Complex	8.85	7.84	2.03	0.0007	0.0045
TC1900011874.hg.1	ZNF878	Coding	5.29	4.27	2.03	2.74E-05	0.0003
TC1100013129.hg.1	CTSD	Multiple_Complex	10.58	9.56	2.02	0.0002	0.0016
TC1900010574.hg.1	ZNF571	Multiple_Complex	7.3	6.28	2.02	0.0037	0.019
TSUnmapped00000308.hg.1	BCL2L14	Coding	7.03	6.02	2.02	9.68E-05	0.0009
TC0X00010266.hg.1	PCDH11X	NonCoding	4.51	3.49	2.02	0.0001	0.0013
TC1900011318.hg.1	ZNF888	Multiple_Complex	7.84	6.82	2.02	7.39E-05	0.0007
TC1700006721.hg.1	SLC16A13	Multiple_Complex	8.76	7.75	2.02	0.0004	0.0029
TC0400013004.hg.1	MTNR1A	Coding	6.34	5.33	2.02	7.97E-05	0.0008
TC0600014126.hg.1	GLTSCR1L	Multiple_Complex	7.57	6.56	2.01	0.0001	0.0013
TC0800006869.hg.1	SLC7A2	Multiple_Complex	5.48	4.47	2.01	3.64E-05	0.0004
TC0900012167.hg.1	GSN	Multiple_Complex	8.36	7.35	2.01	0.0078	0.0343
TSUnmapped00000137.hg.1	ABO	Coding	6.27	5.26	2.01	0.0003	0.0022
TC0X00008945.hg.1	PRKX	Multiple_Complex	8.1	7.09	2.01	0.0003	0.0024
TC0500011260.hg.1	SERINC5	Multiple_Complex	10.03	9.02	2.01	5.18E-05	0.0005
TC0100010111.hg.1	EFNA1	Multiple_Complex	6.96	5.95	2.01	6.27E-05	0.0006
TC0900006539.hg.1	JAK2	NonCoding	4.52	3.51	2.01	1.85E-05	0.0002
TC1400010716.hg.1	HOMEZ	Multiple_Complex	8.92	7.91	2.01	0.0001	0.0011
TSUnmapped00000485.hg.1	RPS6KA1	Coding	8.63	7.62	2.01	1.16E-05	0.0002
TC0500013232.hg.1	SMAD5	Multiple_Complex	10.91	9.91	2.01	1.54E-05	0.0002
TC1000011904.hg.1	ABLIM1	Multiple_Complex	11.13	10.13	2.01	7.86E-06	0.0001
TC0400012213.hg.1	CTSO	Coding	4.51	3.5	2.01	3.41E-05	0.0004
TC0X00011353.hg.1	ASB9	Multiple_Complex	7.25	6.25	2.01	0.0009	0.0057
TC1700008232.hg.1	PDK2	Multiple_Complex	8.88	7.87	2.01	0.0003	0.0022
TC1700008019.hg.1	CCDC103	Coding	5.81	4.8	2.01	0.0001	0.0011
TC0700011710.hg.1	SLC25A40	Multiple_Complex	8.37	7.36	2	1.74E-05	0.0002
TC1400007899.hg.1	SPATA7	Multiple_Complex	6.81	5.81	2	0.0012	0.0074
TC1900011331.hg.1	ZNF665	Multiple_Complex	5.21	4.21	2	7.66E-05	0.0008
TC1600011578.hg.1	FBXO31	Multiple_Complex	9.41	8.41	2	0.001	0.0067
TC0700009691.hg.1	ASIC3	Multiple_Complex	6.61	5.61	2	1.16E-05	0.0002
TC0200010053.hg.1	HOXD1	Multiple_Complex	7.19	6.19	2	0.0005	0.0038
TC0600008632.hg.1	BCKDHB	Multiple_Complex	8.64	7.64	2	0.0011	0.0068
TC0100013804.hg.1	BMP8B	Coding	6.87	5.87	2	0.0001	0.001
TC0800011731.hg.1	KIAA0196	Multiple_Complex	8.95	7.95	2	5.52E-05	0.0006
TC1300008289.hg.1	ZDHHC20	Multiple_Complex	10.02	9.02	2	1.52E-05	0.0002
TC1400006663.hg.1	LRP10	Multiple_Complex	9.04	8.04	2	1.01E-05	0.0001
TC1900011707.hg.1	GPI	Multiple_Complex	11.89	10.89	2	0.0053	0.0252
TC0900009424.hg.1	GLIS3	Multiple_Complex	6.93	5.93	2	5.91E-05	0.0006
TC1900008727.hg.1	ERVV-2	Coding	4.73	3.73	2	0.0002	0.0015
TC0300010931.hg.1	LZTFL1	Multiple_Complex	8.1	7.1	2	3.21E-05	0.0004

TC1900011060.hg.1	LIG1	Multiple_Complex	7.47	6.47	2	0.0015	0.0089
TC2000007202.hg.1	ACSS2	Multiple_Complex	7.52	6.52	2	0.0011	0.007
TC0100018476.hg.1	PEX11B	Multiple_Complex	7.99	6.99	2	2.25E-05	0.0003
TC1100013209.hg.1	PAK1	Multiple_Complex	8.19	7.2	2	3.75E-05	0.0004
TC0700008147.hg.1	RSBN1L	Multiple_Complex	8.11	9.11	-2	0.0051	0.0242
TC0400009029.hg.1	FHDC1	Multiple_Complex	7.08	8.08	-2	0.0005	0.0037
TC1000012525.hg.1	ACBD7	Multiple_Complex	6.21	7.21	-2	0.0178	0.0658
TC0400012386.hg.1	C4orf27	Multiple_Complex	6.94	7.94	-2	0.0002	0.002
TC0600009508.hg.1	TBPL1	Multiple_Complex	7.88	8.88	-2	3.80E-05	0.0004
TC0500013349.hg.1	FBN2	Multiple_Complex	6.2	7.2	-2	0.0003	0.0025
TC0400007128.hg.1	TBC1D19	Multiple_Complex	5.77	6.77	-2	0.0002	0.0016
TC0300009035.hg.1	TRPC1	Multiple_Complex	5.65	6.65	-2	0.0005	0.0033
TC2200008447.hg.1	PES1	Multiple_Complex	8.88	9.88	-2	7.53E-05	0.0007
TC0200010362.hg.1	COQ10B	Multiple_Complex	7.83	8.83	-2	5.06E-05	0.0005
TC0700009816.hg.1	EN2	Coding	5.44	6.44	-2	0.0002	0.002
TC1200008706.hg.1	KIAA1033	Multiple_Complex	7.45	8.45	-2	0.0038	0.0193
TC1600010596.hg.1	DYNC1LI2	Multiple_Complex	9.9	10.9	-2	0.0008	0.0051
TC1000007748.hg.1	CDK1	Multiple_Complex	9.22	10.23	-2	2.60E-05	0.0003
TC0400006990.hg.1	MED28	Multiple_Complex	8.22	9.22	-2	0.0003	0.0021
TC0700008625.hg.1	PRKRIP1	Multiple_Complex	7.61	8.61	-2	2.77E-05	0.0003
TC0200016662.hg.1	CALM2	Multiple_Complex	10.59	11.59	-2	4.56E-05	0.0005
TC0200012925.hg.1	C1D	Multiple_Complex	7.33	8.34	-2	3.34E-05	0.0004
TC0300014020.hg.1	CLDND1	Multiple_Complex	9.25	10.25	-2.01	0.0006	0.0043
TC0700008328.hg.1	RBM48	Coding	7.16	8.17	-2.01	0.0003	0.0024
TC0200014995.hg.1	WIPF1	Multiple_Complex	6.46	7.47	-2.01	1.57E-05	0.0002
TC0800007351.hg.1	TACC1	Multiple_Complex	7.94	8.95	-2.01	0.0004	0.0028
TC0600007302.hg.1	BTN2A2	Multiple_Complex	7.11	8.12	-2.01	0.0008	0.0051
TC0X00010642.hg.1	NKRF	Multiple_Complex	8.03	9.04	-2.01	0.0002	0.0017
TC1700006679.hg.1	RPAIN	Multiple_Complex	8.51	9.52	-2.01	0.0001	0.0011
TC0100013071.hg.1	MFAP2	Multiple_Complex	6.82	7.83	-2.01	6.50E-06	9.41E-05
TC1700012469.hg.1	SUMO2	Multiple_Complex	11.09	12.1	-2.01	0.0001	0.0013
TC0500007123.hg.1	DNAJC21	Multiple_Complex	7.19	8.2	-2.01	0.0041	0.0206
TC1100011811.hg.1	CCDC90B	Multiple_Complex	8.43	9.44	-2.01	5.13E-05	0.0005
TC0200014899.hg.1	METTL8	Multiple_Complex	8.49	9.5	-2.01	0.0142	0.0552
TC1000008898.hg.1	RBM20	Multiple_Complex	6.17	7.18	-2.02	0.0017	0.01
TC1000012600.hg.1	FAM24B	Multiple_Complex	7.09	8.11	-2.02	3.10E-05	0.0004
TC0X00009498.hg.1	FUNDC1	Multiple_Complex	7.39	8.4	-2.02	0.0001	0.001
TC0X00008866.hg.1	VBP1	Multiple_Complex	8.39	9.4	-2.02	1.33E-05	0.0002
TC1700009137.hg.1	RPTOR	Multiple_Complex	6.5	7.51	-2.02	0.0006	0.004
TC0100013545.hg.1	SDC3	Multiple_Complex	6.81	7.82	-2.02	0.0004	0.0032

TC1100008664.hg.1	C11orf73	Multiple_Complex	8.88	9.89	-2.02	3.46E-05	0.0004
TC1700010645.hg.1	KRTAP3-2	Coding	5.56	6.58	-2.02	1.18E-05	0.0002
TC1100010254.hg.1	SAAL1	Multiple_Complex	6.8	7.81	-2.02	0.0002	0.0013
TC0700008849.hg.1	FOXP2	Multiple_Complex	6.27	7.29	-2.02	6.31E-05	0.0006
TC1600006685.hg.1	ZNF75A	Multiple_Complex	5.66	6.68	-2.02	2.01E-05	0.0002
TC0200013899.hg.1	ZC3H8	Multiple_Complex	8.04	9.05	-2.02	5.11E-05	0.0005
TC1000012164.hg.1	DHX32	Multiple_Complex	7.22	8.24	-2.02	0.0003	0.0021
TC1000008927.hg.1	VTI1A	Multiple_Complex	6.68	7.7	-2.03	9.00E-05	0.0009
TC1700011366.hg.1	INTS2	Multiple_Complex	7.31	8.33	-2.03	0.0001	0.0012
TC0900008811.hg.1	STXBP1	Multiple_Complex	8.49	9.51	-2.03	2.59E-05	0.0003
TC0700010619.hg.1	SCRN1	Multiple_Complex	8.66	9.68	-2.03	2.84E-05	0.0003
TC0100016337.hg.1	XCL2	Coding	4.17	5.19	-2.03	8.33E-06	0.0001
TC1300009933.hg.1	RASA3	Multiple_Complex	8.02	9.04	-2.03	1.21E-05	0.0002
TC0200015264.hg.1	SDPR	Multiple_Complex	6.01	7.03	-2.03	2.15E-05	0.0003
TC0700010621.hg.1	FKBP14	Multiple_Complex	8.21	9.23	-2.03	2.04E-05	0.0002
TC2100008461.hg.1	SPATC1L	Coding	5.66	6.69	-2.03	6.75E-05	0.0007
TC0300006961.hg.1	CMTM8	Coding	7.28	8.3	-2.04	0.0009	0.0061
TC1100011310.hg.1	PC	Multiple_Complex	6.46	7.49	-2.04	0.0002	0.0018
TC0X00008383.hg.1	OCRL	Unassigned	4.01	5.03	-2.04	0.0046	0.0225
TC0500006435.hg.1	PLEKHG4B	Multiple_Complex	5.44	6.47	-2.04	0.0002	0.0019
TC0600006731.hg.1	PRPF4B	Multiple_Complex	8.23	9.26	-2.04	5.45E-05	0.0006
TC0200007803.hg.1	LGALSL	Multiple_Complex	7.41	8.43	-2.04	0.0003	0.0023
TC0X00006799.hg.1	SAT1	Multiple_Complex	9.58	10.61	-2.04	0.0002	0.0016
TC1100010746.hg.1	CELF1	NonCoding	6.03	7.06	-2.04	0.0013	0.0082
TC1000008380.hg.1	LIPK	Coding	3.75	4.78	-2.04	0.0192	0.0699
TC0X00009215.hg.1	EIF1AX	Multiple_Complex	9.49	10.52	-2.04	0.0004	0.0029
TC1100012962.hg.1	AMPD3	Multiple_Complex	5.55	6.58	-2.04	0.0002	0.0016
TC0800011634.hg.1	SNTB1	Multiple_Complex	8.26	9.29	-2.04	0.0003	0.0023
TC0600010060.hg.1	MRPL18	Multiple_Complex	9.26	10.29	-2.04	0.001	0.0065
TC0800008370.hg.1	POLR2K	Multiple_Complex	9.15	10.18	-2.04	6.60E-06	9.51E-05
TC0700012296.hg.1	C7orf60	Multiple_Complex	5.84	6.87	-2.04	1.85E-05	0.0002
TC0100015876.hg.1	ILF2	NonCoding	5.38	6.41	-2.04	0.003	0.0158
TC1200007406.hg.1	IRAK4	Multiple_Complex	6.73	7.76	-2.04	0.0041	0.0204
TC1600011408.hg.1	CKLF	Multiple_Complex	8.99	10.03	-2.05	6.53E-06	9.44E-05
TC0500006822.hg.1	OTULIN	Multiple_Complex	7.68	8.71	-2.05	3.60E-05	0.0004
TC0500010081.hg.1	FAM173B	Unassigned	4.56	5.59	-2.05	0.0009	0.0056
TC0100014579.hg.1	ZRANB2	Multiple_Complex	8.29	9.32	-2.05	0.0021	0.012
TC0400008922.hg.1	LSM6	Multiple_Complex	8.25	9.28	-2.05	0.0001	0.0013
TC0500007966.hg.1	XRCC4	Multiple_Complex	7.56	8.6	-2.05	9.40E-06	0.0001
TC0200007054.hg.1	ABHD1	Multiple_Complex	3.95	4.98	-2.05	9.46E-05	0.0009

TC2000009949.hg.1	STX16	Multiple_Complex	8.78	9.81	-2.05	3.13E-05	0.0004
TC1200010569.hg.1	ASB8	Multiple_Complex	8.82	9.86	-2.05	5.03E-05	0.0005
TC0700013065.hg.1	ABCF2	Multiple_Complex	8.09	9.13	-2.05	0.0001	0.0013
TC1000011720.hg.1	CALHM3	Coding	5.13	6.17	-2.05	1.82E-05	0.0002
TC0700006488.hg.1	GPER1	Coding	6.63	7.67	-2.05	1.59E-05	0.0002
TC1300009895.hg.1	PCID2	Multiple_Complex	8.64	9.68	-2.05	1.63E-05	0.0002
TC0600007092.hg.1	FAM8A1	Multiple_Complex	7.08	8.12	-2.06	1.65E-05	0.0002
TC1700012482.hg.1	SIRT7	Multiple_Complex	7.32	8.36	-2.06	4.78E-05	0.0005
TC1100007384.hg.1	ALKBH3	Multiple_Complex	6.83	7.87	-2.06	5.55E-05	0.0006
TC1000009090.hg.1	BAG3	Coding	9.54	10.59	-2.06	3.56E-05	0.0004
TC1100013140.hg.1	TAF10	Multiple_Complex	9.26	10.3	-2.06	4.65E-05	0.0005
TC1000010909.hg.1	AIFM2	Multiple_Complex	6.85	7.9	-2.06	2.50E-05	0.0003
TC0600010757.hg.1	SLC35B3	Multiple_Complex	5.99	7.04	-2.06	4.20E-05	0.0005
TC1700009248.hg.1	TEX19	Coding	4.84	5.88	-2.07	0.0007	0.0049
TSUnmapped00000351.hg.1	VPS11	NonCoding	6.89	7.93	-2.07	6.26E-05	0.0006
TC0100016356.hg.1	F5	Multiple_Complex	5.55	6.6	-2.07	8.58E-05	0.0008
TC1900012000.hg.1	TEAD2	Multiple_Complex	5.92	6.97	-2.07	1.66E-05	0.0002
TC0X00010136.hg.1	TAF9B	Multiple_Complex	8.25	9.3	-2.07	3.75E-06	5.92E-05
TC0200007249.hg.1	GPATCH11	Multiple_Complex	7.74	8.79	-2.07	8.75E-05	0.0009
TC1000012575.hg.1	ATAD1	Multiple_Complex	9.58	10.63	-2.07	0.0002	0.0016
TC1300007161.hg.1	CDADC1	Multiple_Complex	7.83	8.88	-2.07	5.55E-05	0.0006
TC0400009632.hg.1	FRG1	Multiple_Complex	8.59	9.64	-2.08	0.0005	0.0038
TC1200006890.hg.1	APOLD1	Multiple_Complex	9.79	10.84	-2.08	6.11E-05	0.0006
TC0700007822.hg.1	VKORC1L1	Multiple_Complex	9.11	10.16	-2.08	0.0001	0.001
TC0100014995.hg.1	CNN3	Multiple_Complex	9.78	10.84	-2.08	3.99E-06	6.25E-05
TC0300014093.hg.1	PCYT1A	Multiple_Complex	8.54	9.6	-2.08	4.39E-05	0.0005
TC2000007386.hg.1	PLCG1	Multiple_Complex	7.24	8.3	-2.08	1.73E-05	0.0002
TC0X00006433.hg.1	PLCXD1	Multiple_Complex	5.63	6.69	-2.08	0.0004	0.003
TC1700012285.hg.1	PRR11	Multiple_Complex	8.23	9.29	-2.08	2.38E-05	0.0003
TC1600011565.hg.1	CFDP1	Multiple_Complex	7.7	8.76	-2.08	9.73E-06	0.0001
TC1200006905.hg.1	FAM234B	Multiple_Complex	6.49	7.55	-2.08	9.60E-05	0.0009
TC0900011565.hg.1	ANGPTL2	Multiple_Complex	5.58	6.64	-2.08	5.91E-05	0.0006
TC1900011392.hg.1	TFPT	Coding	7.63	8.69	-2.08	1.25E-05	0.0002
TC1100011157.hg.1	PPP1R14B	Multiple_Complex	10.05	11.11	-2.08	7.82E-06	0.0001
TC2000006899.hg.1	XRN2	Multiple_Complex	9.44	10.5	-2.09	6.72E-05	0.0007
TC0X00011326.hg.1	HPRT1	Multiple_Complex	7.91	8.97	-2.09	6.01E-05	0.0006
TC1400008780.hg.1	NOVA1	Multiple_Complex	3.73	4.79	-2.09	1.68E-05	0.0002
TC0500011398.hg.1	CCNH	Multiple_Complex	8.2	9.26	-2.09	6.23E-05	0.0006
TC2000008268.hg.1	SLC23A2	Multiple_Complex	6.44	7.5	-2.09	2.35E-05	0.0003
TC1800007355.hg.1	C18orf54	Multiple_Complex	5.18	6.24	-2.09	0.0003	0.0025

TC0800010944.hg.1	IMPA1	Multiple_Complex	8	9.07	-2.09	0.0002	0.0014
TC0300009713.hg.1	EPHB3	Multiple_Complex	6.15	7.21	-2.09	0.0001	0.0011
TC2100007402.hg.1	ADARB1	Multiple_Complex	6.91	7.97	-2.09	6.85E-05	0.0007
TC0X00008235.hg.1	LONRF3	Multiple_Complex	7.19	8.25	-2.09	7.72E-05	0.0008
TC1100011684.hg.1	PRKRIR	Multiple_Complex	8.31	9.38	-2.09	1.33E-05	0.0002
TC0600011248.hg.1	ZKSCAN4	Multiple_Complex	3.79	4.86	-2.09	0.0002	0.0017
TC1200010185.hg.1	ITPR2	Multiple_Complex	6.7	7.76	-2.09	3.32E-06	5.33E-05
TC0300013296.hg.1	DNAJC19	Multiple_Complex	7.91	8.98	-2.09	1.61E-05	0.0002
TC1500007919.hg.1	ISL2	Multiple_Complex	7.07	8.13	-2.09	0.0008	0.0055
TC0900012066.hg.1	NSMF	Multiple_Complex	8.05	9.12	-2.1	4.54E-06	6.93E-05
TC1500009363.hg.1	COPS2	Multiple_Complex	9.85	10.92	-2.1	0.0002	0.0014
TC1100012419.hg.1	ZPR1	Multiple_Complex	8.71	9.78	-2.1	2.28E-05	0.0003
TC1900011936.hg.1	ZNF781	Multiple_Complex	4.84	5.91	-2.1	8.43E-06	0.0001
TC1200007070.hg.1	ETNK1	Multiple_Complex	9.02	10.09	-2.1	7.76E-06	0.0001
TC1100007805.hg.1	TMEM216	Multiple_Complex	7.59	8.66	-2.1	2.16E-05	0.0003
TC0600011140.hg.1	HIST1H3F	Coding	7.64	8.71	-2.1	9.21E-06	0.0001
TC1700011308.hg.1	LOC653653	Multiple_Complex	5.27	6.34	-2.1	5.56E-06	8.26E-05
TC2100008536.hg.1	PCBP3	Multiple_Complex	4.62	5.7	-2.1	6.84E-06	9.81E-05
TC1500010846.hg.1	GOLGA8K	Multiple_Complex	6.18	7.25	-2.1	0.0063	0.0289
TC0700007244.hg.1	STARD3NL	Multiple_Complex	8.03	9.1	-2.1	5.90E-06	8.69E-05
TC1000007596.hg.1	TIMM23B	Multiple_Complex	8.67	9.74	-2.1	8.65E-06	0.0001
TC0100014964.hg.1	DNTTIP2	Multiple_Complex	7.68	8.76	-2.11	8.52E-06	0.0001
TSUnmapped00000478.hg.1	ZNF502	Coding	3.5	4.58	-2.11	0.0003	0.0022
TC0X00010804.hg.1	ZNF280C	Multiple_Complex	7.86	8.93	-2.11	7.50E-05	0.0007
TC1100011452.hg.1	FGF3	Coding	6.9	7.98	-2.11	9.88E-05	0.0009
TC0600014060.hg.1	PSMG4	Multiple_Complex	7.68	8.76	-2.11	0.0001	0.0011
TC1600010680.hg.1	SLC7A6OS	Multiple_Complex	9.08	10.16	-2.11	1.11E-05	0.0001
TC0200008063.hg.1	DGUOK	Multiple_Complex	8.72	9.8	-2.12	2.44E-05	0.0003
TC0300014092.hg.1	TM4SF19-TCTEX1D2	Multiple_Complex	5.85	6.93	-2.12	9.75E-06	0.0001
TC0300012857.hg.1	DHX36	Multiple_Complex	8.14	9.22	-2.12	0.0002	0.0019
TC1400007036.hg.1	PRPF39	Multiple_Complex	6.99	8.07	-2.12	0.0011	0.007
TC1200012640.hg.1	BLOC1S1	Multiple_Complex	9.79	10.87	-2.12	0.0002	0.0019
TC1100008904.hg.1	YAP1	Multiple_Complex	8.26	9.34	-2.12	1.25E-05	0.0002
TC1100012208.hg.1	CWF19L2	Multiple_Complex	5.9	6.98	-2.12	0.0003	0.002
TC0X00010462.hg.1	RBM41	Multiple_Complex	7.38	8.46	-2.12	0.0002	0.0019
TC2000009887.hg.1	PLCB1	Multiple_Complex	6.9	7.98	-2.12	2.47E-05	0.0003
TC1200007157.hg.1	REP15	Coding	3.22	4.3	-2.12	8.90E-05	0.0009
TC1100010405.hg.1	LIN7C	Multiple_Complex	9.86	10.94	-2.12	0.0003	0.0022
TC1000007700.hg.1	UBE2D1	Multiple_Complex	9.42	10.5	-2.12	7.95E-06	0.0001
TC1100008955.hg.1	AASDHPPT	Multiple_Complex	9.05	10.13	-2.12	0.0003	0.0024

TC0800008011.hg.1	ZFHX4	Multiple_Complex	4.13	5.21	-2.12	2.30E-05	0.0003
TC0400012396.hg.1	AADAT	Multiple_Complex	7.26	8.34	-2.12	2.41E-05	0.0003
TC0X00006764.hg.1	MBTPS2	Multiple_Complex	8.51	9.6	-2.12	0.0028	0.0148
TC1700012470.hg.1	H3F3B	Multiple_Complex	10.48	11.57	-2.12	0.0003	0.0024
TC2200009273.hg.1	APOBEC3F	Multiple_Complex	5.84	6.92	-2.12	1.12E-05	0.0001
TC2200008881.hg.1	RRP7A	Multiple_Complex	7.3	8.39	-2.12	2.11E-05	0.0003
TC1200010832.hg.1	SMUG1	Multiple_Complex	7.96	9.05	-2.12	9.02E-05	0.0009
TC0100018526.hg.1	ILDR2	Multiple_Complex	4.72	5.8	-2.12	2.16E-05	0.0003
TC0300013988.hg.1	PCBP4	Multiple_Complex	7.62	8.7	-2.13	5.51E-06	8.21E-05
TC0800009872.hg.1	TNFRSF10D	Coding	8.17	9.26	-2.13	7.44E-05	0.0007
TC2200007615.hg.1	RIBC2	Multiple_Complex	5.77	6.86	-2.13	2.07E-05	0.0002
TC1200010874.hg.1	MMP19	Multiple_Complex	5.8	6.89	-2.13	3.95E-05	0.0004
TC1700011451.hg.1	SMURF2	Multiple_Complex	7.97	9.06	-2.13	2.91E-05	0.0003
TC0300013025.hg.1	BCHE	Coding	3.14	4.23	-2.13	1.80E-05	0.0002
TC0X00009842.hg.1	SPIN2A	Coding	5.57	6.66	-2.13	1.02E-05	0.0001
TC0600010867.hg.1	MCUR1	Multiple_Complex	7.32	8.41	-2.14	0.0005	0.0037
TC1900007829.hg.1	GRAMD1A	Multiple_Complex	6.46	7.55	-2.14	1.32E-05	0.0002
TC0700011556.hg.1	HIP1	Multiple_Complex	6.65	7.74	-2.14	1.09E-05	0.0001
TC0X00009213.hg.1	MAP7D2	Multiple_Complex	6.33	7.43	-2.14	4.46E-05	0.0005
TC1300007969.hg.1	ABHD13	Coding	7.4	8.5	-2.14	0.0001	0.001
TC1700011519.hg.1	HELZ	Multiple_Complex	8.24	9.34	-2.14	2.37E-05	0.0003
TC1200007061.hg.1	CMAS	Multiple_Complex	8.54	9.64	-2.14	0.0001	0.0011
TC0800007697.hg.1	CHCHD7	Multiple_Complex	7.23	8.33	-2.14	1.85E-05	0.0002
TC0600009851.hg.1	MTHFD1L	Multiple_Complex	8.96	10.07	-2.15	5.73E-06	8.48E-05
TC0500007699.hg.1	SMN1	Multiple_Complex	9.99	11.1	-2.15	0.0001	0.0011
TC0200016419.hg.1	GPN1	Multiple_Complex	8.15	9.26	-2.15	5.56E-06	8.26E-05
TC1700009489.hg.1	ITGAE	Multiple_Complex	7.61	8.71	-2.15	7.79E-06	0.0001
TC0X00008330.hg.1	XIAP	Multiple_Complex	7.93	9.03	-2.15	0.0001	0.0011
TC0300009179.hg.1	MED12L	Multiple_Complex	3.66	4.76	-2.15	0.0013	0.0081
TC0100011382.hg.1	DYRK3	Multiple_Complex	6.2	7.3	-2.15	0.0002	0.0015
TC1000006891.hg.1	VIM	Multiple_Complex	9.48	10.58	-2.15	1.60E-05	0.0002
TC0700013526.hg.1	FAM126A	Multiple_Complex	5.32	6.43	-2.15	2.82E-06	4.68E-05
TC0400011130.hg.1	PAQR3	Multiple_Complex	8.56	9.66	-2.15	0.0002	0.0015
TC1300008179.hg.1	UPF3A	Multiple_Complex	8.7	9.81	-2.15	3.01E-05	0.0003
TC0600007585.hg.1	MICB	Multiple_Complex	7.76	8.87	-2.15	7.78E-06	0.0001
TC1000009851.hg.1	FRMD4A	Multiple_Complex	6.06	7.17	-2.16	5.93E-06	8.72E-05
TC1500009395.hg.1	TRPM7	Multiple_Complex	7.3	8.41	-2.16	0.0003	0.0025
TC0X00009611.hg.1	SLC38A5	Multiple_Complex	10.33	11.44	-2.16	4.77E-06	7.24E-05
TC0200016578.hg.1	NUP35	Multiple_Complex	8.46	9.57	-2.16	2.12E-05	0.0003
TC1500009386.hg.1	GABPB1	Multiple_Complex	8.18	9.29	-2.16	7.64E-06	0.0001

TC0X00011363.hg.1	ZNF182	Multiple_Complex	6.88	7.99	-2.16	1.39E-05	0.0002
TC1600010869.hg.1	BCAR1	Multiple_Complex	8.91	10.02	-2.16	0.0001	0.001
TC1800008952.hg.1	DSEL	Coding	3.56	4.67	-2.16	0.0004	0.0029
TC0700010247.hg.1	RPA3	Multiple_Complex	7.56	8.67	-2.16	9.90E-05	0.0009
TC1100007467.hg.1	C11orf49	Multiple_Complex	7.22	8.34	-2.16	1.99E-05	0.0002
TC1700011523.hg.1	PSMD12	Multiple_Complex	10.03	11.15	-2.17	2.34E-05	0.0003
TC0700007630.hg.1	CCT6A	Multiple_Complex	10.95	12.07	-2.17	0.0001	0.0012
TC0800006738.hg.1	MTMR9	Multiple_Complex	6.33	7.44	-2.17	6.43E-05	0.0007
TC0400007306.hg.1	N4BP2	Multiple_Complex	6	7.11	-2.17	0.0019	0.0109
TC2000006576.hg.1	PRNP	Multiple_Complex	9.64	10.76	-2.17	1.50E-05	0.0002
TC0X00007158.hg.1	ZNF81	Multiple_Complex	6.86	7.98	-2.17	0.0011	0.0071
TC0300009724.hg.1	VPS8	Multiple_Complex	6.84	7.96	-2.17	6.00E-05	0.0006
TC0X00010211.hg.1	CHM	Multiple_Complex	8.39	9.51	-2.18	3.18E-05	0.0004
TC0600011600.hg.1	TAF11	Multiple_Complex	6.72	7.85	-2.18	1.45E-05	0.0002
TC1100012717.hg.1	PUS3	Coding	7.34	8.46	-2.18	4.77E-05	0.0005
TC0700008897.hg.1	LSM8	Multiple_Complex	8.15	9.28	-2.18	0.0001	0.0011
TC1600010734.hg.1	NOB1	Multiple_Complex	10.6	11.72	-2.18	5.00E-06	7.53E-05
TC0300013831.hg.1	SEMA3B	Multiple_Complex	6.25	7.38	-2.18	2.32E-05	0.0003
TC0X00009836.hg.1	SPIN3	Multiple_Complex	6.9	8.03	-2.18	5.61E-05	0.0006
TC1100007394.hg.1	CD82	Multiple_Complex	7.54	8.66	-2.18	1.12E-05	0.0001
TC1000008063.hg.1	ADK	Multiple_Complex	8.85	9.98	-2.19	1.00E-05	0.0001
TC1000007930.hg.1	PALD1	Multiple_Complex	7.78	8.91	-2.19	2.03E-05	0.0002
TC0X00011317.hg.1	TCEAL1	Multiple_Complex	8.43	9.56	-2.19	8.77E-06	0.0001
TC0700009232.hg.1	BPGM	Coding	7.57	8.7	-2.19	3.42E-05	0.0004
TC1000012511.hg.1	PRAP1	Multiple_Complex	5.56	6.69	-2.19	5.04E-05	0.0005
TC1700012279.hg.1	HOXB-AS3	Multiple_Complex	5.41	6.54	-2.19	1.15E-05	0.0002
TC1200010597.hg.1	DDX23	Multiple_Complex	9.76	10.88	-2.19	1.17E-05	0.0002
TC0700009079.hg.1	TSPAN33	Multiple_Complex	5.86	6.99	-2.19	4.02E-05	0.0004
TC1200007809.hg.1	GDF11	Coding	5.37	6.49	-2.19	0.0042	0.0209
TC1200012739.hg.1	PARP11	Multiple_Complex	6.33	7.46	-2.19	0.0044	0.0218
TC1000010699.hg.1	IPMK	Multiple_Complex	8.79	9.92	-2.19	0.0006	0.0044
TC0700010501.hg.1	DFNA5	Multiple_Complex	3.63	4.77	-2.19	1.21E-05	0.0002
TC0X00009254.hg.1	APOO	Multiple_Complex	7.81	8.94	-2.19	1.67E-05	0.0002
TC0X00010954.hg.1	FGF13	Multiple_Complex	5.93	7.06	-2.2	0.0001	0.001
TC1700012400.hg.1	KRT39	Multiple_Complex	4.17	5.3	-2.2	2.15E-05	0.0003
TC0700012602.hg.1	CEP41	Multiple_Complex	5.26	6.39	-2.2	1.01E-05	0.0001
TC0X00010442.hg.1	TMSB15B	Coding	3.27	4.41	-2.2	6.95E-06	9.91E-05
TC2000009247.hg.1	SPATA25	Coding	4.98	6.12	-2.2	0.0237	0.0825
TC1000012580.hg.1	ARHGAP19	Multiple_Complex	7.18	8.32	-2.2	0.0002	0.0017
TC1100011374.hg.1	KMT5B	Multiple_Complex	8.73	9.86	-2.2	0.0001	0.001

TC0500007457.hg.1	SETD9	Multiple_Complex	6.97	8.1	-2.2	9.91E-05	0.0009
TC0200016740.hg.1	ARL5A	Multiple_Complex	7.93	9.07	-2.2	1.13E-05	0.0001
TC1300007706.hg.1	MIR17HG	Multiple_Complex	4.64	5.78	-2.2	0.0003	0.002
TC1700008703.hg.1	NOL11	Multiple_Complex	8.71	9.85	-2.2	6.12E-06	8.95E-05
TC0100010532.hg.1	XCL1	Coding	4.23	5.37	-2.2	1.92E-05	0.0002
TC0300013933.hg.1	THUMPD3-AS1	Multiple_Complex	5.83	6.97	-2.2	5.71E-05	0.0006
TC0100017118.hg.1	YOD1	Multiple_Complex	7.03	8.17	-2.21	1.82E-05	0.0002
TC1200011719.hg.1	CCDC53	Multiple_Complex	8.49	9.63	-2.21	2.65E-05	0.0003
TC0200010511.hg.1	NBEAL1	Multiple_Complex	4.62	5.77	-2.21	6.74E-05	0.0007
TC0200010152.hg.1	UBE2E3	Multiple_Complex	8.1	9.24	-2.22	3.80E-06	5.99E-05
TC1200008686.hg.1	CHST11	Unassigned	4.42	5.57	-2.22	0.0001	0.0012
TC1500007967.hg.1	DNAJA4	Multiple_Complex	8.19	9.34	-2.22	1.53E-05	0.0002
TSUnmapped00000122.hg.1	CCDC84	Coding	6.09	7.24	-2.22	0.0018	0.0103
TC1700010914.hg.1	ARL17B	Multiple_Complex	7.15	8.3	-2.22	0.0011	0.0071
TC1100012545.hg.1	TRIM29	Multiple_Complex	7.47	8.62	-2.22	4.35E-06	6.70E-05
TC2000010034.hg.1	ZNF512B	Multiple_Complex	5.52	6.67	-2.22	0.0001	0.0012
TC0600012875.hg.1	FYN	Multiple_Complex	8.72	9.87	-2.22	8.83E-06	0.0001
TC1000010768.hg.1	EGR2	Multiple_Complex	6.46	7.61	-2.22	1.29E-05	0.0002
TC1100009298.hg.1	GRIK4	Multiple_Complex	5.25	6.4	-2.22	0.004	0.0199
TC0200015216.hg.1	ORMDL1	Multiple_Complex	7.92	9.07	-2.22	5.21E-06	7.81E-05
TC0100013349.hg.1	RSRP1	Multiple_Complex	7.23	8.38	-2.22	4.86E-06	7.35E-05
TC2200009278.hg.1	RBX1	Multiple_Complex	8.85	10	-2.22	7.84E-05	0.0008
TC0300011834.hg.1	FILIP1L	Multiple_Complex	8.87	10.02	-2.22	8.31E-06	0.0001
TC1100009301.hg.1	TBCEL	Multiple_Complex	7.6	8.75	-2.22	0.0002	0.0015
TC0100011200.hg.1	TIMM17A	Multiple_Complex	9.56	10.71	-2.22	7.63E-06	0.0001
TC1200009997.hg.1	GRIN2B	Multiple_Complex	6.23	7.38	-2.22	8.70E-06	0.0001
TC0200016618.hg.1	RNASEH1	Multiple_Complex	8.25	9.4	-2.22	1.12E-05	0.0001
TC1200010157.hg.1	LYRM5	NonCoding	4.97	6.12	-2.22	0.0001	0.0013
TC2200007068.hg.1	SLC35E4	Multiple_Complex	5.29	6.45	-2.23	8.54E-05	0.0008
TC0Y00006858.hg.1	VAMP7	Multiple_Complex	8.28	9.43	-2.23	0.0001	0.001
TC1000009145.hg.1	BTBD16	Multiple_Complex	5.36	6.51	-2.23	0.0001	0.0011
TC0400011383.hg.1	TSPAN5	Multiple_Complex	8.52	9.68	-2.23	6.82E-06	9.79E-05
TC0X00007919.hg.1	SRPX2	Multiple_Complex	4.92	6.07	-2.23	2.35E-05	0.0003
TC1000011713.hg.1	PCGF6	Multiple_Complex	7.29	8.44	-2.23	1.48E-05	0.0002
TC0600007610.hg.1	MSH5	Multiple_Complex	5.09	6.25	-2.23	4.67E-05	0.0005
TC0700008626.hg.1	ORAI2	Multiple_Complex	6.57	7.73	-2.23	6.47E-06	9.38E-05
TC1300008668.hg.1	LHFP	Multiple_Complex	5.61	6.77	-2.23	6.87E-06	9.83E-05
TC2000006571.hg.1	SMOX	Multiple_Complex	7.28	8.44	-2.23	4.10E-06	6.37E-05
TC1300007228.hg.1	WDFY2	Multiple_Complex	7.47	8.63	-2.23	2.71E-05	0.0003
TC0500006730.hg.1	CCT5	Multiple_Complex	10.26	11.42	-2.24	3.98E-06	6.24E-05

TC2000008985.hg.1	RBM39	Multiple_Complex	9.01	10.17	-2.24	3.20E-05	0.0004
TC0300010035.hg.1	SENP5	Multiple_Complex	7.97	9.13	-2.24	4.83E-05	0.0005
TC0X00008009.hg.1	TCEAL4	Multiple_Complex	8.08	9.25	-2.24	4.67E-05	0.0005
TC1000006611.hg.1	AKR1C4	Multiple_Complex	4.12	5.28	-2.24	5.31E-05	0.0006
TSUnmapped00000299.hg.1	TRAPPC4	NonCoding	5.15	6.31	-2.24	0.0019	0.0107
TC0600007480.hg.1	HLA-F	Multiple_Complex	8.2	9.37	-2.24	3.13E-06	5.08E-05
TC1400009299.hg.1	C14orf105	Multiple_Complex	3.48	4.65	-2.25	1.51E-05	0.0002
TC0300011853.hg.1	SENP7	Multiple_Complex	5.29	6.46	-2.25	0.0017	0.0097
TC1700011476.hg.1	GNA13	Multiple_Complex	8.94	10.11	-2.25	3.74E-05	0.0004
TC1100013075.hg.1	DDIAS	Multiple_Complex	7.41	8.58	-2.25	8.04E-06	0.0001
TC0400007460.hg.1	OCIAD1	Unassigned	6.12	7.29	-2.25	5.61E-05	0.0006
TC0400006661.hg.1	STK32B	Multiple_Complex	4.93	6.1	-2.25	1.20E-05	0.0002
TC1700011233.hg.1	MRPS23	Multiple_Complex	7.73	8.9	-2.25	5.63E-05	0.0006
TC1000006859.hg.1	SUV39H2	Multiple_Complex	7.33	8.5	-2.25	9.70E-06	0.0001
TC0600008509.hg.1	KCNQ5	Multiple_Complex	4.3	5.47	-2.25	9.37E-05	0.0009
TC2200008257.hg.1	LRP5L	Multiple_Complex	6.41	7.59	-2.26	1.48E-05	0.0002
TC0600014257.hg.1	HLA-C	Multiple_Complex	8.17	9.34	-2.26	2.06E-06	3.55E-05
TC2000008213.hg.1	DDRGK1	Multiple_Complex	7.2	8.38	-2.26	3.16E-06	5.12E-05
TC0200012994.hg.1	SNRPG	Multiple_Complex	8.48	9.66	-2.26	3.41E-06	5.44E-05
TC1800008287.hg.1	ANKRD29	Multiple_Complex	4.57	5.75	-2.26	0.0003	0.0025
TC1300007032.hg.1	SERP2	Coding	5.16	6.34	-2.27	4.78E-05	0.0005
TC0900006481.hg.1	VLDLR	Multiple_Complex	4.92	6.1	-2.27	1.07E-05	0.0001
TC0200015971.hg.1	SLC16A14	Coding	5.61	6.8	-2.27	4.57E-06	6.96E-05
TC1000012497.hg.1	BORCS7-ASMT	Multiple_Complex	3.89	5.08	-2.27	4.51E-06	6.90E-05
TC1600007829.hg.1	CYLD	Multiple_Complex	6.27	7.46	-2.27	0.0004	0.0033
TC0X00006631.hg.1	TMSB4X	Multiple_Complex	11.57	12.75	-2.28	1.86E-05	0.0002
TC0500009264.hg.1	ADRA1B	Coding	5.76	6.95	-2.28	5.61E-05	0.0006
TC2000009242.hg.1	WFDC3	Multiple_Complex	5.66	6.84	-2.28	0.0003	0.0022
TC0700010678.hg.1	LSM5	Multiple_Complex	8.41	9.6	-2.28	5.96E-06	8.74E-05
TC1100013163.hg.1	APIP	Multiple_Complex	8.53	9.72	-2.28	3.25E-06	5.24E-05
TC1400010692.hg.1	APOPT1	Multiple_Complex	7.72	8.91	-2.28	3.08E-06	5.03E-05
TC0X00008470.hg.1	PHF6	Multiple_Complex	9.56	10.76	-2.29	0.0001	0.0012
TC1700012309.hg.1	METTL23	Multiple_Complex	8.19	9.38	-2.29	4.91E-05	0.0005
TC1900010531.hg.1	ZNF461	Multiple_Complex	6.68	7.87	-2.29	7.60E-05	0.0008
TC0600007307.hg.1	BTN2A1	Multiple_Complex	6.2	7.39	-2.29	0.0002	0.0014
TC1200007326.hg.1	ALG10B	Multiple_Complex	6.78	7.98	-2.29	1.18E-05	0.0002
TC0700009700.hg.1	NUB1	Multiple_Complex	7.79	8.98	-2.29	9.47E-06	0.0001
TC1800007667.hg.1	TIMM21	Multiple_Complex	7.99	9.18	-2.29	7.26E-06	0.0001
TC0X00009153.hg.1	CTPS2	Multiple_Complex	8.84	10.03	-2.29	4.57E-06	6.96E-05
TC0600011162.hg.1	ZNF322	Multiple_Complex	6.25	7.45	-2.29	0.0083	0.0362

TC0500012340.hg.1	NR3C1	Multiple_Complex	3.89	5.09	-2.3	1.56E-05	0.0002
TC0100011953.hg.1	ARV1	Multiple_Complex	7.84	9.04	-2.3	7.32E-06	0.0001
TC1200010833.hg.1	CBX5	Multiple_Complex	9.22	10.42	-2.3	3.81E-05	0.0004
TC0800010421.hg.1	ST18	Multiple_Complex	3.37	4.58	-2.31	0.0001	0.0011
TC0300012918.hg.1	CCNL1	Multiple_Complex	8.72	9.93	-2.31	2.41E-05	0.0003
TC0500010197.hg.1	ZNF622	Multiple_Complex	6.65	7.85	-2.31	2.16E-05	0.0003
TC0700013337.hg.1	UMAD1	Multiple_Complex	6.96	8.17	-2.31	2.33E-05	0.0003
TC0500007421.hg.1	SKIV2L2	Multiple_Complex	8.29	9.5	-2.31	0.0007	0.0048
TC0800008510.hg.1	OXR1	Multiple_Complex	8.13	9.34	-2.32	4.82E-05	0.0005
TC0200016582.hg.1	NABP1	Multiple_Complex	6.99	8.2	-2.32	3.49E-06	5.54E-05
TC1000011168.hg.1	ZCCHC24	Coding	5.29	6.51	-2.32	0.0005	0.0036
TC1200007052.hg.1	GOLT1B	Multiple_Complex	9.36	10.57	-2.32	3.91E-05	0.0004
TC2000006551.hg.1	ATRN	Multiple_Complex	7.33	8.55	-2.32	6.66E-06	9.59E-05
TC1000007103.hg.1	RAB18	Multiple_Complex	8.99	10.2	-2.32	5.03E-06	7.58E-05
TC0300013824.hg.1	TDGF1	Multiple_Complex	6.77	7.98	-2.32	2.13E-06	3.66E-05
TC0600014069.hg.1	TMEM14B	Multiple_Complex	9.03	10.25	-2.32	3.03E-05	0.0003
TC1100012012.hg.1	CWC15	Multiple_Complex	7.13	8.34	-2.32	5.46E-05	0.0006
TC0500012070.hg.1	CDKN2AIPNL	Coding	9.18	10.4	-2.32	2.25E-06	3.84E-05
TC0500006614.hg.1	ICE1	Multiple_Complex	7.5	8.72	-2.32	0.0005	0.0035
TC1000010575.hg.1	ARHGAP22	Multiple_Complex	4.71	5.92	-2.32	4.80E-05	0.0005
TC0100018555.hg.1	LEMD1	Multiple_Complex	4.45	5.67	-2.33	1.21E-06	2.26E-05
TC0900008865.hg.1	CERCAM	Multiple_Complex	5.42	6.64	-2.33	7.29E-05	0.0007
TC0100010218.hg.1	KIRREL	Multiple_Complex	6.06	7.28	-2.33	2.49E-05	0.0003
TC0700009233.hg.1	BPGM	Unassigned	5.1	6.32	-2.33	0.0004	0.003
TC1200008474.hg.1	SOCS2	Coding	5.56	6.78	-2.33	0.0003	0.0022
TC0700011780.hg.1	PEX1	Multiple_Complex	6.38	7.61	-2.33	2.84E-05	0.0003
TC0X00006686.hg.1	GRPR	Coding	4.59	5.82	-2.34	1.14E-05	0.0002
TC0100017018.hg.1	ETNK2	Multiple_Complex	4.13	5.35	-2.34	5.75E-06	8.50E-05
TC2000008379.hg.1	MKKS	Multiple_Complex	7.86	9.09	-2.34	7.39E-06	0.0001
TC0700007831.hg.1	TPST1	Multiple_Complex	3.69	4.92	-2.34	3.50E-05	0.0004
TC0400007933.hg.1	ANXA3	Multiple_Complex	8.71	9.94	-2.34	3.05E-05	0.0003
TC1600011007.hg.1	MPHOSPH6	Multiple_Complex	8.64	9.87	-2.34	8.27E-06	0.0001
TC0600013409.hg.1	HIVEP2	Multiple_Complex	5.31	6.54	-2.34	0.0004	0.0032
TC0800007978.hg.1	LY96	Coding	3.4	4.63	-2.34	0.0004	0.0029
TC0600006864.hg.1	RIOK1	Multiple_Complex	8.23	9.46	-2.35	0.0002	0.0019
TC0100009116.hg.1	RWDD3	Multiple_Complex	6.47	7.7	-2.35	2.79E-05	0.0003
TC0800008028.hg.1	PKIA	Multiple_Complex	3.95	5.18	-2.35	9.95E-05	0.0009
TC0X00008844.hg.1	IKBKG	Multiple_Complex	6.45	7.68	-2.35	1.60E-05	0.0002
TC1800006679.hg.1	NAPG	Multiple_Complex	7.77	9.01	-2.35	3.36E-06	5.38E-05
TC2200007416.hg.1	FAM83F	Multiple_Complex	7.33	8.56	-2.35	5.79E-06	8.54E-05

TC0700013603.hg.1	RASA4	Multiple_Complex	5.08	6.31	-2.35	1.98E-05	0.0002
TC1000007875.hg.1	DDX50	Multiple_Complex	8.55	9.78	-2.35	1.26E-05	0.0002
TC1700008984.hg.1	SEC14L1	Multiple_Complex	7.83	9.06	-2.35	4.16E-06	6.45E-05
TC2200009275.hg.1	ADSL	Multiple_Complex	8.57	9.8	-2.36	2.15E-06	3.70E-05
TC0400012905.hg.1	QDPR	Multiple_Complex	6.69	7.93	-2.36	9.16E-05	0.0009
TC1700009042.hg.1	AFMID	Multiple_Complex	7.58	8.82	-2.36	0.0006	0.0044
TC1700008734.hg.1	AMZ2	Multiple_Complex	8.69	9.93	-2.36	4.04E-06	6.31E-05
TC1200006450.hg.1	WNK1	Multiple_Complex	7.92	9.16	-2.36	2.90E-05	0.0003
TC0Y00006642.hg.1	TMSB4Y	Coding	9.92	11.16	-2.37	1.69E-06	3.01E-05
TC0200006891.hg.1	RHOB	Coding	7.94	9.18	-2.37	8.25E-06	0.0001
TC1300009979.hg.1	UCHL3	Multiple_Complex	9.43	10.67	-2.37	1.10E-06	2.08E-05
TC0X00008198.hg.1	PLS3	Multiple_Complex	8.48	9.73	-2.37	5.53E-06	8.23E-05
TC1000010844.hg.1	RUFY2	Multiple_Complex	7.17	8.42	-2.37	0.0007	0.0046
TC2000007476.hg.1	STK4	Multiple_Complex	8.06	9.31	-2.37	1.25E-05	0.0002
TC0X00008884.hg.1	VAMP7	Multiple_Complex	7.98	9.22	-2.37	4.33E-05	0.0005
TC0500013283.hg.1	BRD9	Multiple_Complex	7.36	8.61	-2.38	1.43E-06	2.62E-05
TC1700008820.hg.1	C17orf80	Multiple_Complex	7.75	9	-2.38	1.35E-05	0.0002
TC0900011148.hg.1	CTNNAL1	Multiple_Complex	7.2	8.45	-2.38	2.65E-06	4.41E-05
TC0300014095.hg.1	TM4SF19	Multiple_Complex	5.05	6.3	-2.38	2.27E-05	0.0003
TC0600011379.hg.1	MDC1	Multiple_Complex	6.67	7.92	-2.38	6.99E-07	1.44E-05
TC1800006710.hg.1	CHMP1B	Multiple_Complex	8.63	9.88	-2.38	7.08E-06	0.0001
TC0500006803.hg.1	TRIO	Multiple_Complex	6.97	8.23	-2.38	1.15E-06	2.17E-05
TC0100008752.hg.1	TYW3	Multiple_Complex	8.01	9.26	-2.39	9.54E-07	1.85E-05
TC2000007251.hg.1	MYL9	Multiple_Complex	7.81	9.07	-2.39	2.21E-05	0.0003
TC1500007975.hg.1	IREB2	Multiple_Complex	7.95	9.21	-2.39	2.28E-05	0.0003
TC0900007863.hg.1	CKS2	Multiple_Complex	9.41	10.67	-2.39	2.06E-05	0.0002
TC1700011298.hg.1	TUBD1	Multiple_Complex	6.68	7.94	-2.39	7.03E-06	0.0001
TC1100012948.hg.1	IFITM2	Multiple_Complex	8.81	10.08	-2.4	4.78E-06	7.26E-05
TC1600008454.hg.1	GABARAPL2	Multiple_Complex	7.46	8.73	-2.4	0.0001	0.0011
TC1200007881.hg.1	INHBE	Multiple_Complex	5.71	6.98	-2.4	6.49E-07	1.35E-05
TC0600009744.hg.1	RAB32	Multiple_Complex	6.01	7.27	-2.4	7.75E-06	0.0001
TC0500007691.hg.1	SMN1	Multiple_Complex	9.8	11.06	-2.41	5.92E-05	0.0006
TC0700008292.hg.1	STEAP1	Multiple_Complex	8.36	9.63	-2.41	2.27E-06	3.87E-05
TC0X00008599.hg.1	SPANXB1	Coding	5.55	6.81	-2.41	3.21E-06	5.18E-05
TC0500013322.hg.1	NAIP	Multiple_Complex	6.03	7.3	-2.41	9.89E-05	0.0009
TC0200013607.hg.1	TXNDC9	Multiple_Complex	6.87	8.14	-2.41	3.76E-06	5.92E-05
TC1600007803.hg.1	CNEP1R1	Multiple_Complex	7.28	8.55	-2.41	3.12E-06	5.08E-05
TC2000009250.hg.1	PLTP	Multiple_Complex	6.05	7.32	-2.41	1.75E-06	3.11E-05
TC0700013391.hg.1	NSUN5P1	Multiple_Complex	7.75	9.02	-2.41	1.99E-06	3.46E-05
TC1100011930.hg.1	CHORDC1	Multiple_Complex	11.05	12.33	-2.42	6.14E-06	8.97E-05

TC0X00007587.hg.1	PIN4		Multiple_Complex	9.66	10.93	-2.42	4.34E-06	6.69E-05
TC0200015082.hg.1	FKBP7		Multiple_Complex	6.53	7.8	-2.42	1.20E-06	2.26E-05
TC1700009043.hg.1	BIRC5		Multiple_Complex	7.95	9.23	-2.42	2.77E-05	0.0003
TC0X00008404.hg.1	SLC25A14		Multiple_Complex	7.02	8.3	-2.42	4.58E-06	6.97E-05
TC1600008141.hg.1	CBFB		Multiple_Complex	10.52	11.8	-2.43	4.41E-06	6.77E-05
TC1700010997.hg.1	HOXB9		Multiple_Complex	6.47	7.75	-2.43	2.18E-06	3.74E-05
TC0100018323.hg.1	IER5		Coding	6.71	8	-2.44	0.0001	0.0012
TC0500011528.hg.1	LIX1		Coding	5.11	6.4	-2.44	3.34E-06	5.36E-05
TC0300013911.hg.1	DNAJB11		Multiple_Complex	9.3	10.59	-2.44	3.22E-06	5.19E-05
TC0600014098.hg.1	ATAT1		Multiple_Complex	5.31	6.6	-2.44	2.33E-05	0.0003
TC1700008995.hg.1		sep-09	Multiple_Complex	8.57	9.86	-2.45	1.13E-06	2.14E-05
TC0700011383.hg.1	SBDS		Multiple_Complex	9.48	10.77	-2.45	2.42E-05	0.0003
TC1600011573.hg.1	GCSH		Multiple_Complex	9.53	10.82	-2.45	2.91E-06	4.81E-05
TC1000008363.hg.1	PTEN		Multiple_Complex	9.56	10.85	-2.45	2.45E-06	4.12E-05
TC0500010932.hg.1	SGTB		Multiple_Complex	7.13	8.42	-2.45	1.68E-05	0.0002
TC1700006587.hg.1	EMC6		Coding	8.45	9.75	-2.45	1.19E-05	0.0002
TC0400011175.hg.1	TMEM150C		Multiple_Complex	4.94	6.24	-2.45	6.86E-06	9.83E-05
TC0600009597.hg.1	TNFAIP3		Multiple_Complex	6.18	7.47	-2.45	2.45E-06	4.13E-05
TSUnmapped00000246.hg.1	CCDC84		NonCoding	7.64	8.94	-2.46	1.27E-06	2.37E-05
TC1200007295.hg.1	ALG10		Multiple_Complex	6.93	8.23	-2.46	1.45E-05	0.0002
TC0100016263.hg.1	TMCO1		Multiple_Complex	7.76	9.06	-2.46	1.14E-06	2.16E-05
TC1100008651.hg.1	TMEM126B		Coding	8.85	10.15	-2.46	1.85E-05	0.0002
TC2000006694.hg.1	SLX4IP		Multiple_Complex	7.6	8.9	-2.46	7.84E-07	1.57E-05
TC0900012153.hg.1	MSANTD3		Multiple_Complex	7.68	8.98	-2.46	2.20E-06	3.76E-05
TC0300013934.hg.1	EMC3		Multiple_Complex	7.66	8.96	-2.46	1.56E-06	2.82E-05
TC2200009246.hg.1	ZNRF3		Multiple_Complex	7.54	8.84	-2.47	7.07E-06	0.0001
TC1600008226.hg.1	NIP7		Multiple_Complex	10.29	11.59	-2.47	2.44E-06	4.12E-05
TC1700008603.hg.1	CEP95		Multiple_Complex	8	9.31	-2.47	0.0001	0.001
TC0100015586.hg.1	PDZK1		Multiple_Complex	5.34	6.65	-2.47	3.59E-05	0.0004
TC2200009248.hg.1	KREMEN1		Multiple_Complex	7.79	9.1	-2.47	6.56E-06	9.48E-05
TC1400009151.hg.1	TRIM9		Multiple_Complex	4.07	5.38	-2.47	5.37E-06	8.02E-05
TC0X00009250.hg.1	ACOT9		Multiple_Complex	8.22	9.53	-2.48	2.64E-06	4.40E-05
TC1700008438.hg.1	RAD51C		Multiple_Complex	8.55	9.86	-2.48	1.30E-05	0.0002
TC1300009714.hg.1	ARGLU1		Multiple_Complex	8.42	9.73	-2.48	8.80E-06	0.0001
TC0X00011313.hg.1	GPRASP2		Multiple_Complex	4.4	5.72	-2.48	1.08E-05	0.0001
TC1600010756.hg.1	ST3GAL2		Multiple_Complex	6.02	7.33	-2.48	7.09E-06	0.0001
TC0600008255.hg.1	EFHC1		Multiple_Complex	5.96	7.27	-2.49	0.0009	0.0061
TC0100016016.hg.1	NES		Multiple_Complex	6.92	8.24	-2.49	9.92E-07	1.91E-05
TSUnmapped00000268.hg.1	CCDC84		NonCoding	5.06	6.38	-2.49	1.19E-06	2.24E-05
TC1900011759.hg.1	APOC1		Multiple_Complex	4.19	5.51	-2.49	0.0002	0.0018

TC0600007617.hg.1	C6orf48	Multiple_Complex	8.36	9.68	-2.49	1.10E-05	0.0001
TC0200011237.hg.1	COPS8	Multiple_Complex	9.51	10.83	-2.5	1.87E-06	3.28E-05
TC0400012934.hg.1	SDAD1	Multiple_Complex	8.83	10.15	-2.5	5.71E-07	1.20E-05
TC0300008142.hg.1	TBC1D23	Multiple_Complex	8.92	10.24	-2.5	1.94E-06	3.38E-05
TCUn_GL000218v100006433.hg.1	LOC389834	Multiple_Complex	4.89	6.22	-2.51	5.22E-06	7.82E-05
TC0300011314.hg.1	C3orf67	Multiple_Complex	4.38	5.7	-2.51	1.47E-05	0.0002
TC0400012814.hg.1	CXCL6	Multiple_Complex	6.32	7.65	-2.51	9.35E-07	1.82E-05
TC0X00006681.hg.1	ZRSR2	Multiple_Complex	6.2	7.53	-2.51	2.96E-06	4.87E-05
TC0100016321.hg.1	GCSH	Multiple_Complex	8.29	9.62	-2.51	1.42E-06	2.61E-05
TC0700009680.hg.1	TMEM176A	Multiple_Complex	3.69	5.02	-2.52	3.01E-06	4.94E-05
TC0300012676.hg.1	PCOLCE2	Multiple_Complex	5.58	6.92	-2.52	1.31E-06	2.42E-05
TC0200012499.hg.1	MCFD2	Multiple_Complex	8.03	9.36	-2.52	6.47E-06	9.38E-05
TC0600011130.hg.1	HIST1H4C	NonCoding	9.32	10.65	-2.52	0.0003	0.0022
TC0800011646.hg.1	HAS2	Multiple_Complex	6.4	7.73	-2.53	0.0011	0.007
TC0700011692.hg.1	KIAA1324L	Multiple_Complex	7.11	8.45	-2.53	6.87E-06	9.83E-05
TC0600007207.hg.1	GMNN	Multiple_Complex	8.31	9.64	-2.53	3.91E-05	0.0004
TC1900011750.hg.1	ZNF155	Multiple_Complex	5.06	6.4	-2.53	4.30E-07	9.47E-06
TC1400007688.hg.1	FCF1	Multiple_Complex	8.51	9.85	-2.53	7.38E-05	0.0007
TC0700012596.hg.1	TMEM209	Multiple_Complex	8.22	9.56	-2.54	2.01E-05	0.0002
TC0100015471.hg.1	ZNF697	Multiple_Complex	6.07	7.42	-2.54	4.06E-06	6.33E-05
TC0700010443.hg.1	STEAP1B	Multiple_Complex	7.81	9.15	-2.54	2.94E-05	0.0003
TC0400012252.hg.1	PPID	Multiple_Complex	9.48	10.83	-2.54	7.22E-05	0.0007
TC2000008666.hg.1	NAPB	Multiple_Complex	7.03	8.37	-2.54	5.62E-07	1.19E-05
TC1200007693.hg.1	IGFBP6	Multiple_Complex	6.58	7.93	-2.55	9.61E-06	0.0001
TC1700011281.hg.1	SKA2	Multiple_Complex	9.4	10.75	-2.55	1.42E-06	2.60E-05
TC1200010198.hg.1	ASUN	Multiple_Complex	9.59	10.94	-2.55	1.57E-06	2.82E-05
TC2100007263.hg.1	PDE9A	Multiple_Complex	5.6	6.95	-2.55	8.37E-07	1.66E-05
TC1400006791.hg.1	FOXG1	Coding	5.55	6.9	-2.55	1.38E-05	0.0002
TC0500012599.hg.1	ADAM19	Multiple_Complex	6.17	7.53	-2.56	4.33E-07	9.52E-06
TC0700008427.hg.1	DLX6	Multiple_Complex	5.02	6.38	-2.56	6.72E-06	9.66E-05
TC0X00009185.hg.1	SCML2	Multiple_Complex	3.9	5.25	-2.56	0.001	0.0065
TC0600007138.hg.1	E2F3	Multiple_Complex	6.13	7.49	-2.56	2.49E-05	0.0003
TC0500009837.hg.1	SLC12A7	Multiple_Complex	8.44	9.8	-2.56	2.68E-07	6.43E-06
TC0200006936.hg.1	KLHL29	Multiple_Complex	4.72	6.07	-2.56	1.26E-05	0.0002
TC1000011964.hg.1	RAB11FIP2	Multiple_Complex	7.63	8.99	-2.57	1.67E-06	2.99E-05
TC0200007717.hg.1	AHSA2	Multiple_Complex	6.61	7.97	-2.57	0.0004	0.0028
TC1400009122.hg.1	VCPKMT	Multiple_Complex	6.91	8.27	-2.57	6.89E-05	0.0007
TC0X00008526.hg.1	FHL1	Multiple_Complex	6.02	7.38	-2.57	3.06E-07	7.18E-06
TC0100015445.hg.1	TBX15	Multiple_Complex	4.09	5.46	-2.57	3.44E-06	5.49E-05
TC0X00010836.hg.1	RAP2C	Multiple_Complex	8.11	9.48	-2.58	3.53E-06	5.61E-05

TC2000008310.hg.1	TRMT6	Multiple_Complex	9.2	10.56	-2.58	3.46E-06	5.51E-05
TC0100018344.hg.1	LYPLAL1	Multiple_Complex	7.87	9.24	-2.58	6.04E-07	1.27E-05
TC1000010113.hg.1	ANKRD26	Multiple_Complex	5.53	6.9	-2.58	3.64E-05	0.0004
TC0500011333.hg.1	TMEM167A	Multiple_Complex	9.03	10.4	-2.58	8.81E-06	0.0001
TC1700007949.hg.1	ARL4D	Coding	5.14	6.51	-2.58	7.35E-05	0.0007
TC1000007272.hg.1	CREM	Multiple_Complex	7.09	8.46	-2.59	1.53E-06	2.76E-05
TC1100008480.hg.1	UVRAG	Multiple_Complex	7.63	9	-2.59	1.41E-06	2.58E-05
TC1000012496.hg.1	AS3MT	Multiple_Complex	4.18	5.56	-2.59	3.13E-06	5.08E-05
TC0900008851.hg.1	DNM1	Multiple_Complex	7.98	9.36	-2.59	1.45E-06	2.64E-05
TC1400007500.hg.1	ARG2	Multiple_Complex	8.16	9.54	-2.59	1.58E-06	2.84E-05
TC1000008974.hg.1	VWA2	Multiple_Complex	6.37	7.75	-2.59	9.60E-07	1.86E-05
TC1700008566.hg.1	MAP3K3	Multiple_Complex	6.23	7.6	-2.6	9.11E-06	0.0001
TC0100008114.hg.1	UROD	Multiple_Complex	8.77	10.15	-2.6	6.19E-06	9.04E-05
TC0100015550.hg.1	FAM72C	Multiple_Complex	8.57	9.95	-2.6	2.22E-06	3.80E-05
TC0500008784.hg.1	REEP2	Multiple_Complex	5.92	7.3	-2.6	8.35E-06	0.0001
TC1700007571.hg.1	ZNF830	Coding	7.4	8.78	-2.61	1.38E-06	2.53E-05
TC1100011737.hg.1	NDUFC2-KCTD14	Multiple_Complex	9.46	10.84	-2.61	1.10E-05	0.0001
TC1200010250.hg.1	ERGIC2	Multiple_Complex	8.51	9.89	-2.61	2.93E-06	4.83E-05
TC0800009600.hg.1	FAM167A	Multiple_Complex	4.71	6.09	-2.61	2.05E-05	0.0002
TC2100008496.hg.1	SLC5A3	Multiple_Complex	8.21	9.6	-2.62	1.77E-05	0.0002
TC2000009447.hg.1	DPM1	Multiple_Complex	9.24	10.62	-2.62	2.57E-06	4.31E-05
TC0700009386.hg.1	TMEM178B	Multiple_Complex	5.93	7.32	-2.62	9.15E-07	1.80E-05
TC2200009274.hg.1	APOBEC3G	Multiple_Complex	4.25	5.64	-2.62	0.0002	0.0015
TC1000010849.hg.1	SLC25A16	Multiple_Complex	6.51	7.91	-2.62	1.79E-06	3.17E-05
TC1000008054.hg.1	PLAU	Multiple_Complex	6.62	8.01	-2.62	1.09E-06	2.07E-05
TC1900011753.hg.1	ZNF284	Multiple_Complex	4.02	5.42	-2.63	1.22E-05	0.0002
TC1700008564.hg.1	TACO1	Multiple_Complex	7.82	9.22	-2.63	4.11E-06	6.39E-05
TC1000011606.hg.1	BLOC1S2	Multiple_Complex	8.14	9.54	-2.63	5.51E-06	8.21E-05
TC0100008664.hg.1	GADD45A	Multiple_Complex	9.11	10.51	-2.63	5.45E-07	1.16E-05
TC0X00009100.hg.1	TRAPPC2	Multiple_Complex	7.54	8.94	-2.64	1.02E-05	0.0001
TC1000007934.hg.1	ADAMTS14	Multiple_Complex	4.93	6.32	-2.64	3.15E-06	5.10E-05
TC1900011762.hg.1	APOC2	Multiple_Complex	6.71	8.11	-2.64	6.39E-07	1.33E-05
TC0500009055.hg.1	ABLIM3	Multiple_Complex	5.26	6.66	-2.64	6.13E-07	1.28E-05
TC0300011815.hg.1	DCBLD2	Multiple_Complex	10.32	11.72	-2.65	7.75E-07	1.56E-05
TC1200012647.hg.1	MYL6B	Multiple_Complex	7.6	9	-2.65	1.00E-05	0.0001
TC2000009973.hg.1	ZNF343	Multiple_Complex	5.23	6.63	-2.65	6.17E-05	0.0006
TC0X00009440.hg.1	CXorf38	Coding	7.38	8.78	-2.65	1.19E-06	2.24E-05
TC0400009978.hg.1	ACOX3	Multiple_Complex	5.32	6.73	-2.65	4.81E-06	7.28E-05
TC1700012340.hg.1	NUP88	Multiple_Complex	8.45	9.85	-2.65	1.53E-06	2.77E-05
TC0600012544.hg.1	RRAGD	Multiple_Complex	5.09	6.5	-2.66	3.61E-07	8.21E-06

TC0200012091.hg.1	ZNF512	NonCoding	3.69	5.1	-2.66	3.39E-06	5.42E-05
TC0200016484.hg.1	RGPD2	Multiple_Complex	3.99	5.4	-2.66	3.05E-06	5.00E-05
TC0200009982.hg.1	RAPGEF4	Multiple_Complex	5.14	6.55	-2.66	4.04E-06	6.31E-05
TC0100015162.hg.1	NBPF4	Multiple_Complex	6.48	7.89	-2.66	4.11E-07	9.11E-06
TC1600011414.hg.1	EDC4	Multiple_Complex	7.2	8.61	-2.66	7.12E-07	1.45E-05
TC0X00010678.hg.1	C1GALT1C1	Multiple_Complex	6.35	7.77	-2.67	1.27E-06	2.37E-05
TC1600011570.hg.1	CMC2	Multiple_Complex	8.68	10.1	-2.67	1.67E-06	2.99E-05
TC2000006559.hg.1	CDC25B	Multiple_Complex	9.08	10.5	-2.67	3.01E-06	4.93E-05
TC1200011894.hg.1	C12orf76	Multiple_Complex	5.07	6.49	-2.67	6.91E-06	9.86E-05
TC0X00007946.hg.1	ARMCX4	Multiple_Complex	3.42	4.83	-2.67	9.92E-06	0.0001
TC0700012444.hg.1	CADPS2	Multiple_Complex	6.07	7.49	-2.68	6.44E-06	9.34E-05
TC1400007712.hg.1	BATF	Multiple_Complex	7.17	8.59	-2.68	2.72E-06	4.53E-05
TC2000006781.hg.1	SNRPB2	Multiple_Complex	9.33	10.75	-2.68	1.96E-07	4.97E-06
TC0X00008724.hg.1	HMGB3	Multiple_Complex	8.42	9.85	-2.68	5.63E-06	8.34E-05
TC0100015945.hg.1	THBS3	Multiple_Complex	6.19	7.61	-2.68	3.12E-07	7.30E-06
TC0X00009869.hg.1	ARHGEF9	Multiple_Complex	6.54	7.97	-2.68	1.03E-06	1.98E-05
TC0600006524.hg.1	FOXQ1	Multiple_Complex	6.45	7.87	-2.69	2.42E-06	4.10E-05
TC0X00010799.hg.1	ELF4	Multiple_Complex	7.82	9.25	-2.7	1.52E-06	2.75E-05
TC0200010284.hg.1	MYO1B	Multiple_Complex	7.58	9.01	-2.7	1.37E-06	2.52E-05
TC0300009866.hg.1	CCDC50	Multiple_Complex	8.37	9.8	-2.7	2.86E-06	4.74E-05
TC0100007295.hg.1	EPHB2	Multiple_Complex	7.95	9.38	-2.7	1.83E-06	3.21E-05
TC0500009304.hg.1	GABRG2	Coding	3.24	4.67	-2.7	0.0002	0.0017
TC2100007534.hg.1	TEKT4P2	Multiple_Complex	5.35	6.78	-2.7	3.33E-05	0.0004
TC1800007905.hg.1	LPIN2	Multiple_Complex	7.06	8.5	-2.71	2.07E-05	0.0002
TC0900007472.hg.1	ANKRD20A1	Multiple_Complex	4.97	6.41	-2.71	1.33E-06	2.45E-05
TC0600014220.hg.1	SERPINB9	Multiple_Complex	5.56	7	-2.71	5.40E-06	8.06E-05
TC0600006926.hg.1	PAK1IP1	Multiple_Complex	8.6	10.04	-2.71	3.34E-06	5.36E-05
TC1000011012.hg.1	ECD	Multiple_Complex	9.02	10.46	-2.71	4.78E-07	1.04E-05
TC1500007174.hg.1	EID1	Multiple_Complex	9	10.45	-2.71	3.20E-05	0.0004
TC1300009229.hg.1	MZT1	Coding	8.89	10.33	-2.72	1.45E-06	2.65E-05
TC1100010718.hg.1	LRP4	Multiple_Complex	5.76	7.2	-2.72	2.62E-05	0.0003
TC0200016470.hg.1	SNRNP27	Multiple_Complex	7.14	8.59	-2.73	1.18E-06	2.23E-05
TC0X00009107.hg.1	GEMIN8	Multiple_Complex	6.6	8.05	-2.73	1.53E-06	2.76E-05
TC0300013083.hg.1	LRRC34	Multiple_Complex	6.21	7.66	-2.73	0.0002	0.0018
TC1800009289.hg.1	ACAA2	Multiple_Complex	7.73	9.18	-2.74	0.0002	0.0015
TC0500010640.hg.1	C5orf28	Multiple_Complex	8.2	9.65	-2.74	1.96E-06	3.42E-05
TC1200012867.hg.1	RIMBP2	Multiple_Complex	6.54	7.99	-2.74	5.57E-06	8.27E-05
TC0100008602.hg.1	CACHD1	Multiple_Complex	7.55	9.01	-2.74	8.94E-08	2.59E-06
TC0100015975.hg.1	RIT1	Multiple_Complex	7.2	8.66	-2.74	4.54E-06	6.93E-05
TC1700011448.hg.1	POLG2	Multiple_Complex	6.48	7.94	-2.75	1.48E-05	0.0002

TC0X00011211.hg.1	G6PD	Multiple_Complex	9.27	10.73	-2.75	2.97E-07	6.98E-06
TC0500011448.hg.1	ARRDC3	Multiple_Complex	7.59	9.05	-2.76	3.91E-07	8.73E-06
TC0100018238.hg.1	DNAJB4	Multiple_Complex	7.71	9.19	-2.77	2.17E-06	3.73E-05
TC0700007870.hg.1	STAG3L4	Multiple_Complex	7.68	9.15	-2.77	2.11E-07	5.27E-06
TC0600013119.hg.1	SOGA3	Multiple_Complex	3.66	5.13	-2.77	2.36E-05	0.0003
TC0600011386.hg.1	IER3	Coding	9.18	10.65	-2.78	1.05E-06	2.01E-05
TC0100008620.hg.1	DNAJC6	Multiple_Complex	6.06	7.54	-2.78	3.92E-07	8.75E-06
TC0400009179.hg.1	TMA16	Multiple_Complex	6.75	8.22	-2.78	5.65E-06	8.37E-05
TC1100008469.hg.1	SERPINH1	Multiple_Complex	9.11	10.59	-2.78	3.56E-07	8.11E-06
TC2000007016.hg.1	GINS1	Multiple_Complex	8.52	10	-2.78	1.00E-05	0.0001
TC0200015214.hg.1	OSGEPL1	Multiple_Complex	5.25	6.73	-2.79	0.0002	0.0018
TSUnmapped00000446.hg.1	INPP5D	Coding	6.25	7.74	-2.79	1.69E-06	3.01E-05
TC2000008946.hg.1	GGT7	Multiple_Complex	5.77	7.26	-2.8	1.02E-06	1.96E-05
TC1600010375.hg.1	CRNDE	Multiple_Complex	9.19	10.67	-2.8	1.49E-06	2.70E-05
TC0500012039.hg.1	ZCCHC10	Multiple_Complex	7.13	8.62	-2.8	2.71E-07	6.49E-06
TC1700006655.hg.1	ENO3	Multiple_Complex	6.43	7.92	-2.8	5.23E-06	7.83E-05
TC1100006494.hg.1	CD151	Multiple_Complex	8.81	10.3	-2.81	3.16E-07	7.36E-06
TC0200012405.hg.1	THADA	Multiple_Complex	5.96	7.45	-2.81	2.48E-06	4.17E-05
TC1900011763.hg.1	APOC4-APOC2	Multiple_Complex	5.75	7.24	-2.81	3.06E-06	5.01E-05
TC2000008111.hg.1	MYT1	Multiple_Complex	5.85	7.34	-2.81	3.23E-07	7.50E-06
TC2000009956.hg.1	PCMTD2	Multiple_Complex	7.63	9.12	-2.82	1.94E-06	3.38E-05
TC0600011376.hg.1	PPP1R18	Multiple_Complex	6.99	8.49	-2.82	6.92E-07	1.43E-05
TC0200007886.hg.1	PNO1	Multiple_Complex	7.59	9.08	-2.82	2.59E-07	6.22E-06
TC0700007345.hg.1	STK17A	Multiple_Complex	7.61	9.11	-2.82	8.29E-07	1.65E-05
TC0300006520.hg.1	LMCD1	Multiple_Complex	5.68	7.17	-2.82	7.07E-07	1.45E-05
TC1000007890.hg.1	HKDC1	Multiple_Complex	6.12	7.62	-2.82	2.02E-06	3.49E-05
TC1700008879.hg.1	ICT1	Multiple_Complex	7.34	8.85	-2.83	0.0001	0.001
TC1200007891.hg.1	DTX3	Multiple_Complex	5.88	7.39	-2.85	7.84E-07	1.57E-05
TC0100017094.hg.1	FAM72A	Multiple_Complex	8.08	9.59	-2.85	2.89E-07	6.83E-06
TC0X00009268.hg.1	PCYT1B	Multiple_Complex	4.39	5.9	-2.85	1.00E-06	1.93E-05
TC0200015397.hg.1	CLK1	Multiple_Complex	4.45	5.96	-2.85	0.0001	0.0011
TC1600006483.hg.1	MSLN	Multiple_Complex	5.2	6.71	-2.85	3.65E-07	8.28E-06
TC0900007113.hg.1	RECK	Multiple_Complex	4.52	6.03	-2.86	2.79E-07	6.65E-06
TC0100009723.hg.1	FAM72D	Coding	8.19	9.7	-2.86	6.47E-07	1.34E-05
TC2200006827.hg.1	GNAZ	Multiple_Complex	5.28	6.79	-2.86	1.64E-07	4.29E-06
TC0X00011404.hg.1	IDS	Multiple_Complex	7.66	9.18	-2.86	7.24E-07	1.47E-05
TC0X00010355.hg.1	TIMM8A	Multiple_Complex	7.58	9.1	-2.87	3.26E-06	5.26E-05
TC0800010163.hg.1	FGFR1	Multiple_Complex	5.53	7.05	-2.87	1.45E-07	3.88E-06
TC1400007584.hg.1	TTC9	Coding	5.51	7.03	-2.88	2.35E-06	3.99E-05
TC0900010311.hg.1	FAM27E3	Multiple_Complex	7.58	9.1	-2.88	4.10E-07	9.09E-06

TC0300006580.hg.1	IRAK2	Multiple_Complex	5.18	6.71	-2.88	8.81E-07	1.74E-05
TC0100018256.hg.1	NBPF6	Multiple_Complex	6.3	7.83	-2.88	3.85E-06	6.05E-05
TC0400010598.hg.1	GABRA2	Multiple_Complex	3.26	4.78	-2.88	5.82E-06	8.57E-05
TC0300007409.hg.1	SLC38A3	Multiple_Complex	5.84	7.36	-2.88	6.04E-07	1.27E-05
TC1600010262.hg.1	BRD7	Multiple_Complex	9.28	10.81	-2.89	6.69E-06	9.63E-05
TC2000008381.hg.1	JAG1	Multiple_Complex	8.19	9.72	-2.89	2.34E-07	5.72E-06
TC0200006665.hg.1	TAF1B	Multiple_Complex	4.94	6.48	-2.9	6.95E-07	1.43E-05
TC0X00007574.hg.1	ACRC	Multiple_Complex	5.56	7.1	-2.9	1.42E-05	0.0002
TC0800008321.hg.1	LAPTM4B	Multiple_Complex	7.41	8.95	-2.91	1.74E-07	4.51E-06
TC1700007704.hg.1	ARHGAP23	Multiple_Complex	5.84	7.38	-2.91	1.19E-07	3.29E-06
TC0900012158.hg.1	PALM2-AKAP2	Coding	5.3	6.84	-2.91	4.63E-07	1.01E-05
TC0X00011278.hg.1	PRRG1	Multiple_Complex	7.6	9.15	-2.92	9.17E-07	1.80E-05
TC0X00008826.hg.1	FLNA	NonCoding	6.54	8.08	-2.92	1.81E-05	0.0002
TC0100010874.hg.1	RGL1	Multiple_Complex	7.28	8.83	-2.92	1.07E-06	2.04E-05
TC1000012088.hg.1	C10orf88	Multiple_Complex	6.49	8.04	-2.92	1.03E-06	1.98E-05
TC1700008915.hg.1	SMIM6	Multiple_Complex	6.21	7.75	-2.92	1.64E-07	4.28E-06
TC0500009996.hg.1	NSUN2	Multiple_Complex	7.99	9.53	-2.93	1.41E-07	3.79E-06
TC0X00008481.hg.1	FAM122C	Multiple_Complex	6.77	8.33	-2.93	1.07E-06	2.03E-05
TC2000006755.hg.1	NDUFAF5	Multiple_Complex	7.68	9.23	-2.93	2.19E-06	3.75E-05
TC0200016643.hg.1	DNMT3A	Multiple_Complex	6.49	8.04	-2.93	2.13E-05	0.0003
TC0500011490.hg.1	TTC37	Multiple_Complex	7.18	8.73	-2.94	2.83E-07	6.72E-06
TC1100008144.hg.1	GSTP1	Multiple_Complex	9.29	10.85	-2.94	4.54E-07	9.94E-06
TC1600011424.hg.1	DDX19B	Multiple_Complex	8.7	10.26	-2.94	8.83E-07	1.74E-05
TC0500010511.hg.1	C5orf42	Multiple_Complex	4.67	6.23	-2.95	5.01E-06	7.55E-05
TC0500008540.hg.1	PHAX	Multiple_Complex	9	10.57	-2.95	5.32E-06	7.96E-05
TC2200009028.hg.1	CERK	Multiple_Complex	7.22	8.78	-2.95	1.23E-06	2.30E-05
TC1800006905.hg.1	CABYR	Multiple_Complex	4.37	5.94	-2.96	4.44E-06	6.80E-05
TC0200006687.hg.1	HPCAL1	Multiple_Complex	7.08	8.65	-2.97	7.28E-07	1.48E-05
TC1600011428.hg.1	TERF2IP	Coding	8.84	10.41	-2.97	5.82E-08	1.77E-06
TC0500006472.hg.1	TRIP13	Multiple_Complex	8.16	9.73	-2.98	8.34E-08	2.45E-06
TC1300007483.hg.1	BORA	Multiple_Complex	6.89	8.47	-2.98	8.81E-08	2.56E-06
TC0100011487.hg.1	TRAF5	Multiple_Complex	7.16	8.73	-2.98	6.54E-08	1.97E-06
TC2000006628.hg.1	MCM8	Multiple_Complex	9.09	10.66	-2.98	2.62E-05	0.0003
TC0600014237.hg.1	EEF1E1-BLOC1S5	Multiple_Complex	7.02	8.59	-2.98	1.10E-07	3.09E-06
TSUnmapped00000377.hg.1	INPP5D	NonCoding	6.97	8.55	-2.99	2.16E-07	5.36E-06
TC2000009198.hg.1	ADA	Multiple_Complex	4.57	6.15	-2.99	4.66E-07	1.01E-05
TC1400007120.hg.1	ATL1	Multiple_Complex	5.12	6.7	-2.99	4.10E-06	6.37E-05
TC1100009169.hg.1	TAGLN	Multiple_Complex	6.06	7.64	-2.99	1.99E-07	5.02E-06
TC1900010969.hg.1	IGFL4	Multiple_Complex	6.64	8.23	-2.99	4.80E-07	1.04E-05
TC0900008587.hg.1	TLR4	Multiple_Complex	5.14	6.73	-3.01	2.53E-07	6.11E-06

TC1400007201.hg.1	CDKN3	Multiple_Complex	9.19	10.78	-3.01	9.82E-08	2.80E-06
TC1500010914.hg.1	HDGFRP3	Multiple_Complex	6.71	8.3	-3.01	2.05E-07	5.14E-06
TC1000012595.hg.1	ZDHHC6	Multiple_Complex	7.06	8.65	-3.01	1.13E-07	3.15E-06
TC1200012612.hg.1	MED21	Multiple_Complex	8.45	10.04	-3.01	4.27E-08	1.36E-06
TC0200016445.hg.1	MSH6	Multiple_Complex	6.96	8.55	-3.01	5.17E-07	1.11E-05
TC0X00010434.hg.1	SLC25A53	Multiple_Complex	4.39	5.98	-3.01	8.83E-08	2.56E-06
TC1000009152.hg.1	HTRA1	Multiple_Complex	4.73	6.32	-3.01	3.34E-06	5.36E-05
TC0X00008136.hg.1	ALG13	Multiple_Complex	7.63	9.22	-3.02	1.57E-07	4.15E-06
TC2200008444.hg.1	SEC14L4	Multiple_Complex	5.26	6.86	-3.02	2.20E-06	3.76E-05
TC0700012760.hg.1	ZC3HAV1L	Multiple_Complex	7.44	9.04	-3.03	3.06E-07	7.17E-06
TC0700011948.hg.1	FAM200A	Coding	4.22	5.82	-3.03	2.34E-05	0.0003
TC0500010842.hg.1	PDE4D	Multiple_Complex	7.7	9.3	-3.03	1.60E-07	4.21E-06
TC0400012251.hg.1	C4orf46	Multiple_Complex	8.37	9.97	-3.04	8.72E-07	1.73E-05
TC0200013075.hg.1	TPRKB	Multiple_Complex	6.65	8.25	-3.04	1.39E-07	3.73E-06
TC1900011944.hg.1	FBXO17	Multiple_Complex	5.84	7.45	-3.04	2.17E-07	5.39E-06
TC1100006810.hg.1	IPO7	Multiple_Complex	9.39	11	-3.06	1.66E-07	4.32E-06
TC0100018349.hg.1	CNIH3	Multiple_Complex	5.18	6.79	-3.06	1.99E-06	3.45E-05
TC0500010920.hg.1	SREK1IP1	Multiple_Complex	8.72	10.34	-3.06	3.35E-07	7.69E-06
TC0500013222.hg.1	LOC100505841	Multiple_Complex	6.37	7.98	-3.07	0.0007	0.0048
TC0800007335.hg.1	LETM2	Multiple_Complex	6.07	7.69	-3.07	4.05E-07	9.00E-06
TC0100009534.hg.1	SLC22A15	Multiple_Complex	5.36	6.98	-3.07	0.0004	0.0033
TC0100014313.hg.1	PPAP2B	Unassigned	4.54	6.16	-3.08	6.50E-06	9.40E-05
TC1100010467.hg.1	WT1	Multiple_Complex	5.98	7.61	-3.08	4.33E-08	1.38E-06
TC0X00010196.hg.1	RPS6KA6	Multiple_Complex	4.28	5.91	-3.08	0.0001	0.001
TC1900008932.hg.1	RFPL4AL1	Coding	4.17	5.79	-3.08	2.22E-07	5.47E-06
TC1700007603.hg.1	RASL10B	Multiple_Complex	6.23	7.86	-3.08	4.35E-07	9.55E-06
TC0900012156.hg.1	PALM2	Multiple_Complex	4.81	6.44	-3.08	9.87E-07	1.91E-05
TC1100012020.hg.1	SESN3	Unassigned	2.86	4.48	-3.09	2.90E-06	4.80E-05
TC1000012117.hg.1	CHST15	Multiple_Complex	7.21	8.84	-3.1	3.96E-06	6.22E-05
TC1100009004.hg.1	FDX1	Coding	6.98	8.61	-3.1	1.29E-07	3.53E-06
TC2000008439.hg.1	ESF1	Multiple_Complex	8.06	9.69	-3.1	2.18E-07	5.40E-06
TC2000009017.hg.1	SOGA1	Multiple_Complex	6.6	8.23	-3.11	1.39E-06	2.55E-05
TC1100007819.hg.1	MYRF	Multiple_Complex	7.1	8.73	-3.11	7.02E-07	1.45E-05
TC0700008181.hg.1	GNAI1	Multiple_Complex	9.54	11.17	-3.11	3.74E-07	8.44E-06
TC1900011652.hg.1	ZNF358	Multiple_Complex	4.86	6.5	-3.11	2.50E-06	4.20E-05
TC0800008197.hg.1	OTUD6B	Multiple_Complex	7.43	9.06	-3.11	3.32E-05	0.0004
TC1300006594.hg.1	TNFRSF19	Multiple_Complex	5.7	7.34	-3.11	1.21E-07	3.34E-06
TC1500007814.hg.1	NEO1	Multiple_Complex	6.25	7.89	-3.11	1.67E-06	2.98E-05
TC0300009471.hg.1	SKIL	Multiple_Complex	7.28	8.92	-3.11	2.74E-07	6.54E-06
TC0X00010482.hg.1	PSMD10	Multiple_Complex	8.88	10.52	-3.12	3.75E-07	8.46E-06

TC0600006530.hg.1	FOXF2	Coding	6.12	7.77	-3.13	3.31E-07	7.62E-06
TC0100015509.hg.1	FAM72B	Multiple_Complex	8.17	9.82	-3.14	1.65E-07	4.31E-06
TC0X00011389.hg.1	NKAP	Multiple_Complex	6.07	7.72	-3.15	1.80E-06	3.18E-05
TC1600007113.hg.1	C16orf62	Multiple_Complex	4.37	6.02	-3.15	3.70E-06	5.84E-05
TC1100007050.hg.1	PRMT3	Multiple_Complex	7.67	9.32	-3.15	5.60E-07	1.19E-05
TC0X00008862.hg.1	FUNDC2	Multiple_Complex	7.79	9.44	-3.15	2.23E-07	5.50E-06
TC1200007153.hg.1	PPFIBP1	Multiple_Complex	8.36	10.02	-3.15	2.72E-07	6.49E-06
TC0X00009057.hg.1	MID1	Multiple_Complex	8.35	10.01	-3.15	6.07E-08	1.84E-06
TC1000011563.hg.1	SFRP5	Coding	6.03	7.69	-3.16	2.63E-08	8.97E-07
TC1900006985.hg.1	PDE4A	Multiple_Complex	5	6.66	-3.17	7.44E-07	1.50E-05
TC0600010921.hg.1	ATXN1	Multiple_Complex	6.61	8.28	-3.17	2.42E-06	4.09E-05
TC1900010651.hg.1	GMFG	Multiple_Complex	5.07	6.73	-3.17	7.34E-08	2.18E-06
TC1900007826.hg.1	ZNF30	Multiple_Complex	4.48	6.14	-3.17	4.35E-05	0.0005
TC1700012458.hg.1	CEP112	Multiple_Complex	3.34	5.01	-3.17	1.04E-06	1.98E-05
TC1100008199.hg.1	GAL	Multiple_Complex	8.12	9.78	-3.17	1.23E-07	3.39E-06
TC0500008785.hg.1	EGR1	Coding	8.32	9.99	-3.18	4.55E-06	6.94E-05
TC0200010572.hg.1	ZDBF2	Multiple_Complex	3.72	5.39	-3.18	1.48E-06	2.68E-05
TC0200011121.hg.1	INPP5D	Multiple_Complex	6.66	8.34	-3.19	1.72E-06	3.05E-05
TC0500013153.hg.1	MTRR	Multiple_Complex	8.27	9.94	-3.2	8.14E-08	2.40E-06
TC0200015793.hg.1	OBSL1	Multiple_Complex	5.37	7.05	-3.2	3.39E-07	7.78E-06
TC0600006918.hg.1	GCNT2	Multiple_Complex	3.52	5.2	-3.2	1.81E-07	4.66E-06
TC2000009604.hg.1	PMEPA1	Multiple_Complex	7.14	8.81	-3.2	5.46E-08	1.68E-06
TC0700013441.hg.1	IFRD1	Multiple_Complex	9.42	11.09	-3.2	1.01E-07	2.86E-06
TC0100015171.hg.1	HENMT1	Multiple_Complex	3.5	5.18	-3.22	1.28E-06	2.37E-05
TC0600014238.hg.1	BLOC1S5	Multiple_Complex	7.09	8.78	-3.22	6.36E-08	1.92E-06
TC1800006650.hg.1	RAB31	Multiple_Complex	4.07	5.76	-3.23	9.23E-06	0.0001
TC1200006787.hg.1	GABARAPL1	Multiple_Complex	6.31	8.01	-3.25	2.91E-07	6.88E-06
TC1700007204.hg.1	SPECC1	Multiple_Complex	5.7	7.4	-3.26	1.35E-06	2.50E-05
TC0600008071.hg.1	PTK7	Multiple_Complex	6.58	8.3	-3.3	1.14E-07	3.19E-06
TC0500011529.hg.1	RIOK2	Multiple_Complex	6.77	8.49	-3.31	1.49E-06	2.69E-05
TC1200010740.hg.1	KRT80	Multiple_Complex	7.17	8.9	-3.32	3.70E-08	1.20E-06
TC0700010463.hg.1	IGF2BP3	Multiple_Complex	7.57	9.3	-3.32	2.65E-08	9.00E-07
TC0300013520.hg.1	CLDN1	Multiple_Complex	9.4	11.13	-3.32	3.69E-07	8.34E-06
TC0400011159.hg.1	PRKG2	Multiple_Complex	5.42	7.15	-3.33	4.56E-05	0.0005
TC0X00006875.hg.1	MAGEB2	Coding	5.04	6.77	-3.33	1.97E-05	0.0002
TC1200007892.hg.1	ARHGEF25	Multiple_Complex	3.67	5.41	-3.33	1.64E-07	4.28E-06
TC0700013119.hg.1	XRCC2	Multiple_Complex	8	9.73	-3.33	9.56E-08	2.74E-06
TC0400006433.hg.1	ZNF595	Multiple_Complex	5.77	7.51	-3.34	1.44E-08	5.40E-07
TC0300009625.hg.1	TTC14	Multiple_Complex	5.68	7.42	-3.35	7.87E-06	0.0001
TC1000007132.hg.1	BAMBI	Multiple_Complex	8.28	10.03	-3.35	3.67E-08	1.20E-06

TC0200014085.hg.1	TFCP2L1	Multiple_Complex	5	6.74	-3.35	9.60E-07	1.86E-05
TC0700008000.hg.1	LAT2	Multiple_Complex	6.09	7.84	-3.36	5.10E-07	1.10E-05
TC1200010531.hg.1	SLC38A4	Coding	3.36	5.11	-3.36	5.63E-06	8.34E-05
TC1200011506.hg.1	BTG1	Multiple_Complex	5.48	7.24	-3.37	5.21E-08	1.62E-06
TC1200007147.hg.1	ARNTL2	Multiple_Complex	8.06	9.81	-3.37	1.15E-07	3.21E-06
TC0300010315.hg.1	IQSEC1	Multiple_Complex	5.52	7.27	-3.37	1.15E-07	3.21E-06
TC0600010066.hg.1	IGF2R	Multiple_Complex	6.69	8.45	-3.38	1.26E-08	4.81E-07
TC1700008598.hg.1	MILR1	Multiple_Complex	4.13	5.89	-3.39	8.74E-08	2.54E-06
TC0100010877.hg.1	TSEN15	Multiple_Complex	6.79	8.55	-3.39	3.79E-07	8.52E-06
TC0600011173.hg.1	GUSBP2	Multiple_Complex	9.27	11.03	-3.4	8.93E-08	2.58E-06
TC1300008938.hg.1	DLEU2	Multiple_Complex	6.78	8.55	-3.41	4.91E-07	1.06E-05
TC2000007792.hg.1	PFDN4	Multiple_Complex	8.37	10.14	-3.41	1.64E-07	4.28E-06
TC0700011962.hg.1	AZGP1	Multiple_Complex	6.58	8.36	-3.43	2.51E-06	4.21E-05
TC0800010506.hg.1	PLAG1	Multiple_Complex	5.37	7.15	-3.44	9.11E-07	1.79E-05
TC1400008963.hg.1	MBIP	Multiple_Complex	7.27	9.05	-3.45	9.13E-07	1.79E-05
TC1000007404.hg.1	CSGALNACT2	Multiple_Complex	6.56	8.35	-3.45	2.74E-07	6.53E-06
TC1000010642.hg.1	A1CF	Multiple_Complex	3.82	5.61	-3.46	1.64E-06	2.94E-05
TC0200009871.hg.1	CSRNP3	Multiple_Complex	5.22	7.01	-3.47	2.16E-07	5.36E-06
TC0100014937.hg.1	FAM69A	Coding	5.32	7.12	-3.49	7.71E-06	0.0001
TC0700013182.hg.1	CNPY1	Multiple_Complex	4.84	6.64	-3.49	3.70E-08	1.20E-06
TC0200016252.hg.1	HDAC4	Multiple_Complex	7.8	9.61	-3.5	1.45E-08	5.41E-07
TC1100008804.hg.1	ANKRD49	Multiple_Complex	6.12	7.93	-3.51	5.35E-08	1.66E-06
TC1700012295.hg.1	ARSG	Multiple_Complex	4.5	6.32	-3.52	1.16E-07	3.21E-06
TC1400009351.hg.1	SIX1	Multiple_Complex	6.72	8.54	-3.52	7.44E-09	3.04E-07
TC0200014382.hg.1	TMEM163	Multiple_Complex	5.8	7.61	-3.53	2.37E-07	5.78E-06
TC1400007443.hg.1	HSPA2	Multiple_Complex	6.9	8.72	-3.53	2.49E-08	8.57E-07
TC2000006501.hg.1	SIRPA	Coding	5.73	7.55	-3.53	1.28E-07	3.50E-06
TC0100014774.hg.1	C1orf52	Multiple_Complex	7.58	9.42	-3.58	7.89E-08	2.33E-06
TC1300009740.hg.1	LIG4	Coding	5.66	7.51	-3.6	2.76E-07	6.58E-06
TC2000007633.hg.1	DDX27	Multiple_Complex	7.83	9.68	-3.61	8.45E-08	2.47E-06
TC2000006894.hg.1	KIZ	Multiple_Complex	5.33	7.19	-3.61	1.27E-07	3.48E-06
TC1600010599.hg.1	NAE1	Multiple_Complex	8.52	10.38	-3.61	1.37E-08	5.15E-07
TC1500008360.hg.1	SLCO3A1	Multiple_Complex	6.05	7.9	-3.61	1.86E-06	3.27E-05
TC1200010229.hg.1	PTHLH	Multiple_Complex	6.49	8.35	-3.62	1.22E-07	3.36E-06
TC1100012775.hg.1	ETS1	Multiple_Complex	6.77	8.63	-3.62	5.35E-08	1.66E-06
TC0800010685.hg.1	MYBL1	Multiple_Complex	3.98	5.85	-3.64	7.62E-07	1.54E-05
TC1600011375.hg.1	QPRT	Multiple_Complex	6.23	8.1	-3.64	1.29E-08	4.89E-07
TC0X00006715.hg.1	SCML1	Multiple_Complex	7.4	9.27	-3.64	9.84E-08	2.81E-06
TC0X00009537.hg.1	ZNF674	Multiple_Complex	6.87	8.74	-3.65	1.60E-07	4.21E-06
TC1200007804.hg.1	METTL7B	Multiple_Complex	7.45	9.32	-3.65	3.18E-08	1.06E-06

TC0700013342.hg.1	CHN2	Multiple_Complex	5.92	7.8	-3.67	1.45E-07	3.88E-06
TC0200010164.hg.1	SSFA2	Multiple_Complex	7.06	8.94	-3.68	3.42E-07	7.84E-06
TC1000007701.hg.1	TFAM	Multiple_Complex	9.59	11.47	-3.69	8.42E-08	2.47E-06
TC0X00010406.hg.1	TCEAL8	Multiple_Complex	5.07	6.96	-3.7	1.60E-08	5.83E-07
TC0400009892.hg.1	CRMP1	Multiple_Complex	6.1	7.99	-3.7	7.17E-09	2.97E-07
TC0600014328.hg.1	OSTM1	Multiple_Complex	7.11	9	-3.71	4.18E-08	1.34E-06
TC1100007184.hg.1	DNAJC24	Multiple_Complex	6.95	8.84	-3.72	4.46E-08	1.41E-06
TC1200007137.hg.1	FGFR1OP2	Multiple_Complex	8.37	10.27	-3.72	3.05E-08	1.02E-06
TC0800008769.hg.1	TRMT12	Multiple_Complex	4.38	6.28	-3.72	2.78E-08	9.40E-07
TC1200012637.hg.1	HOXC10	Multiple_Complex	6.75	8.65	-3.73	9.91E-09	3.90E-07
TC1800008580.hg.1	PSTPIP2	Multiple_Complex	3.7	5.6	-3.74	5.18E-08	1.61E-06
TC0100015867.hg.1	S100A3	Multiple_Complex	7.04	8.94	-3.74	1.65E-08	5.97E-07
TC0800010630.hg.1	GGH	Multiple_Complex	7.61	9.52	-3.74	3.48E-08	1.14E-06
TC0600010609.hg.1	PXDC1	Multiple_Complex	4.3	6.2	-3.75	5.80E-08	1.77E-06
TC1600010175.hg.1	C16orf87	Multiple_Complex	6.64	8.55	-3.76	3.25E-08	1.07E-06
TC1400008919.hg.1	CFL2	Multiple_Complex	6.5	8.42	-3.77	5.65E-08	1.73E-06
TC1600008585.hg.1	CMIP	Multiple_Complex	8.68	10.6	-3.77	3.77E-07	8.49E-06
TC0900012155.hg.1	MSANTD3-TMEFF1	Coding	4.86	6.78	-3.78	7.08E-08	2.12E-06
TC1700012460.hg.1	ABCA5	Multiple_Complex	4.78	6.7	-3.79	6.88E-06	9.83E-05
TC1800008121.hg.1	SPIRE1	Multiple_Complex	4.82	6.75	-3.8	1.81E-07	4.66E-06
TC0700012230.hg.1	NRCAM	Multiple_Complex	5.68	7.6	-3.8	9.07E-08	2.62E-06
TC1000012536.hg.1	MPP7	Multiple_Complex	7.05	8.98	-3.8	1.31E-07	3.57E-06
TC1100009929.hg.1	OR51I1	Coding	3.73	5.66	-3.81	1.85E-07	4.72E-06
TC0700009886.hg.1	DNAJB6	Multiple_Complex	8.8	10.74	-3.83	3.19E-09	1.50E-07
TC1700009619.hg.1	DLG4	Multiple_Complex	5.8	7.73	-3.83	3.53E-07	8.05E-06
TC1500006925.hg.1	THBS1	Multiple_Complex	6.48	8.41	-3.83	5.54E-08	1.70E-06
TC1200009397.hg.1	FZD10	Coding	6.15	8.08	-3.83	2.22E-08	7.79E-07
TC0600014239.hg.1	EEF1E1	Coding	8.33	10.27	-3.83	1.46E-08	5.44E-07
TC1100013134.hg.1	HBE1	Multiple_Complex	5.38	7.32	-3.84	5.41E-08	1.67E-06
TC0300010720.hg.1	EPM2AIP1	Multiple_Complex	4.82	6.76	-3.85	1.08E-06	2.05E-05
TC0X00008080.hg.1	VSIG1	Multiple_Complex	4.81	6.76	-3.85	1.22E-07	3.36E-06
TC1000008221.hg.1	TMEM254	Multiple_Complex	3.72	5.67	-3.85	2.11E-07	5.27E-06
TC1500010006.hg.1	STRA6	Multiple_Complex	6.4	8.36	-3.87	1.09E-08	4.22E-07
TC1000012227.hg.1	CLRN3	Coding	6.63	8.59	-3.88	1.38E-07	3.72E-06
TC2100008285.hg.1	CBS	Multiple_Complex	6.79	8.75	-3.88	2.40E-07	5.85E-06
TC1500009641.hg.1	RORA	Multiple_Complex	4.71	6.66	-3.89	2.06E-05	0.0002
TC0700013524.hg.1	MACC1	Multiple_Complex	8.83	10.79	-3.89	2.10E-08	7.42E-07
TC0100015182.hg.1	TAF13	Multiple_Complex	8.5	10.46	-3.9	7.72E-07	1.56E-05
TC0600007616.hg.1	HSPA1B	Coding	9.6	11.56	-3.91	3.91E-09	1.78E-07
TC0200015093.hg.1	ZNF385B	Multiple_Complex	4.02	6	-3.93	1.69E-08	6.08E-07

TC0700011003.hg.1	HUS1	Multiple_Complex	8.41	10.39	-3.93	1.36E-08	5.15E-07
TC1100007220.hg.1	DEPDC7	Multiple_Complex	7.51	9.49	-3.95	7.33E-09	3.01E-07
TC1100013082.hg.1	PIWIL4	Multiple_Complex	3.86	5.85	-3.96	9.25E-07	1.81E-05
TC1700009150.hg.1	BAIAP2	Multiple_Complex	6.39	8.38	-3.96	2.27E-07	5.58E-06
TC1200007731.hg.1	HOXC13	Multiple_Complex	6.2	8.19	-3.96	8.74E-09	3.52E-07
TC1600011368.hg.1	LAT	Multiple_Complex	6.6	8.59	-3.97	2.51E-08	8.62E-07
TC0700011665.hg.1	SEMA3A	Unassigned	3.83	5.82	-3.98	0.0005	0.0035
TC0600012113.hg.1	BMP5	Multiple_Complex	5.03	7.03	-3.98	3.74E-08	1.21E-06
TC0500009881.hg.1	MRPL36	Multiple_Complex	7.91	9.9	-3.99	2.57E-08	8.79E-07
TC1500010692.hg.1	MKRN3	Coding	4.79	6.79	-3.99	2.12E-05	0.0003
TC0X00008492.hg.1	SMIM10	Multiple_Complex	5.02	7.01	-4	4.15E-09	1.87E-07
TC0900012154.hg.1	TMEFF1	Multiple_Complex	5.28	7.28	-4	1.10E-07	3.09E-06
TC1200011812.hg.1	CRY1	Multiple_Complex	5.04	7.04	-4.02	6.25E-07	1.31E-05
TC0700013050.hg.1	KCNH2	Multiple_Complex	6.44	8.45	-4.02	2.45E-09	1.22E-07
TC0300011391.hg.1	ADAMTS9	Multiple_Complex	3.14	5.15	-4.02	4.79E-06	7.26E-05
TC0X00009890.hg.1	ZC4H2	Multiple_Complex	5.18	7.19	-4.03	2.37E-07	5.78E-06
TC0400012641.hg.1	PDLIM3	Multiple_Complex	5.91	7.92	-4.03	4.09E-07	9.09E-06
TC0700010719.hg.1	TBX20	Multiple_Complex	4.51	6.52	-4.04	2.83E-08	9.50E-07
TC0500011725.hg.1	MCC	Multiple_Complex	5.32	7.33	-4.04	2.42E-05	0.0003
TC1200006520.hg.1	TSPAN9	Multiple_Complex	6.3	8.33	-4.06	2.27E-06	3.87E-05
TC0700010989.hg.1	TNS3	Multiple_Complex	8.35	10.37	-4.07	1.88E-09	9.72E-08
TC0400012754.hg.1	ZNF141	Multiple_Complex	5.17	7.2	-4.07	7.38E-07	1.50E-05
TC0300010760.hg.1	SCN5A	Multiple_Complex	6.09	8.12	-4.07	5.46E-08	1.68E-06
TC0100018246.hg.1	LRRC8C	Multiple_Complex	5.37	7.4	-4.08	7.24E-09	2.99E-07
TC0300013513.hg.1	P3H2	Multiple_Complex	4.12	6.16	-4.09	5.89E-09	2.51E-07
TC0700010005.hg.1	ZFAND2A	Multiple_Complex	7.49	9.53	-4.09	2.93E-09	1.41E-07
TC0500010019.hg.1	FASTKD3	Multiple_Complex	5.33	7.36	-4.1	1.13E-07	3.17E-06
TC1700010847.hg.1	GJC1	Multiple_Complex	4.29	6.33	-4.13	1.05E-07	2.97E-06
TC0300011084.hg.1	CAMKV	Multiple_Complex	4.8	6.84	-4.13	2.02E-08	7.16E-07
TC1200007105.hg.1	LYRM5	Multiple_Complex	7.27	9.31	-4.13	6.96E-09	2.90E-07
TC0500009521.hg.1	CPEB4	Multiple_Complex	6.01	8.06	-4.15	9.56E-09	3.82E-07
TC1000007861.hg.1	TET1	Multiple_Complex	5.28	7.34	-4.17	4.03E-09	1.82E-07
TC0800008032.hg.1	ZC2HC1A	Multiple_Complex	5.61	7.67	-4.18	1.13E-07	3.16E-06
TC0700012754.hg.1	ATP6V0A4	Multiple_Complex	5	7.07	-4.19	8.06E-09	3.27E-07
TC2100007204.hg.1	FAM3B	Multiple_Complex	3.52	5.59	-4.2	7.65E-09	3.11E-07
TC0400009879.hg.1	CYTL1	Coding	5.56	7.63	-4.22	4.01E-09	1.82E-07
TC1500007365.hg.1	LIPC	Multiple_Complex	5.47	7.55	-4.23	2.97E-07	6.98E-06
TC1500007346.hg.1	CGNL1	Multiple_Complex	4.68	6.77	-4.26	1.82E-07	4.68E-06
TC0500008835.hg.1	CXXC5	Multiple_Complex	6.62	8.71	-4.26	2.41E-09	1.20E-07
TC0300014064.hg.1	PFN2	Multiple_Complex	6.59	8.68	-4.27	9.30E-08	2.68E-06

TC1900008931.hg.1	RFPL4A	Coding	3.95	6.06	-4.29	3.83E-07	8.59E-06
TC1700011815.hg.1	MXRA7	Multiple_Complex	7.43	9.53	-4.3	1.34E-09	7.24E-08
TC0600007143.hg.1	CDKAL1	Multiple_Complex	7.4	9.51	-4.3	3.35E-08	1.10E-06
TC1400010045.hg.1	SERPINA1	Multiple_Complex	5.95	8.06	-4.3	5.45E-09	2.34E-07
TC0300007406.hg.1	SEMA3F	Multiple_Complex	4.93	7.04	-4.33	9.85E-09	3.88E-07
TC0500009987.hg.1	MED10	Multiple_Complex	8.57	10.68	-4.34	7.38E-09	3.02E-07
TC0900009949.hg.1	TPM2	Multiple_Complex	7.3	9.43	-4.36	1.67E-08	6.02E-07
TC1700012184.hg.1	CHRNB1	Multiple_Complex	5.4	7.53	-4.37	3.20E-08	1.06E-06
TC1000011933.hg.1	HSPA12A	Multiple_Complex	4.76	6.9	-4.41	6.80E-07	1.41E-05
TC0400007520.hg.1	RASL11B	Multiple_Complex	4.39	6.53	-4.41	2.50E-09	1.23E-07
TC0200016224.hg.1	PER2	Multiple_Complex	3.93	6.07	-4.41	3.99E-08	1.29E-06
TC1200009796.hg.1	SLC2A14	Multiple_Complex	6.92	9.07	-4.43	3.31E-09	1.55E-07
TC0700006945.hg.1	MPP6	Multiple_Complex	6.7	8.85	-4.43	4.52E-08	1.42E-06
TC0900010284.hg.1	ANKRD20A1	Multiple_Complex	5.24	7.39	-4.45	3.00E-08	1.00E-06
TC0700007034.hg.1	CREB5	Multiple_Complex	6.79	8.95	-4.46	9.35E-08	2.69E-06
TC0300014094.hg.1	TCTEX1D2	Multiple_Complex	5.66	7.81	-4.46	1.67E-06	2.98E-05
TC1100009926.hg.1	OR51B2	Multiple_Complex	3.93	6.08	-4.46	7.55E-08	2.24E-06
TC0500009856.hg.1	CLPTM1L	Multiple_Complex	7.1	9.26	-4.46	8.71E-09	3.51E-07
TC0200014735.hg.1	RBMS1	Multiple_Complex	6.31	8.48	-4.48	8.84E-08	2.56E-06
TC0200015876.hg.1	SERPINE2	Multiple_Complex	6.34	8.51	-4.49	9.67E-10	5.39E-08
TC0100015866.hg.1	S100A4	Multiple_Complex	7.88	10.05	-4.49	1.12E-08	4.34E-07
TC0X00011194.hg.1	FLNA	Multiple_Complex	8.23	10.41	-4.52	7.11E-08	2.12E-06
TC0X00010880.hg.1	MOSPD1	Multiple_Complex	8.42	10.61	-4.54	1.55E-08	5.68E-07
TC0300012397.hg.1	PLXND1	Multiple_Complex	5.76	7.96	-4.58	1.91E-08	6.79E-07
TC0X00010366.hg.1	ARMCX6	Multiple_Complex	5.59	7.79	-4.59	4.34E-09	1.93E-07
TC1700008511.hg.1	TBX2	Multiple_Complex	5.95	8.16	-4.62	1.81E-09	9.42E-08
TC1100007030.hg.1	NAV2	Multiple_Complex	6.59	8.8	-4.63	3.58E-09	1.66E-07
TC1100008999.hg.1	ZC3H12C	Multiple_Complex	3.96	6.17	-4.63	1.34E-07	3.62E-06
TC0X00011237.hg.1	RAB39B	Coding	4.93	7.14	-4.64	2.48E-06	4.17E-05
TC1100010123.hg.1	DKK3	Multiple_Complex	4.31	6.53	-4.64	7.76E-06	0.0001
TC0700006737.hg.1	ARL4A	Coding	6.59	8.82	-4.68	1.60E-07	4.22E-06
TC2000009357.hg.1	PREX1	Multiple_Complex	4.43	6.66	-4.7	5.55E-10	3.38E-08
TC1000010827.hg.1	DNAJC12	Multiple_Complex	6.43	8.66	-4.71	7.14E-10	4.15E-08
TC0100010360.hg.1	HSPA6	Multiple_Complex	6.61	8.84	-4.71	2.21E-09	1.11E-07
TC0X00010785.hg.1	SMARCA1	Multiple_Complex	6.3	8.54	-4.74	2.28E-08	7.98E-07
TC1900008134.hg.1	CYP2B6	Multiple_Complex	5.01	7.25	-4.74	4.59E-09	2.01E-07
TC0100008496.hg.1	FGGY	Multiple_Complex	6.32	8.57	-4.75	5.26E-09	2.27E-07
TC0100013999.hg.1	ZSWIM5	Multiple_Complex	5.21	7.46	-4.77	8.16E-09	3.29E-07
TC0700009394.hg.1	AGK	Multiple_Complex	7.35	9.61	-4.79	4.30E-09	1.92E-07
TC1900011774.hg.1	EMP3	Multiple_Complex	7.21	9.48	-4.8	1.64E-08	5.96E-07

TC0300012155.hg.1	HGD	Multiple_Complex	5.6	7.87	-4.82	1.20E-09	6.57E-08
TC0200015000.hg.1	CHRNA1	Multiple_Complex	4.89	7.16	-4.83	1.11E-06	2.11E-05
TC0X00011413.hg.1	L1CAM	Multiple_Complex	6.02	8.3	-4.83	3.96E-07	8.82E-06
TC1100008113.hg.1	RHOD	Multiple_Complex	6.78	9.06	-4.86	5.11E-10	3.15E-08
TC1100009374.hg.1	VWA5A	Multiple_Complex	4.31	6.6	-4.88	7.06E-07	1.45E-05
TC2100006909.hg.1	HUNK	Multiple_Complex	5.35	7.64	-4.89	1.80E-09	9.39E-08
TC0800010918.hg.1	ZNF704	Multiple_Complex	5.22	7.52	-4.9	3.89E-09	1.78E-07
TC1200010538.hg.1	AMIGO2	Coding	5.85	8.15	-4.93	3.45E-09	1.61E-07
TC0400008041.hg.1	PTPN13	Multiple_Complex	3.53	5.84	-4.95	2.44E-06	4.11E-05
TC1100010058.hg.1	DENND5A	Multiple_Complex	6.28	8.6	-5	2.81E-09	1.36E-07
TC1400009967.hg.1	TTC7B	Multiple_Complex	5.7	8.02	-5.01	6.42E-10	3.82E-08
TC0300012598.hg.1	RBP1	Multiple_Complex	6.17	8.5	-5.02	4.91E-10	3.03E-08
TC1100009924.hg.1	OR51B4	Coding	4.41	6.73	-5.02	3.84E-07	8.60E-06
TC1500007640.hg.1	IQCH	Multiple_Complex	6.41	8.74	-5.02	8.83E-09	3.55E-07
TC1300009674.hg.1	KDELC1	Multiple_Complex	6.46	8.79	-5.02	5.95E-09	2.53E-07
TC0900011246.hg.1	ZNF883	Multiple_Complex	3.81	6.14	-5.03	9.81E-09	3.88E-07
TC0X00007107.hg.1	KRBOX4	Multiple_Complex	5.35	7.69	-5.05	7.77E-10	4.48E-08
TC2000006442.hg.1	NRSN2	Multiple_Complex	5.47	7.82	-5.09	4.03E-10	2.57E-08
TC0100018443.hg.1	PLPP3	Multiple_Complex	7.49	9.84	-5.09	2.54E-09	1.24E-07
TC1100008340.hg.1	FOLR1	Multiple_Complex	4.22	6.58	-5.12	5.28E-09	2.28E-07
TC0800008062.hg.1	ZBTB10	Coding	4.92	7.28	-5.12	8.25E-06	0.0001
TC0X00009942.hg.1	PJA1	Multiple_Complex	5.34	7.7	-5.14	1.54E-08	5.68E-07
TC1500008038.hg.1	ARNT2	Multiple_Complex	5.87	8.24	-5.17	9.07E-10	5.08E-08
TC0X00009139.hg.1	AP1S2	Multiple_Complex	8.44	10.81	-5.17	1.39E-09	7.44E-08
TC0500012164.hg.1	GFRA3	Multiple_Complex	4.48	6.86	-5.19	5.72E-10	3.45E-08
TC1700009215.hg.1	ASPSCR1	Multiple_Complex	6.95	9.33	-5.2	5.33E-10	3.28E-08
TC0500013301.hg.1	DAB2	Multiple_Complex	4.78	7.16	-5.22	8.63E-08	2.52E-06
TC1900009824.hg.1	DNAJB1	Multiple_Complex	9.87	12.27	-5.26	5.42E-10	3.32E-08
TC1500009457.hg.1	MYO5A	Multiple_Complex	6.1	8.49	-5.26	4.38E-09	1.95E-07
TC1400010624.hg.1	PTGR2	Multiple_Complex	6	8.4	-5.28	6.73E-09	2.83E-07
TC0500012975.hg.1	DBN1	Multiple_Complex	6.61	9.02	-5.32	6.82E-10	3.99E-08
TC1100007307.hg.1	C11orf74	Multiple_Complex	6.1	8.51	-5.33	3.08E-09	1.46E-07
TC0300011628.hg.1	ROBO1	Multiple_Complex	7.89	10.31	-5.37	1.91E-10	1.41E-08
TC0Y00006898.hg.1	DHRSX	NonCoding	4.67	7.1	-5.41	1.57E-07	4.15E-06
TC0700009472.hg.1	EPHB6	Multiple_Complex	5.75	8.19	-5.42	1.13E-08	4.38E-07
TC0700010286.hg.1	VWDE	Multiple_Complex	4.09	6.55	-5.5	8.53E-10	4.84E-08
TC1800007471.hg.1	PMAIP1	Multiple_Complex	6.81	9.27	-5.51	6.83E-09	2.86E-07
TC0300006691.hg.1	FGD5	Multiple_Complex	4.75	7.22	-5.53	4.51E-09	1.99E-07
TC2200008134.hg.1	ZNF280B	Multiple_Complex	4.57	7.05	-5.56	7.22E-09	2.99E-07
TC1100009285.hg.1	TMEM136	Multiple_Complex	5.09	7.57	-5.56	2.56E-07	6.17E-06

TC0200009933.hg.1	ERICH2	Multiple_Complex	3.65	6.13	-5.58	5.67E-10	3.42E-08
TC1000012428.hg.1	AKR1C3	Multiple_Complex	7.6	10.12	-5.73	8.41E-10	4.78E-08
TC1500010888.hg.1	PARP6	Multiple_Complex	5.73	8.26	-5.78	2.52E-09	1.24E-07
TC1100012389.hg.1	CADM1	Multiple_Complex	3.88	6.41	-5.8	8.58E-10	4.86E-08
TC0X00007951.hg.1	ARMCX3	Multiple_Complex	5.18	7.72	-5.81	8.24E-10	4.74E-08
TC1000010716.hg.1	FAM13C	Multiple_Complex	5.52	8.06	-5.82	1.43E-08	5.38E-07
TC1100012535.hg.1	PVRL1	Multiple_Complex	7.56	10.11	-5.85	1.21E-10	9.58E-09
TC0300009258.hg.1	MME	Multiple_Complex	4.16	6.72	-5.88	2.04E-09	1.04E-07
TC1300008533.hg.1	HSPH1	Multiple_Complex	9.16	11.71	-5.88	3.29E-10	2.18E-08
TC2000008027.hg.1	NTSR1	Multiple_Complex	6.3	8.88	-5.96	3.28E-08	1.09E-06
TC0200015002.hg.1	CHN1	Multiple_Complex	7.11	9.7	-6.02	1.73E-10	1.30E-08
TC0X00011238.hg.1	CLIC2	Multiple_Complex	4.58	7.19	-6.1	2.01E-10	1.47E-08
TC1200007146.hg.1	STK38L	Multiple_Complex	8.35	10.96	-6.13	4.45E-10	2.81E-08
TC1400010620.hg.1	SNAPC1	Multiple_Complex	6.47	9.11	-6.21	2.82E-08	9.48E-07
TC0X00008001.hg.1	WBP5	Coding	5.15	7.79	-6.21	7.34E-08	2.18E-06
TC1100011797.hg.1	RAB30	Multiple_Complex	6.16	8.81	-6.28	2.86E-10	1.94E-08
TC0900011192.hg.1	LPAR1	Multiple_Complex	5.13	7.78	-6.28	3.26E-09	1.53E-07
TC1000012516.hg.1	AKR1C2	Multiple_Complex	4.8	7.46	-6.3	6.12E-11	5.51E-09
TC1100011446.hg.1	FGF19	Coding	6.97	9.63	-6.31	9.92E-11	8.15E-09
TC0X00011121.hg.1	GABRE	Multiple_Complex	5.3	7.96	-6.31	9.54E-08	2.74E-06
TC0200010966.hg.1	RHBDD1	Multiple_Complex	5.97	8.63	-6.36	2.24E-09	1.12E-07
TC0600009862.hg.1	AKAP12	Multiple_Complex	7.71	10.4	-6.41	3.40E-10	2.22E-08
TC0500010639.hg.1	CCL28	Multiple_Complex	5.66	8.35	-6.45	1.47E-10	1.14E-08
TC1200012876.hg.1	CHFR	Multiple_Complex	5.04	7.74	-6.47	2.00E-07	5.05E-06
TC1200010839.hg.1	ITGA5	Multiple_Complex	4.6	7.3	-6.49	4.97E-09	2.16E-07
TC0200010165.hg.1	PPP1R1C	Multiple_Complex	5.04	7.74	-6.5	7.68E-10	4.44E-08
TC0600013010.hg.1	MAN1A1	Multiple_Complex	7.04	9.74	-6.5	6.59E-10	3.91E-08
TC0400009223.hg.1	CPE	Coding	4.26	6.96	-6.5	3.65E-10	2.36E-08
TC2000009813.hg.1	EEF1A2	Multiple_Complex	6.06	8.76	-6.51	1.11E-10	8.92E-09
TC1200010130.hg.1	SOX5	Multiple_Complex	5.08	7.79	-6.56	4.02E-10	2.57E-08
TC1100013109.hg.1	GRAMD1B	Multiple_Complex	6.12	8.84	-6.6	2.45E-11	2.64E-09
TC0700011675.hg.1	SEMA3D	Multiple_Complex	3.9	6.62	-6.6	9.44E-07	1.84E-05
TC0700008252.hg.1	CROT	Multiple_Complex	3.02	5.75	-6.64	1.78E-10	1.33E-08
TC1900007975.hg.1	ZNF570	Multiple_Complex	3.9	6.64	-6.68	1.63E-08	5.92E-07
TC2100007491.hg.1	CBS	Multiple_Complex	5.36	8.11	-6.74	6.75E-10	3.97E-08
TC0900012157.hg.1	AKAP2	Multiple_Complex	5.55	8.3	-6.75	1.18E-09	6.47E-08
TC0X00010198.hg.1	HDX	Multiple_Complex	4.08	6.84	-6.76	1.31E-09	7.12E-08
TC1200008683.hg.1	CHST11	Multiple_Complex	6.83	9.6	-6.79	8.81E-11	7.47E-09
TC0900012126.hg.1	ANKRD20A3	Multiple_Complex	3.99	6.76	-6.81	7.25E-09	2.99E-07
TC0500012044.hg.1	FSTL4	Multiple_Complex	5.37	8.15	-6.89	2.48E-09	1.23E-07

TC1000007641.hg.1	DKK1	Multiple_Complex	3.83	6.64	-7.02	5.37E-07	1.14E-05
TC0700008389.hg.1	ASB4	Multiple_Complex	5.87	8.69	-7.04	1.39E-09	7.44E-08
TC0600007613.hg.1	HSPA1A	Coding	9.14	11.95	-7.06	1.17E-10	9.28E-09
TC0500012388.hg.1	DPYSL3	Multiple_Complex	4.64	7.46	-7.06	3.61E-10	2.34E-08
TC0X00010877.hg.1	FAM122B	Multiple_Complex	7.14	9.96	-7.07	4.90E-10	3.03E-08
TC0600012652.hg.1	FAXC	Multiple_Complex	5.28	8.11	-7.1	1.58E-10	1.21E-08
TC0700013578.hg.1	SEMA3A	Multiple_Complex	8.54	11.4	-7.26	8.04E-11	6.94E-09
TC2200007312.hg.1	LGALS1	Multiple_Complex	7.62	10.49	-7.3	3.64E-11	3.63E-09
TC1900008692.hg.1	ZNF480	Multiple_Complex	5.3	8.17	-7.32	1.74E-10	1.31E-08
TC1000012427.hg.1	AKR1C1	Multiple_Complex	5.22	8.09	-7.34	1.13E-10	9.00E-09
TC0500012870.hg.1	STC2	Multiple_Complex	5.49	8.37	-7.36	1.24E-10	9.69E-09
TC0X00010326.hg.1	PCDH19	Multiple_Complex	4.65	7.54	-7.38	6.94E-11	6.20E-09
TC1700008677.hg.1	CACNG4	Coding	5.19	8.08	-7.39	1.62E-10	1.23E-08
TC1700011919.hg.1	CEP295NL	Multiple_Complex	6.46	9.35	-7.4	4.47E-10	2.81E-08
TC1400008546.hg.1	LINC01296	Multiple_Complex	4.9	7.8	-7.44	9.64E-08	2.76E-06
TC0400007836.hg.1	CXCL8	Multiple_Complex	6.12	9.02	-7.45	1.04E-10	8.45E-09
TC0400011005.hg.1	RASSF6	Multiple_Complex	5.64	8.53	-7.45	9.22E-11	7.69E-09
TC0200014991.hg.1	GPR155	Multiple_Complex	4.65	7.55	-7.45	1.65E-09	8.72E-08
TC0100013339.hg.1	RUNX3	Multiple_Complex	4.6	7.51	-7.51	8.30E-11	7.12E-09
TC2000006627.hg.1	CHGB	Multiple_Complex	5.7	8.62	-7.57	1.95E-11	2.16E-09
TC1700011435.hg.1	ICAM2	Multiple_Complex	4.93	7.86	-7.65	8.36E-11	7.14E-09
TC0500008202.hg.1	FAM174A	Multiple_Complex	4.24	7.18	-7.7	2.29E-07	5.61E-06
TC0X00007781.hg.1	ZNF711	Multiple_Complex	5.33	8.29	-7.73	4.78E-09	2.09E-07
TC0600007495.hg.1	HLA-A	Multiple_Complex	7.72	10.68	-7.82	3.31E-11	3.43E-09
TC1300009598.hg.1	GPR183	Coding	4.21	7.18	-7.83	1.08E-09	5.96E-08
TC1900011946.hg.1	FBXO27	Multiple_Complex	5.18	8.15	-7.83	2.71E-09	1.31E-07
TC1000007913.hg.1	H2AFY2	Coding	6.82	9.79	-7.85	9.10E-11	7.66E-09
TC1400006853.hg.1	AKAP6	Multiple_Complex	4.38	7.37	-7.94	3.35E-08	1.10E-06
TC1000006604.hg.1	AKR1E2	Multiple_Complex	4.91	7.91	-7.98	1.06E-09	5.88E-08
TC1300009218.hg.1	DACH1	Multiple_Complex	4.99	7.99	-7.99	7.10E-11	6.32E-09
TC1200006645.hg.1	CD4	Multiple_Complex	4.21	7.21	-8.01	2.32E-11	2.52E-09
TC1300008916.hg.1	CAB39L	Multiple_Complex	5.14	8.14	-8.01	8.33E-12	1.09E-09
TC1000011363.hg.1	RNLS	Multiple_Complex	4.8	7.81	-8.07	5.18E-11	4.83E-09
TC1600008128.hg.1	CMTM3	Multiple_Complex	4.82	7.84	-8.11	2.43E-10	1.69E-08
TC2200008654.hg.1	MFNG	Multiple_Complex	6.55	9.57	-8.12	1.34E-11	1.58E-09
TC0200010696.hg.1	SPAG16	Multiple_Complex	3.75	6.77	-8.12	4.50E-09	1.99E-07
TC1000008628.hg.1	ABCC2	Multiple_Complex	6.35	9.4	-8.25	3.55E-10	2.31E-08
TC0100011661.hg.1	MARK1	Multiple_Complex	3.9	6.96	-8.37	9.17E-11	7.68E-09
TC0100015333.hg.1	PTPN22	Multiple_Complex	3.6	6.68	-8.49	3.89E-09	1.78E-07
TC0X00009867.hg.1	SPIN4	Coding	3.72	6.8	-8.49	7.48E-09	3.05E-07

TC0500013291.hg.1	FAM173B	Multiple_Complex	6.12	9.24	-8.71	1.34E-09	7.24E-08
TC0200009095.hg.1	INHBB	Coding	5.3	8.42	-8.72	2.54E-07	6.12E-06
TC1700011558.hg.1	SLC16A6	Multiple_Complex	4.45	7.59	-8.8	1.45E-07	3.88E-06
TC1100011966.hg.1	SLC36A4	Multiple_Complex	4.26	7.43	-9.01	4.31E-09	1.93E-07
TC0700012684.hg.1	AKR1B1	Multiple_Complex	5.5	8.68	-9.01	3.43E-12	5.31E-10
TC0100010609.hg.1	DNM3	Multiple_Complex	3.75	6.94	-9.1	9.26E-11	7.70E-09
TC1800006589.hg.1	PTPRM	Multiple_Complex	3.57	6.77	-9.17	1.62E-10	1.23E-08
TC1200012748.hg.1	CD163L1	Multiple_Complex	5.02	8.23	-9.25	8.38E-12	1.09E-09
TC0300006827.hg.1	UBE2E2	Multiple_Complex	4.44	7.65	-9.25	4.71E-12	6.73E-10
TC1200009587.hg.1	CACNA2D4	Multiple_Complex	5.34	8.56	-9.3	2.86E-10	1.94E-08
TC0100016018.hg.1	CRABP2	Coding	4.54	7.78	-9.43	9.85E-11	8.13E-09
TC0200009955.hg.1	CYBRD1	Multiple_Complex	3.42	6.68	-9.55	5.60E-11	5.16E-09
TC0100016664.hg.1	COLGALT2	Multiple_Complex	6.11	9.42	-9.93	1.00E-11	1.26E-09
TC14_GL000194v1_random0000643	MAFIP	Multiple_Complex	7.03	10.39	-10.26	5.43E-12	7.46E-10
TC1600008617.hg.1	CDH13	Multiple_Complex	4.49	7.87	-10.39	3.36E-11	3.46E-09
TC0700008582.hg.1	SERPINE1	Multiple_Complex	5.46	8.84	-10.44	7.18E-11	6.32E-09
TC0700009236.hg.1	CALD1	Multiple_Complex	5.5	8.93	-10.71	9.63E-12	1.22E-09
TC1300006658.hg.1	WASF3	Multiple_Complex	3.82	7.26	-10.83	3.00E-09	1.44E-07
TC0200015865.hg.1	SCG2	Coding	4.84	8.29	-10.94	7.13E-11	6.32E-09
TSUnmapped00000661.hg.1	INPP5D	Coding	3.89	7.34	-10.95	3.67E-11	3.64E-09
TC1700010982.hg.1	SKAP1	Multiple_Complex	4.94	8.4	-10.96	4.18E-11	4.09E-09
TC1700010643.hg.1	KRT40	Coding	5.1	8.57	-11.12	2.03E-11	2.23E-09
TC0300007657.hg.1	C3orf14	Multiple_Complex	3.16	6.64	-11.14	6.39E-11	5.73E-09
TC0600008473.hg.1	SMAP1	Multiple_Complex	5.34	8.84	-11.28	3.38E-10	2.22E-08
TC1700009318.hg.1	FAM101B	Multiple_Complex	4.48	7.98	-11.36	2.08E-08	7.34E-07
TC1700008186.hg.1	IGF2BP1	Multiple_Complex	5.97	9.48	-11.38	1.34E-12	2.55E-10
TC0200008501.hg.1	ITPRIPL1	Coding	4.81	8.39	-11.89	2.43E-10	1.69E-08
TC0300012718.hg.1	PLOD2	Multiple_Complex	4.45	8.04	-12.03	6.04E-10	3.62E-08
TC1400009194.hg.1	FERMT2	Multiple_Complex	4.77	8.37	-12.14	5.91E-11	5.35E-09
TC0500008309.hg.1	CAMK4	Multiple_Complex	4.82	8.45	-12.35	7.00E-12	9.27E-10
TC0700013583.hg.1	MTERF1	Multiple_Complex	4.26	7.89	-12.36	2.26E-12	3.96E-10
TC1800006675.hg.1	APCDD1	Multiple_Complex	5.52	9.17	-12.54	1.40E-12	2.63E-10
TC0800008235.hg.1	FAM92A1	Multiple_Complex	4.36	8.03	-12.72	2.04E-12	3.65E-10
TC1700012084.hg.1	NOTUM	Multiple_Complex	6.26	9.94	-12.81	1.40E-11	1.64E-09
TC0100017844.hg.1	NID1	Multiple_Complex	4.87	8.57	-12.95	3.92E-12	5.88E-10
TC2200009271.hg.1	APOBEC3C	Multiple_Complex	4.58	8.28	-13.05	1.33E-11	1.57E-09
TC0X00007668.hg.1	UPRT	Multiple_Complex	4.17	7.9	-13.28	3.81E-11	3.77E-09
TC0100017984.hg.1	AKT3	Multiple_Complex	3.83	7.57	-13.35	2.20E-10	1.57E-08
TC1200008028.hg.1	MSRB3	Multiple_Complex	4.17	7.91	-13.35	4.88E-11	4.59E-09
TC0100017834.hg.1	GNG4	Multiple_Complex	6.86	10.59	-13.36	1.44E-12	2.69E-10

TC0600008486.hg.1	OGFRL1	Multiple_Complex	3.12	6.88	-13.56	1.25E-12	2.49E-10
TC0400010694.hg.1	ERVMER34-1	Multiple_Complex	4.15	7.92	-13.67	3.42E-13	1.03E-10
TC1000008891.hg.1	DUSP5	Multiple_Complex	4.78	8.57	-13.78	2.98E-12	4.80E-10
TC0800010382.hg.1	SNAI2	Coding	5.31	9.1	-13.81	1.24E-10	9.69E-09
TC0900011305.hg.1	TNC	Multiple_Complex	6.79	10.58	-13.82	9.73E-13	2.07E-10
TC0X00011302.hg.1	MSN	Multiple_Complex	7.06	10.87	-14.05	1.19E-11	1.45E-09
TC1700010641.hg.1	KRT23	Multiple_Complex	4.72	8.54	-14.09	1.14E-11	1.40E-09
TSUnmapped00000135.hg.1	INPP5D	Coding	4.35	8.21	-14.58	1.55E-09	8.21E-08
TC0900009740.hg.1	ELAVL2	Multiple_Complex	4.15	8.03	-14.71	9.23E-12	1.18E-09
TC1500010251.hg.1	HOMER2	Multiple_Complex	5.63	9.51	-14.77	1.69E-11	1.90E-09
TC0200007261.hg.1	QPCT	Multiple_Complex	5.2	9.1	-14.98	3.34E-13	1.03E-10
TC1400008705.hg.1	SLC7A8	Multiple_Complex	5.44	9.4	-15.53	4.69E-12	6.73E-10
TC0X00007744.hg.1	SH3BGRL	Multiple_Complex	4.87	8.85	-15.77	1.58E-11	1.79E-09
TC2000008218.hg.1	C20orf194	Multiple_Complex	2.82	6.84	-16.15	8.14E-12	1.07E-09
TC1000010132.hg.1	MKX	Coding	4.1	8.12	-16.25	1.12E-08	4.32E-07
TC0700009977.hg.1	PDGFA	Multiple_Complex	5.65	9.71	-16.63	4.95E-13	1.30E-10
TC0100011755.hg.1	DNAH14	Multiple_Complex	2.93	6.99	-16.75	8.52E-14	3.58E-11
TC1100013165.hg.1	SLC43A3	Multiple_Complex	6.04	10.12	-16.91	2.36E-13	8.03E-11
TC0300009412.hg.1	SERPINI1	Multiple_Complex	4.01	8.1	-17.05	1.01E-10	8.26E-09
TC0300012764.hg.1	HLTF	Multiple_Complex	3.53	7.64	-17.27	3.45E-11	3.50E-09
TC0600012709.hg.1	POPDC3	Multiple_Complex	3.94	8.05	-17.28	1.67E-12	3.03E-10
TC1700012457.hg.1	AXIN2	Multiple_Complex	5.89	10.01	-17.36	1.35E-13	5.09E-11
TC0100014743.hg.1	TTLL7	Multiple_Complex	4.28	8.42	-17.65	8.20E-13	1.85E-10
TC0200016418.hg.1	ZNF512	Multiple_Complex	3.82	8.01	-18.27	4.08E-13	1.15E-10
TC0300009474.hg.1	CLDN11	Multiple_Complex	6.11	10.32	-18.57	6.87E-12	9.16E-10
TC1600007931.hg.1	LPCAT2	Multiple_Complex	4.34	8.57	-18.83	4.22E-12	6.22E-10
TC0100010140.hg.1	SYT11	Coding	4.78	9.06	-19.46	3.67E-12	5.62E-10
TC0800010631.hg.1	TTPA	Multiple_Complex	4.27	8.55	-19.48	4.58E-10	2.86E-08
TC0500009023.hg.1	JAKMIP2-AS1	Multiple_Complex	3.62	7.92	-19.61	2.47E-13	8.13E-11
TC1200008275.hg.1	SYT1	Multiple_Complex	6.72	11.05	-20.02	4.89E-13	1.29E-10
TC0X00010926.hg.1	MAP7D3	Multiple_Complex	4.46	8.81	-20.41	7.88E-11	6.84E-09
TC0300007033.hg.1	MLH1	Multiple_Complex	3.94	8.29	-20.42	2.85E-13	9.14E-11
TC0200007908.hg.1	ANTXR1	Multiple_Complex	3.7	8.07	-20.7	2.59E-12	4.30E-10
TC0X00008002.hg.1	NGFRAP1	Multiple_Complex	5.06	9.43	-20.71	5.90E-13	1.42E-10
TC1700006524.hg.1	SERPINF1	Multiple_Complex	6.05	10.44	-20.92	4.97E-14	2.54E-11
TC1000007199.hg.1	ZEB1	Multiple_Complex	5.25	9.65	-21.01	6.86E-13	1.62E-10
TC0X00009356.hg.1	TMEM47	Coding	6.55	11	-21.97	4.15E-14	2.41E-11
TC0100011566.hg.1	PROX1	Multiple_Complex	6.34	10.82	-22.19	8.93E-14	3.62E-11
TC1200012752.hg.1	KLRC3	Coding	3.99	8.5	-22.74	2.52E-12	4.25E-10
TC0600008931.hg.1	GRIK2	Multiple_Complex	4.76	9.31	-23.51	6.23E-14	2.85E-11

TC0700013356.hg.1	EPDR1	Coding		3.68	8.27	-23.93	4.70E-14	2.52E-11
TC1200009800.hg.1	SLC2A3	Multiple_Complex		4.77	9.38	-24.45	8.08E-13	1.84E-10
TC2000007005.hg.1	CST7	Coding		5.33	9.98	-25.17	2.57E-12	4.30E-10
TC1000007703.hg.1	BICC1	Multiple_Complex		4.18	8.86	-25.56	8.01E-14	3.51E-11
TC0700010334.hg.1	AGMO	Multiple_Complex		3.72	8.43	-26.26	3.62E-13	1.06E-10
TC0100008450.hg.1	PRKAA2	Multiple_Complex		4.66	9.38	-26.31	2.39E-13	8.03E-11
TC1100013135.hg.1	OR51B5	Multiple_Complex		3.94	8.67	-26.55	5.27E-14	2.54E-11
TC0X00007686.hg.1	PBDC1	Multiple_Complex		5.21	9.94	-26.57	1.24E-14	1.02E-11
TC0X00007559.hg.1	GJB1	Coding		4.7	9.44	-26.7	1.66E-14	1.19E-11
TC0100009938.hg.1	MLLT11	Multiple_Complex		4.82	9.62	-27.75	2.41E-14	1.56E-11
TC0100008874.hg.1	PRKACB	Multiple_Complex		4.01	8.91	-29.93	5.84E-14	2.73E-11
TC1700009694.hg.1	MYH10	Multiple_Complex		5.52	10.49	-31.27	3.68E-14	2.19E-11
TC1500009335.hg.1	MYEF2	Multiple_Complex		3.14	8.24	-34.21	5.25E-13	1.33E-10
TC0X00007267.hg.1	MAGED1	Multiple_Complex		3.6	8.72	-34.81	4.33E-15	5.16E-12
TC1300008840.hg.1	LCP1	Multiple_Complex		4.98	10.16	-36.25	1.10E-12	2.29E-10
TC1100007727.hg.1	FAM111B	Multiple_Complex		3.35	8.78	-43.17	1.09E-14	9.37E-12
TC1100012262.hg.1	RDX	Multiple_Complex		3.81	9.27	-44.01	2.70E-14	1.70E-11
TC1600007819.hg.1	NKD1	Multiple_Complex		6.23	11.78	-46.84	1.95E-13	6.76E-11
TC1200012753.hg.1	KLRC2	Coding		3.7	9.28	-47.72	7.04E-15	6.86E-12
TC1100012186.hg.1	MSANTD4	Multiple_Complex		3.97	9.54	-47.83	8.87E-15	7.93E-12
TC1100012019.hg.1	SESN3	Multiple_Complex		3.77	9.47	-51.71	2.94E-15	4.51E-12
TC0300012048.hg.1	ZBTB20	Multiple_Complex		4.12	9.85	-53.01	1.19E-12	2.42E-10
TC0500007738.hg.1	MAP1B	Multiple_Complex		3.9	9.65	-53.55	4.82E-14	2.52E-11
TC1300009673.hg.1	TEX30	Multiple_Complex		4.02	9.78	-54.1	7.41E-15	6.91E-12
TC1200011495.hg.1	LUM	Multiple_Complex		3.5	9.36	-57.93	3.26E-12	5.10E-10
TC1000007714.hg.1	PHYHIPL	Multiple_Complex		3.15	10.25	-136.91	2.92E-15	4.51E-12
