# Supplementary material for: A prognostic six‐gene expression risk‐score derived from proteomic profiling of the metastatic colorectal cancer secretome
Source: J Pathol Clin Res. 2022 Sep 22;8(6):495–508. doi: 10.1002/cjp2.294 (PMC9535096; doi:10.1002/cjp2.294)
Supplement: Supplementary file 6 — Table S4. Determination of HRs and long rank P values for the up‐regulated secreted proteins in four CRC datasets [file CJP2-8-495-s003.pdf]

A prognostic six-gene expression risk-score derived from proteomic profiling of the metastatic colorectal cancer secretome

J Robles *et al.* *J Pathol Clin Res* DOI: https://doi.org/10.1002/cjp2.294

Table S4. Determination of hazard ratios and long rank p-values for the up-regulated secreted proteins in four CRC datasets

	GSE39582		COADREAD		GSE14333		GSE17538	
Gene symbol	p-value	Hazard ratio	p-value	Log rank	p-value	Hazard ratio	p-value	Hazard ratio
CD109	0.02	1.2956	0.01385	6.057	0	1.6524	0	1.7591
IGFBP3	0.0071	1.6118	0.04106	4.174	0	2.0961	0.0069	1.8983
NPC2	0.1744	1.5669	0.03759	4.323	2.00E-04	1.4919	0.0295	2.6606
LTBP1	0.0661	1.4016	0.03585	3.767	0.0055	1.8046	0.0013	2.8292
BMP1	0.1212	1.8735	0.01062	6.527	0.01	2.1465	0.5968	1.3771
PSAP	0.0768	1.985	0.0379	4.31	0.0152	2.9554	0.3445	1.4141
THBS2	0.0322	1.2262	0.02348	5.133	0.264	1.5466	0.003	1.6377
NRP1	0.3822	1.8482	0.00126	10.4	0.7581	1.0455	0.5044	0.6092
TPP1	0.0385	3.2131	0.0601	3.535	0.9873	0.9967	0.7951	0.7646
LAMC1	5.00E-04	2.5411	0.6767	0.1739	0.0024	2.0427	0.006	2.7683
NOV	0.0319	1.9879	0.05479	3.688	0.0024	1.6052	0.9367	0.9687
LAMC2	7.00E-04	1.8411	0.2996	1.076	0.0842	1.5005	0.1801	1.8588
HSPG2	0.0348	1.8184	0.09374	2.809	0.3111	1.1077	0.7663	0.8825
HEXA	0.243	1.8075	0.9236	0.009195	0.7553	0.9574	0.8831	1.1413
PTGFRN	0.0904	1.7689	0.478	0.5035	0.9619	1.0182	0.2404	1.787
GANAB	0.2098	1.7495	0.08985	2.877	0.388	0.388	0.9324	1.0438
CTSB	0.0049	1.7402	0.5535	0.3511	0.0043	1.9694	0.1468	1.4419
ALCAM	0.1004	1.6781	0.2696	1.219	0.5062	1.1207	4.00E-04	3.7047
CTSC	0.0813	1.6774	0.7392	0.1108	0.8622	1.0493	0.117	1.7016
GAA	0.0859	1.647	0.4783	0.5027	1.00E-04	2.8347	0.3413	1.4954
PLOD1	0.0675	1.6371	0.698	0.1506	0.0298	1.9182	0.0235	2.67
GRN	0.1495	1.6027	0.6071	0.2644	0.0623	1.7424	0.7972	0.8884
LAMA5	0.0231	1.5996	0.2923	1.109	0.0039	2.0014	0.0113	2.6212
PPT1	0.1568	1.5741	0.5064	0.4414	0.6207	1.265	0.095	2.6454
S100A6	0.213	1.5644	0.7033	0.1451	0.0048	5.1506	0.7599	0.8382
PDIA3	0.246	1.5409	0.5985	0.2773	0.6384	0.8309	0.1216	2.1645
ULBP2	0.085	1.525	0.2285	1.45	0.0323	1.3034	0.1266	2.4218
VEGFA	0.0962	1.5076	0.1222	2.388	0.001	2.5416	0.012	2.5832
PLOD3	0.1231	1.48	0.902	0.01515	0.5057	1.1895	0.8977	1.0513

GNS	0.1131	1.4728	0.2032	1.619	0.1707	1.3089	0.5703	0.8257
CTGF	0.0508	1.3931	0.3211	0.9845	4.00E-04	1.6771	0.0081	1.6872
ADAM10	0.3101	1.3856	0.9202	0.01003	0.0543	2.2224	0.0361	3.9648
AGRN	0.5992	1.3435	0.668	0.184	0.6267	1.0828	0.1849	0.3931
QSOX1	0.2506	1.3289	0.4507	0.5689	0.3704	1.131	0.5131	1.2905
CDH3	0.0852	1.323	0.08706	2.928	0.3374	1.1467	0.5938	1.1811
NUCB1	0.4193	1.2924	0.3527	0.8637	0.2514	1.4759	0.4805	0.7022
LOXL1	0.0747	1.2909	0.06006	3.536	0.0205	1.4208	0.0157	1.662
PRDX4	0.3601	1.2768	0.2802	1.166	0.2682	0.6751	0.9596	0.977
SLC39A10	0.2215	1.2696	0.575	0.3143	0.198	1.3889	0.2282	1.4685
PROS1	0.11	1.259	0.6347	0.2258	0.0522	1.4468	0.1343	1.4528
P4HB	0.588	1.2574	0.3549	0.8558	0.8088	1.1372	0.5288	0.7366
SERPINB5	0.4883	1.2559	0.2737	1.198	0.0426	1.3238	0.239	2.1301
SLK	0.3198	1.2549	0.1294	2.299	0.0203	2.2544	0.01	2.5035
CTSD	0.4528	1.2144	0.178	1.814	9.00E-04	1.9993	0.8652	1.0648
SPON1	0.0786	1.2059	0.07535	3.162	2.00E-04	1.5696	0.0129	1.7968
ERO1L	0.5149	1.1947	0.48569	0.48606	8.00E-04	3.5221	0.163	1.8375
PHGDH	0.2482	1.1892	0.8596	0.03128	0.7569	1.0489	0.1006	1.5771
ASAH1	0.5747	1.171	0.2926	1.107	0.8719	1.0364	0.2755	0.7467
LAMA3	0.5253	1.1529	0.1141	2.496	0.1161	1.3057	0.7747	0.8557
HSP90B1	0.684	1.1473	0.5465	0.3637	0.5886	0.8486	0.0228	2.9045
LGMN	0.7031	1.1066	0.9499	0.003942	0.0592	1.7302	0.0788	1.9799
LAMB1	0.6742	1.0919	0.3258	0.9654	0.3751	1.2573	0.003	2.7317
GALNT1	0.6873	1.0857	0.5607	0.3385	0.0277	1.7486	0.0944	1.8534
SPINT1	0.7832	1.0683	0.6844	0.1652	0.9597	0.9849	0.2315	0.4512
LSR	0.834	1.0632	0.7559	0.09665	0.8359	1.0569	0.7918	0.9113
CALU	0.8127	1.0578	0.9433	0.005062	0.0026	2.5366	0.1688	1.8709
ALDH1A1	0.6419	1.0438	0.1064	2.608	0.411	1.0427	0.2712	1.1651
RARRES1	0.7446	1.0307	0.1542	2.03	0.3076	1.1374	0.1745	1.2398
S100P	0.8989	1.0172	0.2824	1.156	0.9236	1.0156	0.2384	0.7658
IGSF8	0.9851	1.0062	0.05148	3.792	0.8922	1.0353	0.1087	0.4672
SNX6	0.9999	1	0.7885	0.07194	0.0207	2.6331	0.3333	1.8386
MESDC2	0.9956	0.9979	0.5571	0.3447	0.2392	0.5876	0.2546	2.005

LIPA	0.9842	0.9954	0.1414	2.162	0.113	1.4719	0.3346	1.3545
LYZ	0.7942	0.983	0.4387	0.5996	0.4254	0.9497	0.4956	1.0701
GCNT3	0.8237	0.9795	0.3175	0.9994	0.9658	0.9979	0.1215	1.2872
MGAT5	0.9554	0.9657	0.3778	0.7779	0.5984	0.9269	0.0771	0.1073
NEU1	0.8644	0.9644	0.2534	1.304	0.6495	0.9201	0.9463	0.9796
MMP1	0.5459	0.9596	0.2698	1.218	0.1014	0.8808	0.3935	1.0974
CTSA	0.8595	0.9574	0.6495	0.2065	0.6757	1.0953	0.0114	0.4259
REG4	0.3577	0.9533	0.01208	6.299	0.9921	1.0006	0.2461	1.111
PRKCSH	0.8837	0.9512	0.6439	0.2137	0.823	1.0798	0.2954	0.67
PLBD2	0.8588	0.9511	0.4992	0.4566	2.00E-04	3.5995	0.3919	1.6691
CALR	0.8075	0.9501	0.03331	4.53	0.7715	1.0673	0.5132	1.2533
DAG1	0.8933	0.9442	0.7266	0.1222	0.8317	0.9026	0.9721	1.0225
CTH	0.7174	0.9422	0.9469	0.00443	0.4216	0.8714	0.4418	1.2096
CPVL	0.6291	0.9325	0.05216	3.771	0.7459	1.0545	0.4709	1.1842
TFF1	0.3417	0.93	0.3375	0.92	0.5131	0.9392	0.6972	1.0513
A2ML1	0.9251	0.9299	NaN	NaN	0.656	0.9124	0.4187	0.5447
CA2	0.2754	0.9252	0.1807	1.792	0.7827	0.9765	0.2773	1.1398
CD44	0.7193	0.9138	0.2464	1.343	0.7069	1.1512	0.7696	0.8752
CD9	0.6193	0.9081	0.5574	0.3442	0.6169	1.0658	0.1967	0.5241
DNASE2	0.6879	0.8913	0.3241	0.9722	0.2392	1.3718	0.3057	0.4465
PCSK9	0.5077	0.8908	0.9876	0.0002423	0.9035	1.0149	0.2158	1.4539
VGF	0.7122	0.8798	0.02925	4.753	0.3111	1.2458	0.4695	0.6259
IGFBP2	0.1287	0.8726	0.9423	0.005233	0.4318	0.9462	0.8938	0.9804
HEXB	0.7282	0.8695	*0.04817*	*3.904*	0.0028	0.2935	0.0954	0.3609
S100A14	0.3286	0.8649	0.1917	1.704	0.2978	1.201	0.5081	1.1719
CXCL1	0.1606	0.8567	*0.03249*	*4.573*	0.1969	0.8452	0.846	0.9683
TFF3	0.1568	0.8557	0.4256	0.6348	0.0074	0.7379	0.738	0.9403
MANF	0.6108	0.8465	0.1583	1.99	0.1839	0.6316	0.792	1.143
EPCAM	0.4497	0.8292	0.4104	0.6776	0.1971	0.4401	0.0364	0.4471
CLSTN1	0.5332	0.8231	0.6501	0.2057	0.5019	1.3132	0.1729	0.4552
SDF4	0.4657	0.7978	0.5035	0.4476	0.1896	0.6743	0.0022	0.1663
TSPAN6	0.1757	0.7907	0.02502	5.022	0.1409	0.7667	0.0118	0.5631
B2M	0.4973	0.7887	0.8842	0.02121	0.5856	1.1078	0.862	0.9342

DSG2	0.3222	0.771	0.1105	2.547	0.2076	0.6522	0.2399	0.6615
APLP2	0.4324	0.7655	0.9589	0.002658	0.9759	0.9916	0.8262	0.914
ERP44	0.5458	0.7618	0.9355	0.006544	0.7902	1.121	0.1711	5.2242
NENF	0.4509	0.7489	0.584	0.2999	0.5217	1.2254	0.8634	1.135
CLDN3	0.0549	0.7468	0.8848	0.02101	0.0591	0.7683	0.0078	0.5404
ANP32A	0.4757	0.7411	0.4179	0.6563	0.3272	1.4281	0.764	1.1957
AGR2	0.0097	0.731	0.6652	0.1873	0.3414	1.2668	0.7959	1.052
HSPA5	0.3638	0.7297	0.5578	0.3435	0.8701	0.9184	0.3147	2.2142
ERP29	0.4354	0.7269	0.7764	0.08065	0.0069	0.2794	0.5972	1.3821
GLA	0.218	0.7169	0.09131	2.851	0.8917	1.0393	0.9172	0.9538
CDH1	0.2246	0.6955	0.3646	0.822	0.5701	0.897	0.4102	0.8128
TOR1B	0.275	0.6954	0.957	0.002901	0.3788	0.7503	0.4325	0.7011
LDLR	0.6168	0.6901	0.3958	0.7209	0.2581	1.1174	8.00E-04	3.3009
FUCA2	0.3865	0.6896	0.2031	1.62	0.6476	0.8265	0.9469	1.0434
C19orf10	0.2752	0.684	0.689	0.1602	0.8568	0.923	0.9417	1.0404
ST14	0.1389	0.6446	0.5001	0.4547	0.1837	0.7469	0.2058	0.3843
RNASE4	0.0092	0.6302	0.5348	0.3852	0.441	1.1609	0.7306	1.092
CLU	0.4452	0.6276	0.02752	4.858	0.2707	1.1253	0.0055	1.8286
ANG	0.0029	0.6113	0.789	0.07161	0.9497	0.9911	0.6965	1.11
PDIA4	0.1292	0.6045	0.1221	2.39	0.0854	0.5913	0.42	1.4082
MINPP1	0.0387	0.5975	0.7412	0.109	0.1405	0.6555	0.2797	1.4438
P4HA1	0.5168	0.5651	0.08714	2.926	0.9862	0.9981	0.1813	0.0935
IDH1	0.0044	0.3785	0.4034	0.6981	0.1915	0.6227	0.7315	0.7789
SHMT1	0.1928	0.3494	0.6704	0.1812	0.8556	1.0329	0.7201	1.2531
