# Supplementary material for: A prognostic six‐gene expression risk‐score derived from proteomic profiling of the metastatic colorectal cancer secretome
Source: J Pathol Clin Res. 2022 Sep 22;8(6):495–508. doi: 10.1002/cjp2.294 (PMC9535096; doi:10.1002/cjp2.294)
Supplement: Supplementary file 7 — Table S5. HRs of the six selected genes (SEC6) in different datasets [file CJP2-8-495-s008.pdf]

A prognostic six-gene expression risk-score derived from proteomic profiling of the metastatic colorectal cancer secretome

J Robles *et al.* *J Pathol Clin Res* DOI: https://doi.org/10.1002/cjp2.294

Table S5. Hazard ratios of the 6 selected genes (SEC6) in different datasets

	GSE14333		GSE17538		GSE39582		TCGA COADREAD	
	**HR (95% CI)**	**p-value**	**HR (95% CI)**	**p-value**	**HR (95% CI)**	**p-value**	**HR (95% CI)**	**p-value**
BMP1	2.15 (1.20-3.84)	0.010	1.38 (0.42-4.51)	0.597	1.87 (0.98-4.86)	0.121	1.24 (0.88-1.75)	0.226
CD109	1.65 (1.33-2.05)	5.31E-06	1.76 (1.34-2.31)	4.10E-05	1.30 (1.02-1.59)	0.020	1.07 (0.94-1.21)	0.335
IGFBP3	2.10 (1.55-2.84)	1.89E-06	1.90 (1.19-3.02)	0.007	1.61 (1.04-2.13)	0.007	1.24 (1.01-1.53)	0.044
LTBP1	1.81 (1.19-2.74)	0.006	2.83 (1.50-3.36)	0.001	1.40 (0.92-1.90)	0.066	1.19 (0.97-1.47)	0.100
NPC2	1.49 (1.21-3.01)	2.00E-04	2.66 (1.10-6.42)	0.030	1.57 (0.89-3.26)	0.174	1.79 (1.11-2.89)	0.018
PSAP	2.95 (1.23-7.09)	0.015	2.24 (0.69-7.24)	0.178	1.99 (0.95-4.51)	0.077	1.18 (0.78-1.77)	0.435
