# Supplementary material for: A prognostic six‐gene expression risk‐score derived from proteomic profiling of the metastatic colorectal cancer secretome
Source: J Pathol Clin Res. 2022 Sep 22;8(6):495–508. doi: 10.1002/cjp2.294 (PMC9535096; doi:10.1002/cjp2.294)
Supplement: Supplementary file 8 — Table S6. List of genes associated to cell migration, adhesion, EC organisation, and cell cycle in high‐risk CRC patients [file CJP2-8-495-s001.pdf]

A prognostic six-gene expression risk-score derived from proteomic profiling of the metastatic colorectal cancer secretome

J Robles *et al.* *J Pathol Clin Res* DOI: https://doi.org/10.1002/cjp2.294

Table S6. List of genes associated with cell migration, adhesion, EC organisation and cell cycle in high risk CRC patients

Upregulated in high risk				Downregulated in high risk	
**All**	**Cell migration**	**Cell adhesion**	**EC organisation**	**All**	**Cell cyle**
PRICKLE1	VEGFC	EFEMP2	AEBP1	ZDHHC23	AURKA
LAYN	GLI3	HLX	EFEMP2	PDSS1	MYB
VEGFC	NRP2	GLI3	COL8A2	HOOK1	BRIP1
KCNE4	TNS1	NRP2	ITGAM	MAP7	KIF15
ZFPM2	ITGAM	COL8A2	COMP	BRI3BP	GMNN
AEBP1	NOX4	ITGAM	SFRP2	AURKA	CDK1
DFNA5	AMOTL1	COMP	COL6A2	MYB	TOP2A
TGFB3	MITF	SFRP2	ANTXR1	EPB41L5	BRCA1
GFPT2	SFRP2	COL6A2	JAM3	MARVELD2	RMI1
EFEMP2	SPOCK1	SPOCK1	TIMP2	AGMAT	DNA2
HLX	JAM3	ANTXR1	DDR2	PDP2	TOM1L1
COLEC12	SLIT2	JAM3	CYP1B1	BRIP1	E2F8
GLI3	EFS	EFS	FBN1	PIP5K1B	PTPN3
CDK14	DPYSL3	DDR2	CCDC80	SRPK1	RRM2
NRP2	DDR2	CYP1B1	COL10A1	EPT1	DHFR
DOK5	CYP1B1	FBN1	BGN	PARPBP	PRR11
COL8A2	CDH2	CCDC80	ITGA5	KIF15	WDR12
MRC2	ITGA5	CERCAM	OLFML2B	GMNN	MAD2L1
TNS1	AXL	CDH2	HTRA1	CDK1	NDC1
ITGAM	PLEKHO1	BOC	LOXL1	TOP2A	ZWINT
LOC102725271	SYDE1	NUAK1	ECM2	BRCA1	CDC25C
GAS1	ZEB2	MXRA8	EMILIN1	ZNF367	SMC2
NOX4	EMILIN1	ITGA5	P4HA3	RMI1	CCNA2
KIAA1462	FERMT2	AXL	ADAM12	LRPPRC	KIF11
IFFO1	PRKD1	AOC3	FAP	CMTM8	SPC25
AMOTL1	FGFR1	ECM2	SULF1	ZNF823	NCAPH
MAFB	FAP	MFGE8	LAMB2	CMC1	PLK4
BNC2	AKT3	TGFB1I1	COL8A1	DNA2	ANAPC1
NARR	MSN	ISLR	VCAN	MARS2	RAD51AP1
TSHZ3	HTR2B	EMILIN1	PXDN	CYCS	TPX2
MITF	SULF1	FERMT2	MMP19	NDUFAF4	PRIM1
MRAS	FLNA	ADAM12	SPARC	TOM1L1	DBF4
MSRB3	LAMB2	FAP	COL5A1	E2F8	MIS18A
NXN	VCAN	CDH11	COL5A2	PAICS	CENPK
COMP	PALLD	SCARF2	CRISPLD2	C2orf47	DSN1
PTRF	IGFBP6	MSN	DCN	PTPN3	RAB11FIP4

UBE2QL1	AKAP12	THBS3	TIMP1	RRM2	RFC3
CMTM3	SPARC	LGALS1	MFAP5	TMEM177	CENPA
CRYAB	THY1	FLNA	THBS1	HSPE1	KIF2A
SFRP2	COL5A1	LAMB2	COL6A3	ACP1	RACGAP1
COL6A2	KANK2	COL8A1	COL6A1	PIGX	BRCC3
SPOCK1	SERPINF1	FEZ1	JAM2	STRBP	HJURP
ANTXR1	GPNMB	VCAN	ADAMTS12	FUT4	SPAG5
FBXL7	GPC6	PALLD	TIE1	EPB41L4B	HMMR
JAM3	DCN	THY1	COL18A1	FARP2	TOPBP1
SLIT2	TIMP1	COL5A1	LAMA4	DHFR	RFC4
TSPYL5	FSTL1	PCDH7	CTSL	FZD5	CDK12
TIMP2	CSF1R	ENTPD1	ITGA11	CALML4	ATAD5
EHD2	MAP1B	MYL9	COL11A1	MAPKAPK5	GEN1
MIR100HG	PDGFC	GPNMB	ADAMTS2	AKAP1	BUB1B
HEG1	PTPRM	IGFBP7	COL1A2	PRR11	TTK
EFS	C5AR1	GPC6	COL3A1	TMPO	CDC25A
MXRA7	THBS4	HMCN1	PHLDB2	FAR2	CNOT1
DPYSL3	TREM2	THBS2	LUM	SRSF1	NEK2
PEA15	IL1R1	PTPRM	LOX	WDR12	SPC24
DDR2	PDGFRB	THBS4	FBLN2	PTCD3	DTL
C20orf194	ITGBL1	FSTL3	RECK	MAD2L1	NCAPG2
PRELP	CLIC4	ITGBL1	ITGB2	OCLN	BORA
CYP1B1	SPHK1	ACTN1	MMP14	NDC1	RAD54B
FRMD6	THBS1	THBS1	BMP1	ZWINT	NCAPD3
GLIS2	JAM2	COL6A3	ENG	CDC25C	MKI67
FBN1	IGFBP5	COL6A1	FLRT2	CASP6	OVOL1
CCDC80	EVL	VSIG4	LAMC1	CLRN3	CCNB1
CERCAM	DCLK1	JAM2	MMP2	SUCLG1	EXO1
TUBB6	ADAMTS12	FCGR2B	FBLN5	CDX1	NUSAP1
CNRIP1	ATP2B4	TNFSF4	PECAM1	DNAJA3	CEP55
PDLIM3	NRP1	ADAMTS12	SH3PXD2A	BDH1	KIF20B
COL10A1	TIE1	NRP1	POSTN	SMC2	PAXIP1
OSMR	IGFBP3	COL18A1	CTSK	CKAP2L	PBK
229479_at	MYLK	OLR1	LOXL2	HNF4G	CHEK2
CDH2	OLR1	LAMA4	ITGAX	MRPL3	SKP2
VIM	SDC2	CLDN11	TGFB1	CCNA2	RBL1
BOC	LAMA4	TENM4	LOXL3	LOC101927157	DDIAS
BGN	ITGA11	ITGA11	VCAM1	KIF11	RPS27A
CHST11	GYPC	PLXND1	SERPINH1	DEPDC1B	GINS1
PTGIS	CTHRC1	LRRC32	HSPG2	GUCY2C	E2F1

NUAK1	PLXND1	SSPN	ITGB5	NARS2	SUV39H2
MXRA8	COL1A2	COL3A1	FBLN1	SPC25	STIL
PPAPDC1A	COL3A1	PHLDB2	COL15A1	CDCA7	CCNE2
COPZ2	PHLDB2	LAPTM5	ADAMTSL4	NCAPH	NUF2
LOC101928916	VASH1	FBLN2	A2M	GGCT	MND1
ITGA5	C3AR1	ITGB2	COL1A1	GPX2	CKS2
RAB31	LOX	MMP14	COL4A2	PLK4	DCLRE1A
DACT3	RECK	NEXN	COL24A1	PANK1	CCNB2
OLFML2B	CCDC88A	SRPX	FN1	ANAPC1	BUB1
PLXDC2	ITGB2	ENG	SERPINE1	MFSD9	CKAP2
TSPAN4	MMP14	FLRT2	COL12A1	MRPS22	OIP5
AXL	NEXN	LAMC1	LTBP3	RAD51AP1	EZH2
HTRA1	TWIST1	DLC1	MMP16	DDC	CHEK1
PDLIM7	APOE	AIF1	COL16A1	MRPS35	SFPQ
VGLL3	ENG	FBLN5	MFAP2	TMEM97	CENPH
FNDC1	FLRT2	TENM3	ETS1	BEND3	SKA3
GGT5	LAMC1	GLI2	ITGB1	TPX2	HELLS
HOPX	DLC1	BCL6	NID2	PHB	GTSE1
EMP3	AIF1	PECAM1	DPT	PRIM1	NCAPG
SERPING1	MCC	FZD4	CAV1	ODC1	AUNIP
PLEKHO1	SLAMF8	TNFAIP6	PDPN	RPIA	KIF18A
EVC	TNFSF12	POSTN	SPP1	NIFK	E2F2
LOXL1	PECAM1	TMEM47	CAV2	SLMO2	FANCI
C10orf10	GNAI2	LOXL2	GREM1	TRIM2	CNOT11
AOC3	TNFAIP6	CSRP1	ADAM8	MARVELD3	CDT1
SYDE1	POSTN	SPARCL1	MATN3	ERI2	RMI2
ZEB2	NAV3	NLGN4X	ADAMTS5	RBM47	CENPN
PLEKHO2	LOXL2	CCL2	ICAM1	DBF4	MSH2
MAP1A	RARRES2	CD93	SH3PXD2B	MIS18A	CDC6
ECM2	CCL2	ITGAX	TNC	GAS2L3	KIF4A
FAM20C	FCER1G	TGFB1	COL5A3	LRRC31	MELK
MFGE8	TWIST2	APBB1IP	LRP1	HSDL2	ASPM
TGFB1I1	ITGAX	ILK	MYH11	KIAA1958	BRCA2
ISLR	TGFB1	STAB1	PDGFB	CENPK	PPAT
228481_at	ILK	PARVA	TGFB2	TFAM	MYO19
MAGI2-AS3	NDN	THEMIS2	MMP11	QRSL1	PTTG1
STON1	GLIPR2	KIF26B	COL4A1	DSN1	DSCC1
COX7A1	PARVA	LOXL3	ITGAV	RAB11FIP4	FBXO5
EMILIN1	THBD	CD84	VWF	PRR15L	ORC1
FERMT2	CD84	VCAM1	ITGB3	RAD18	BLM

TAGLN	VCAM1	SGCE	ITGA7	C4orf19	FIGNL1
ZNF521	RHOJ	ITGB5	MMP9	RFC3	CDCA3
PRKD1	ROR2	CXCL12	TGFBR1	CDS1	LIG3
P4HA3	ITGB5	FBLN1	LAMA2	MTIF2	CDCA8
FGFR1	CXCL12	CNTNAP1	PLOD1	SFXN4	GINS3
227618_at	FBLN1	COL15A1	FSCN1	IL17RB	NUP35
ADAM12	CLEC7A	PARVG	COL13A1	CENPA	CENPF
C1R	SYNPO2	COL1A1	ADAMTS1	KIF2A	FEN1
FXYD6	FGR	HAVCR2	COL14A1	RACGAP1	ERCC6L
LOC400043	COL1A1	HCK	MFAP4	BRCC3	NUP37
PPP1R18	HCK	APOD	ITGA1	SOWAHA	BIRC5
FAM19A5	RHOQ	PCDHB7	NID1	HSPD1	FANCD2
FAP	MAP3K3	FN1	ELN	MPC2	SMC4
CDH11	MEOX2	SERPINE1	ADAMTS6	ESRP1	TADA2A
LINC01279	LRRC15	NCKAP1L	ADAM19	PKP2	MCM6
NPR3	APOD	COL12A1	FOXC2	RTN4IP1	NCAPD2
SCARF2	TREM1	SELPLG	LOXL4	HJURP	CDKN3
ACTA2	MMRN2	COL16A1	GPM6B	KIAA0101	HAUS6
LHFP	LDB2	ETS1	ADAMTSL3	241948_at	KNTC1
FZD1	MEF2C	MYH10	ABI3BP	GGH	CDC45
CLEC11A	GJA1	SNED1	ADAMTS16	RNF114	CNOT10
AKT3	FN1	PLXNC1	ADAMTS3	SPAG5	SAPCD2
ATP8B2	SERPINE1	ITGB1	LCP1	POF1B	C10orf99
TRPS1	NCKAP1L	VCL	ADAMTSL1	HMMR	EIF4E
TUBA1A	SCG2	NID2	KDR	TOPBP1	LLGL2
C3orf80	FMNL3	SIGLEC7	TPSAB1	MRPL48	CCNF
MSN	SELPLG	DPT	PDGFRA	RAB3IP	CENPI
GPR124	ETS1	LILRB2	COL22A1	RFC4	GSPT1
WISP1	PLXNC1	CAV1	RAMP2	LSM5	SPIRE2
SFRP4	ITGB1	PDPN	NTNG2	SLC35G1	SUZ12
HTR2B	VCL	TRO	FGF2	SGOL2	KIF18B
C14orf132	CD248	SPP1	PTX3	PDZD8	DACH1
PRDM6	SSH1	SNAI2	ITGAL	CDK12	RPA3
NAP1L3	CAV1	PTAFR	HAS1	NIF3L1	NUDT15
MGP	PDPN	FOLR2	PAPLN	QTRTD1	RRM1
CYS1	SNAI2	SRGAP2	TNFRSF1A	NDUFA10	NPM1
RCN3	PTAFR	CD4	IBSP	ATAD5	CENPE
MPDZ	SRGAP2	CCR1	FMOD	UBE2T	MECOM
GAS7	PODN	GREM1	RGCC	GEN1	VRK1
SULF1	CCR1	MAP4K4	OLFML2A	MRPS16	YEATS4

PABPC4L	TBXA2R	CD300A	FOXC1	BUB1B	ECT2
ANGPTL2	GREM1	ADAM8	LAMB1	ZBED4	DLGAP5
C1QTNF5	MAP4K4	PLAU	PLEC	DLEU1	USH1C
THBS3	CD300A	NT5E	CRTAP	TTK	PDIK1L
ATP10A	ARHGAP24	LILRB4	NFKB2	CDC25A	SSX2IP
LGALS1	NAV1	ANXA1	MMP13	POLE2	CENPU
FLNA	SRGAP2B	SIRPA	SULF2	CEBPG	CENPM
CLIP4	ADAM8	CYTH3	FLOT1	CNOT1	BTG3
PLN	PLAU	CR1	ADAMTS14	NEK2	RHNO1
LTBP2	SRGAP2C	SIGLEC1	EGFLAM	RAVER2	KIF2C
LAMB2	ANXA1	ICAM1	COL7A1	DIAPH3	CENPV
SYNDIG1	SIRPA	MILR1	PLOD2	FHDC1	MCM4
ARL4C	ANXA6	LRRC4C	APLP1	SPC24	PSMA5
COL8A1	RBFOX2	MCAM	SCUBE3	FAM84A	POLD2
FEZ1	FGF1	TNC	LAMC2	INADL	LIN9
VCAN	SLC7A7	MYADM	GAS6	NOL10	RFC5
PALLD	ICAM1	DCHS1	ADAMTS18	DTL	CDCA5
PXDN	CCL18	COL5A3	ITGA4	SPATA2	POLA1
ZNF532	ADARB1	CDH5	IL6	CLCN3	KIFC1
SYNC	CLEC14A	CNTN1	ICAM2	NCAPG2	ITGB3BP
MMP19	MCAM	CD86	CAPN2	ZNRF2	BCCIP
IGFBP6	MYADM	LRP1	FKBP10	BORA	CHMP4C
AKAP12	FGF7	GSN	PDGFA	LINC01106	PLK1
SPARC	DCHS1	BVES	ANXA2	NSMCE4A	ESPL1
TMEM200B	SOX17	IL4I1	VWA1	HSPA14	PSME3
WBP1L	CDH5	TNFSF13B	ADAMTS9	KIAA1804	TXLNG
LAMP5	DOK2	FYN	P4HA1	KDF1	SASS6
PDLIM4	LRP1	PCDHGA1	DST	RAD54B	MCM3
THY1	PIK3CD	SHC1	ACAN	TMEM106C	STAMBP
COL5A1	BVES	PCDH9	LTBP4	NCAPD3	CUL3
LINC01094	FYN	LYVE1	MMP8	DARS2	PRPF40A
PCDH7	AGTR1	PDGFB	COL21A1	CAAP1	LBR
KANK2	SHC1	TGFB2	ITGA10	ATP2C2	BUB3
GPR176	PDGFB	ITGAV	VIPAS39	MKI67	CENPW
AK021804	CCBE1	CD276	FOXF1	OVOL1	CDK5RAP1
COL5A2	TGFB2	TEK	COLGALT1	MRPL35	NUP43
FAM26E	ITGAV	OBSL1	SPOCK2	KIF9	PARD6B
PMP22	ECM1	VWF	SLC2A10	GPD1L	INTS7
PRRX1	TEK	HAPLN3	MMP7	WDR77	PPP1CC
TSHZ2	ALOX5	SVEP1	AGRN	CCNB1	LIG1

ZNF667-AS1	LRRK2	ALOX5	ADAMTS8	TRMT10C	KIF23
ENTPD1	CMKLR1	ITPKB	CMA1	RPP38	UBE2C
ISM1	ITGB3	OMD	TNFRSF1B	EXO1	NEK4
CRISPLD2	SPI1	SUSD5	MIA	OXNAD1	STRADB
TMEM204	EPHA3	ITGB3	KLK7	NUSAP1	KIF3B
MSR1	NISCH	LIMS2	CTSG	HMGB2	CENPL
CLIP3	DOCK4	IGSF21	COL27A1	LSM3	TRIP13
MYL9	MMP9	SPI1		CEP55	MCM7
LIX1L	TGFBR1	EPHA3		PLS1	FOXM1
SERPINF1	IGF1	SDK1		TOMM70A	TFDP2
GLT8D2	GATA3	PTPRS		STARD7	RPRD1B
MAF	MACF1	PCDH12		KIF20B	NAE1
CALHM2	LAMA2	ITGA7		SLC25A15	CEP85
GPNMB	CCL21	SCN1B		TIMM8A	KIF14
IGFBP7	PLAT	AMIGO2		PAXIP1	POLD3
C1S	ESAM	IGF1		PBK	RAD51
OLFML1	CXCR4	GATA3		CHEK2	CDC123
GPC6	RRAS	MACF1		FAM136A	ANLN
HMCN1	RIN3	LAMA2		TPD52	MYC
DCN	BEX4	CCL21		AIMP1	CENPJ
TIMP1	PRKG1	CADM1		RHPN2	TIMELESS
MFAP5	FSCN1	ESAM		LARS2	IQGAP3
MGC24103	PREX1	CXCR4		SKP2	ESCO2
PRICKLE2	DOCK8	RRAS		C5orf30	RANBP1
SYNPO	ADAMTS1	EDIL3		LRR1	MPLKIP
238151_at	ACVR1	PLAUR		ZNF664	KNSTRN
FSTL1	DOCK10	CD83		SLC35A3	RFWD3
CSF1R	NR2F2	CNTN4		RP11-350F4.2	ZWILCH
S1PR3	CSPG4	PRKG1		TRUB1	CEP57
THBS2	CD34	PREX1		LRRC8B	ROCK2
LMCD1	CCL8	COL13A1		GSTCD	PRC1
LOC101929122	PEAK1	DOCK8		EPHB2	POC1B
MAP1B	CD81	HLA-DPB1		SLC27A2	CKS1B
CTSB	S1PR1	ACVR1		ATP5G1	PCNA
RUNX1T1	PODXL	CD34		DBI	TUBGCP4
CLMP	SLC8A1	CD99L2		OXSM	NEK3
PDGFC	CSF3R	PEAK1		MRPL30	NUP107
MRVI1	FAT3	COL14A1		CCDC47	CCAR1
PTPRM	GNA12	PCDH17		DUS1L	BID
C5AR1	HMOX1	CD81		GIPC2	RANBP2

241473_at	TPBG	TLN1		FBXO45	RAD1
THBS4	FMNL1	S1PR1		CSE1L	TFDP1
TPM2	ITGA1	PODXL		POLQ	PSMA7
FSTL3	TPM1	PCDHB5		MAP2K6	RAD54L
BASP1	PIP5K1C	CSF3R		GINS2	MCM2
C1orf54	DIXDC1	PDLIM5		RBL1	CIT
MAP7D1	ROBO4	FAT3		HADH	PSMA2
LZTS1	CCL7	CD72		AHCY	MAPK13
LMOD1	GPR183	ZFP36L1		DDIAS	CHAF1A
LTBP1	GPM6A	CDH19		MYO5B	BECN1
HSPB2	TIAM1	MFAP4		YAE1D1	ZW10
SYT11	CCR2	CD33		RPS27A	FANCA
TREM2	HSPB1	TPBG		GINS1	ACVR1B
IL1R1	DUSP1	TFE3		PI4K2B	TFAP4
PDGFRB	LGMN	SPON1		E2F1	CKAP5
PEG3	PPM1F	CLEC4A		NOL11	CENPO
ITGBL1	CYGB	ITGA1		242890_at	DONSON
PCOLCE	FERMT3	TPM1		DDX18	PKP4
CILP	FLT4	NID1		SLC38A1	MASTL
FAM101B	SELP	PIP5K1C		ATP8B1	CEP78
ACTN1	RAB13	HLA-DRB1		MTCH2	GPSM2
ARMCX1	SFRP1	SIGLEC10		CDX2	E2F5
DZIP1	HDAC9	FES		ALG14	KIF22
SPSB1	CSF1	JAK3		SUV39H2	NOLC1
TIMP3	ENPP2	NINJ1		UBA2	NUP160
EFEMP1	FOXC2	ROBO4		FRAT2	AXIN2
MEIS1	FLT1	TIAM1		COA3	MCM8
CLIC4	ADORA3	LPP		ORC6	LRRCC1
SPHK1	CD74	CCR2		STIL	CBX3
THBS1	SGK1	PCDHB4		STXBP6	TBCE
COL6A3	CCR5	PRNP		PRELID2	EML4
PDLIM2	PLCG2	ABL2		CCNE2	KIF20A
ZFHX4	ACKR3	HSPB1		EPS8L3	TICRR
RFTN1	PIK3CG	HLA-DPA1		NUF2	POLE
CTGF	LCP1	DUSP1		RPP14	CSPP1
COL6A1	RHOG	CD36		TTC39A	CDCA2
HSPB8	CD99	PPM1F		SERINC5	AURKB
LDOC1	ZMIZ1	HLA-DQB1		MND1	NASP
PRKCDBP	PRCP	FERMT3		COX11	DLG1
ASPN	CITED2	CLDN22		CKS2	KPNB1

ST6GALNAC5	NLRP3	PEAR1	DCLRE1A	CCNE1
CPXM2	GPSM3	SELP	STX3	DIS3L2
215306_at	CLDN5	SFRP1	HSD11B2	ATR
VSIG4	GPC1	ADAM19	CERS6	RAN
GNB4	FAM89B	CSF1	FAM83F	RUVBL1
JAM2	MYO1G	FOXC2	CCNB2	EME1
GPR68	CCL13	SASH3	241466_at	NDC80
242397_at	ARHGDIB	ZYX	BUB1	CHAF1B
MN1	VEGFB	GPM6B	EIF2S2	PRKCA
CD109	STK10	TWSG1	CKAP2	MYBL2
WIPF1	PML	NEGR1	MCCC2	CCNH
PDGFRL	MYH9	CD74	FAM105A	ANAPC7
LEPRE1	KDR	PLEK	OIP5	OVOL2
PTPN14	CX3CR1	ROBO3	SGOL1	WDHD1
IGFBP5	IL16	ABI3BP	RBM15	SLC39A5
MXRA5	PLK2	EMCN	CCDC34	NAT10
FCGR2B	CD63	ACKR3	MCF2L-AS1	SMIM22
TMEM45A	F2R	PIK3CG	236279_at	ZBED3
CYR61	PTPRC	CD99	BCL2L14	SET
LOC440416	ASAP3	ZMIZ1	EZH2	XPO1
EVL	P2RY6	CITED2	FAM195A	EPB41
DCLK1	LPXN	NLRP3	CHEK1	CD2AP
TNFSF4	LMO4	CLDN5	PREPL	E2F7
BTBD19	PDGFRA	MYO1G	CYP2J2	STMN1
ADAMTS12	RDX	TNFAIP8L2	SFPQ	STOX1
LAIR1	CEP85L	EGFL7	1559524_at	GTPBP4
INHBA	NR4A3	SH2B3	FAM117B	SLC9A3R1
ATP2B4	SASH1	LRFN5	PHF20	SETD2
CALU	PTN	NLGN2	POLR1B	NAA50
FAM20A	HGF	EMILIN2	CENPH	CDC23
SYNE1	S100A8	ARHGDIB	SKA3	POLDIP2
NRP1	ADD2	PGM5	CCL15	MZT1
1558803_at	NTNG2	SCARF1	MYRIP	SKA1
PILRA	SELL	HLA-DMB	HELLS	CEP70
TIE1	GLUL	GPR4	ARFGEF2	AURKAIP1
SLC11A1	STC1	PRPH2	SMAGP	RPS6KA3
RP3-428L16.2	FGF2	ADAMTSL1	VIL1	HUS1
NKX3-2	ITGAL	STK10	CFTR	TXNL4A
COL18A1	HAS1	MAP3K8	GRHL2	NUP205
C1orf162	DAB2	PSTPIP1	ESRRA	PKMYT1

228202_at	LEF1	PML		DTYMK	PDCD2L
P2RX7	PLVAP	MYH9		GTSE1	CCNC
CHST15	JUP	KDR		EXOC6	CASP3
IGFBP3	CHRD	CX3CR1		GCSH	NLE1
QKI	CCL3	FBLN7		ABCC6	CETN3
PSAP	NOG	CD63		ACOT11	DCLRE1B
KCNMB1	MAPRE2	IL1RAP		UQCC1	PSMD14
CALD1	BMPR2	PPFIA2		NCAPG	NUP98
SPG20	VAV1	PTPRC		DCTPP1	PRIM2
PDE1A	SEMA4C	LPXN		SLC5A1	HDAC8
MYLK	RGCC	PDGFRA		DANCR	ZNF830
SMARCD3	SOX18	RDX		AUNIP	RAD50
MARCO	PLA2G7	IL6ST		PRMT3	CENPQ
PKD2	MAGI2	NR4A3		MRPS33	USP39
STOM	LIMCH1	ADAM23		MRPL45	SOX9
TRPC1	IQSEC1	PTN		DKC1	SMC1A
OLR1	FOXC1	CD209		NEDD4L	SETMAR
VAT1	CCL14	S100A8		FASTKD2	CCAR2
CACNA2D1	LAMB1	ADD2		CORO2A	TIPIN
LOC100132891	OR51E2	NTNG2		KIF18A	RIOK2
C5orf46	RAP2B	SELL		E2F2	RAD21
SDC2	ZNF304	NOTCH4		DEPDC1	TUBG1
LAMA4	CCL4	ITGAL		PLAGL2	SMC5
ARHGEF17	MCTP1	HAS1		FANCI	TRIAP1
CTSL	ELMO1	GBP1		CNOT11	SLC2A8
FAM110B	FAM107A	LEF1		CDT1	CDC20
CLDN11	CD48	CORO2B		PTRH2	TTLL12
FAM129A	CCL19	LIMS1		ESRP2	APAF1
TENM4	SDC3	JUP		PLA2G12A	PSMD12
ITGA11	HIF1A	CHRD		RMI2	DNM2
COL11A1	ANGPT1	IBSP		CENPN	RBBP7
PPP1R3C	SEMA3G	VAV1		DDX52	KATNB1
GYPC	GRN	C1QTNF1		AVL9	CEP152
EVC2	GLG1	SIGLEC5		SPAG1	PPM1D
CTHRC1	PHACTR1	KIFC3		CMAS	NSUN2
PLXND1	ADIPOQ	RGCC		FARSB	PER2
LRRC32	PLXNA4	HLA-DRA		SLC12A2	MCTS1
UBE2E2	CNN2	LIMCH1		MSH2	CDK7
GPX8	NDEL1	PPFIBP1		UTP18	RNASEH2B
LATS2	MYOCD	FZD7		PREP	CDK13

FCGR1A	CORO1C	IL1RN	CDC6	RRS1
1556138_a_at	EGR3	IL4R	KIF4A	MSH3
SSPN	RAC2	PCDHB15	TLDC2	NABP2
FIBIN	RUFY3	LAMB1	SNORA5B	TUBD1
CD14	ROBO1	ACTB	GART	TLK2
HIC1	TNFAIP3	RAP2B	C16orf91	POC5
HTRA3	MEGF10	CCL4	NDUFS1	DDX11
CNN1	LRP12	ZBTB16	CYCSP33	NUP85
WWTR1	CDC42BPA	PDCD1LG2	LOC101928747	HOXA13
MRGPRF	BTG1	CHL1	CISD3	SKA2
NOTCH3	LMNA	FAM107A	TMC5	CNOT6L
BCAT1	TUBB2A	CCL19	INIP	RAE1
PLA2G5	LPAR1	ANGPT1	MELK	CSNK2A1
CHST3	BST1	LSAMP	ABCE1	TUBGCP3
ADAMTS2	PDE4B	FLOT1	LOC100289019	ASNS
COL1A2	CD274	HLA-E	LOC157860	NUP155
COL3A1	GLI1	PCDHB16	GPR160	POLA2
IQCJ-SCHIP1	NTN1	EGFLAM	BOLA3	HNRNPU
DACT1	CD200	ADIPOQ	AK6	TTC19
TPST1	CD40	PLXNA4	A1CF	CEP72
PHLDB2	BST2	SOCS5	ASPM	COPS5
EVA1B	CCL5	EFNB3	NUDT16P1	TACC3
EGR2	SUN2	CORO1C	MRPL42	HEPACAM2
LUM	SWAP70	EGR3	NLN	IPO7
PNMAL1	CXCL16	DLG4	SVIP	PHC3
RAI14	LAMC2	RAC2	SRSF3	RAD51C
FBXO32	GAS6	CDON	BRCA2	DBF4B
VASH1	ITGA4	ROBO1	HSD17B12	CCNJ
C3AR1	SELE	MEGF10	ZNF443	RFC2
LAPTM5	KRT16	NINJ2	TSEN2	E2F4
LOX	IL6	CDH6	AP1M2	CSNK1A1
CFL2	TNFSF14	SIRPB1	ARHGAP8	SUV39H1
CHSY3	CCL23	CHST10	NDFIP2	UHRF1
TYROBP	RIN2	SORBS1	NOP58	USP37
FBLN2	CAP1	COL7A1	EARS2	RCC1
PTGER3	LHX6	BST1	CMTM4	TCF3
RECK	ABI3	ADORA2A	PIGL	MTBP
CCDC88A	FER	CD274	TIMM13	NUP88
FST	RHOB	CSTA	MTFR2	LIN54
SPOCD1	RELN	NTN1	PPAT	RECQL5

LY86	SP100	CYTIP	MYO19	AHCTF1
HSPB7	ARSB	APLP1	ANKS4B	IPO5
SPATS2L	ENPEP	CD200	231606_at	PSMD3
ITGB2	CDH13	CCL5	JPH1	SUMO1
MMP14	DUSP22	CD80	PRPF38A	CNOT6
RASSF2	DLG5	HOXD3	ALG6	E2F3
FCGR1B	CRTAM	SWAP70	ECE2	TACC2
BMP1	BDKRB1	LAMC2	TFCP2L1	CHMP4B
241780_at	CORO6	GAS6	PTTG1	PAK4
LOC728392	RHOH	ADAMTS18	PLCH1	RINT1
ADAP2	SEMA6B	ARPC2	COA6	CDC7
DNM3OS	DUSP3	ITGA4	SMIM15	FANCM
NEXN	PDGFA	PCDHB6	MINA	FZD3
TWIST1	PKN1	SELE	ZNF91	PSRC1
SRPX	IL10	IL6	DSCC1	BRD7
SGCD	SMAD7	TNFSF14	DLD	MSH5
SLIT3	ZFAND5	ICAM2	UMPS	ORC3
MOXD1	PLP1	RIN2	HDHD3	TXNL4B
APOE	ARHGEF2	UBASH3B	235363_at	CLSPN
ENG	CYP7B1	ZFHX3	SLC25A10	ZNF703
CRISPLD1	MIR22	FER	MSI2	PDS5A
C16orf45	NBL1	DTX1	FBXO5	PSMD6
FLRT2	IGF1R	FGL2	GALE	SPAST
AP1S2	SNAI1	MYOT	REPIN1	CBX5
LAMC1	DUSP10	RHOB	CXADR	ANAPC10
LOC100996724	ARID5B	RELN	DNAJC9	CCNT2
ITPRIP	GRB10	TGM2	MRPS9	SRPK2
DLC1	PPARD	SIGLEC9	AMMECR1	SMPD3
AIF1	TTBK2	CD28	CCDC59	CCNB1IP1
MMP2	ADAMTS9	COL6A6	SLC22A5	RSPH1
C10orf128	DISC1	PCDHB10	ORC1	GAS2
GABARAPL1	MYO1C	CDH13	AK022793	PTPN11
ARHGAP31	FGF10	CBLB	BZW2	PCID2
INMT	NEDD9	DUSP22	AIM1	CNTD1
CFH	NDRG4	DLG5	GLCE	NUDT16
BICC1	CORO1A	GP1BB	HNRNPA2B1	HAUS8
MCC	DAPK3	CRTAM	FGFR1OP	UBE2S
SLAMF8	S100A9	CLEC4G	ARIH2	CHTF18
MIR143HG	WDPCP	EFNA5	ATP5G3	APPL2
FBLN5	PAK3	RHOH	BLM	PTP4A1

TNFSF12	CX3CL1	CEBPB		AIFM1	CEBPA
JPH2	S100A11	DUSP3		TJP2	SEH1L
TP73-AS1	SEMA3E	ANXA2		242053_at	PIWIL2
TCF4	STAT5A	IL10		ATAD2	AATF
TENM3	SOX10	RUNX3		TPRN	TUSC2
DSE	RHOC	SMAD7		H2AFZ	PSMA3
ST3GAL6	LYST	FBLIM1		GFM1	TUBA4A
MLLT11	PPP2R3A	PCDHGA3		FIGNL1	DYNLL2
GLI2	SEMA7A	DUSP10		CDCA3	TUBB4B
ANKRD6	FPR2	PPARD		LIG3	ANAPC5
BCL6	GCNT2	ADAMTS9		MRS2	PRMT1
NOTCH2	DDX58	DISC1		ORC2	ARID3A
PECAM1	RHOD	NEDD9		CHCHD3	NEDD1
SLC1A3	AMOTL2	CYTH1		CHD7	MSH6
ENOX1	CCL22	NLGN4Y		CDCA8	DMC1
CYTH4	TRADD	DST		GINS3	CSNK2A2
GNAI2	CD2	PCDHGA10		NUP35	KHDRBS1
DENND5A	TNFRSF18	SIPA1		CASC5	OR2A4
JAZF1	NDE1	TNIP1		ATP10B	TERF1
MYO5A	PLXNA1	CORO1A		TSEN54	PKN2
FZD4	PGF	DAPK3		PAWR	PPP5C
TNFAIP6	WASF2	S100A9		ZC3H15	
MEDAG	TP53INP1	WDPCP		CENPF	
CD59	CCL26	PCDHA1		SLC25A33	
SH3PXD2A	CTNNA3	PAK3		GPA33	
POSTN	ARF4	CX3CL1		PRLR	
CTSK	FOXF1	S100A11		CCL20	
SELM	STAT5B	SEMA3E		PHOSPHO2	
AHNAK2	GPX1	ACAN		MRRF	
LINC00702	ADORA1	MUC16		FEN1	
TMEM47	SRPX2	PRTG		ERCC6L	
NAV3	S1PR2	ATP2A2		AK2	
TM6SF1	TNN	FPR2		ATP5F1	
NRK	S100A12	PCDHB12		COX5A	
LOXL2	SEMA4A	GCNT2		TTF2	
RBMS3	MMP7	PCDHB11		NUP37	
KLC1	HRH1	BCAM		LNX1	
CLEC5A	SOD2	NPTN		BIRC5	
UNC5B	ZAP70	EPHB6		ACSM3	
CSRP1	STX4	RASGRP1		FANCD2	

PDE4DIP	CXCL9	RAPGEF1		ACSL5	
GUCY1B3	SMURF2	IL2RA		ACTL6A	
40787	PTPRU	RHOD		SMC4	
SPARCL1	CXCL10	CD2		NEIL3	
C8orf88	PLXNB3	TNFRSF18		PHBP8	
NLGN4X	RTN4	BTN2A2		239832_at	
RP11-134G8.8	MERTK	ITGA10		GRPEL1	
CD163	RHOA	PLXNA1		TADA2A	
TCEAL7	CXCL13	RARA		RAD51L3-RFFL	
PINLYP		SORBS3		PXMP2	
F13A1		CTNNA3		MCM6	
HSD17B6		CDKN2A		EHF	
RARRES2		FOXF1		PXMP4	
CCL2		STAT5B		CAMSAP3	
FCER1G		SPOCK2		C9orf41	
LOC100996668		PIK3R6		POLR2D	
NFATC1		SLITRK3		NOL4L	
TLR2		MSLN		ORC4	
CPZ		SRPX2		NCAPD2	
TWIST2		TNN		CMSS1	
ARSI		NRXN1		HMGCR	
HS3ST3A1		NCAM2		DNAJC19	
CD93		IL7R		DLAT	
GLRB		BMP6		PDE12	
ITGAX		ZAP70		FASTKD1	
NR3C1		STX4		PDHA1	
TGFB1		NLGN1		SLC35B3	
PPM1M		PCDHB13		TMPRSS2	
APBB1IP		CNTNAP3		CDH17	
232544_at		PCDHB2		MCAM	
FABP4		PTPRU		IDH1	
38231		CD70		CCDC125	
ZBTB4		PLXNB3		SLC25A5	
ILK		PCDHB3		USP7	
DKK3		RTN4		CDKN3	
LOC100130872		FAT4		LINC00511	
SH3BP5		MERTK		CHCHD4	
STAB1		PPP1R12A		PEX7	
NDN		TIGIT		ORC5	
GLIPR2		RHOA		MANSC1	

PARVA		NFASC		TMEM45B	
HCFC1R1		CXCL13		BRIX1	
PXDC1		RSU1		DDX31	
THEMIS2				PAIP1	
TRPV2				GSS	
LOC101929304				RP11-498C9.15	
KIF26B				HAUS6	
THBD				CPSF6	
VSTM4				PEX1	
TMEM140				SLC25A13	
LOXL3				LINC00543	
PIP4K2A				COA7	
CD84				KNTC1	
ZBTB47				CDC45	
GADD45B				CHDH	
ERG				L2HGDH	
GALNT15				PCBD2	
LY96				KLF5	
ACTC1				ZNRF3	
LINC00312				CNOT10	
NEFH				POLR3K	
VCAM1				PMM2	
NCF2				POC1A	
DPEP2				GFPT1	
ZNF469				TCAIM	
EBF2				GALNT3	
KCNJ8				KPNA2	
RHOJ				LYAR	
SERPINH1				LRRC8D	
C3				UBE3D	
SLC39A13				CPSF3	
CCDC8				FGFR4	
ROR2				238822_at	
244579_at				LMNB1	
FUT11				ERLIN1	
KLF2				UGT8	
MRC1				C1orf112	
KCNJ15				SAPCD2	
KCND2				LRBA	
FCN1				INTS2	

SGCE	LTV1
ARMCX2	C10orf99
238592_at	NT5DC1
UCHL1	EEF1E1
HSPG2	EIF4E
DOK3	SLC35D1
NR2F1-AS1	LLGL2
NALCN	CCNF
ITGB5	FOPNL
ZNF677	CENPI
PIEZO2	ASF1B
CXCL12	SERBP1
CYBRD1	METTL5
ELK3	HDAC1
244740_at	TMEM170A
FBLN1	COIL
ADCY2	IARS
OSCAR	TFB2M
KIRREL	NDUFAB1
HEYL	STARD10
DIP2C	FAM135A
CLEC7A	NIT2
PLD3	MRPL50
ZNF667	TP53RK
CNTNAP1	ZC3H8
VAMP5	SELENBP1
COL15A1	LOC101060264
ANXA5	GSPT1
LOC100506119	SPIRE2
PLA2G4C	TINAG
233090_at	TAMM41
SYNPO2	COQ7
ADAMTSL4	CACNA1D
TCEAL3	DUSP16
ARHGEF6	SUZ12
235759_at	LOC101928881
A2M	METTL2A
FAM49A	DAZAP2
PARVG	NOL7
C1QTNF3	KIF18B

CLU				DACH1		
RNF150				RPA3		
RGS16				SARS		
SAMD4A				THUMPD3		
FGR				BSPRY		
COL1A1				ITGA6		
STAT2				LSM4		
HAVCR2				RBM12		
HCK				GMDS		
RHOQ				ILVBL		
MPRIP				NUDT15		
MAP3K3				PARM1		
MEOX2				RRM1		
229327_s_at				NPM1		
DPYD				C2orf15		
COL4A2				ZDHHC6		
LRRC15				PIGU		
CHST1				NOP16		
APOD				CENPE		
TREM1				LRRC16A		
ALDH1A3				ADAM1A		
MMRN2				ING5		
PPAPDC3				NHEJ1		
PRR24				SEL1L3		
DAAM2				WHSC1		
EBF1				TRABD2A		
SOX11				PIGF		
HOXB2				ATP5C1		
LDB2				RPE		
RASL12				MECOM		
FRMD4A				NCBP1		
PCDHB7				ST14		
KIAA0247				230752_at		
SUGCT				LINC01184		
PHLDA3				WDR43		
NUDT11				CLDN3		
LOC100507165				MUC13		
MEF2C				C4orf29		
COL24A1				ANKRD22		
SLC15A3				VRK1		

MEIS2				TMEM161B	
GJA1				DDX21	
FN1				FANCF	
FAM229B				PTGES3	
KIF1B				ETNK1	
SERPINE1				240180_at	
RGS4				DDAH1	
NCKAP1L				DHX15	
ARL10				PPARA	
CHRNA3				CHPT1	
COL12A1				VAPB	
SCG2				YEATS4	
233036_at				DHRS11	
TTC7B				ZNF57	
ALOX5AP				GMCL1	
LTBP3				ECT2	
RASSF8				PPA1	
FMNL3				C9orf40	
NAGK				USP10	
SELPLG				DLGAP5	
MMP16				RSL1D1	
FPR3				MCCC1	
TMEM119				MIOS	
SNX18				THOC7	
COL16A1				241458_at	
MCEMP1				LOC100506538	
MFAP2				USH1C	
ETS1				METTL15	
236764_at				PDIK1L	
MYH10				KIAA1524	
MS4A4A				MRPS23	
				MAGOH	
				NCR3LG1	
				SSX2IP	
				RP1-193H18.2	
				OLA1	
				CSTF1	
				GNL3	
				SFXN2	
				SS18L2	

				FITM2		
				CASP5		
				XRCC6BP1		
				RP11-1094M14.11		
				MMACHC		
				CENPU		
				CENPM		
				BTG3		
				RHNO1		
				GAR1		
				B3GNT3		
				MRPS25		
				CYP39A1		
				KIF2C		
				CASP8		
				DDX20		
				CENPV		
				MCM4		
				PSMA5		
				CLCN2		
				ETS2		
				POLD2		
				IFRD2		
				GJA9		
				UCHL3		
				LSM6		
				213598_at		
				ICT1		
				C12orf66		
				HNRNPAB		
				STRA13		
				SH3YL1		
				LIN9		
				SLC37A4		
				ABCB10		
				DSG2		
				MAPKAPK5-AS1		
				FH		
				FAM134B		
				RFC5		

				CDH1	
				PSMG1	
				DUT	
				ZBTB24	
				DPM1	
				MGST2	
				CTBP2	
				MRPL37	
				TRMT61B	
				CCT2	
				LRRC1	
				CDCA5	
				PPFIBP2	
				POLA1	
				PARS2	
				TRAP1	
				COQ3	
				GJA9-MYCBP	
				LARP4	
				PLEKHG6	
				TSPAN6	
				KIFC1	
				DDX39A	
				FAM173B	
				VAV3	
				MCM10	
				TCFL5	
				ITGB3BP	
				MRPS17	
				CCDC138	
				NSG1	
				BCCIP	
				CHMP4C	
				PLK1	
				PALB2	
				F2RL1	
				TFB1M	
