# Supplementary material for: A prognostic six‐gene expression risk‐score derived from proteomic profiling of the metastatic colorectal cancer secretome
Source: J Pathol Clin Res. 2022 Sep 22;8(6):495–508. doi: 10.1002/cjp2.294 (PMC9535096; doi:10.1002/cjp2.294)
Supplement: Supplementary file 9 — Table S7. HRs (95% CI) of high‐ and low‐risk patients classified by stages in survival analyses [file CJP2-8-495-s002.pdf]

Table S7. Hazard ratios (95% CI) of high and low risk patients classified by stages in survival analyses.

		All stages		Stage I		Stage II		Stage III		Stage IV	
		HR (95% CI)	p-value	HR (95% CI)	p-value	HR (95% CI)	p-value	HR (95% CI)	p-value	HR (95% CI)	p-value
GSE17538	OS	2.10 (1.30-4.08)	0.00251	1.68 (0.23-12.5)	0.607	6.20 (1.97-19.55)	0.00183	2.32 (1.07-5.04)	0.038	1.23 (0.65-2.32)	0.519
	DSS	2.42 (1.18-4.96)	0.0154			3.26 (0.78-13.68)	0.106	3.95 (1.50-10.40)	0.00537	1.46 (0.62-3.45)	0.385
TCGA COADREAD	OS	2.11 (1.41-3.15)	0.00026	9.16 (1.67-50.29)	0.0108	1.54 (0.70-3.50)	0.3	2.83 (1.39-5.76)	0.00423	0.89 (0.39-2.01)	0.777
	DSS	2.4 (1.37-4.23)	0.00232			4.33 (0.86-21.86)	0.0756	4.44 (1.5-13.1)	0.007	0.77 (0.33-1.82)	0.557
	PFI	2.02 (1.39-2.93)	2.00E-04	3.39 (0.57-20.38)	0.182	2.41 (1.23-4.73)	0.0103	1.94 (0.98-3.87)	0.0587	1.07 (0.56-2.05)	0.828
GSE39582	OS	1.99 (1.45-2.73)	2.08E-05			1.29 (0.73-2.26)	0.381	2.3 (1.39-3.82)	0.00117	1.19 (0.63-2.26)	0.592
	RFS	2.08 (1.5-2.88)	1.19E-05			2.22 (1.26-3.94)	0.00604	1.39 (0.84-2.29)	0.196	1.12 (0.57-2.2)	0.745
